# Long-Chain *S*-Acylation Is a Key Modulator During the Macrophage Inflammatory Response

**DOI:** 10.1016/j.mcpro.2026.101600

**Published:** 2026-06-10

**Authors:** Anneroos E. Nederstigt, Samiksha Sardana, Francine Rodrigues Ianiski, Marc P. Baggelaar

**Affiliations:** 1 Biomolecular Mass Spectrometry and Proteomics, Bijvoet Center for Biomolecular Research and Utrecht Institute for Pharmaceutical Sciences, University of Utrecht, Utrecht, The Netherlands; 2 Netherlands Proteomics Center, Utrecht, The Netherlands

**Keywords:** long-chain *S*-acylation, macrophage activation, inflammation, 2-bromopalmitate, Palmostatin B, secretion

## Abstract

Long-chain *S*-acylation is a reversible lipid modification critical for regulating protein localization, stability, and signaling, yet its role in macrophage-mediated inflammation remains incompletely understood. Here, we combine stable isotope labeling by amino acids in cell culture (SILAC) with site-specific acyl-biotin exchange (ssABE) to generate a comprehensive map of the long-chain *S*-acylation landscape in THP-1 macrophages polarized to M0 and M(LPS + IFNɣ) states. Our quantitative proteomics reveal polarization-specific *S*-acylation patterns and uncover numerous inflammation-related modification sites, including novel *S*-acyl-peptidoforms, which are distinct *S*-acylated variants of the same peptide such as WARS (C305/C309), highlighting their potential relevance in macrophage activation. Pharmacological inhibition of *S*-acylation with the broad-spectrum inhibitor 2-bromopalmitate suppresses secretion of key pro-inflammatory chemokines (CXCL9, CXCL10, and CCL4) and disrupts IDO1-mediated tryptophan catabolism, while Palmostatin B mainly stabilizes *S*-acylation on GPCR signaling proteins. Together, these findings position long-chain *S*-acylation as a key regulatory mechanism during macrophage activation and a promising target for therapeutic intervention in inflammatory disease.

## Highlights

• Quantitative strategy for the site-specific analysis of long-chain *S*-acylation.
• Upregulation of long-chain *S*-acylation during macrophage polarization.
• Distinct long-chain *S*-acylated peptidoforms identified.
• Possible link between chemokine secretion and long-chain *S*-acylation.

## Graphical Abstract

**Figure:**
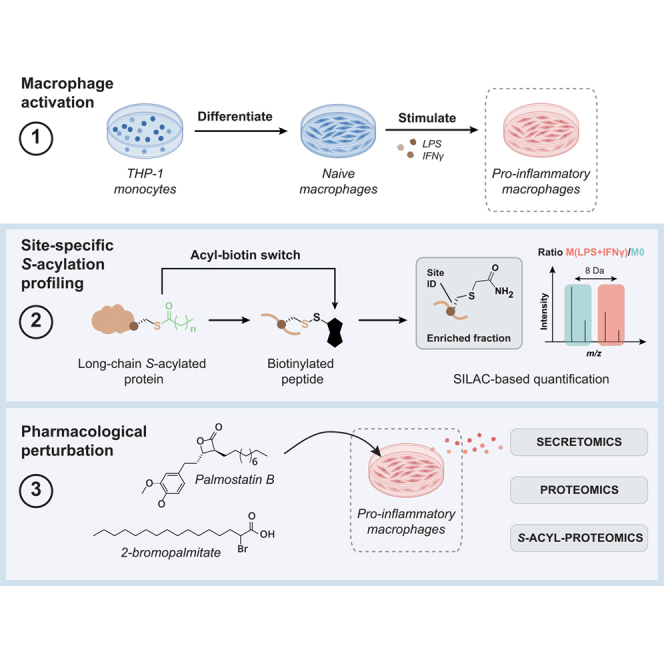

## In Brief

Long-chain *S*-acylation is a reversible lipid modification that regulates protein function, yet its role in the macrophage inflammatory response is poorly understood. By using a site-specific acyl-biotin exchange strategy, we mapped long-chain *S*-acylation of the macrophage inflammatory response in THP1 cells, uncovering polarization-specific patterns and novel *S*-acyl peptidoforms. Inhibition of long-chain *S*-acylation suppressed pro-inflammatory chemokine secretion (CXCL9, CXCL10, CCL4) and disrupted IDO1-mediated tryptophan catabolism. These findings point to long-chain *S*-acylation as a key regulatory axis in macrophage activation and a potential therapeutic target in inflammatory disease.

Protein lipidation is a hydrophobic post-translational modification (PTM) that plays a crucial role in cellular processes such as protein–protein interactions, protein stability, signaling pathways, and protein secretion (1, 2, 3). Among all types of protein lipidation, long-chain *S*-acylation is one of the most abundant in the human proteome, and there is evidence that >10% of human proteome can be *S*-acylated (4). Long-chain *S*-acylation occurs when a fatty acid forms a thioester bond with a cysteine residue of a protein. Unlike other fatty acid modifications, which are irreversible, long-chain *S*-acylation is enzymatically regulated (5, 6, 7). Twenty-three ZDHHC *S*-acyltransferases catalyze the attachment of fatty acids to proteins, while eight deacylating enzymes (APT1, APT2, ABHD10, ABHD17A-C, ABHD13 and ABHD16A) catalyze their removal (8, 9, 10, 11, 12, 13). *S*-acylation is therefore a dynamic process that can be fine-tuned to meet cellular needs at any given moment.

Direct detection methods for achieving in-depth coverage of the long-chain *S*-acyl-proteome are not readily available due to the hydrophobic nature of long-chain *S*-acylation, its sub-stoichiometric levels of occurrence, and the lack of specific enrichment methods (14). Consequently, indirect protein-based detection methods dominate the field. These methods fall into two categories: (1) lipid-centric lipid metabolic labeling and (2) cysteine-centric acyl-biotin exchange (ABE) strategies (15, 16, 17). In the latter, cells or tissues are lysed, and all free cysteines are capped with an alkylating reagent such as *N*-ethylmaleimide or iodoacetamide (18, 19). Thioester bonds are then selectively cleaved with neutral hydroxylamine, after which newly released thiol groups are labeled with a thiol-reactive biotinylation reagent. *S*-acylated proteins are thus converted to biotinylated proteins, which can be enriched by streptavidin affinity purification and identified by mass spectrometry (MS) (20, 21, 22).

While protein-based ABE is effective for detecting *S*-acylated proteins, it lacks the resolution required for detailed site-specific information. Site-specific information is crucial because many long-chain *S*-acylated proteins contain multiple long-chain *S*-acylation sites, which may be occupied with different stoichiometries (23, 24, 25). Several site-specific ABE (ssABE) strategies have recently been developed that enrich biotinylated peptides rather than biotinylated proteins (19, 25, 26, 27). However, the field is still in its infancy, and robust strategies that enable accurate relative quantification of long-chain *S*-acylation sites are highly desired.

Macrophages utilize long-chain *S*-acylation to regulate various pathways, including cyto- and chemokine signaling as well as NOD and Toll-like receptor signaling (28, 29). Dysregulation of long-chain *S*-acylation has been implicated in numerous inflammatory disorders, such as inflammatory bowel diseases (IBD; Crohn’s disease, ulcerative colitis) and liver inflammatory diseases (alcoholic steatohepatitis, NAFLD) (30, 31, 32, 33, 34, 35). Several key proteins involved in these disorders undergo long-chain *S*-acylation at multiple cysteine residues, such as STAT3 (32), NOD1/2 (31, 35), MYD88 (36, 37), the NLRP3 inflammasome (30, 38, 39, 40, 41), and notably, Gasdermin D (42, 43, 44, 45). While the importance of long-chain *S*-acylation in inflammation is recognized on a protein-by-protein basis, few studies have explored its full scope in human inflammatory macrophages (46, 47). Moreover, the dynamic nature of long-chain *S*-acylation, coupled with the presence of multiple *S*-acylation sites within the same protein, suggests that macrophages may use distinct *S*-acyl-proteoforms, proteins with diverse patterns of site occupancy, to respond to different stimuli (48). However, the landscape and functional significance of these *S*-acyl-proteoforms in inflammatory macrophages remains largely unexplored. A comprehensive understanding of site-specific long-chain *S*-acylation in macrophages during inflammation could pave the way for novel therapeutic strategies to treat inflammatory disorders.

In this work, we address the need for (1) robust, quantitative, site-specific strategies to detect *S*-acylation across diverse biological contexts and (2) a comprehensive site-specific map of the dynamics of long-chain *S*-acylation during the macrophage inflammatory response. We utilized THP-1 cells as a human macrophage model within a quantitative proteomics framework, leveraging Stable Isotope Labeling by Amino Acids in Cell Culture (SILAC). A SILAC-based ssABE workflow was implemented to analyze differentially activated THP-1 macrophages. We first examined changes in the whole proteome. Then, we analyzed site-specific changes in the *S*-acyl-proteome and extended our investigation to assess the presence of distinct *S-*acyl peptidoforms, with the goal of defining the prevalence and distribution of long-chain *S*-acylation across the macrophage-mediated inflammatory response. Finally, we examined the effects of 2-bromopalmitate, an inhibitor of ZDHHC *S*-acyltransferases, and Palmostatin B, an inhibitor of long-chain de-*S*-acylases, on the proteome, *S*-acyl-proteome, and secretome of inflammatory macrophages.

## Experimental Procedures

### Cell Culture

THP-1 cells (ATCC, TIB-202, obtained from the Vidarsson lab stock at Sanquin) with a passage number below 20 were cultured in “full RPMI” medium consisting of RPMI-1640 (Capricorn, RPMI-STA) supplemented with 10% fetal bovine serum (FBS, Gibco, A5256701), 100 U/ml penicillin/streptomycin (pen/strep, Gibco, 15140-122). THP-1 cells were kept at concentrations between 3e5-1e6 live cells/ml in standing culture flasks (Greiner). All cells were grown in a humidified atmosphere, 5% CO_2_ and 37 °C. Cells tested negative for *mycoplasma* by PCR.

### SILAC Labeling

THP-1 cells were cultured in suspension in full “Heavy” ^8^K^10^R medium consisting of RPMI-1640 Medium for SILAC (Thermo, 88365) with 10% dialyzed FBS (Gibco, 26400-044), 100 U/ml pen/strep, 0.453 mM ^13^C_6_^15^N_4_ L-arginine: HCl, 99% (Cambridge Isotope Laboratories #CNLM-539-H) and 0.524 mM ^13^C_6_^15^N_2_ L-lysine: HCl, 98% (Silantes, 211603902). For “Light” ^0^K^0^R medium, medium was supplemented with L-arginine:HCl (Sigma, A6969) and L-lysine: HCl (Sigma, L8662). Aliquots of “heavy” cultured cells were isolated after a minimum of six doublings to check heavy isotope incorporation by MS. These aliquots were harvested by centrifugation at 150*g* for 10 min at 4 °C. Resulting pellets were washed twice with ice-cold PBS, with centrifugation at 300*g,* 10 min, 4 °C in between each wash. Resulting pellets were flash-frozen using liquid nitrogen and stored at −80 °C until further use. Cells had >98% heavy label incorporation (Supplemental Fig. S1).

### THP-1 Differentiation

#### Label-free

M(LPS + IFNɣ) differentiation protocol by Baxter *et al.* (2020) was followed with some minor adjustments (49). Cells were resuspended in full RPMI medium supplemented with 5 ng/ml phorbol 12-myristate 13-acetate (PMA, Sigma P8139) and seeded at concentrations of 3e5 live cells/ml. Cells were incubated for 24 h, washed thrice with phosphate-buffered saline (PBS, Capricorn, PBS-1A) and rested in full RPMI medium without PMA for 72 h. Cells were washed twice with PBS. THP-1 M0 macrophages where then stimulated with 20 ng/ml IFNɣ (Thermo, AF-300-02) and 250 ng/ml LPS (Sigma, L5293), or PBS in full RPMI for 48 h to acquire proinflammatory M(LPS + IFNɣ) and M0 THP-1 macrophages respectively. Cells were harvested by scraping. Cells were washed twice with ice-cold PBS, with centrifugation at 150*g* for 10 min at 4 °C following the first wash and at 300 *g* for 10 min at 4 °C following the second wash. Pellets were flash-frozen with liquid nitrogen and stored at −80 °C until further use. Two M(LPS + IFNɣ) and three M0 replicates were produced from two independent experiments.

#### SILAC

THP-1 differentiation was performed as in the label-free experiment with some adjustments. 1.02e7 heavy or light cells were resuspended in heavy or light PMA-containing full RPMI for SILAC medium. Cells were seeded in eight T175 flasks at 3e5 live cells/ml (four heavy, four light flasks). Cells were rested in heavy or light full SILAC medium for 72 h. THP-1 M0 macrophages were then stimulated with 20 ng/ml IFNɣ and 250 ng/ml LPS (two heavy, two light flasks), or PBS (two heavy, two light flasks) for 48 h to acquire proinflammatory M(LPS + IFNɣ) and M0 THP-1 macrophages, respectively. Cells were harvested as described in the label-free differentiation experiment. Four heavy and four light replicates were produced from two independent experiments.

### ZDHHC or APT inhibition Assay

8.5e6 cells were seeded at a concentration of 3e5 cells/ml in 30 T75 flasks (5 flasks per replicate). THP-1 cells were differentiated label-free to M(LPS + IFNɣ) polarized macrophages following the protocol outlined in “THP-1 differentiation”, with minor adjustments. Cells were stimulated with 20 ng/ml of IFNɣ and 250 ng/ml LPS for 3 h instead of 48 h. After 3 h, the medium was removed, and the cells were washed once with warm PBS and twice with warm phenol-red-free RPMI-1640 medium (Capricorn, RPMI-XRXA). Then, 5 ml of secretomics medium (serum, antibiotic, and phenol-red-free RPMI-1640) supplemented with either 50 μM 2-bromopalmitate (2-BP), 10 μM Palmostatin B (PalmB) or 0.1% DMSO, and 2 mM L-glutamine was added. Cells were incubated for 75 min (2-BP) or 2 h (PalmB) at 37 °C and 5% CO_2_. For each replicate, supernatants from five flasks were pooled in two 15 ml tubes. 1x complete EDTA-free protease inhibitor cocktail (Roche, 11836170001) was added, after which supernatants were centrifuged at 1000 rpm, 4 °C for 5 min to remove cell debris. Supernatants were then passed through a 0.22 μm PES filter into new 15 ml tubes. Secretomes were flash-frozen with liquid nitrogen and lyophilized for ∼48 h. Adherent cells were harvested with 5 ml of ice-cold PBS using a scraping. Cells were centrifuged for 10 min at 4 °C and 150 *g*. Supernatants were aspirated to remove dead cells. Pellets were washed twice more with ice-cold PBS and centrifuged for 5 min, 600 *g*, and 4 °C. Remaining pellets were flash-frozen and stored at −80 °C until further use.

### SILAC and Label-Free ssABE

#### Protein Extraction, Reduction, and N-Ethylmaleimide Alkylation

Cells were lysed with 50 μl of lysis buffer (50 mM TEA pH 7.5, 10 mM NaCl, 2 mM MgCl_2_, 0.5% (w/v) NP-40, 0.2.% (w/v) SDS, 1x complete EDTA-free protease inhibitor cocktail, 62.5 U/ml benzonase (EMD Millipore, 70746) and 500 nM PalmB (Sigma, 178501) and incubated for 10 min at RT and 800 rpm. The lysates were diluted with one volume of 50 mM TEA pH 7.5, 10 mM EDTA, 6% (w/v) SDS and three volumes of 50 mM TEA pH 7.5, 10 mM EDTA, 3% (w/v) SDS. The protein concentration was determined by bicinchoninic acid assay (Thermo, 23227). 450 μg (900 μl) of heavy lysate and 450 μg (900 μl) of light lysate were combined (*n* = 4 biological replicates). Samples were reduced with 200 μl 500 mM TCEP at RT, and 800 rpm for 30 min. Samples were then alkylated with 65 μl of 1 M *N*-ethylmaleimide (NEM, Sigma, 128287) and incubated at RT for 1 h. Again, 65 μl 1 M NEM followed by 1.004 g urea (8 M) was added. The samples were incubated for 3 h at RT and 800 rpm, followed by one methanol/chloroform (MeOH/CHCl_3_) precipitation. Pellets were redissolved in 400 μl 50 mM TEA pH 7.5, 150 mM NaCl, 5 mM EDTA, 2% (w/v) SDS (dissolving buffer), and transferred to 1.5 ml tubes. These lysates were then reduced with 21 μl of 500 mM TCEP for 30 min at RT and 800 rpm and alkylated once more with 22 μl NEM for 1 h at RT and 800 rpm. Samples were then precipitated with MeOH/CHCl_3_ four times. Pellets were redissolved in 200 μl dissolving buffer. For label-free pellets, all conditions and buffer components/concentrations were identical to the SILAC experiment with some adjustments. Cell pellets were lysed in 60 μl lysis buffer. 1040 μg of lysate (1040 μl) starting material was reduced, alkylated, and precipitated as described earlier. After the final precipitation, samples were divided into 2 x 200 μl (515 μg each).

#### Thioester Hydrolysis and Biotinylation

200 μl of 3.3 mM HPDP-biotin (in DMF/Milli-Q (MQ) = 2:1, Cayman chemical, 16459-50) was added to the samples and incubated for 5 min at RT and 800 rpm. All samples were incubated with 62 μl of 1 M hydroxylamine pH 7.5. Samples were incubated for 2 h at RT and 800 rpm and precipitated with MeOH/CHCl_3_ thrice. For label-free samples, the same amount of 3.3 mM HPDP-biotin was added, but this was followed by incubation with 62 μl of 1 M hydroxylamine (HA), pH 7.5 or 62 μl of MQ. Samples were incubated for 2 h at RT and 800 rpm and precipitated with MeOH/CHCl_3_ thrice. On the last methanol wash, samples were sonicated on ice at 70 to 80 Hz, 0.5 s on/off for 7 s to create a cloudy suspension. Samples were centrifuged for 2 min at the highest speed to pellet the protein.

#### Digestion

Protein pellets were resuspended in 150 μl (1.6 mg/sample) of 50 mM HEPES pH 8, 1% (w/v) sodium deoxycholate (SDC). Samples were digested with 1:100 (w/w) LysC (Wako chemicals, 121-02541) at 37 °C and 750 rpm for 1 h and 1:100 (w/w) trypsin (Sigma, 1426) at 37 °C and 750 rpm for 17 h. The digestion was quenched with 1 mM of phenylmethyl sulfonyl fluoride (PMSF, Sigma, P7626) dissolved in ethanol for 30 min at RT and 750 rpm. Label-free samples were digested similarly.

#### Biotin Peptide Enrichment and 2-Chloroacetamide Alkylation

Samples were diluted to 0.15% SDC (final volume = 1 ml) with 50 mM TEA pH 7.5, 150 mM NaCl, 5 mM EDTA. Samples were then added to 33 μl of NeutraVidin beads (pre-washed with 50 mM TEA pH 7.5, 150 mM NaCl, 5 mM EDTA, 0.1% SDS) and incubated for 2 h at RT with end-to-end rotation. Beads were washed 3x with 500 μl 50 mM TEA pH 7.5, 150 mM NaCl, 5 mM EDTA, 0.1% SDS and washed 2x with 500 μl 50 mM TEA pH 7.5, 150 mM NaCl, 5 mM EDTA. Lastly, they were washed 2x with 500 μl 50 mM ammonium bicarbonate (AMBIC) pH 8, 150 mM NaCl, 5 mM EDTA. Then, 150 μl of 50 mM TCEP, 50 mM AMBIC, pH 8, 5 mM EDTA prepared with HPLC-grade water was added to the beads and incubated for 30 min at RT with end-to-end rotation to elute peptides. Eluates were transferred to 1.5 ml low-binding tubes and centrifuged for 2 min at 2500 *g*. Supernatants were isolated and newly freed thiols were alkylated with 50 mM 2-chloroacetamide (CAA) in 50 mM AMBIC for 2.5 h at RT and 800 rpm in the dark. They were then dried by lyophilization and stored at −20 °C until LC-MS/MS analysis. Label-free biotinylated peptides were enriched, eluted, and alkylated similarly, except biotinylated peptides were initially added to 17.2 μl of pre-washed NeutrAvidin beads instead of 33 μl.

### Total Proteome MS Sample Preparation

#### SILAC Differentiation

20 μg (0.5 mg/ml) of combined heavy/light total proteome samples were reduced with 20 mM DTT in 50 mM AMBIC pH 8 for 40 min at RT and 800 rpm. Samples were then alkylated with 40 mM iodoacetamide (IAA) in 50 mM AMBIC pH 8 for 45 min at RT and 800 rpm in the dark. S-trap protocol was done according to the manufacturer’s instructions as followed. 2x SDS protein solubilization buffer (100 mM TEAB pH 7.5, 10% SDS) was added at a 1:1 (v/v) ratio. Samples were acidified with 12% phosphoric acid at a 1:10 (v/v) ratio, followed by the addition of six sample volumes of S-trap binding buffer (100 mM TEAB, pH 7.1 in 90% MeOH). Samples were loaded onto micro-S-trap columns (Protifi) and washed 5x with 150 μl S-trap binding buffer. 20 μl of digestion buffer (1:20 (w/w) trypsin (Sigma) in 50 mM AMBIC pH 8) was added to the column and incubated at 47 °C for 1 h. Samples were then eluted sequentially with (1) 40 μl 50 mM AMBIC pH 8, 40 μl 0.2% formic acid (FA) and (2) 35 μl 0.2% FA in 50% acetonitrile. Peptide samples were dried by lyophilization and stored at 20 °C until LC-MS/MS analysis.

#### 2-BP and PalmB Total Proteome

20 μg (0.5 mg/ml, 2-BP; 1 mg/ml, PalmB) of each proteome sample was reduced with 20 mM DTT in 50 mM HEPES pH 7.4 for 40 min at RT and 800 rpm. Samples were then alkylated with 40 mM IAA in 50 mM HEPES pH 7.4 for 45 min at RT and 800 rpm in the dark. Proteomes were digested using an SP3 protocol. Briefly, SP3 beads (hydrophilic:hydrophobic bead ratio 1:1) were washed with MQ thrice. Beads were then reconstituted in MQ for a final concentration of 50 μg/μl 4 μl of bead mastermix was added to each sample (beads-to-protein ratio 10:1). 168 μl ethanol was added to achieve a final concentration of 75% ethanol. Samples were incubated at RT and 1000 rpm for 20 min. Tubes were placed on a magnetic rack to remove supernatants. Beads were washed twice with 200 μl 80% ethanol and once with 200 μl 100% acetonitrile. Beads were resuspended in 100 μl 100 mM AMBIC pH 8 and sonicated in a water bath for 30 s 0.8 μg LysC and 0.8 μg trypsin was added and incubated for 18 h at 37 °C and 800 rpm. Samples were acidified with TFA, collected, and dried by Speedvac.

#### 2-BP and PalmB Secretome

Lyophilized secretomes were resuspended in 750 μl MQ. 2 × 750 μl was combined for each replicate (3 replicates total for each condition, each of 1.5 ml) and transferred to a new 2 ml tube. Samples were flash-frozen and lyophilized once more and resuspended in 300 μl resuspension buffer 1 (20 mM HEPES pH 7.4, 110 mM potassium acetate, 2 mM MgCl_2_, 1 μM ZnCl_2_, 1 μM CaCl_2_, 1% Triton X-100, 0.1% Tween-20, 0.5% sodium deoxycholate, 1x cOmplete mini EDTA-free protease inhibitor cocktail; 2-BP) or Resuspension buffer 2 (20 mM HEPES pH 7.4, 110 mM potassium acetate, 2 mM MgCl_2_, 1 μM ZnCl_2_, 1 μM CaCl_2_, 1% Triton X-100, 0.1% Tween-20, 5% SDS, 1x cOmplete mini EDTA-free protease inhibitor cocktail; PalmB). Resuspensions were sonicated in a water bath for 1 min. Secretomes were digested with an SP3 protocol, like the total proteome samples, with minor adjustments. Post-incubation, beads were washed 6 times with 200 μl 100% acetonitrile, instead of once. Beads were then resuspended in 500 μl 100 mM AMBIC pH 8, instead of 100 μl. Proteins were digested overnight at 37 °C and 1000 rpm with 0.04 μg LysC and 0.04 μg trypsin.

### Mass Spectrometry (MS) Data Acquisition

All dried peptide samples were dissolved in 20 μl 2% (v/v) FA for LC-MS/MS analysis.

#### SILAC ssABE, Label-free ssABE and Total Proteome Samples

Samples were analyzed on a nanospray UHPLC system Ultimate 3000 (ThermoFisher Scientific) coupled to an Orbitrap Exploris 480 mass spectrometer (ThermoFisher Scientific) in data-dependent acquisition mode. Peptides were initially loaded onto a PepMap Neo 5 μM C_18_ trap column (5 mm × 0.3 mm, ThermoFisher Scientific) with solvent A (0.1% formic acid) and separated on a homemade analytical column (SILAC/Label-free samples: ReproSil-Pur 120 C_18_-AQ, 2.4 μM, 75 μm × 50 cm; Total proteome samples: ReproSil-Pur 120 C_18_-AQ, 1.9 μm, 75 μm × 50 cm, in-house packed column with integrated emitter) at a flow rate of 0.3 μl/min. A gradient of 60 min was used for SILAC/label-free samples: 9% solvent B (0.1% FA in 80% acetonitrile) for 1 min, 9 to 13% for 1 min, 13 to 44% in 37 min, 44 to 55% in 5 min, 55 to 99% in 1 min, 99% for 5 min, and 9% for 10 min. A gradient of 180 min was used for total proteome samples: 9% solvent B (0.1% FA in 80% acetonitrile) for 1 min, 9 to 13% for 1 min, 13 to 44% in 155 min, 44 to 55% in 5 min, 55 to 99% in 5 min, 99% for 3 min, and 9% for 10 min. MS1 scans were performed at a resolution of 60,000 between 375 and 1600 m/z after reaching the normalized AGC target with automatic injection time every second. Top intense precursors were fragmented with normalized collision energy of 28% and 10 s dynamic exclusion time (SILAC/label-free peptides) or 24 s dynamic exclusion time (total proteome peptides). HCD fragmentation was performed on precursors at a resolution of 15,000 (SILAC/label-free peptides) or 30,000 (total proteome samples).

#### 2-BP and PalmB Inhibition Total Proteome

Samples were analyzed on a nanospray UHPLC system Ultimate 3000 (ThermoFisher Scientific) coupled to an Orbitrap Exploris 480 mass spectrometer (ThermoFisher Scientific) in data-independent acquisition (DIA) mode. Peptides were initially loaded onto a PepMap Neo 5 μM C_18_ trap column (5 mm × 0.3 mm, ThermoFisher Scientific) with solvent A and separated on a homemade analytical column (ReproSil-Pur 120 C_18_-AQ, 1.9 μm, 75 μm × 50 cm, in-house packed column with integrated emitter) at a flow rate of 0.3 μl/min. A gradient of 90 min was used: 4% solvent B (0.1% FA in 80% acetonitrile) for 1 min, 4 to 11% in 3 min, 11 to 30% in 58 min, 30 to 44% in 5 min, 44 to 55% in 5 min, 55 to 99% in 3 min, 99% for 5 min and 4% for 10 min. MS1 scans were performed at a resolution of 60,000 between 375 and 1600 m/z after reaching the normalized AGC target with automatic injection time every second. Fragment spectra were recorded in profile mode over 30 consecutive windows of 20 m/z (1 m/z overlap) covering the precursor mass range of 400 to 1000 m/z and using a resolution of 30,000 using HCD fragmentation with normalized collision energy of 28%.

#### 2-BP and PalmB Inhibition Secretome

Samples were analysed on a nanospray UHPLC system Ultimate 3000 (ThermoFisher Scientific). Peptides were initially loaded onto a PepMap Neo 5 μM C_18_ trap column (5 mm × 0.3 mm, ThermoFisher Scientific) with solvent A and separated on a homemade analytical column (ReproSil-Pur 120 C_18_-AQ, 1.9 μm, 75 μm × 50 cm, in-house packed column) heated to 50 °C by an external column oven (Sonation). The peptides were separated in a 60 min gradient (1% B for 1 min, 1–3% in 0.1 min, 3–35.2% in 39.9 min, 35.2–80% in 3 min, 80% for 5 min, 1% for 10 min) at a flow rate of 0.3 μl/min. The LC was coupled to a trapped ion mobility quadrupole time-of-flight mass spectrometer timsTOF HT (Bruker Daltonics) via a nanoelectrospray ion source CaptiveSpray (Bruker Daltonics). Data acquisition on the timsTOF HT was performed using timsControl 6.0.0.13692 and Compass HyStar 6.3.208.1 (Bruker Daltonics) using the standard proteomics application method dia-PASEF – long gradient. This method utilized a capillary voltage of 1600 V and a nebulizer dry gas flow rate of 3.0 L/min at 180 °C. Data were acquired in the range of 100 to 1700 *m*/*z*. Fragment spectra were recorded over a precursor mass range of 400 to 1201 m/z over 16 MS/MS ramps and 32 25 m/z MS/MS windows (100 ms accumulation/ramp), with a total cycle time of ∼1.80 s. The ion mobility range was 0.60 to 1.60 1/K_0_. The collision-induced dissociation energies were linearly ramped as a function of ion mobility, ranging from 20.00 (1/K_0_ = 0.6 V cm^–2^) to 59 eV (1/K_0_ = 1.60 V cm^–2^). The diaPASEF isolation window table is listed in Supplemental Table S3-6.

### Database Search

All SILAC data, as well as label-free differentiation data was processed with MaxQuant version 2.4.14.0 and 2.5.0.0 and the MS/MS spectra were searched using the Andromeda search engine. Total proteome and secretome data from the ZDHHC and APT inhibition experiments were processed with DIA-NN (version 1.9 or 1.9.2). ssABE data from the APT inhibition experiment was additionally processed by Fragpipe (version 22.0). All raw files (originating from different experiments and processed with different search engines) were searched against the same human UniProt database (version May 2024, UP000005640, SwissProt-reviewed proteins, excluding protein isoforms, 20,644 entries).

#### Label-Free Differentiation

Raw files were searched by parameter groups (+HA replicates and -HA replicates). Methionine oxidation, protein N-term acetylation and cysteine carbamidomethylation were set as variable modifications. Variable modification NEM on cysteines (+125.047678 Da) was also included. All files were analyzed by using the built-in label-free quantification (LFQ) algorithm separated based on parameter groups. Enzyme specificity was set to Trypsin and LysC with an allowance of two missed cleavages. Match-between-runs was enabled. Precursor mass tolerances were set to 20 ppm in the first search, and 4.5 ppm in the main search. Fragment ion mass tolerance was set to 20 ppm. The false discovery rate was controlled with a target-decoy approach at less than 1% for PSMs and less than 1% for protein identifications.

#### SILAC Differentiation

SILAC ssABE and total proteome raw files were processed and searched separately. For each search, labeling multiplicity was set at 2, with Arg10 and Lys8 selected as heavy labels. Methionine oxidation, protein N-terminal acetylation and cysteine carbamidomethylation were set as variable modifications. NEM modification on cysteines was included for the ssABE files. Enzyme specificity was set to LysC and trypsin for ssABE, and solely trypsin for the total proteome. In both cases, the maximum number of missed cleavages allowed was 2. Match-between-runs and requantify were both enabled. Minimum peptide length was set to six for ssABE samples. Precursor tolerances, fragment ion tolerances, PSM FDR thresholds and protein FDR thresholds were the same as the label-free differentiation experiment

#### 2-BP or PalmB Inhibition Total Proteomes and Secretomes

DIA-NN’s software was used in library-free mode. Trypsin/P was selected as the protease, and two missed cleavages were tolerated. N-term methionine excision was allowed. Cysteine carbamidomethylation was allowed as a fixed modification and methionine oxidation and N-term acetylation were allowed as variable modifications. A maximum of three variable modifications per peptide was allowed. The peptide length range was 7 to 50 amino acids. Precursor charge range was set between 2 and 6. Match-between-runs, no shared spectra and the heuristic inference options were enabled. The protein inference was set to Genes, and the IDs, RT, and IM profiling were used for the library generation. The false discovery rate was at the default 1%. For secretome samples, the search was done like the total proteome search, with the only adjustment that the mass accuracy was set to 20 ppm and the MS1 accuracy to 10 ppm.

#### PalmB Inhibition S-Acyl-Proteome

Raw files were searched with MaxQuant by parameter group (±PalmB, ±HA). All other parameters were identical to those described in the “label-free differentiation” search. For searches to check for endogenous protein N-term myristoylation, an additional variable modification was included in the search: glycine myristoylation (+210.19836 Da).

To verify endogenous myristoylation sites detected by MaxQuant, Exploris raw files were additionally searched against the same human Uniprot database as the other searches, using Fragpipe v22.0 with MSFragger 4.1, IonQuant 1.10.27 search engines with default settings. Decoys and common contaminants were added. MSFragger search specifications were as follows: cleavage specificity was set to strict trypsin with a maximum of two missed cleavages and protein N-terminal methionine cleavage. A peptide length between 7 and 50 was allowed. Oxidation of methionine, acetylation of the protein N-terminus, carbamidomethylation of cysteines (57.02146, C), NEM modification of cysteines (125.047676, C) and myristoylation of the protein N-terminus (210.19836, [ˆ) were set as variable modifications. The following mass offsets were defined: 0.00000(aa=); 15.99492(aa = M); 57.02146(aa = C); 210.19836(aa = G); 125.04767(aa = C). The maximum number of variable modifications on a peptide was set to 4. Precursor and fragment mass tolerance were both set to 20 ppm. PeptideProphet was used for PSM validation with defaults for a closed search. ProteinProphet was used for protein interference. The false discovery rate for PSMs, peptides and proteins was set to 1% using a target-decoy approach. MaxLFQ was calculated. Match-between-runs (MBR ion FDR, 1%) was allowed, and intensity was normalized across runs. Min site localization probability was set at 0.75.

### Data Processing and Statistical Analysis

All data was processed using a combination of in-house R scripts (v4.4.0) with RStudio (v2024.04.2 + 764), Perseus (v1.6.14.0) and Excel (Microsoft Office 365, v16.0.14326.20852).

#### Benchmark List of HA-Sensitive Long-Chain S-Acylation Sites in THP-1 Macrophages

The evidence.txt file from MaxQuant was imported into RStudio. Reverse hits, potential contaminants, and PSMs lacking MS/MS scan numbers or detected intensities were excluded. PSMs containing cysteines were selected, and the modification columns (“Carbamidomethyl (C)” and “NEM”) along with the “Intensity” column were used to assess carbamidomethylation efficiency. The peptides.txt file was then imported, filtered to remove reverse hits and potential contaminants, and peptides without detected intensities in any replicate were discarded. The “C.count” column was used to determine cysteine-peptide enrichment efficiency per replicate. For site analysis, the Carbamidomethyl (C)Sites.txt file was read into RStudio. False positives (e.g., E2 ubiquitin-conjugating enzymes, E1 ubiquitin-activating enzymes), reverse hits, and potential contaminants were removed. Samples were grouped by condition (M(LPS + IFNɣ) or M0) and by the presence or absence of HA treatment. Raw intensities were log_2_-transformed, and replicates were filtered based on valid values (present in 2/2 or 2/3 +HA replicates for M(LPS + IFNɣ) and M0 conditions, respectively). A minimum of two + HA replicates per condition was required to have a carbamidomethyl localization probability of ≥0.75. Confident sites (i.e., sites with no valid values in the -HA replicates but ≥2 valid values in the +HA replicates) were temporarily excluded. Two lists of sites (M(LPS + IFNɣ) and M0) were generated and imported into Perseus. Replicates were grouped by ± HA treatment and imputed by replacing missing values with those from a normal distribution. To identify significant differences between ± HA conditions, an unpaired Student’s *t-*test was performed (FDR 0.05, S0 0.1, fold change ≥1). Sites significantly upregulated in M(LPS + IFNɣ) and/or M0 conditions were combined with confident sites. These sites were considered HA-sensitive. Finally, M(LPS + IFNɣ) and M0 HA-sensitive sites were merged. The final list was manually curated to remove false positives due to imputation. For M(LPS + IFNɣ), if a single -HA replicate had a non-imputed value and the other one was imputed, the site was considered “not detected” unless the average raw intensity of the +HA replicates was at least twice the raw intensity of the non-imputed -HA replicate. For M0, sites with any imputed values in the -HA condition were considered “not detected” unless the average raw intensity in the +HA replicates was at least twice that of the non-imputed -HA values. Sites that remained were used to benchmark the SILAC ssABE experiment. MS/MS spectra were visualized using Annotator.

#### SILAC Proteome

The proteinGroups.txt file from MaxQuant was imported into RStudio. Reverse hits, proteins identified only by site, potential contaminants, and proteins identified by ≤ 1 unique peptide were excluded. Heavy and light intensity columns were grouped by condition (M(LPS + IFNɣ): Intensity.H.1H, Intensity.H.2H, Intensity.L.3L, Intensity.L.4L; M0: Intensity.L.1H, Intensity.L.2H, Intensity.H.3L, Intensity.H.4L) and log_2_-transformed. Proteins were filtered to include only those with valid values in at least three out of four replicates under M(LPS + IFNɣ) and/or M0 conditions. Confident protein IDs, defined as those with valid values in three out of four replicates for one condition but one or fewer valid values in the other, were temporarily excluded from analysis. Missing values were imputed from a normal distribution in Perseus after grouping replicates by M(LPS + IFNɣ) or M0 polarization. Significant differences between M(LPS + IFNɣ) and M0 conditions were identified using a paired two-sample *t*-test (FDR 0.05, S0 0.1, fold change ≥1.25). Proteins that were significantly upregulated, including confident protein IDs, were then subjected to Gene Ontology Analysis using PantherDB (www.pantherdb.org, v19.0).

#### SILAC ssABE

The “evidence.txt,” “peptides.txt,” and “Carbamidomethyl (C)Sites.txt” files were loaded into RStudio. Carbamidomethylation efficiency and cysteine-peptide enrichment were assessed using the “evidence.txt” and “peptides.txt” files, following a similar approach to label-free ssABE. Site analysis was conducted using the “Carbamidomethyl (C)Sites.txt” file. Reverse hits, potential contaminants, and false positive sites were excluded, as in the label-free ssABE procedure. Heavy- and light-intensity columns were grouped by condition (M(LPS + IFNɣ): Intensity.H.1H, Intensity.H.2H, Intensity.L.3L, Intensity.L.4L; M0: Intensity.L.1H, Intensity.L.2H, Intensity.H.3L, Intensity.H.4L) and log_2_-transformed. Ratios were manually calculated. Sites were filtered based on a carbamidomethylation localization probability of ≥0.75 in at least three out of four replicates. Confident sites, defined as those with valid values in at least three out of four replicates in one condition but one or fewer in the other, were retained since they could serve as on/off switches. Sites quantified in fewer than three out of four replicates were excluded. The resulting list of confident or consistently quantified sites was filtered for HA sensitivity using the benchmark from the label-free ssABE experiment, removing non-sensitive sites. Missing values were imputed from a normal distribution, and quantified sites were subjected to paired two-sample *t*-tests (FDR 0.05, S0 0.1, −0.5 ≥ fold change ≥0.5). Confident and significantly upregulated sites in M(LPS + IFNɣ) or M0 conditions were compared to the SwissPalm database (www.swisspalm.org) (4). The parent proteins of these sites were analyzed for transmembrane protein type using the Uniprot database (www.uniprot.org, release 2024_04) and the “Subcellular location [CC]” column. For *S*-acyl-peptidoform abundance analysis of multi-site peptides, PSMs corresponding to detected HA-sensitive sites with a localization probability ≥0.75 in three out of four replicates were extracted from the “evidence.txt” file. PSMs for multi-site peptides were isolated, and those without detected intensities in both heavy and light channels were discarded. Methionine-oxidized or acetylated protein N-term versions of multisite peptides were grouped with their non-oxidized/non-acetylated counterparts. Missed cleavages were addressed by in-silico digestion. The resulting digested peptide sequences were grouped with existing site-peptide sequences or designated as novel unique peptide sequences if no match was found. For each multi-site peptide, PSMs corresponding to each detected *S*-acyl-peptidoform were grouped. The summed PSM intensity for each *S*-acyl-peptidoform (based on “Intensity” column), as well as the intensity sums for M(LPS + IFNɣ) and M0 conditions individually (based on “Intensity.H” and “Intensity.L” columns) were divided by the total sum PSM intensity across all *S*-acyl-peptidoforms and conditions respectively, yielding fractions representing relative *S*-acyl-peptidoform abundance under each condition and in aggregate. Only *S*-acyl-peptidoforms that made up ≥ 5% of the total average intensity of a multi-site peptide in either the M(LPS + IFNɣ) or M1 condition were included in the *S*-acyl-peptidoforms versus number of cysteines in peptide sequence analysis.

**Fig. 1:**
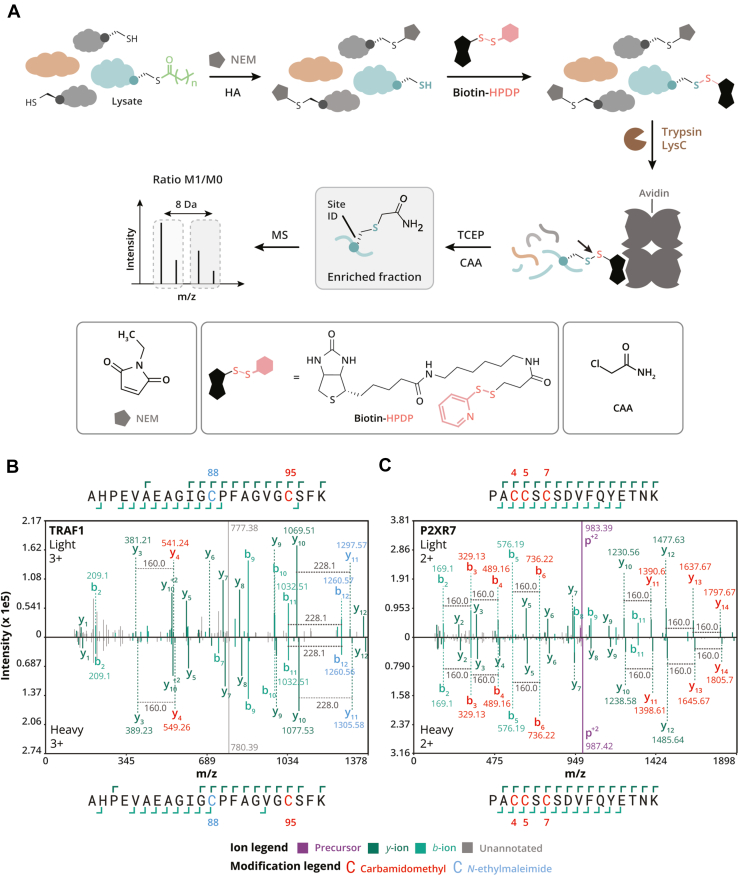
**SILAC ssABE workflow.***A*, ssABE was performed by digesting protein mixtures prior to biotin enrichment. Enriched peptides were alkylated with CAA after elution. The resulting peptide mixtures were lyophilized and analyzed by nano-LC-MS/MS. *B–C*, MS2 spectra of (*B*) TNF receptor-associated factor 1 (TRAF1) and (*C*) Purinoceptor 7 (P2RX7). Labels of peaks corresponding to putative long-chain *S*-acylation sites are colored *red*. Labels of peaks corresponding to cysteines modified with NEM are colored *light-blue*. These colors are also used to color the respective cysteine residues in the peptide annotations. If no charge is indicated on the individual ions then the charge is +1.

**Fig. 2:**
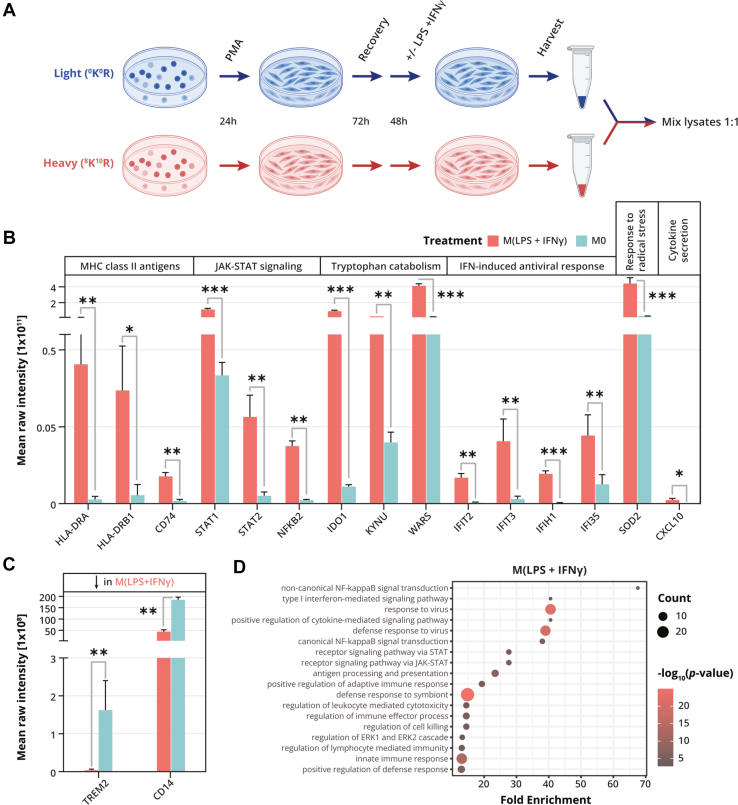
**SILAC total proteome analysis of pro-inflammatory M(LPS + IFNɣ) and naïve M0 THP-1 macrophages.***A*, THP-1 cells were labelled with heavy (^8^K^10^R) or light (^0^K^0^R) medium for approximately 2 weeks. Cells were then differentiated with PMA and recovered in PMA-free medium. Inflammation was induced in heavy and light cells with LPS and IFNɣ. As negative controls, light and heavy cells were treated with PBS. Cells were lysed and (LPS + IFNɣ)-heavy was combined with PBS-light and (LPS + IFNɣ)-light was combined with PBS-heavy in a 1:1 ratio. The experiment was performed in *n* = 4 biological replicates. *B*, raw intensity of pro-inflammatory proteins in M(LPS + IFNɣ) and M0 macrophages. The adjusted *p*-values were calculated using ratio paired *t*-tests employing Holm-Šidák’s multiple comparisons test to determine significant differences between M(LPS + IFN_Ɣ_) and M0 macrophages (ns = not significant, *∗p* ≤ 0.0332, *∗∗p* ≤ 0.0021, *∗∗∗p* ≤ 0.0002, *∗∗∗∗p* ≤ 0.0001). Data were mean ± SD. *C*, raw intensity of proteins TREM2 and CD14 that are downregulated during inflammation. Significance was determined as described before, and data were mean ± SD. *D*, GO biological process analysis for proteins upregulated in M(LPS + IFNɣ) macrophages (FDR <0.05).

**Fig. 3:**
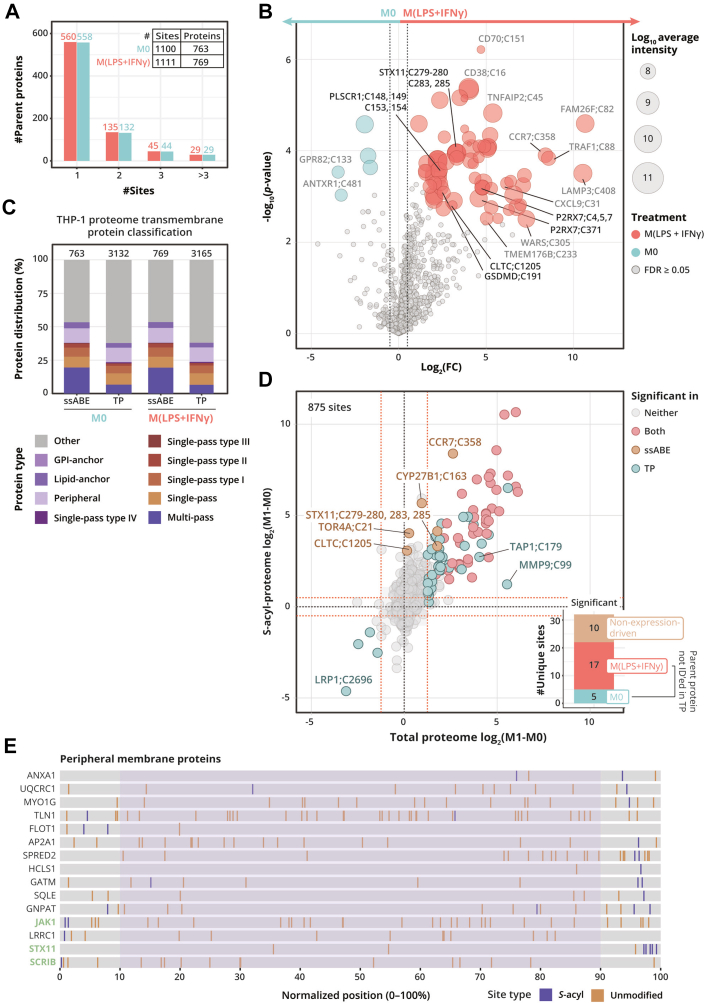
**SILAC ssABE analysis of pro-inflammatory M(LPS + IFNɣ) and naive M0 THP-1 macrophages.***A*, Bar chart of HA-sensitive *S*-acyl parent proteins categorized by number of putative *S*-acyl sites detected. *B*, 3D volcano plot showing the differential abundance of HA-sensitive and quantified *S*-acyl sites in M(LPS + IFNƔ) and M0 macrophages. Bubble size of significant sites is determined by the average log_10_ raw intensity of the site in the condition it is significant in. Sites not significantly up- or downregulated are depicted in *grey* and have the same bubble size regardless of their average log_10_ raw intensity. Significance was determined by two-sample paired *t-*test (FDR 0.05, S0 0.1 and *n* = 4 biological replicates). Vertical dashed lines are at log_2_ (FC) = 0.5 and = −0.5. *C*, stacked bar-chart showing the number of HA-sensitive *S*-acyl parent proteins (ssABE) and the experimentally determined THP-1 proteome, classified by transmembrane protein type. Transmembrane protein classification was done using the Uniprot database. *D*, correlation plot showing the SILAC log_2_ fold changes of sites and parent proteins identified by SILAC ssABE and total proteome analysis. *S*-acyl-proteome log_2_ fold changes are reported for specific site peptides. Total proteome log_2_ fold changes are reported for the parent protein of a specific site peptide. Sites with significant changes in abundance in SILAC ssABE are *teal*. Sites with significant changes in parent protein abundance in the proteome are *light-blue*. Sites with significant changes in abundance in SILAC ssABE and parent protein abundance are *light-red. Red dashed lines* indicate log_2_ (FC) significance thresholds; For total proteome |log_2_ (FC)| = 1.25, for ssABE |log_2_ (FC)| = 0.5. Sites with no significant changes in abundance in either SILAC ssABE or total proteome analysis are *grey*. A total of 875 sites with matching protein abundances are shown. Inset bar chart shows the sites (i) whose parent protein was not detected in the total proteome, but were significant in either M0 or M(LPS + IFNƔ) macrophages, (ii) were significant after correction for protein abundance (“non-expression driven”). *E*, some peripheral membrane parent proteins that were identified. Protein length was normalized to 100%, and *S*-acyl sites (*purple*) and unmodified cysteines (*teal*) were mapped relative to protein length. Shown are the top 15 proteins whose detected long-chain *S*-acylation sites reside closest to the protein N- or C-terminus.

**Fig. 4:**
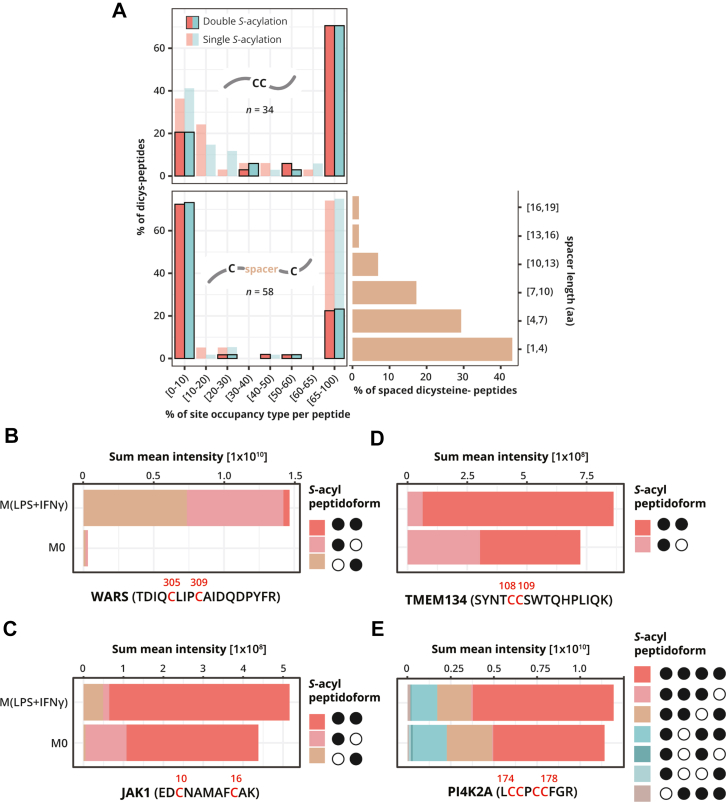
**Long-chain *S*-acylation peptidoforms.***A*, overview of occupancy distributions for di-cys-containing peptides. Coral corresponds to M(LPS + IFN_ɣ_), cyan to M0. *Left panels*: relationship between the percentage of di-cys-containing peptides (*y*-axis) and the percentage that can be attributed to their double and single *S*-acylated modiforms (*x*-axis). *Bottom right panel*: overview of spacer length (*y*-axis) for spaced-di-cys-peptides (*x*-axis). *B–E*, peptides with multiple sites, the detected *S*-acyl-peptidoforms and their summed extracted ion currents (XICs). PSMs detected for a multi-site peptide were grouped by their unique combination of modifications (*S-*acyl and NEM). Each unique combination is considered an *S*-acyl peptidoform. Bar charts for all 118 multisite peptides are included in Appendix B.

#### 2-BP or PalmB Inhibition Proteome and Secretome

The report.tsv file from DIA-NN was read into Rstudio. The data was filtered based on the FDR criteria 1% (Q Value, Lib Q Value and Lib PG Q Value). Only genes with at least two unique peptides were retained and the data was pivoted to a protein grouped table. PG MaxLFQ intensities were log_2_-transformed. Proteins were removed and had no MaxLFQ intensity in any of the replicates. Confident proteins (proteins that were detected in ≥2/3 replicates in one condition and 0/3 replicates in the other, and vice versa) were temporarily removed from the table. Data were filtered to allow proteins with 2/3 valid values in any of the conditions. The data were imputed with the missForest R package. The protein table was imported into Perseus, and an unpaired two-sample *t*-test (FDR 0.05, S0 0.1, fold change >0.5) was performed to determine significant differences between 2-BP and DMSO (vehicle) treated samples. The secretome DIA-NN output was treated in the same way with slight alterations. Proteins were considered secreted if they were featured in the list of secreted proteins from the Human Protein Atlas (downloaded on March 12, 2025) or a list of proteins with their Subcellular Location “Secreted Extracellular Space” extracted from Uniprot (version February 2025). If SignalP 6.0 predicted a signal peptide, proteins were also retained. Proteins were considered downregulated in 2-BP compared to vehicle if they were either absent in the 2-BP-treated secretomes or showed an FDR <0.05, fold change ≤ −1.76, and had 2/3 values not imputed in the vehicle condition.

#### PalmB Inhibition S-Acyl-Proteome

The “Carbamidomethyl (C)Sites.txt” file was loaded into RStudio. Reverse hits, potential contaminants, and false positive sites were excluded. Intensity columns were grouped by condition and log_2_-transformed. Sites were retained which had valid values in two out of two technical replicates, for three out of three biological replicates in the HA treated conditions. Sites further had to have a localization probability ≥0.75 in two out of two technical replicates, for ≥2 out of three biological replicates in the HA-treated conditions. All replicates were then median normalized, and technical replicates were averaged. Confident sites were defined as those with valid values in all + HA replicates (technical and biological) and ≤1 valid value in all -HA replicates (technical and biological). Sites with valid values for both technical replicates in at least one biological replicate were retained. Data were imputed by replacing missing values with those from a normal distribution. Means were recalculated and significant differences between ± HA conditions were determined with an unpaired Student’s *t*-test (FDR 0.05, S0 0.1, fold change ≥0.5). Confident sites and sites found significant after *t*-testing were considered HA-sensitive. Long-chain *S*-acylation sites were then filtered based on the presence of HA-sensitivity in both DMSO (vehicle) and PalmB-treated samples. Resulting mean normalized log_2_ intensities were imported into Perseus, and significant differences between vehicle and PalmB were determined using an unpaired Student’s *t*-test (FDR 0.05, S0 0.1, fold change ≥0.5).

### Data Visualization

All processed data were visualized using RStudio (v.2024.12.0-0467), R (v.4.4.0), Graphpad Prism (v10.1.2324), Pymol (v3.03) and meta-chart.com. SILAC MS2 spectra were annotated and visualized with Annotator (v1.0.0) (50). Images were further processed in Adobe Illustrator 2024/2025/2026.

### Experimental Design and Statistical Rationale

The label-free differentiation experiment included *n* = 2 biological M(LPS + IFNγ) replicates (±HA) and *n* = 3 biological M0 replicates (±HA), generated across two independent experiments. These replicate numbers were sufficient to enable comparisons between ± HA samples within each condition; however, no quantitative comparisons were performed between biological states in this dataset. For SILAC ssABE and total proteome analyses, *n* = 4 biological replicates per condition were analyzed, incorporating isotopic label swapping and derived from two independent experiments. This design ensured reproducibility, enabled quantitative comparisons between conditions, and controlled for variability arising from isotopic labeling. The zDHHC and APT inhibition experiments (total proteome, secretome, and ssABE if applicable) were conducted with *n* = 3 biological replicates per condition. Each replicate consisted of pooled material from five T75 culture flasks. This level of replication was sufficient to assess reproducibility and to perform quantitative comparisons between conditions within each experiment.

## Results and Discussion

### Establishing a Robust and Sensitive ssABE Strategy to Detect Long-Chain *S*-Acylation

A previously reported site-specific acyl-biotin exchange (ssABE) approach relied on TCEP-mediated peptide elution followed by immediate sample clean-up and analysis via nano-LC-MS/MS (19, 27). We adapted this workflow by including an additional capping step after elution, with the rationale this may further stabilize newly liberated cysteines during downstream processing (Fig. 1*A*). In ABE-based protocols more broadly, incomplete alkylation of non-*S*-acylated cysteines prior to thioester hydrolysis can contribute to false-positive identifications. In earlier work, we showed that a double *N*-ethylmaleimide (NEM) alkylation step achieves near-complete capping of non-*S*-acylated cysteines in a protein-level ABE workflow (20). Based on this observation, we incorporated the same double alkylation strategy into the present ssABE protocol to reduce false-positive identifications.

We applied our ssABE approach to THP-1 macrophages under differential stimulation (M(LPS + IFNɣ): lipopolysaccharides (LPS) + interferon gamma (IFNɣ), M0: vehicle (PBS)) in a label-free experimental setup and compared hydroxylamine (HA)-treated and untreated replicates, as HA selectively cleaves thioester-linked modifications. Analysis of the eluted peptides revealed highly efficient cysteine-containing peptide enrichment and CAA capping after TCEP-mediated release, with 96% to 99% of the total peptide intensity in each replicate attributable to peptides containing at least one cysteine residue (Supplemental Fig. S2, *A*–*D*, Supplemental Table S1).

Next, we assessed site identification. We identified 3083 putative long-chain *S*-acylation sites, either exclusively detected in +HA samples or significantly enriched in +HA compared to -HA samples (log_2_ fold change ≥1, FDR <0.05 in either M0 or M(LPS + IFNɣ) condition or in both) (Supplemental Fig. S2*E*, Supplemental Table S1). These sites mapped to 1880 unique parent proteins. For most proteins, a single long-chain *S-*acylation site was detected (Figs. 1*B* and S2*F*). However, 36% harbored two or more long-chain *S*-acylation sites, highlighting the importance of site-specific resolution when detecting long-chain *S*-acylation (Figs. 1*C* and S2*F*).

To evaluate the biological plausibility of the identified long-chain *S*-acylation sites, we cross-referenced our dataset with entries in SwissPalm, a curated database of long-chain *S*-acylated proteins (4). While only 11% of the identified sites had been previously reported (Supplemental Fig. S3*A*), 70% of parent proteins had been identified in prior palmitoyl-proteomics studies (Supplemental Fig. S3*B*). This discrepancy likely reflects the limited availability of site-resolved data in SwissPalm. Consistently, only 4% of parent proteins lacked any supporting evidence from prior studies, predicted *S*-acylation, or the presence of corresponding orthologues (Supplemental Fig. S3*B*).

We next compared our dataset with published MS-based proteomics studies reporting long-chain *S*-acylation sites. As no site-resolved datasets are available for THP-1 macrophages, we focused on studies performed in HeLa cells and HK9/11 cells using complementary enrichment strategies. These strategies included iodoTMT labeling (Supplemental Fig. S3*C*) (27), nanographite fluoride-based solid-phase extraction (nGF-SPE; Supplemental Fig. S3*D*) (51), PASSILE (Supplemental Fig. S3*E*) (52), and lipid metabolic labeling (LML; Supplemental Fig. S3*F*) (53). Site-level overlap was limited, ranging from 3.7% (nGF-SPE) to 7.5% (PASSILE). Only C39 on SLC1A5 was consistently identified across all techniques (Supplemental Table S1-1). A small number of sites were shared with multiple methods (e.g. C492 on STAT1 with PASSILE and LML), while others were detected by one other approach only (e.g., C263 on zDHHC20 by nGF-SPE; C175 on CD81 by iodoTMT) (Supplemental Table S1-1). The limited overlap likely reflects both methodological differences in enrichment strategies and the distinct biological functions of THP-1 macrophages compared to HeLa or HK9/11 cells.

To further assess data robustness, we assessed common omics-level metrics across the 3083-site dataset. Coefficient of variation (CV) distributions were centered below 20% for + HA replicates of M(LPS + IFNγ) macrophages and ∼50% for M0 macrophages (Supplemental Fig. S4, *A*–*C*). PCA showed clear separation of +HA and −HA samples along PC1 for both M0 (76.55%) and M(LPS + IFNγ) (81.87%) conditions (Supplemental Fig. S4, *D* and *E*), Consistently, +HA replicates exhibited high Pearson correlations (>0.9 for M0; >0.96 for M(LPS + IFNγ); Supplemental Fig. S4, *F* and *G*). +HA samples contained fewer missing values than -HA samples (Supplemental Fig. S4, *H*–*K*). Lastly, missing values were predominantly imputed in the low-intensity range, minimizing the risk of bias in downstream analyses (Supplemental Fig. S5).

The combination of high-efficiency capping, robust cysteine-containing peptide enrichment and carbamidomethyl labeling after TCEP-mediated peptide release from the beads, and the ability to detect both single- and multisite peptides shows the effectiveness of this ssABE workflow.

### LPS + IFNɣ Treatment of THP-1 Macrophages Produces a Distinct Pro-Inflammatory Signature

We developed a site-specific, quantitative method to study the long-chain *S*-acylation landscape in inflammatory macrophages by integrating SILAC into our ssABE workflow. Sufficiently isotopically labeled THP-1 monocytes were matured into M(LPS + IFNɣ) pro-inflammatory macrophages with phorbol 12-myristate 13-acetate (PMA) followed by treatment with LPS and IFNɣ using a protocol described by Baxter *et al.* (Fig. 2*A*, Supplemental Fig. S1) (49). To ensure quantification accuracy, four biological replicates combined with SILAC label-swaps were included for the experiment.

First, we analyzed protein abundances. For both inflammatory and non-inflammatory conditions, approximately 83% of identified proteins were shared among all four replicates (Supplemental Fig. S6, *A* and *B*). Log_2_ intensity distributions were very similar (Supplemental Fig. S6*C*), and replicates showed strong correlations (Supplemental Fig. S6*D*). These metrics indicate high experimental reproducibility. Interestingly, nearly all detected proteins were present in both M0- and M(LPS + IFNɣ) macrophages, with only 44 proteins unique to the pro-inflammatory state (Supplemental Fig. S4*E*). Despite this extensive overlap in protein identifications, M(LPS + IFNɣ) and M0 macrophage populations were distinguishable, as shown by PCA analysis of the log_2_ intensities of proteins from both conditions (Supplemental Fig. S4*F*). We observed a significant upregulation of key pro-inflammatory markers and associated pathways essential for inflammation, in M(LPS + IFNɣ) macrophages compared to M0 macrophages. These pathways included the JAK-STAT signaling pathway (54, 55, 56, 57), HLA class II histocompatibility antigens (58), tryptophan catabolism (59, 60, 61, 62, 63), pro-inflammatory cytokine secretion (64, 65), and response to ROS stress (66, 67) (Figs. 2*B* and S7).

Additionally, interferon-induced proteins such as IFIT3, IFIT2, IFIT1, IFIH1 and IFI35 (all with log_2_ fold change >2) were significantly upregulated, indicating an anti-viral state (Figs. 2*B* and S7). In contrast, several proteins were downregulated, including well-known anti-inflammatory triggering receptor TREM2 (log_2_ fold change = −4.38, *q*-value = 0.029) and LPS co-receptor CD14 (log_2_ fold change = −2.098, *q*-value = 0.019) (Figs. 2*C* and S7) (68, 69).

Finally, a gene ontology (GO) analysis of proteins significantly upregulated in M(LPS + IFNɣ) macrophages revealed enrichment in biological processes such as defense response to viruses, receptor signaling via the JAK-STAT pathway, antigen processing and presentation and positive regulation of adaptive immune response (FDR <0.05) (Figs. 2*D* and S8).

These trends align well with established pro-inflammatory macrophage functions and are consistent with recent proteomic studies of LPS-stimulated THP-1 cells by Mulvey *et al.* (2021) and Ctortecka *et al.* (2024) (70, 71). Therefore, with the pro-inflammatory state confirmed by total proteome analysis, we were well-positioned to profile the *S*-acyl-proteome for long-chain *S*-acylation modifications.

### Site-Specific *S*-Acyl-Proteome Analysis Reveals Upregulation of Long-Chain *S*-Acylation in Pro-Inflammatory Macrophages

Using our ssABE strategy in both naïve and pro-inflammatory macrophages, we identified 1111 *S*-acylated cysteines across 769 unique parent proteins. Of these, 11 sites were exclusively detected in pro-inflammatory macrophages (Figs. 3*A* and S9*D*, Supplemental Table S2-1). Label-free benchmark analysis in both inflammatory and naïve states was used to control for site-specific HA-sensitivity (Supplemental Table S1-1). Although there was significant overlap in site identifications between conditions, Pearson correlation analysis revealed that *S*-acylation abundance between the two macrophage populations was still distinct (Supplemental Fig. S9, *A*–*C*), and we observed an increase in long-chain *S*-acylation in M(LPS + IFNɣ) macrophages compared to M0 macrophages (Fig. 3*B*).

Notably, ∼73% of parent proteins had just a single detectable site (560/769 for M(LPS + IFNɣ) and 558/763 for M0). Furthermore, 38% of parent proteins contained at least one transmembrane domain, ∼11% were peripheral membrane proteins and ∼4.5% were lipid-anchored membrane proteins (Fig. 3*C*). In comparison, only ∼24% of all proteins detected in the total THP-1 proteome contained at least one transmembrane domain (Fig. 3*C*). Closer inspection revealed that this enrichment was primarily driven by multi-pass transmembrane proteins (Fig. 3*C*). These data indicate an enrichment of long-chain *S*-acylated proteins among (trans)membrane proteins in THP-1 macrophages, consistent with previous observations in other cell types (19, 25, 72, 73, 74).

Rank abundance analysis of long-chain *S*-acylation site intensities in M0 and M(LPS + IFNɣ) macrophages revealed that phospholipid scramblase 1 (PLSCR1; C148-149) was among the most highly abundant sites in both naïve and M(LPS + IFNɣ) macrophages (Figs. 3*B* and S9*D*, Appendix A). PLSCR1 is a well-established *S*-acylated protein that localizes to lipid rafts which is mediated by a cysteine-rich motif (^184^CCCPCC^189^) (75). Upon interferon treatment, deacylation of this motif promotes the nuclear translocation of PLSCR1 in Hey1B and MEF cells, where it activates other antiviral Interferon Stimulated Genes (ISGs) (75, 76, 77). Additionally, PLSCR1 plays a direct antiviral role in Huh7.5 and A549 cells by blocking cell membrane fusion mediated by the SARS-CoV-2 spike protein (78).

We did not detect long-chain *S*-acylation at the classical cysteine-rich region (C184-189), which might be due to the technical limitations associated with peptide identification after tryptic digestion. However, long-chain *S*-acylation of PLSCR1 was found to be significantly upregulated in M(LPS + IFNɣ) compared to M0 macrophages at several other sites, including C148, C149, C153, C154, C234, C237, C239, C240, and C254 (Appendix A). The high abundance of long-chain *S*-acylated PLSCR1 and its upregulation in pro-inflammatory macrophages may indicate an important role for long-chain *S*-acylation of PLSCR1 during macrophage-mediated inflammation

Several well-characterized long-chain *S*-acylation sites with critical roles in immune cell function were detected and upregulated upon the inflammatory stimulus. We identified seven such sites on purinoceptor 7 (P2RX7): C4-5, C7, C371, C373-374, and C377 (Figs. 1*C*, 3B and S10, Appendix A). These sites are located peripherally to the cell membrane, where long-chain *S*-acylation prevents desensitization of P2RX7 to ATP during inflammation. This prolongs inflammasome activation, enhancing IL-18 and IL-1β secretion (79, 80, 81, 82). In addition, we confirmed long-chain *S*-acylation of Gasdermin D (GSDMD) at C191 (Appendix A), a site recently linked to pore formation during macrophage pyroptosis (43, 44, 45, 83).

To assess whether changes in site abundance were driven by protein-level changes, we compared log_2_ fold changes of individual sites with those of their corresponding parent proteins, where available. In total, 875 sites could be matched with protein-level fold changes (Supplemental Table S2-3). For most sites, changes reflected alterations in parent protein abundance (Fig. 3*D*), although several exceptions were observed. For example, C21 of AAA^+^ ATPase Torsin-4A (TOR4A) was significantly upregulated (log_2_ FC = 4.03, *q*-value = 0), while its protein abundance remained unchanged after stimulation (log_2_ FC = 0.26, *q*-value = 0.27) (Fig. 3*D*, Supplemental Table S2).

Furthermore, we found five C-terminal long-chain *S*-acylation sites upregulated on the immune-specific SNARE protein Syntaxin-11 (STX11): C279-280, C282-283, and C285 that were only partially explained by changes in protein abundance (Fig. 3*D*, Supplemental Table S2, Appendix A). Unlike other Syntaxins, STX11 lacks a transmembrane domain. Instead, its C-terminal long-chain *S*-acylations function as a lipid anchor for membrane association and for its polarization to the immunological synapse in natural killer cells (84). In macrophages, STX11 localizes primarily to late endosomal membranes, where it facilitates trafficking between late endosomal compartments and either the plasma membrane or lysosomal compartments (85, 86). Reduced *S*-acylation of STX11 impairs its membrane association (86). The observed site-specific increase in long-chain *S*-acylation, independent of proportional protein-level changes, therefore, suggests a potential regulatory role for *S*-acylation in modulating STX11 membrane anchoring and function during inflammatory activation.

Given this well-characterized “lipid-anchoring” example, we asked whether terminal long-chain *S*-acylation might represent a broader feature of peripheral membrane proteins lacking canonical transmembrane domains. To address this, we systematically mapped long-chain *S*-acylation sites in our dataset onto the 81 identified parent peripheral membrane proteins (as annotated by Uniprot) to assess positional trends and potential anchoring motifs (Figs. 3*E* and S11). In total, 28 of the 81 identified peripheral membrane proteins harbored more than one long-chain *S*-acylation site. As an illustrative example, we identified N-terminal long-chain *S*-acylation on a shared peptide between tumor suppressor protein Scribble (SCRIB) and Leucine-rich repeat and coiled-coil domain-containing protein 1 (LRCC1) at C4 (Figs. 3*E* and S11, Appendix A). C4 is a previously reported site that is essential for the membrane localization and function of SCRIB (87, 88).

To assess whether spatial patterning extends to transmembrane proteins, we analyzed long-chain *S*-acylation relative to transmembrane domain (TMD) boundaries (25). Consistent with prior findings, we found a strong enrichment of modification sites near TMD edges, with 34 transmembrane proteins harboring *S*-acylation sites within five residues of a TMD boundary (Supplemental Fig. S12). Several such sites are significantly upregulated during inflammation, including GSDMD (C191), CD70 (C17), and Tapasin (TAPBP, C440) (Supplemental Fig. S12*B*, Appendix A). TAPBP, which functions as a chaperone for the MHC class I peptide-loading complex, interacts with the TAP transporter to mediate peptide translocation into the endoplasmic reticulum (Supplemental Fig. S12*B*, inset).

In summary, our findings show that upregulation of long-chain *S*-acylation during macrophage polarization is mainly driven by changes in protein expression, with several exceptions. Furthermore, our findings suggest that long-chain *S*-acylation is spatially organized and might contribute to membrane localization and activity of immune-relevant proteins. In peripheral membrane proteins, terminal cysteine long-chain *S*-acylation can substitute for transmembrane domains, as seen in STX11 and SCRIB. In transmembrane proteins, we observe enrichment of long-chain *S*-acylation sites at TMD boundaries, consistent with prior observations. Inflammatory regulation of several of these sites further supports a broader role for long-chain *S*-acylation as a dynamic and potentially important membrane-targeting mechanism in immune responses.

### Identification of Multi-Site Long-Chain *S*-Acylated Peptidoforms

We identified a subset of 210 proteins with multiple long-chain *S*-acylation sites (Fig. 3*A*, Supplemental Table S2-1). While multi-site proteins are less common than single-site proteins, there are indications for their biological relevance. For example, research by Baskin *et al.* (2022) demonstrated that different patterns of long-chain *S*-acylation on EFR3B direct PI4KIIIα complex I and II to either liquid-ordered or liquid-disordered regions of the plasma membrane (48). Despite this, no study has systematically investigated the *S-*acyl-peptidoform landscape across different stimuli on a global scale. The SILAC ssABE approach is suited to address this question, as it enables the detection of *S*-acyl-peptidoforms derived from parent proteins. By analyzing SILAC ssABE datasets from both M0 and M(LPS + IFNɣ) macrophages, we identified a total of 118 multi-site peptides (peptides containing more than one *S-*acylated cysteine) originating from 102 parent proteins (Supplemental Table S2-1 Appendix B). Peptides with three cysteines showed an approximately equal distribution of one, two, and three *S*-acyl-peptidoforms. In contrast, most peptides with two cysteines exhibited two distinct *S*-acyl-peptidoforms. (Supplemental Fig. S13*A*).

Peptides for which we identified two long-chain *S*-acylation sites (*n* = 92) could be divided into two subclasses: peptides with adjacent sites (*n* = 34), and peptides whose sites were separated by an amino acid spacer (*n* = 58). For more than 70% of adjacent-site peptides, most of the detected abundance corresponded to their doubly *S*-acylated peptidoforms. In contrast, peptides with spaced cysteine sites were more frequently detected in singly *S*-acylated forms (Fig. 4*A*). There are multiple possible explanations for these observations. One possible explanation is that adjacent cysteines are structurally positioned to facilitate simultaneous dual acylation, enhancing membrane anchoring or protein-membrane interactions through increased local hydrophobicity. It is also conceivable that such adjacent sites are preferentially recognized or processed by a single ZDHHC enzyme, potentially through a cooperative mechanism that favors full modification.

Of the 102 total detected multi-site parent proteins, several contained multi-site containing peptides that were significantly upregulated in M(LPS + IFNɣ) macrophages compared to M0 macrophages. Notable examples include CD38 (C15, C16), PLSCR1 (C240, 234, 237, 239; C148, 149, 153, 154) and TRAF1 (C88, C95) (Supplemental Table S2-1, Appendix A), suggesting that these *S*-acylation-rich regions play a role in the inflammatory response.

We mapped the differences in the relative observed intensities of various *S*-acyl-peptidoforms of multi-*S*-acylation site-containing peptides between M(LPS + IFNɣ) and M0 conditions across all 118 detected multisite peptides. Interestingly, for the cytoplasmic tryptophan tRNA ligase WARS, where both C305 and C309 were detected, we observed, in M(LPS + IFNɣ) macrophages, an approximately (50/50) distribution of peptides with either C305 *S*-acylated and C309 unmodified or C309 *S*-acylated and C305 unmodified. A negligible portion of the detected intensity corresponded to peptides where both sites were *S*-acylated (Figs. 4*B* and S13*B*). In contrast, the other two-site peptides exhibited more skewed distributions. Tyrosine-protein kinase JAK1 (C10, C16) primarily displayed double *S*-acylation in both M(LPS + IFN_Ɣ_) and M0 macrophages (Figs. 4*C* and S13*C*). The observed site-occupancy patterns differed for transmembrane protein 134 (TMEM134, C108-C109) between M(LPS + IFNɣ) and M0 macrophages. The overall multi-site peptide intensity between conditions was comparable. However, under unstimulated conditions, the detected peptide pool was evenly split between doubly *S*-acylated peptides and those where only C108 was *S-*acylated. Upon stimulation, the observed distribution shifted, with nearly all detected intensity corresponding to peptides where both C108 and C109 were *S*-acylated (Figs. 4*D* and S13*D*).

Intriguingly, we confirm long-chain *S*-acylation of the PI4K2A CCPCC motif within the kinase domain, a modification associated with its active state. This modification enables PI4K2A to stably associate with Golgi and endosomal membranes (89, 90, 91, 92). Previous studies have shown that over 90% of PI4K2A is membrane-bound, suggesting that it remains predominantly active regardless of cellular context (90, 93). Our data is consistent with this, as the majority of the detected signal for the CCPCC-containing peptide corresponds to quadruple *S*-acylation (Figs. 4*E* and S13*E*). Given that PI4K2A lacks a transmembrane domain, it is plausible that such modification contributes to membrane anchoring (Figs. 4*E* and S13*E*).

Our identification of numerous long-chain *S*-acyl-peptidoforms suggest that, like glyco- and phosphoproteoforms, long-chain *S*-acylation may generate distinct functional states regulated by cellular context. However, technical effects must be considered. The observed intensity distributions cannot be assumed to reflect only biological differences. They may partially arise from a combination of technical and biological factors such as differences in capping efficiency, ionization behavior, peptide composition, or cysteine spacing. On the other hand, recent work on the SNAP25 protein family by Mejuto *et al.* (2026), demonstrates that α/β hydrolase domain (ABHD) de-*S*-acylases exhibit distinct substrate selectivity depending on cysteine proximity, with proximal cysteines being less efficiently deacylated in some contexts (94). While this does not exclude technical contributions in our dataset, it supports the possibility that at least part of the observed peptidoform diversity may reflect biologically relevant regulation. In this framework, the frequent detection of doubly occupied proximal cysteines (Fig. 4*A*) could be consistent with limited de-*S*-acylase accessibility or activity at closely spaced sites.

Although the relative quantities and functional relevance of multi-site long-chain *S*-acylation patterns remain underexplored, our data indicate that such modification patterns are relatively common across the proteome. Notably, several inflammation-related proteins also exhibit distinct *S*-acyl-peptidoforms, highlighting the importance of site-specific analysis to uncover how long-chain *S*-acylation may fine-tune protein function during macrophage activation and inflammation.

### Modulating Long-Chain *S*-Acylation with APT and ZDHHC Inhibitors Tunes the Inflammatory Response after LPS + IFNɣ Stimulation

Upon LPS + IFNɣ stimulation, macrophages initiate a robust inflammatory response characterized by the secretion of numerous cytokines and chemokines. The nuclear translocation of some transcription factors involved in this response is, in part, regulated by dynamic protein lipidation events. For instance, STAT3 undergoes ZDHHC7-mediated *S*-acylation for targeting to the plasma- and endomembrane, while its deacylation by APT2 facilitates nuclear translocation (32). Building on our identification of many long-chain *S*-acylation sites in pro-inflammatory macrophages, we hypothesized that pharmacological modulation of the regulatory enzymes of long-chain *S*-acylation could alter the magnitude of the inflammatory response.

**Fig. 5:**
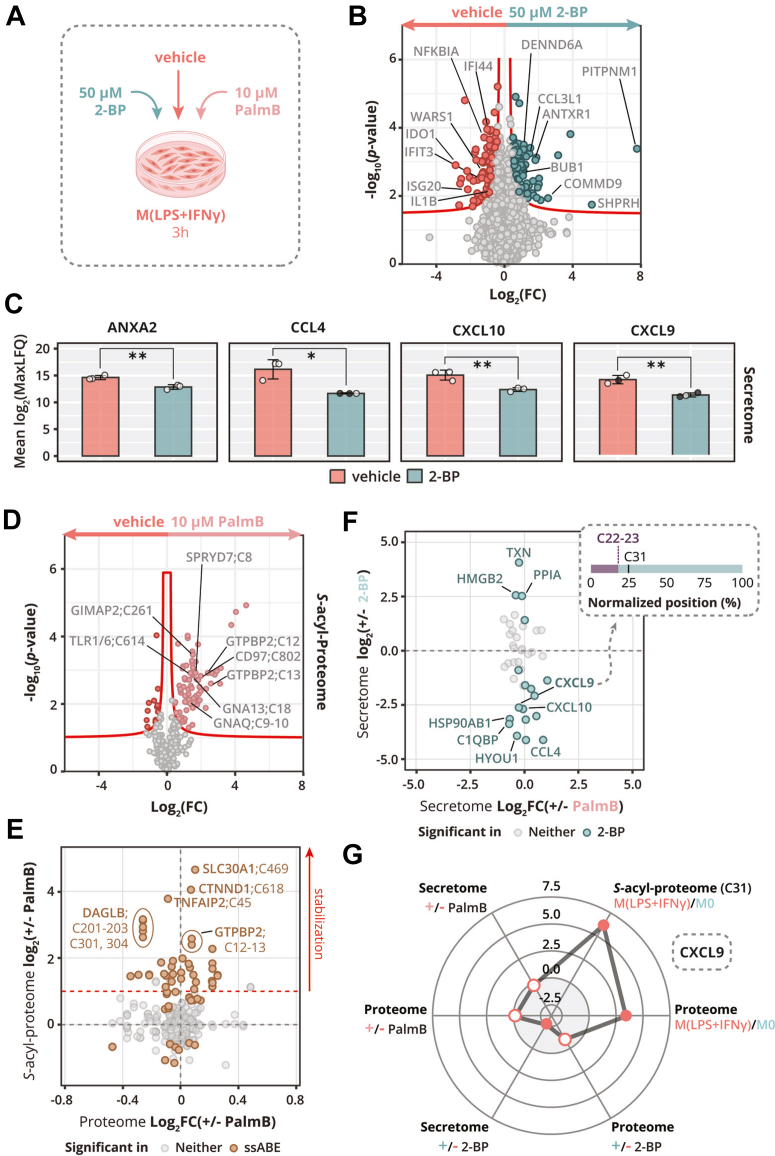
**Proteome, *S*-acyl-proteome and secretome analysis of pro-inflammatory macrophages treated with 50 μM 2-BP or 10 μM PalmB.***A*, experimental workflow. THP-1 monocytes were differentiated as before, but LPS + IFN_ɣ_ stimulation spanned 3 h. Cells were treated with vehicle (DMSO), 50 μM 2-BP or 10 μM PalmB in serum-, phenol-red- and antibiotic-free RPMI-1640. Experiment was done in biological *n* = 3. *B*, Volcano plot of proteins enriched in M(LPS + IFN_ɣ_) macrophages treated with vehicle or 2-BP. Proteins that were present exclusively in either condition are not included in the plot. *Red lines* show FDR <0.05. *C*, Side-by-side bar charts of mean log_2_(MaxLFQ) of secreted proteins. Two-tailed *p*-values were calculated using unpaired parametric *t*-tests to determine significant differences between vehicle and 2-BP treated macrophages (ns = not significant, *∗p* ≤ 0.0332, *∗∗p* ≤ 0.0021, *∗∗∗p* ≤ 0.0002, *∗∗∗∗p* ≤ 0.0001). Datapoints that were imputed have a *black* fill. Data are mean ± SD. *D*, Volcano plot of long-chain *S*-acylation site differential abundances in PalmB and vehicle treated inflammatory macrophages. *Red lines* show FDR <0.05. *E,* comparative plot showing the fold changes of proteins found in both the total proteome and *S*-acyl-proteome of PalmB vs. vehicle treated samples. *Red dashed line* indicates stabilization cut-off fold change = 1. *F*, Comparative plot showing the fold changes of 49 secreted proteins detected in both the 2-BP and PalmB experiments. Inset shows the normalized protein length of CXCL9. *Black line* indicates a putative long-chain *S*-acylation site detected in the benchmark list. The signal peptide is *purple*. *G*, radial plot showing fold changes for CXCL9 compared to the control across several datasets. Radial layers representing negative fold changes are colored *grey*. Datapoint border color is defined by whether the fold change is positive or negative and the accompanying condition. Datapoints are filled if the fold change is significant. Datapoints are empty if fold change is not significant. If no “M” polarization is assigned, the treatments were done on M(LPS + IFN_ɣ_) macrophages.

To test our hypothesis, we treated M(LPS + IFNɣ)-polarized THP-1 macrophages with either vehicle (DMSO), the non-specific ZDHHC inhibitor 2-bromopalmitate (2-BP, 50 μM for 1 h 15 min), or the broad-spectrum protein-thioesterase inhibitor Palmostatin B (PalmB, 10 μM, 2 h) (Fig. 5*A*). We then analyzed the proteome and secretome from both 2-BP, and PalmB treated cells. In addition, we isolated the *S*-acyl-proteome of PalmB treated cells. Comparative analysis across these three proteomic datasets allowed us to investigate the role of long-chain *S*-acylation in modulating inflammatory protein expression and secretion.

The abundances of 165 proteins were significantly increased in 2-BP treated M(LPS + IFNɣ) macrophages, compared to 107 in vehicle treated M(LPS + IFNɣ) macrophages (Supplemental Table S3-1). Interestingly, a pro-inflammatory signature comprising 37 proteins upregulated after 24 h of LPS and IFNɣ stimulation (Supplemental Fig. S14*A*), including IDO1, ISG20, WARS, and SLAMF7, was reduced in 2-BP-treated cells following 3 h stimulation relative to vehicle-treated cells (Supplemental Table S3-1). This suggests a potential attenuation of the inflammatory response. To contextualize the widespread proteome changes observed upon 2-BP treatment (Fig. 5*B*), we assessed cell viability by trypan blue exclusion, which revealed a ∼20% decrease compared to vehicle (Supplemental Fig. S14*B*). Consequently, part of the observed remodeling effects might be due to 2-BP off-target effects and cytotoxicity. In contrast, PalmB treatment left the proteome unchanged (Supplemental Fig. S14, *C* and *D*, Supplemental Table S3-3).

We identified 593 proteins in the secretome of the 2-BP dataset, of which 57 were classified as secreted proteins according to the human protein atlas (HPA) or Uniprot (Supplemental Table S3-2). Of these, 28 have signal peptides according to SignalP 6.0 predictions. Given the use of detergents for secretome solubilization, we next assessed whether extracellular vesicle (EV)-associated proteins were also recovered. To evaluate this, we compared our dataset with ExoCarta (Supplemental Table S3-7), a curated database of exosomal proteins, RNAs, and lipids (95), as well as with a 2017 study profiling EVs from THP-1 macrophages upon *Candida albicans* (*C. albicans*) stimulation (96). Nearly 80% of proteins in our dataset overlapped with entries in ExoCarta, and ∼40% overlapped with EV proteins identified in *C. albicans*-stimulated THP-1 macrophages (Supplemental Table S3-2). These overlaps suggest that our secretome preparation likely captures both EV-associated proteins and conventionally secreted proteins.

Among the 57 annotated secreted proteins, 15 were significantly downregulated in the 2-BP treated secretomes. We focused on this subset, as increased cell death can lead to passive protein release into the extracellular space, resulting in an overall increase in abundance of secretome proteins in 2-BP-treated samples. Downregulated proteins are therefore of particular interest as they are less likely to reflect passive leakage. These proteins included pro-inflammatory chemokines CXCL9, CXCL10 and CCL4 (Fig. 5*C*, Supplemental Table S3-2). Additionally, the secretion of IGHG1 (FDR <0.05, log_2_ fold change = −4.05) and C1QBP (FDR <0.05, log_2_ fold change = −3.38) was also reduced. Notably, none of the 15 proteins significantly downregulated in the secretome were significantly up- or down-regulated in the proteome (Supplemental Table S3-1).

1520 proteins were detected in the secretome of the PalmB dataset of which about 10% were classified as secreted/predicted to be secreted, with functions such as “Enzyme”, “Immunity”, “Cytokine”, and “Chemokine” as classified by HPA (Supplemental Fig. S14, *E* and *F*, Supplemental Table S3-4). Like the proteome, PalmB left the secretome unchanged (Supplemental Table S3-4). The pronounced effects of 2-BP, compared to the minimal impact of PalmB on the proteome and secretome, probably stem from 2-BP’s greater cytotoxicity and non-specificity, as well as the more disruptive cellular consequences of inhibiting long-chain *S*-acylation addition rather than its removal.

While PalmB treatment did not significantly alter the inflammatory macrophage proteome or secretome, we observed clear stabilizing effects at the *S*-acyl-proteome level. Specifically, 93 long-chain *S*-acylation sites were significantly stabilized following 2 h of PalmB treatment (Supplemental Table S3-5). Gene ontology (GO) analysis of the parent proteins (biological process) revealed an enrichment for the adenylate cyclase-activating GPCR signaling pathway (fold enrichment = 17.19; raw *p*-value = 1.95e–08; FDR = 2.89e–04).

Consistent with this enrichment, we observed stabilization of protein N-terminal long-chain *S*-acylation motifs on several known dynamically modified guanine nucleotide-binding protein subunits, including GNA11 (C9-10), GNA13 (C14,18), GNA15 (C9-10, 13), GNAQ (C9-10), and GTPBP2 (C12-13) (Fig. 5*D*, Supplemental Table S3-5, Appendix A) (97). In addition, long-chain *S*-acylation was stabilized on key inflammation-related proteins previously identified in our SILAC ssABE dataset, including CD38 (C15-16), TNFAIP2 (C45), and the cysteine-rich region of PI4K2A (C174–175, 177–178) (Fig. 5*E*, Supplemental Table S3-5, Appendix A), suggesting that these proteins undergo dynamic long-chain *S*-acylation during inflammation. Interestingly, JAK1 harbors two previously unreported N-terminal long-chain *S*-acylated cysteines at positions C10 and C16, both of which were stabilized by PalmB treatment (Supplemental Table S3-5, Appendix A). Our multi-site peptidoform analysis revealed that JAK1 predominantly exists in its doubly *S*-acylated form (Fig. 4*D*). As a peripheral membrane protein with C10 and C16 positioned near the N-terminus (Fig. 3*E*), it is plausible that dual acylation acts as a lipid anchor to facilitate plasma membrane localization, potentially under dynamic regulatory control. Notably, all identified stabilization events reflect increases in long-chain *S*-acylation stoichiometry, as none of the corresponding proteins showed significant changes at the proteome level (Fig. 5*E*).

Guanine nucleotide-binding proteins are known to undergo N-terminal glycine myristoylation upstream of *S*-acylation sites (98, 99). To explore whether our dataset captured this dual lipidation, we reanalyzed it and identified a peptide from GNAI2 bearing both *N*-myristoylation and adjacent long-chain *S*-acylation (Supplemental Fig. S15*A*). We also detected myristoylation and novel adjacent *S*-acylation sites on Battenin (CLN3, Supplemental Fig. S15*B*), a protein associated with neuroinflammation, and Fibroblast Growth Factor Receptor Substrate 2 (FRS2) (100, 101, 102). These findings demonstrate that our ssABE approach can detect long-chain *S*-acylation sites indirectly, and other non-thioester-linked lipid modifications directly, identifying sites that may play important roles during inflammation.

We also detected stabilization of a long-chain *S*-acylation on a peptide shared by Toll-like receptor one and 6 (C614 and C619, fold change = 1.38, *p*-value = 2.92), key sensors in innate immunity (Fig. 5*D*, Supplemental Table S3-5, Appendix A). Previous studies have shown that TLRs, upon engagement with microbial ligands, rapidly internalize into endocytic recycling compartments (ERCs), where they can initiate downstream signaling and inflammatory gene expression (36, 46, 85, 103, 104). Our finding that TLR1 and/or six are putatively long-chain *S*-acylated at C614 and C619, and this modification is stabilized by PalmB treatment, suggests that these sites may be dynamically regulated. This, in turn, may reflect a mechanism by which TLR1 and/or six cycles between the plasma membrane and ERCs, consistent with established trafficking behavior observed for other TLR family members.

Finally, CXCL9, a key pro-inflammatory chemokine implicated in T cell recruitment and interferon-driven immune responses, emerged as an interesting target of long-chain *S*-acylation in our multiproteomic analysis. We observed significant upregulation of CXCL9 in the whole-cell proteome following LPS + IFNɣ stimulation, consistent with classical M1 macrophage activation (Table 2-2). SILAC ssABE further revealed inducible long-chain *S*-acylation of CXCL9 that could be traced back to either C31 or C33 (Appendix A), located within a conserved CXCL family dicysteine motif proximal to the predicted signal peptide cleavage site (C22-23) (Fig. 3*B*, 5, *F*, *G* and S14*G*). Moreover, pharmacological inhibition of ZDHHC-mediated long-chain *S*-acylation with 2-BP led to a marked reduction in secreted CXCL9 levels, despite no significant change in its intracellular abundance, pointing towards a potential regulatory role for long-chain *S*-acylation in controlling CXCL9 release (Fig. 5, *F* and *G*; Supplemental Table S3-1, S3-2). In contrast, inhibition of deacylation with PalmB had no impact on CXCL9 abundance in the proteome or secretome (Figs. 5*F* and S14*D*; Supplemental Table S3-3, S3-4). These results suggest that addition, rather than removal of long-chain *S*-acylation, is critical for CXCL9 secretion during inflammation. The combined observations support a model in which long-chain *S*-acylation contributes to the regulated secretion of CXCL9 in the inflammatory response.

These integrated proteomic analyses reveal that while 2-BP broadly disrupts inflammatory protein expression, and secretion, PalmB selectively stabilizes a distinct set of *S*-acylation sites, particularly on GPCR-related signaling and immune-regulatory proteins, without altering global proteome or secretome profiles, underscoring its greater specificity. Together, these results demonstrate that modulation of long-chain *S*-acylation, either by blocking its addition or attenuating its removal, can differentially influence the inflammatory response, establishing long-chain *S*-acylation as a tunable regulatory axis in macrophage activation.

## Conclusions

We applied SILAC-based ssABE to THP-1 macrophages and generated a benchmark dataset of 3083 putative long-chain *S*-acylation sites across M0 and M(LPS + IFNɣ) polarization states. Our proteome-wide analyses confirmed expected polarization markers and revealed a dynamic, inflammation-sensitive long-chain *S*-acylation landscape. This included both previously known sites, such as P2RX7 (C5, C6, C7) and GSDMD (C191), and novel sites on proteins like CLTC (C1205), CD40 (C258), CCR7 (C358), and CD38 (C15, C16).

We further identified *S*-acyl-peptidoforms, defined as coexisting site-specifically modified variants of the same peptide, for over 100 site-containing peptides. In some cases, these species were differentially abundant in M(LPS + IFNɣ) versus M0 macrophages (e.g., WARS C305 and C309). While the extent to which this reflects biological regulation versus technical effects cannot be fully resolved here, the data support the presence of distinct *S*-acylated peptide species, highlighting potential complexity in long-chain *S*-acylation regulation. It also highlights the critical importance of site-specific profiling, as such information would be obscured by protein-level ABE approaches.

Pharmacological perturbation of long-chain *S*-acylation had divergent effects. Inhibition of long-chain *S*-acylation addition with 2-BP significantly suppressed secretion of key pro-inflammatory chemokines (CXCL9, CXCL10, and CCL4) and attenuated broader inflammatory protein expression. Inhibition of deacylation with PalmB stabilized long-chain *S*-acylation at specific regulatory nodes, particularly GPCR signaling proteins, without altering the global proteome or secretome.

It is important to note that ABE-based methodologies capture thioester-linked cysteine modifications broadly and do not discriminate between long-chain and other acyl species. While long-chain *S*-acylation is generally considered the predominant thioester-linked acyl modification in mammalian cells, some caution is warranted when attributing the observed inflammatory phenotypes exclusively to long-chain *S*-acylation, as a minor subset may originate from non-long-chain *S*-acylation events.

Nevertheless, taken together, these findings support a model in which long-chain *S*-acylation is a dynamically regulated and consequential feature of macrophage activation. Moreover, they suggest that modulating the balance between *S*-acylation and deacylation can influence inflammatory responses. Given the central role of macrophages in inflammation-driven disease, these datasets provide a resource and framework for further investigation of long-chain *S*-acylation in immune regulation and its potential therapeutic relevance.

## Data Availability

The mass spectrometry proteomics data have been deposited to the ProteomeXchange Consortium via the PRIDE partner repository with the dataset identifier PXD066221 (105).

## Supplemental data

This article contains supplemental data (27, 51, 52, 53, 74, 95, 96).

## Conflict of Interest

The authors declare that they have no conflicts of interest with the contents of this article.
