# Supplementary material for: Long-Chain S-Acylation Is a Key Modulator During the Macrophage Inflammatory Response
Source: Mol Cell Proteomics. 2026 Jun 10;25(7):101600. doi: 10.1016/j.mcpro.2026.101600 (PMC13380729; doi:10.1016/j.mcpro.2026.101600)
Supplement: Appendix A [file mmc1.pdf]

Description

Spectra were annotated using Annotator software.

Peak color scheme

- Peaks corresponding to **precursor ions** are colored **purple**.
- Peaks corresponding to ***y*-ions** are colored **darkgreen**.
- Peaks corresponding to ***b*-ions** are colored **lightgreen**.
- Peaks corresponding to ***a*-ions** are colored **lightblue**.
- **Unannotated peaks** are colored **grey**.

Labeling scheme

- Peaks are labelled with their ion type, charge and *m/z*.
- Cysteine residues in the peptide annotations are colored red if they are carbamidomethylated (= long-chain *S*-acylation site). In these cases, the label of their corresponding ion peak is also colored red.
- Cysteine residues in the peptide annotations are colored lightblue if they are modified with *N*-ethylmaleimide (NEM). In this case the label of their corresponding ion peak is also colored lightblue.
- Horizontal dotted lines denote the mass shifts between peaks surrounding the carbamidomethylated or NEM modified cysteines.

Additional information

- The top left of each spectrum contains the following information, from top to bottom:
    - Isotopic label of peptide (heavy, light, or label-free)
        - If heavy, this means that all R and K in the peptide sequence are heavy labelled
    - MS/MS scan number
    - Raw file (refer to PRIDE submission)
    - Precursor charge
- Unlabeled peaks are colored by ion-type, but could also correspond to a water-loss. Ions that are labeled will not belong to water losses unless explicitly stated.

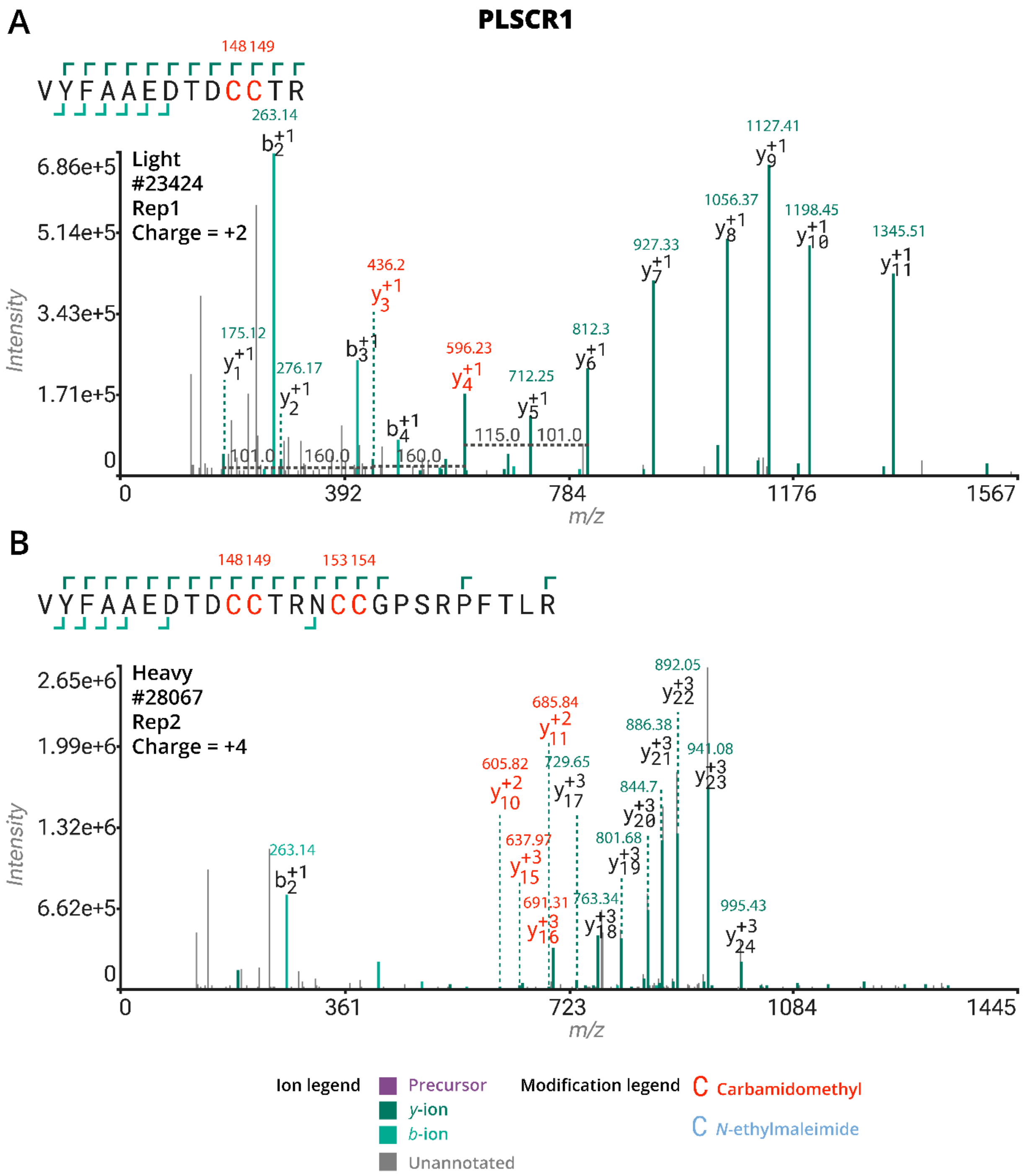
A
PLSCR1
148 149
VYFAAEDTDCCTR
Light
#23424
Rep1
Charge = +2
Intensity
m/z
0 392 784 1176 1567
6.86e+5 5.14e+5 3.43e+5 1.71e+5 0
B
148 149 153 154
VYFAAEDTDCCTRNCCGPSRPFTLR
Heavy
#28067
Rep2
Charge = +4
Intensity
m/z
0 361 723 1084 1445
2.65e+6 1.99e+6 1.32e+6 6.62e+5 0
Ion legend
Precursor
y-ion
b-ion
Unannotated
Modification legend
C Carbamidomethyl
C N-ethylmaleimide

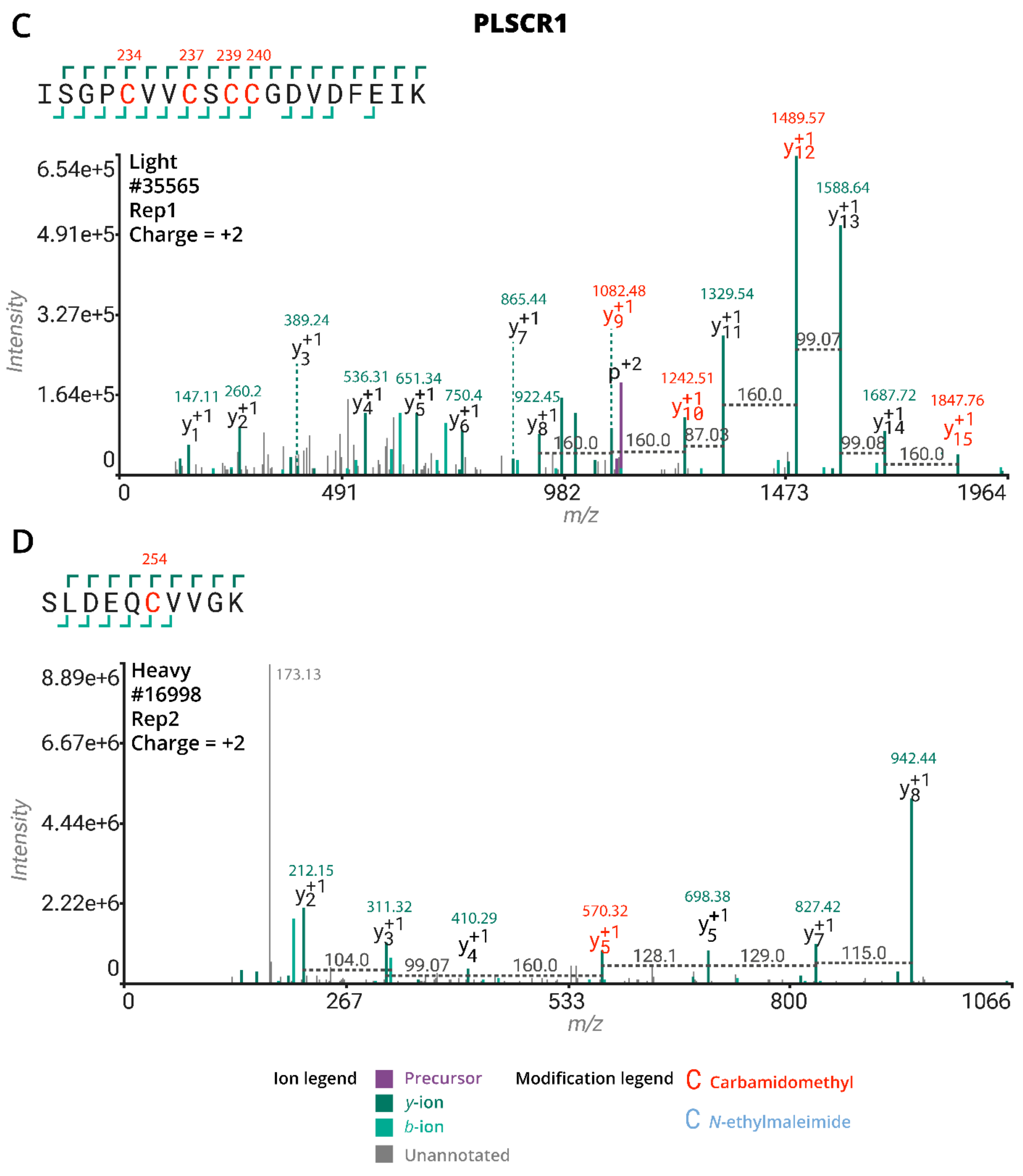

A1. Figure 1. Representive MS2 spectra of long-chain *S*-acylation sites on Phospholipid scramblase 1 (PLSCR1). SILAC ssABE dataset.

(A) C148-149 **(B)** C153-154 **(C)** C234, 237, 239, 240 **(D)** C254

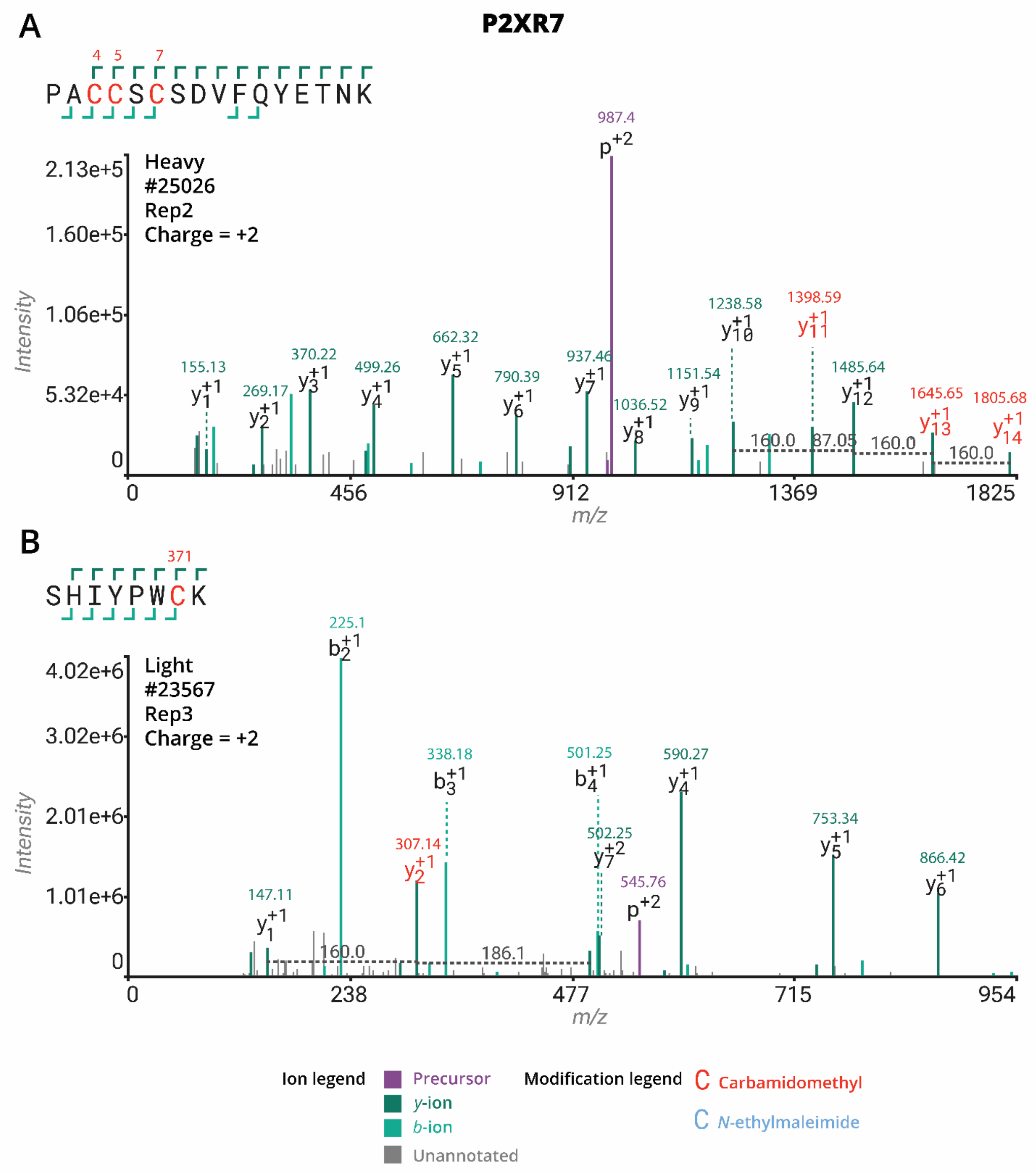
A
P2XR7
PACCSCSDVFQYETNK
Heavy
#25026
Rep2
Charge = +2
Intensity
m/z
B
SHIYPWCK
Light
#23567
Rep3
Charge = +2
Intensity
m/z
Ion legend
Precursor
y-ion
b-ion
Unannotated
Modification legend
Carbamidomethyl
N-ethylmaleimide

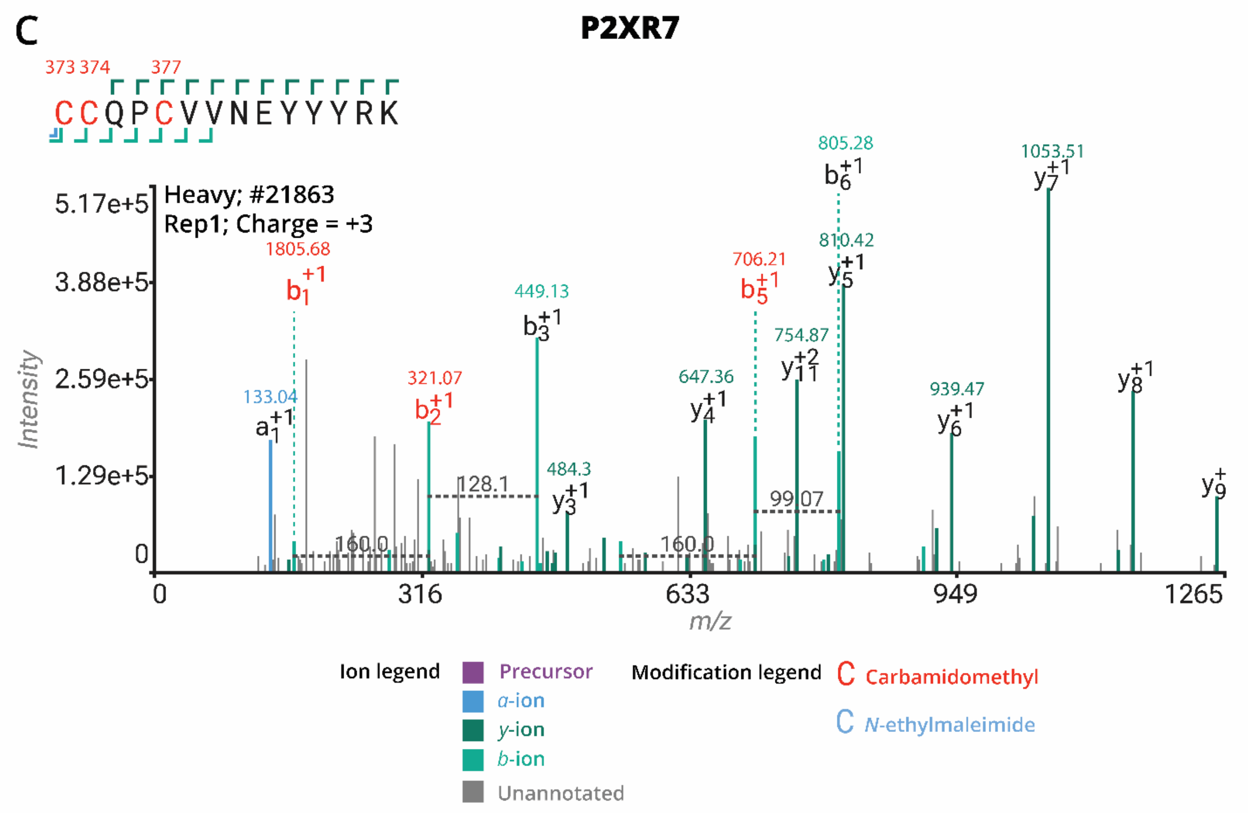

A1. Figure 2. Representative MS2 spectra of long-chain *S*-acylation sites on Purinoceptor 7 (P2XR7). SILAC ssABE dataset.

(B) C4-5, C7 **(B)** C371 **(C)** C373-374, 377.

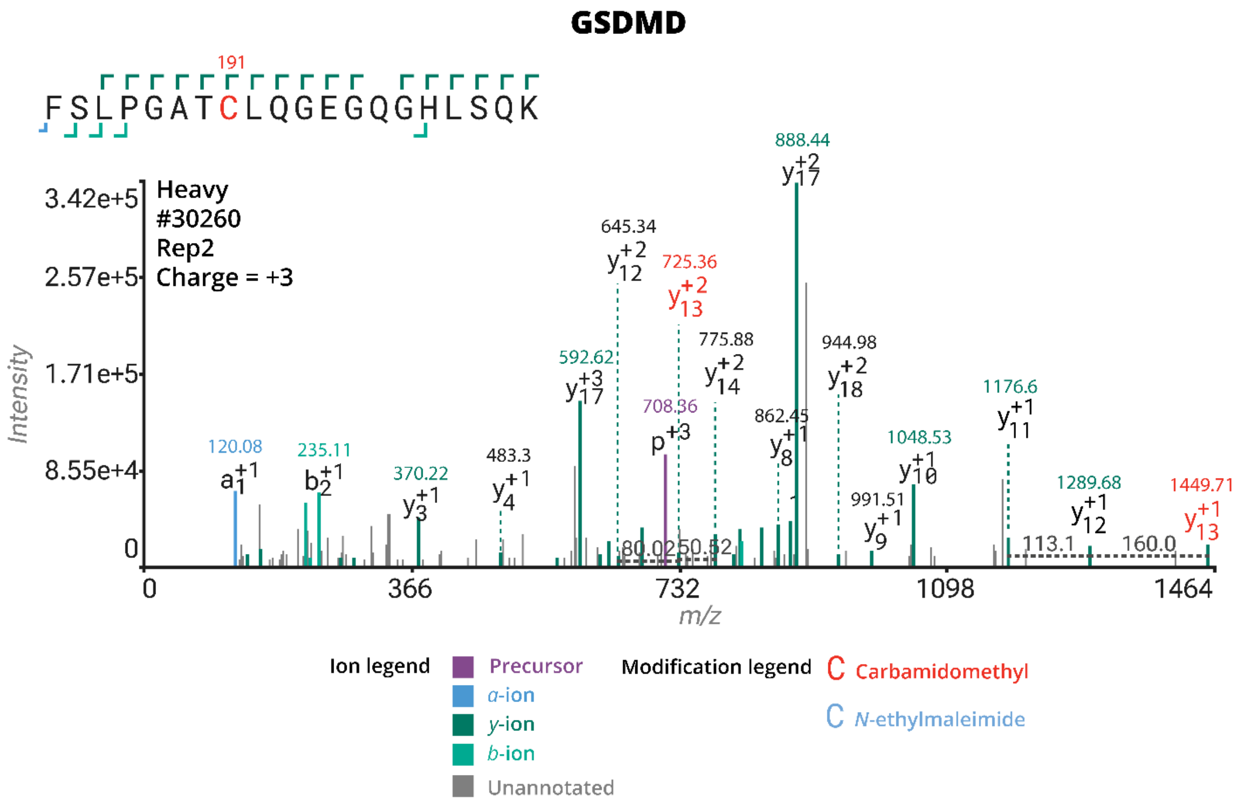

A1. Figure 3. Representative MS2 spectrum of C191 on Gasdermin D (GSDMD). SILAC ssABE dataset.

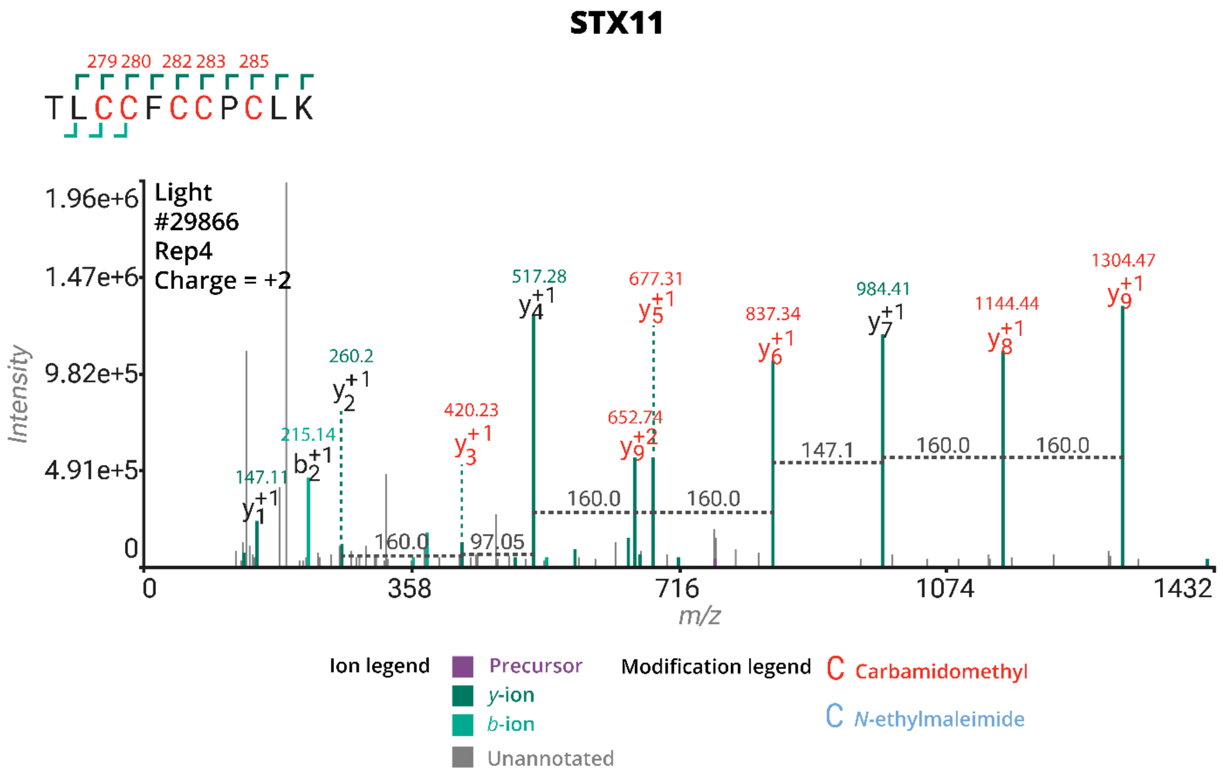

A1. Figure 4. Representative MS2 spectrum of C279-C280, C282-283, and C285 on Syntaxin 11 (STX11). SILAC ssABE dataset.

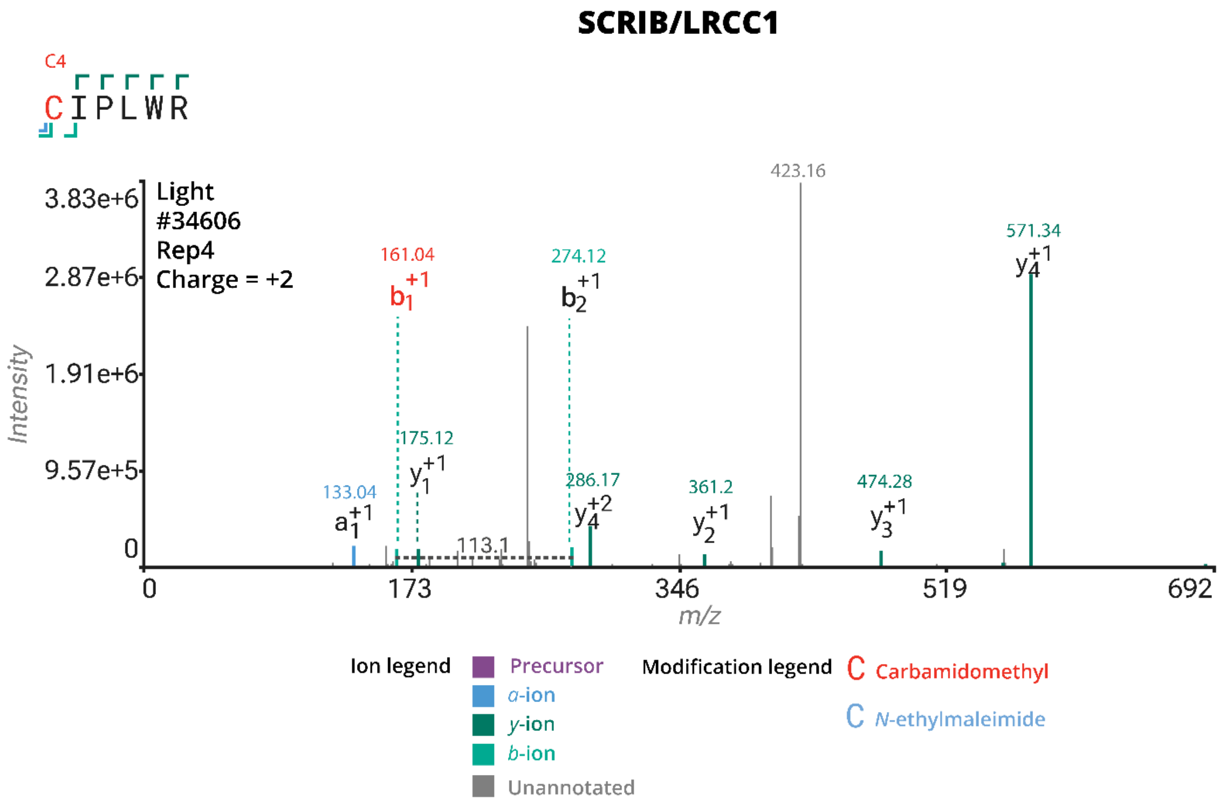

A1. Figure 5. Representative MS2 spectrum of C4 on a shared peptide between SCRIB (Protein scribble homolog) and LRRC1 (Leucine-rich repeat-containing protein 1). SILAC ssABE dataset.

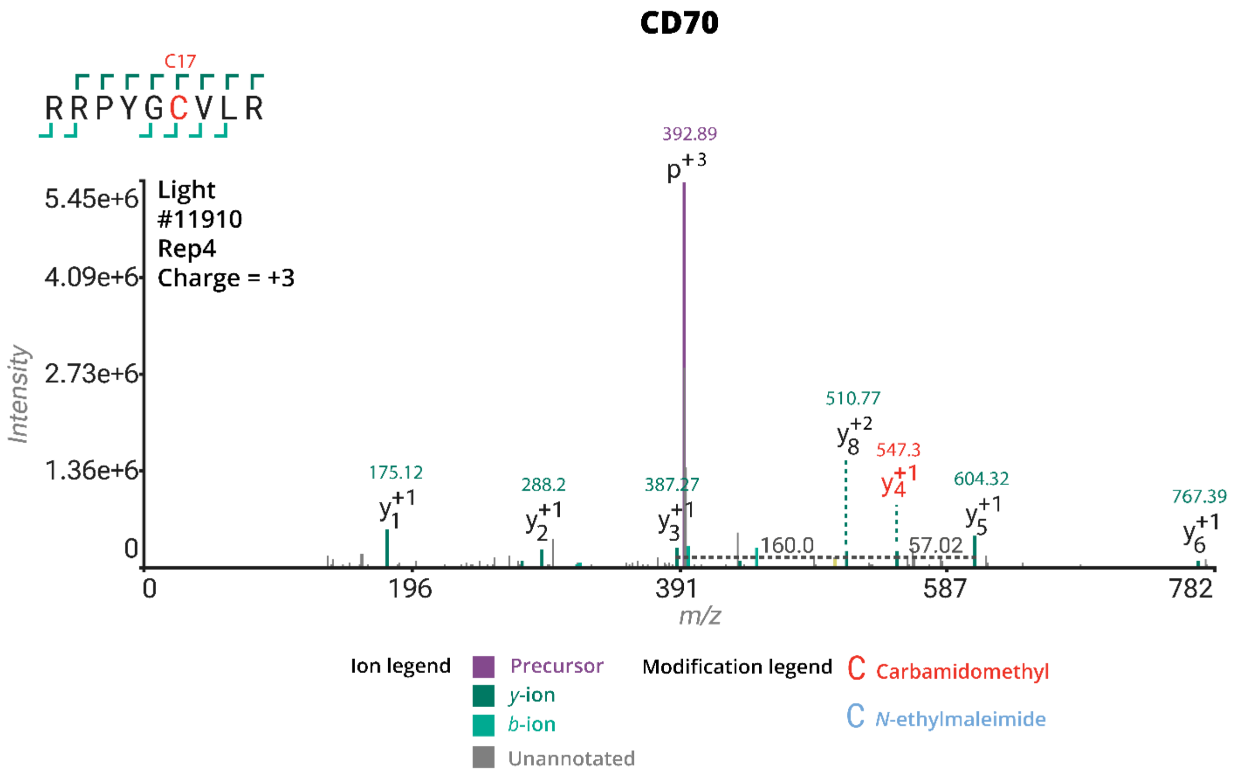

A1. Figure 6. Representative MS2 spectrum of C17 on CD70 (CD70 antigen). SILAC ssABE dataset.

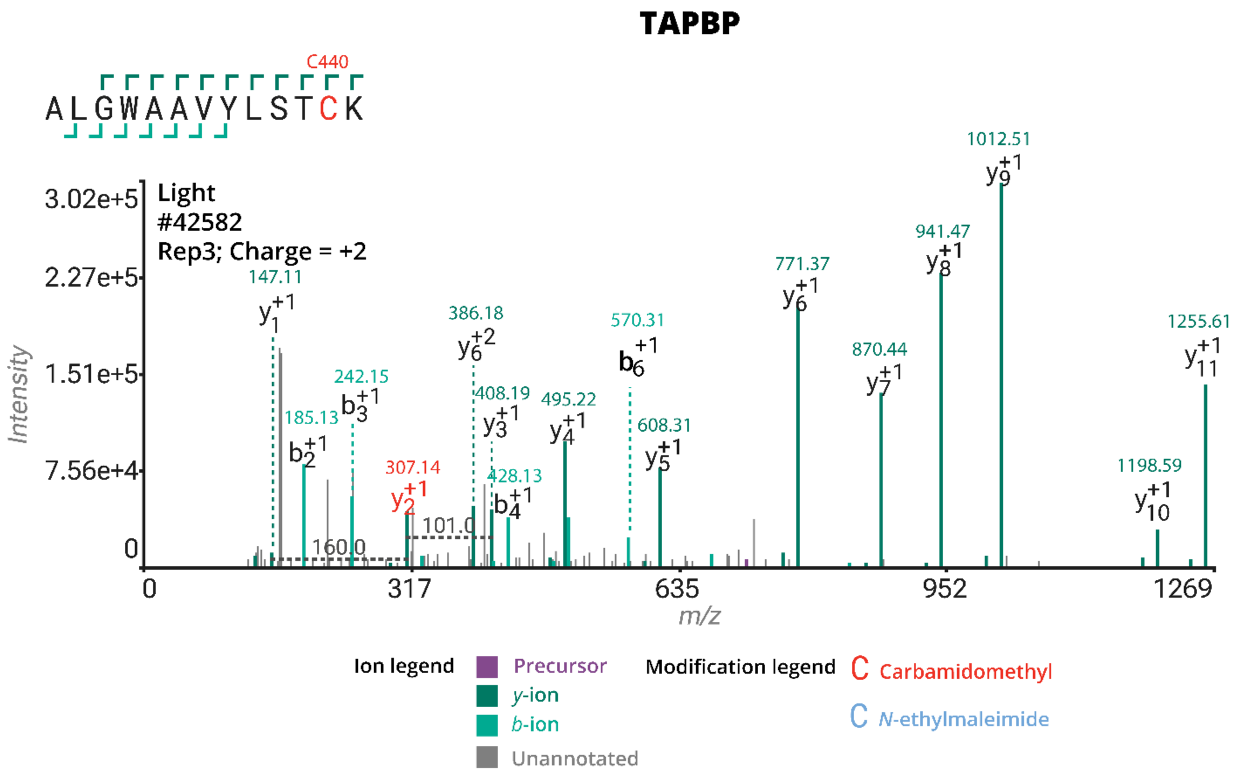

A1. Figure 7. Representative MS2 spectrum of C440 on TAPBP (Tapasin). SILAC ssABE dataset.

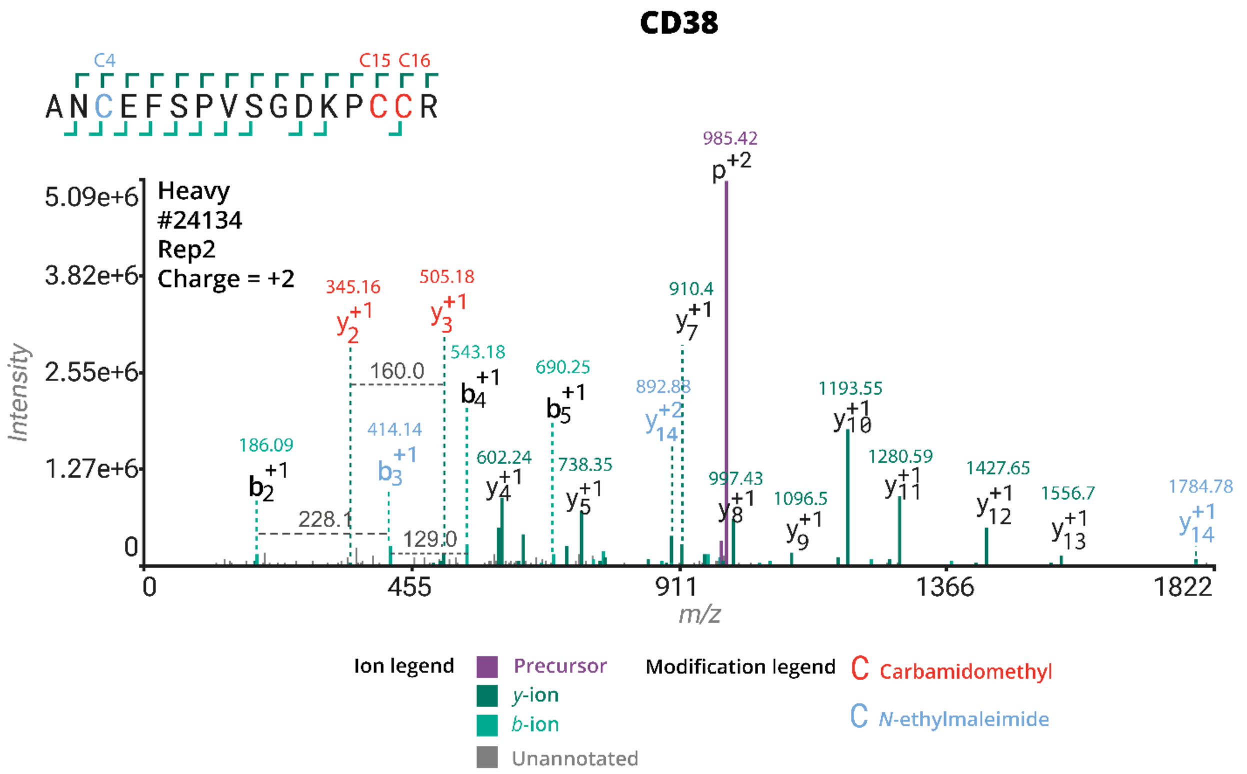

A1. Figure 8. Representative MS2 spectrum of carbamidomethylation at C15-16, and *N*-ethylmaleimide alkylation at C4 on CD38 (ADP-ribosyl cyclase/cyclic ADP-ribose hydrolase 1). SILAC ssABE dataset.

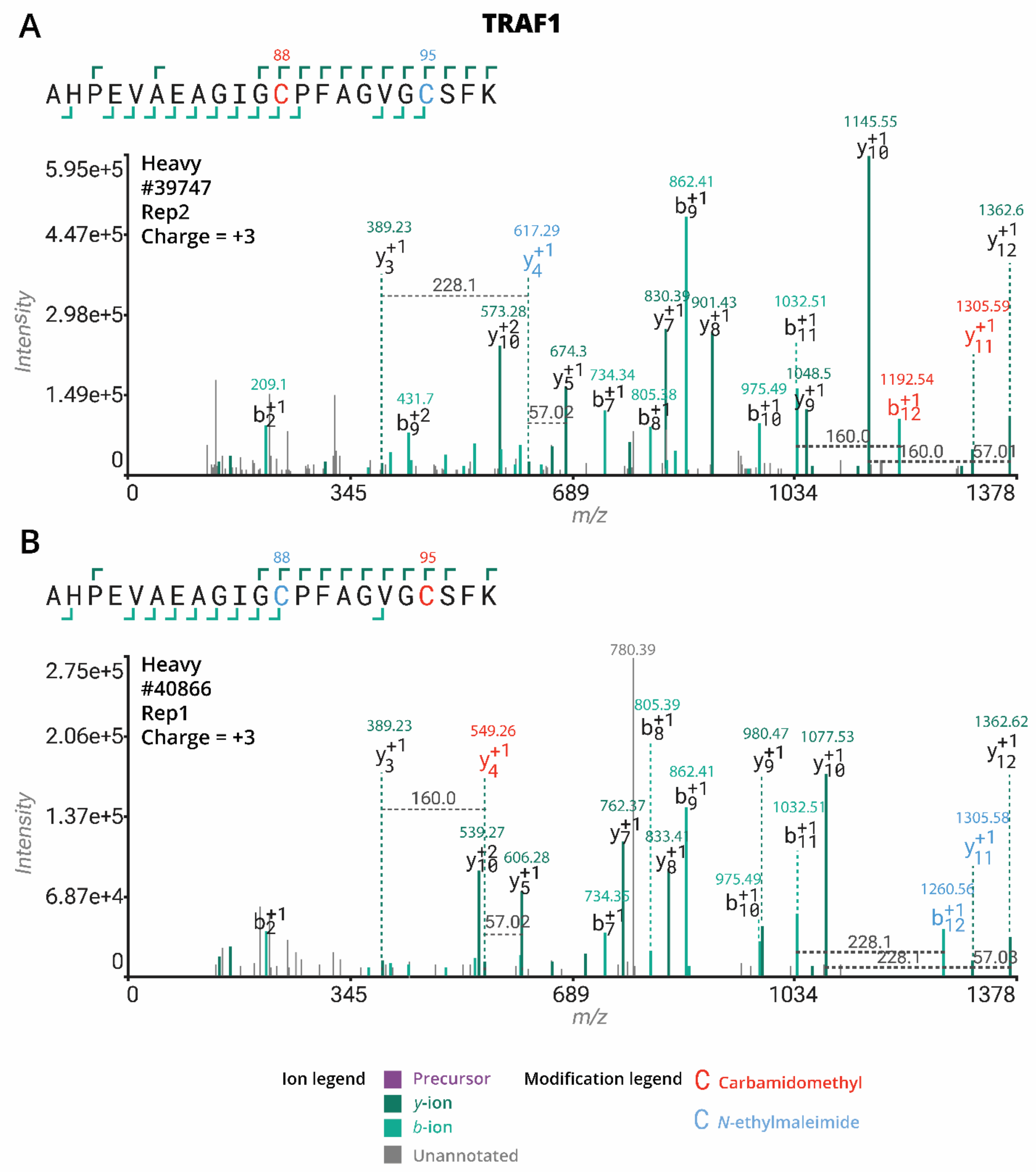

A1. Figure 9. Representative MS2 spectra of long-chain *S*-acylation sites on TNF receptor-associated factor 1 (TRAF1). SILAC ssABE dataset.

(A) C88 **(B)** C95.

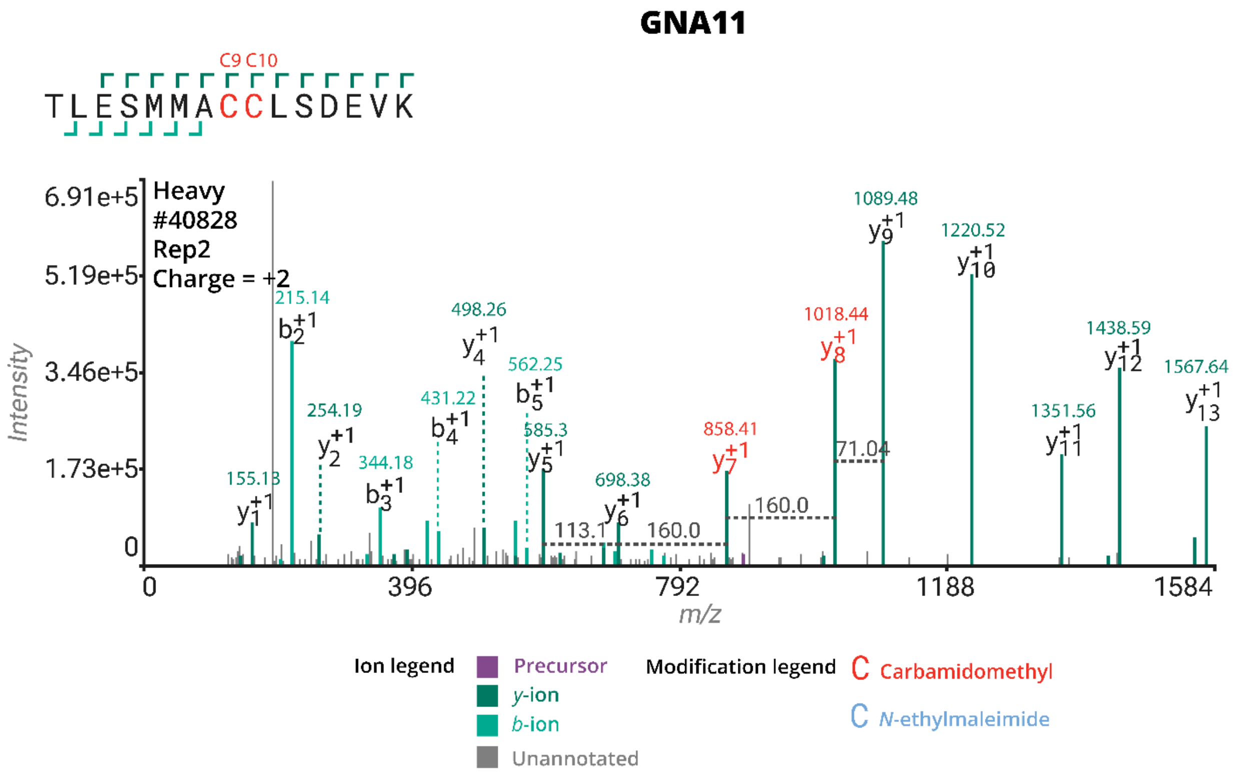

A1. Figure 10. Representative MS2 spectrum of carbamidomethylation at C9-10 on Guanine nucleotide-binding protein subunit alpha-11 (GNA11). SILAC ssABE dataset.

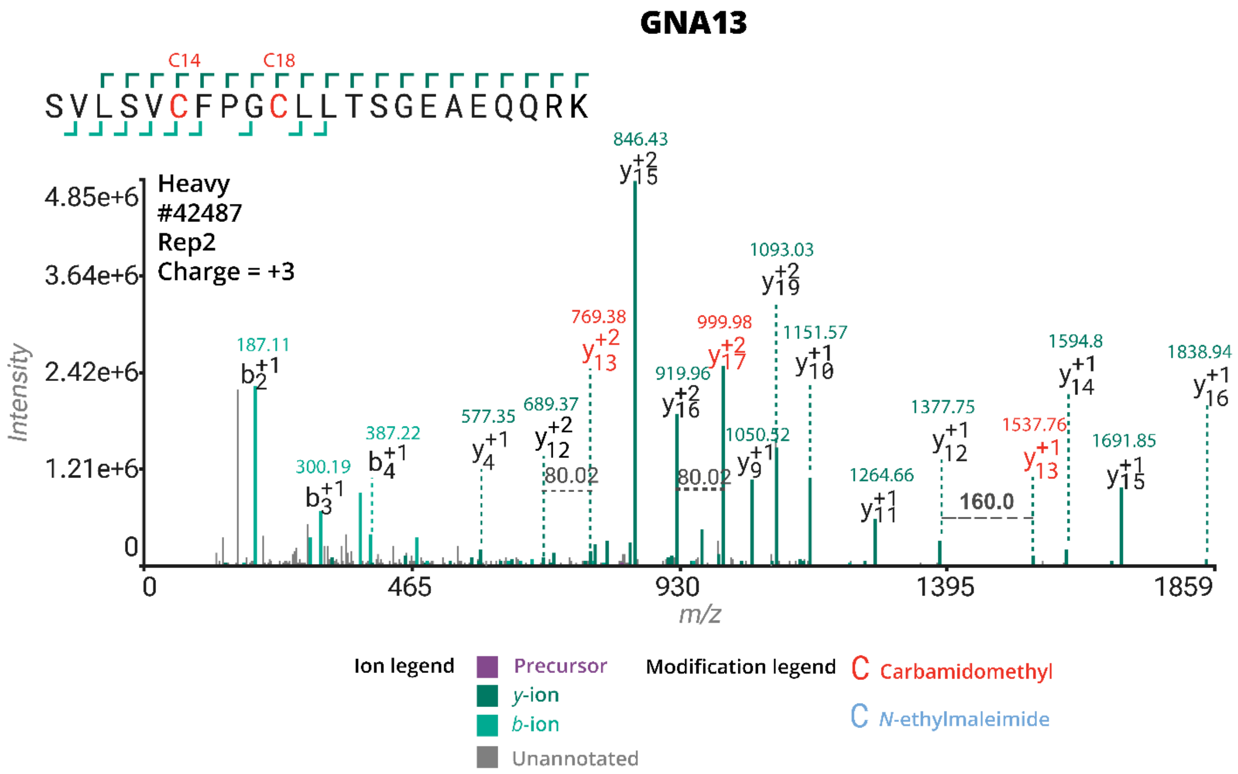

A1. Figure 11. Representative MS2 spectrum of carbamidomethylation at C14 and C18 on Guanine nucleotide-binding protein subunit alpha-13 (GNA13). SILAC ssABE dataset.

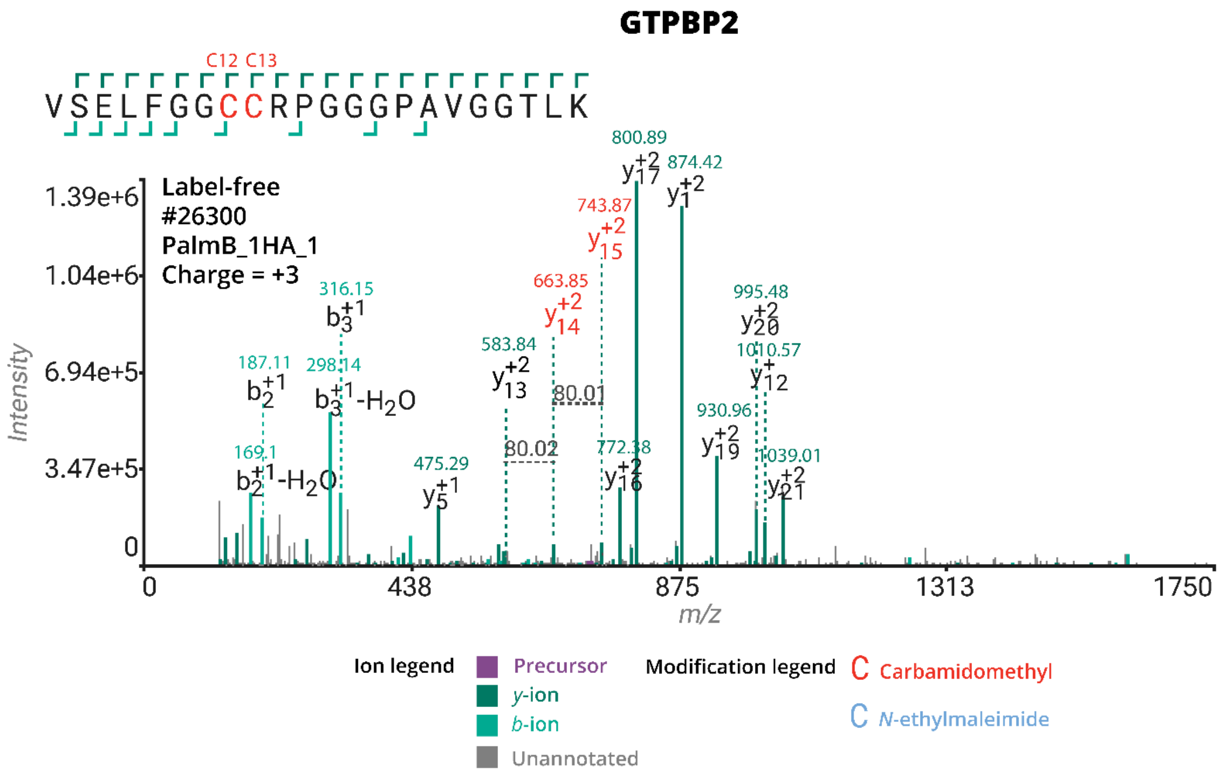

A1. Figure 12. Representative MS2 spectrum of carbamidomethylation at C12 -13 on GTP-binding protein 2 (GTPBP2). Label-free ssABE +/- Palmostatin B dataset.

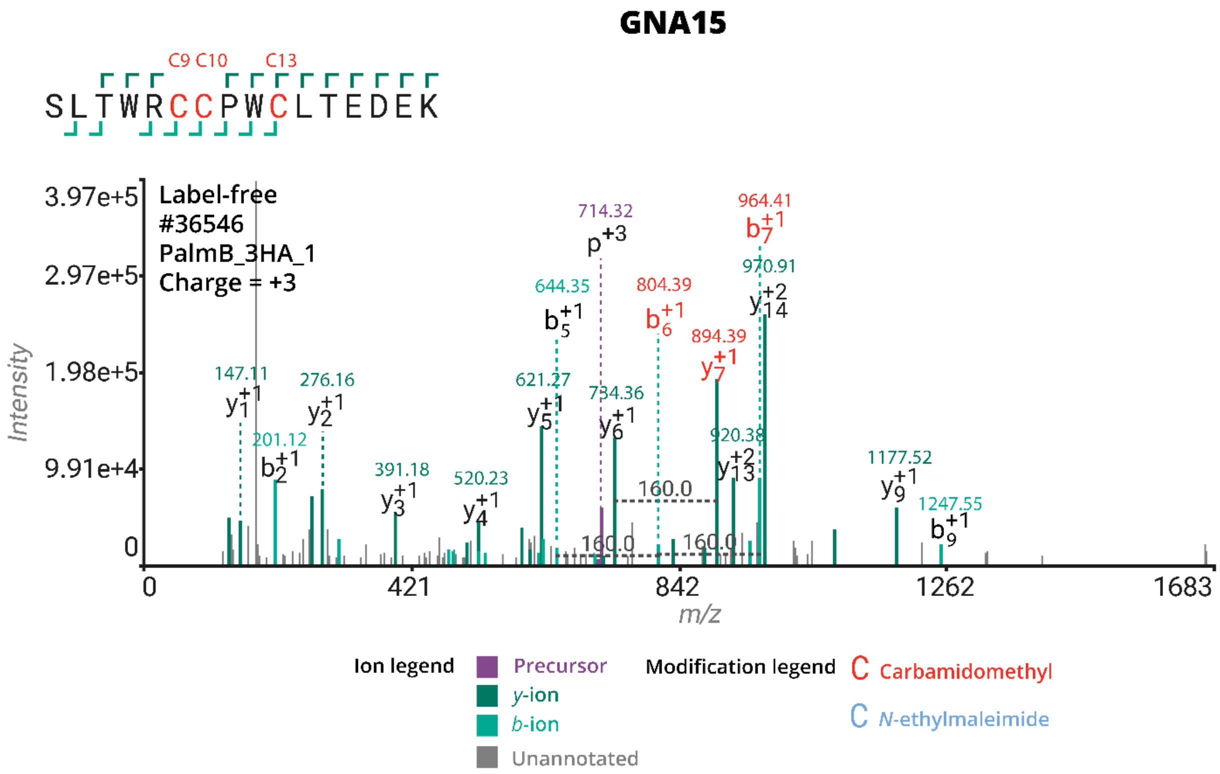

A1. Figure 13. Representative MS2 spectrum of carbamidomethylation at C9 -10, and C13 on Guanine nucleotide-binding protein subunit alpha-15 (GNA15). Label-free ssABE +/- Palmostatin B dataset.

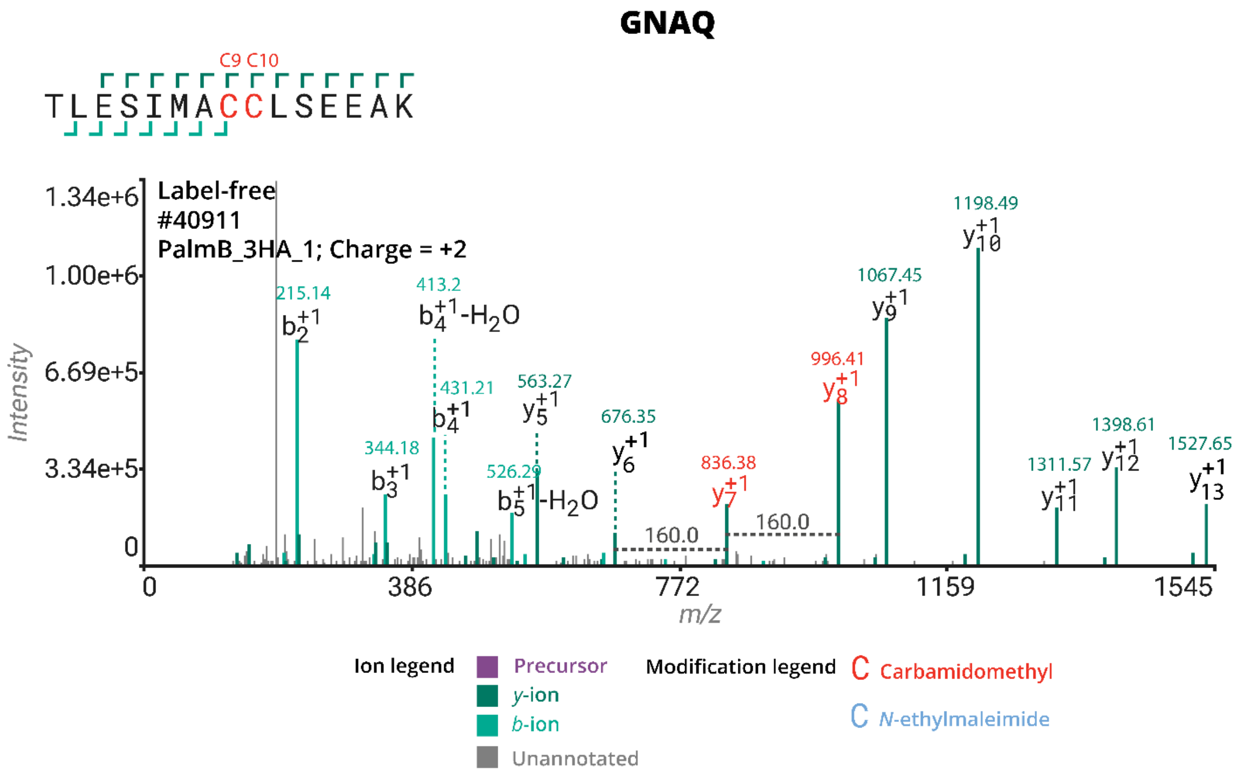

A1. Figure 14. Representative MS2 spectrum of carbamidomethylation at C9 -10 on Guanine nucleotide-binding protein G(q) subunit alpha (GNAQ). Label-free ssABE +/- Palmostatin B dataset.

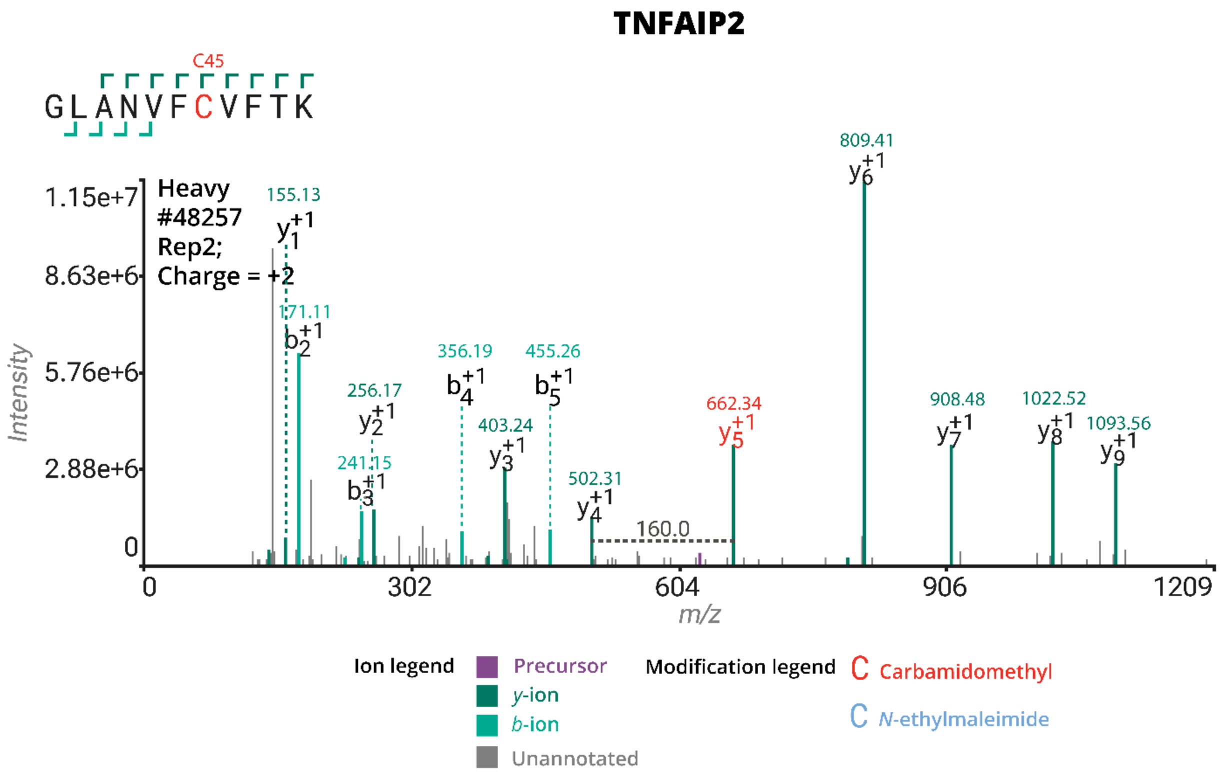

A1. Figure 15. Representative MS2 spectrum of carbamidomethylation at C45 on Tumor necrosia factor alpha-induced protein 2 (TNFAIP2). SILAC ssABE dataset.

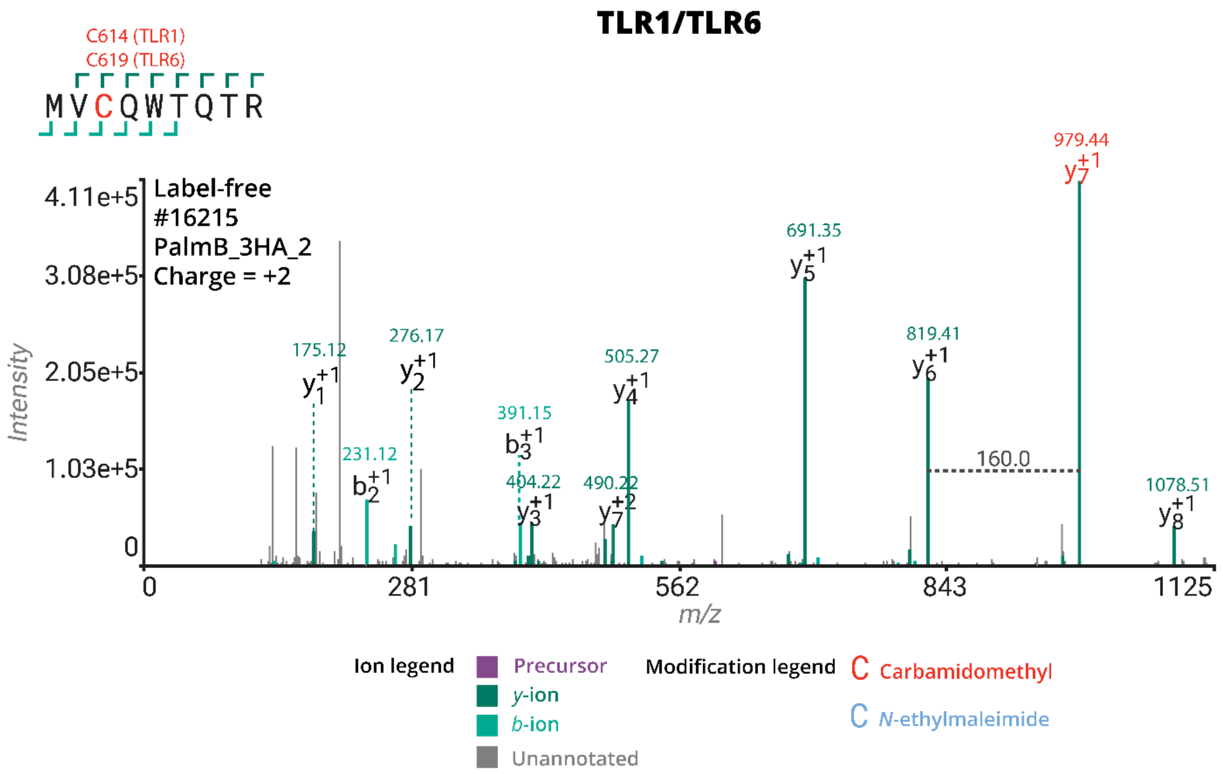

A1. Figure 16. Representative MS2 spectrum of C614/C619 on a shared peptide between Toll-like receptors 1 and 6 (TLR1 & TLR6). Label-free ssABE +/- Palmostatin B dataset.

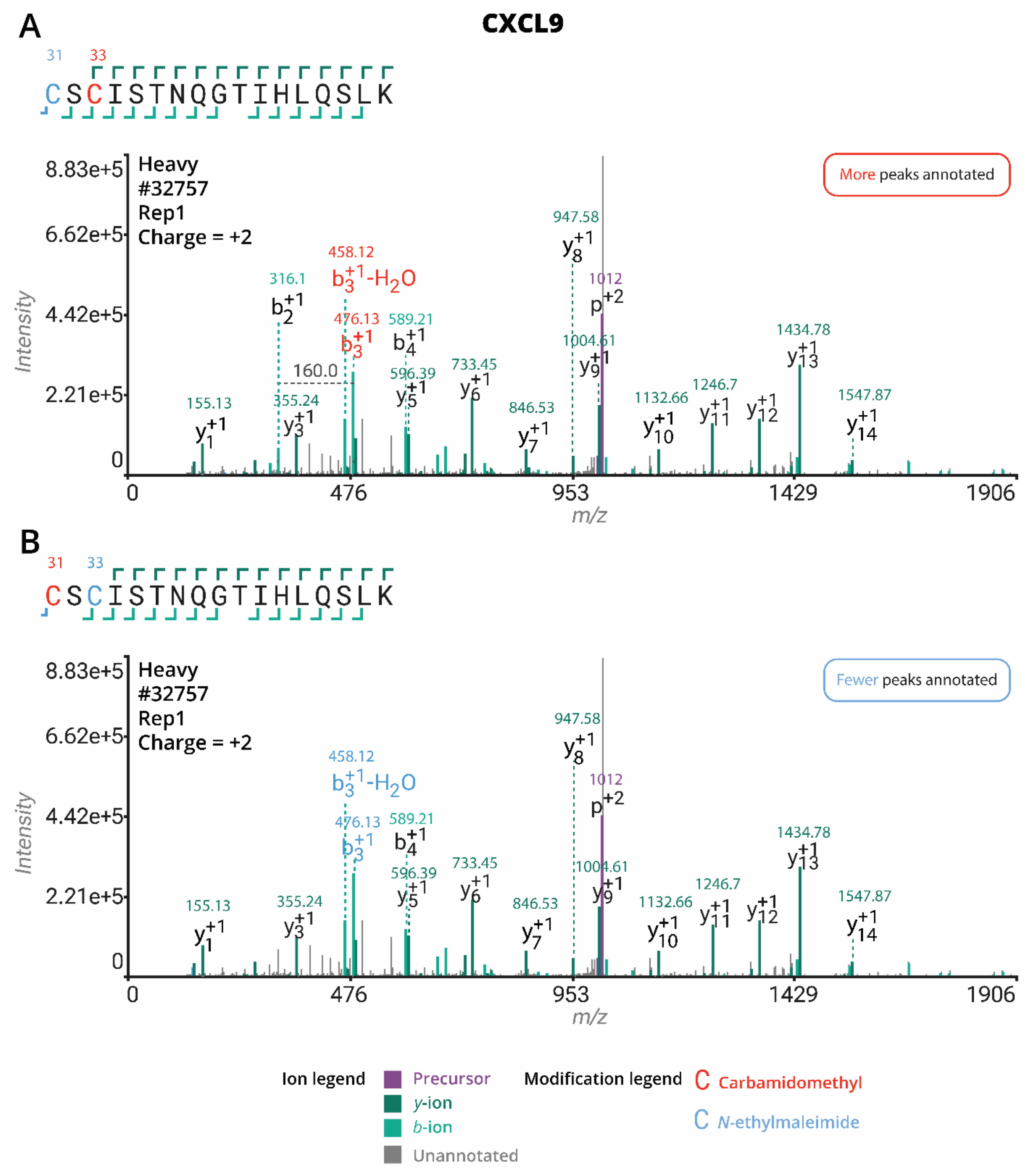
A
CXCL9
31 33
CSCISTNQGTIHLQSLK
8.83e+5
6.62e+5
4.42e+5
2.21e+5
0
Intensity
Heavy
#32757
Rep1
Charge = +2
More peaks annotated
0
476
953
1429
1906
m/z
B
31 33
CSCISTNQGTIHLQSLK
8.83e+5
6.62e+5
4.42e+5
2.21e+5
0
Intensity
Heavy
#32757
Rep1
Charge = +2
Fewer peaks annotated
0
476
953
1429
1906
m/z
Ion legend
Precursor
y-ion
b-ion
Unannotated
Modification legend
C Carbamidomethyl
C N-ethylmaleimide

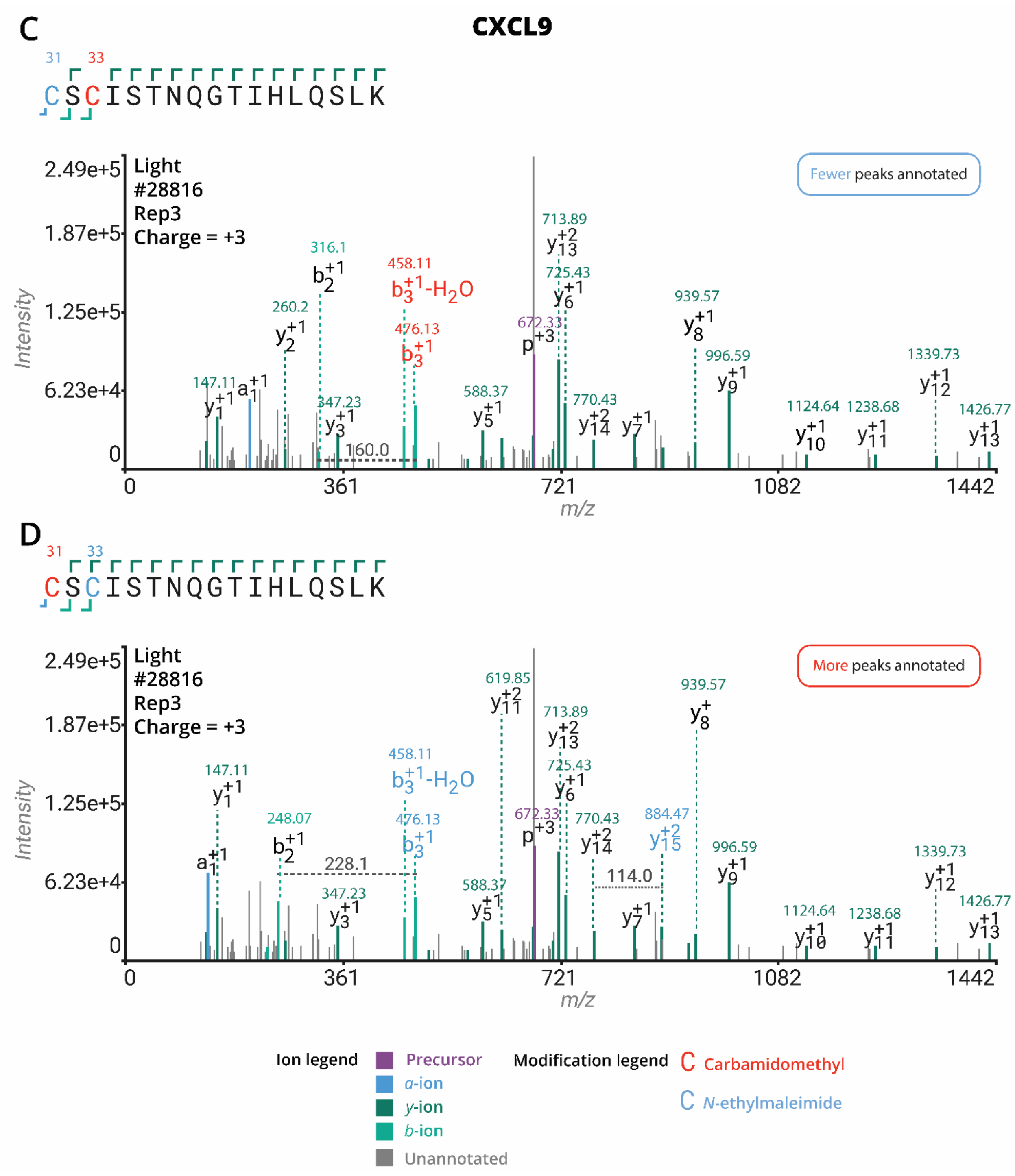

A1. Figure 17. Representive MS2 spectra of long-chain *S*-acylation sites on C-X-C motif chemokine 9 (CXCL9). SILAC ssABE dataset.

(A-B) Annotated spectrum of MS/MS scan number 32757 (Rep1) where long-chain S-acylation sites are localized to either **(A)** C33 or **(B)** C31. **(C-D)** Annotated spectrum of MS/MS scan number 28816 (Rep3) where long-chain S-acylation sites are localized to either **(C)** C33 or **(D)** C31.

Brief user guide for annotating MS2 spectra with Annotator

Identifying the MS2 spectrum to visualize

1. Identify the long-chain *S*-acylation site of interest and the peptide sequence to which it localizes (e.g., by consulting “Supporting Table S2-2”).
2. Open the ***evidence.txt*** file from the MaxQuant output corresponding to the dataset of interest.
3. Locate the peptide sequence in the “Sequence” column.
4. Refine your selection using the “Modified sequence” column to specify the desired combination of modifications (e.g., carbamidomethylation and NEM, or carbamidomethylation only).
5. To select the highest-confidence PSM with carbamidomethylation localization:
    a. Ensure the localization probability is as high as possible (see “Carbamidomethyl (C) Probabilities” column)
    b. Ensure the score difference is also as high as possible (see “Carbamidomethyl (C) Score Diff” column).
6. Once the PSM is selected, record the following details:
    a. Precursor charge (column “Charge”).
    b. Raw file or experiment name (column “Raw file” or “Experiment”).
    c. Isotopic labeling state, if applicable (column “Labeling State”).
    d. Modifications and their residue positions (column “Modified sequence”).
    e. MS/MS scan number (column “MS MS scan number”).

Visualizing and annotating your spectrum of interest in Annotator

1. Download and install the Annotator software. For details, refer to: *“A Universal Spectrum Annotator for Complex Peptidoforms in Mass Spectrometry-Based Proteomics”* by Douwe Schulte, Rien W. Leuvenink, Shelley Jager, Albert J. R. Heck, and Joost Snijder, published in ACS Analytical Chemistry (2025).
   DOI: 10.1021/acs.analchem.5c02832.
2. Open the Annotator software after installation.
3. Click “Load raw data file” to upload your raw data file.
4. Under “Model,” select “CID.” This ensures that only relevant ion types are annotated and prevents assignment of unsupported ions (e.g., *z*-ions from non-HCD fragmentation).
5. Enter the MS/MS scan number of the spectrum you wish to annotate. Subtract 1 from the actual scan number to load the correct spectrum.
    a. Example: For scan 25546, enter “25545.”

6. Enter your peptide sequence in the “Peptidoform sequence” field.
7. Specify modifications using Unimod notation. Examples of modified peptide sequences are shown below:

Example 1

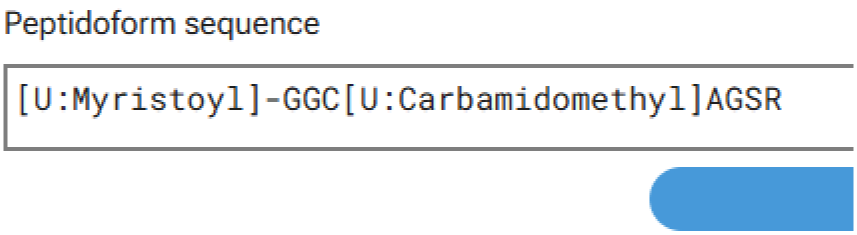

Example 2

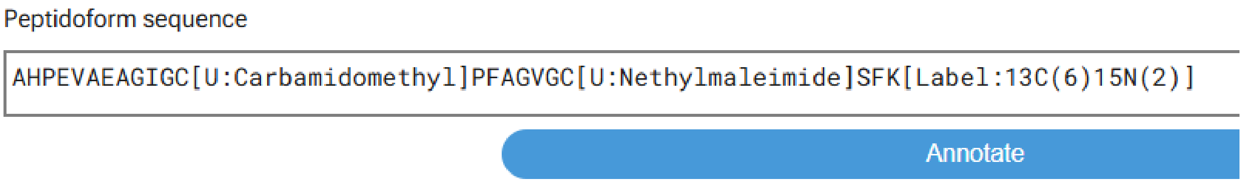

8. Relevant modifications for this study:
    a. **Cysteine carbamidomethylation**: [U:Carbamidomethylation]
    b. **Cysteine *N*-ethylmaleimide alkylation:** [U:Nethylmaleimide]
    c. **N-terminal glycine myristoylation**: [U:Myristoyl]-
9. Relevant isotopic labels for this study:
    a. **Heavy arginine**: [Label:13C(6)15N(4)]
    b. **Heavy lysine**: [Label:13C(6)15N(2)]
10. Click “Annotate” and inspect the resulting spectrum.
11. If modification localization is uncertain (e.g., cysteine assignment of NEM vs. carbamidomethylation), adjust the peptidoform sequence and compare the resulting annotations.
