# Supplementary material for: Long-Chain S-Acylation Is a Key Modulator During the Macrophage Inflammatory Response
Source: Mol Cell Proteomics. 2026 Jun 10;25(7):101600. doi: 10.1016/j.mcpro.2026.101600 (PMC13380729; doi:10.1016/j.mcpro.2026.101600)
Supplement: Appendix B [file mmc2.pdf]

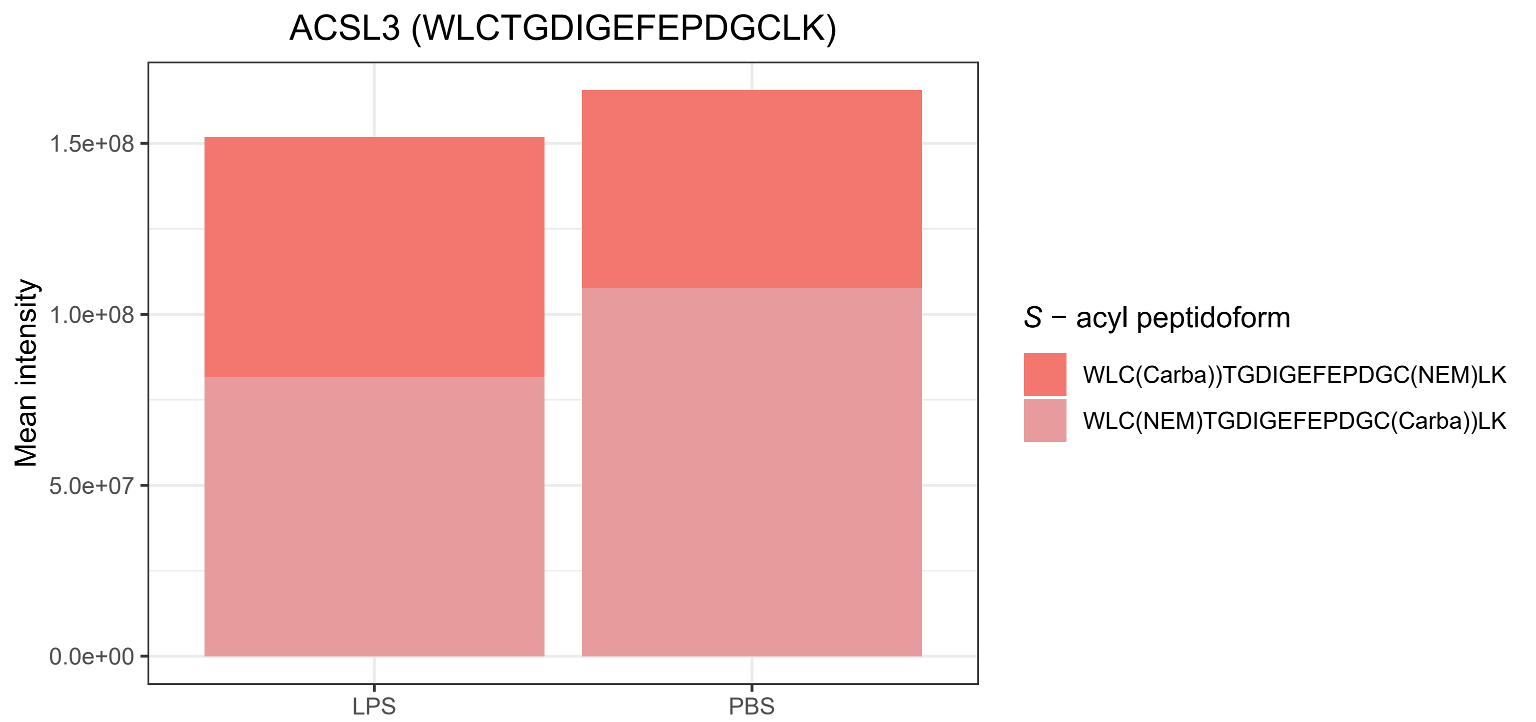

ACSL3 (WLCTGDIGEFEPDGCLK)
Mean intensity
1.5e+08
1.0e+08
5.0e+07
0.0e+00
LPS
PBS
S − acyl peptidoform
WLC(Carba))TGDIGEFEPDGC(NEM)LK
WLC(NEM)TGDIGEFEPDGC(Carba))LK

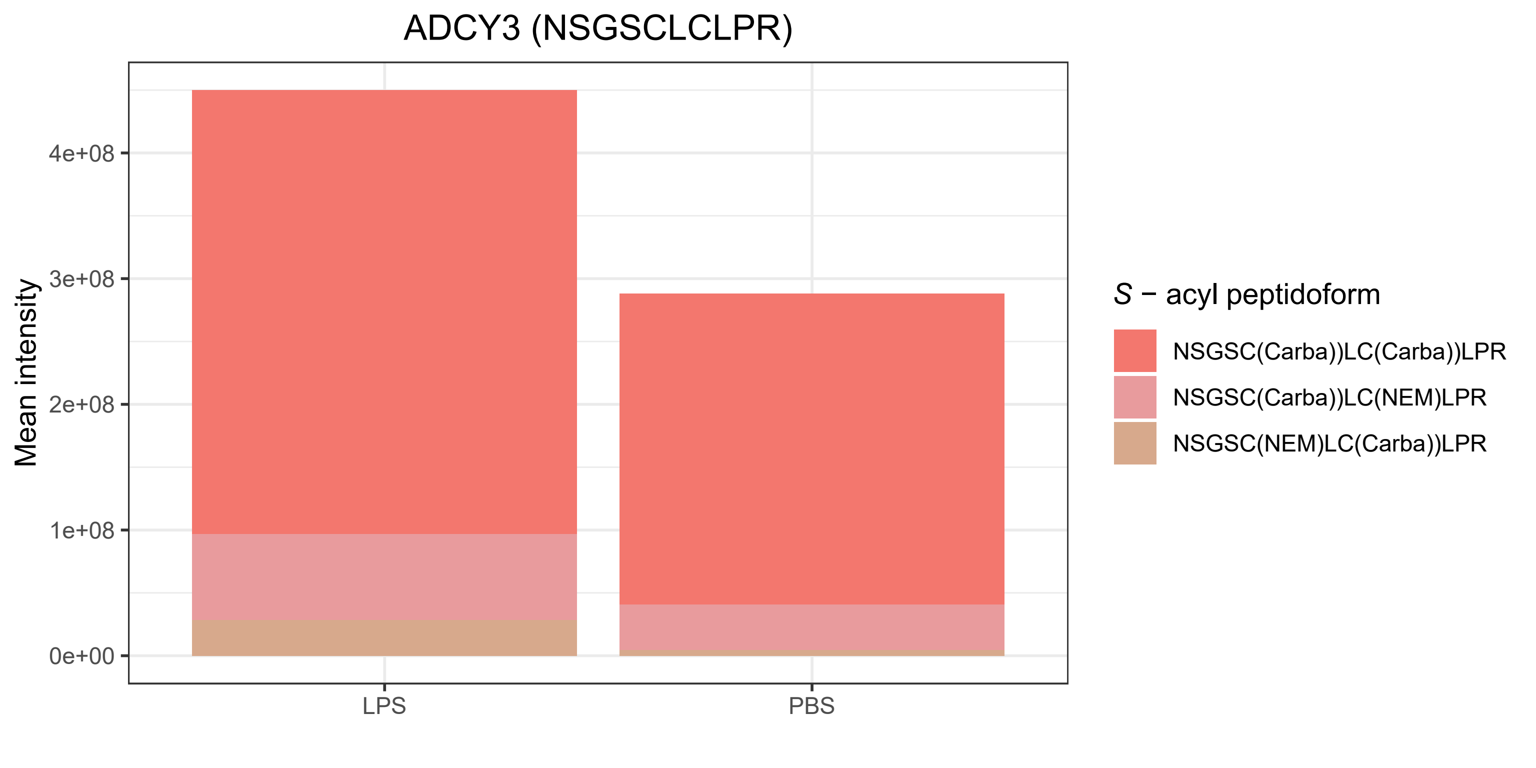

ADCY3 (NSGSCLCLPR)
Mean intensity
4e+08
3e+08
2e+08
1e+08
0e+00
LPS
PBS
S − acyl peptidoform
NSGSC(Carba))LC(Carba))LPR
NSGSC(Carba))LC(NEM)LPR
NSGSC(NEM)LC(Carba))LPR

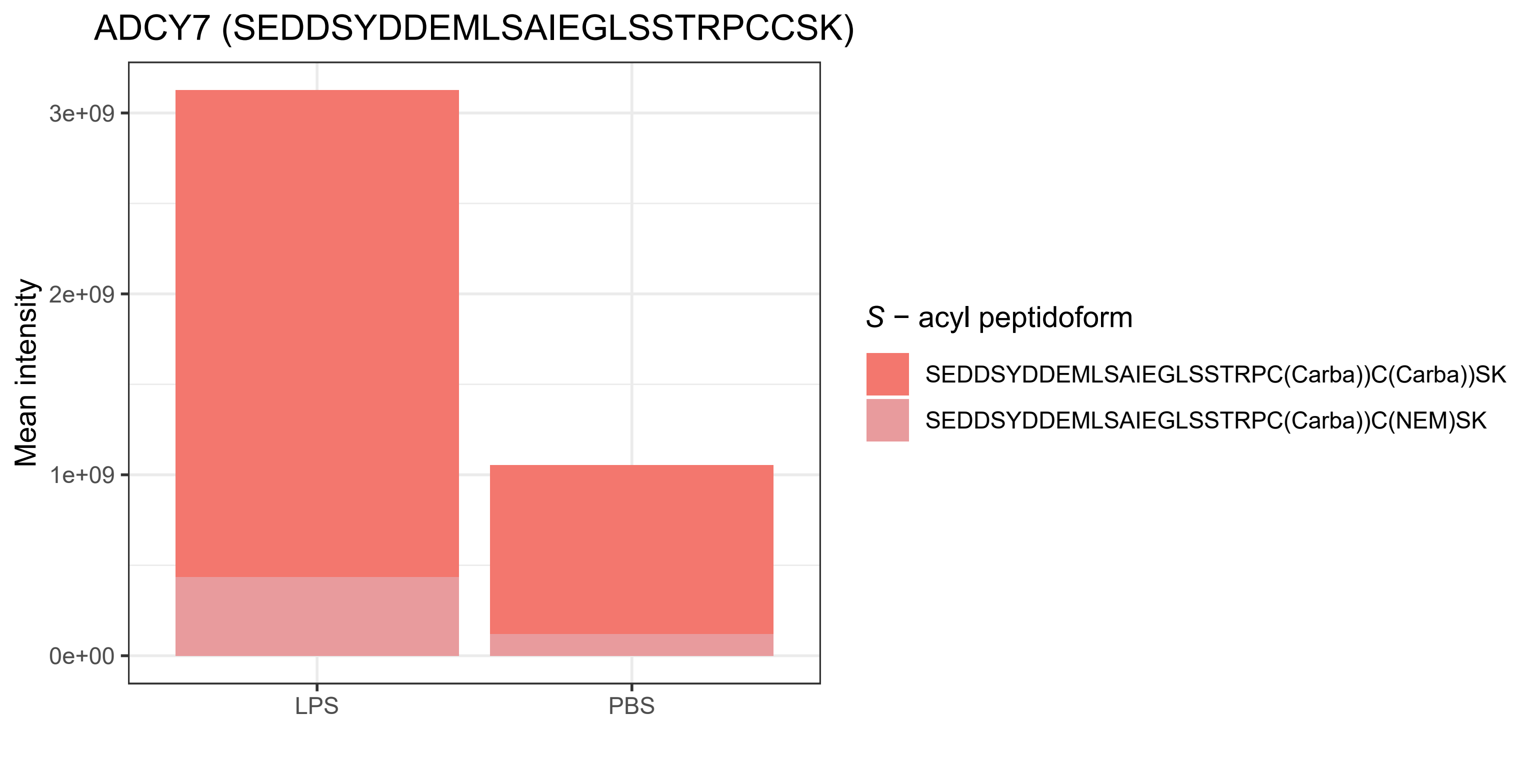
ADCY7 (SEDDSYDDEMLSAIEGLSSTRPCCSK)
Mean intensity
3e+09
2e+09
1e+09
0e+00
LPS
PBS
S − acyl peptidoform
SEDDSYDDEMLSAIEGLSSTRPC(Carba))C(Carba))SK
SEDDSYDDEMLSAIEGLSSTRPC(Carba))C(NEM)SK

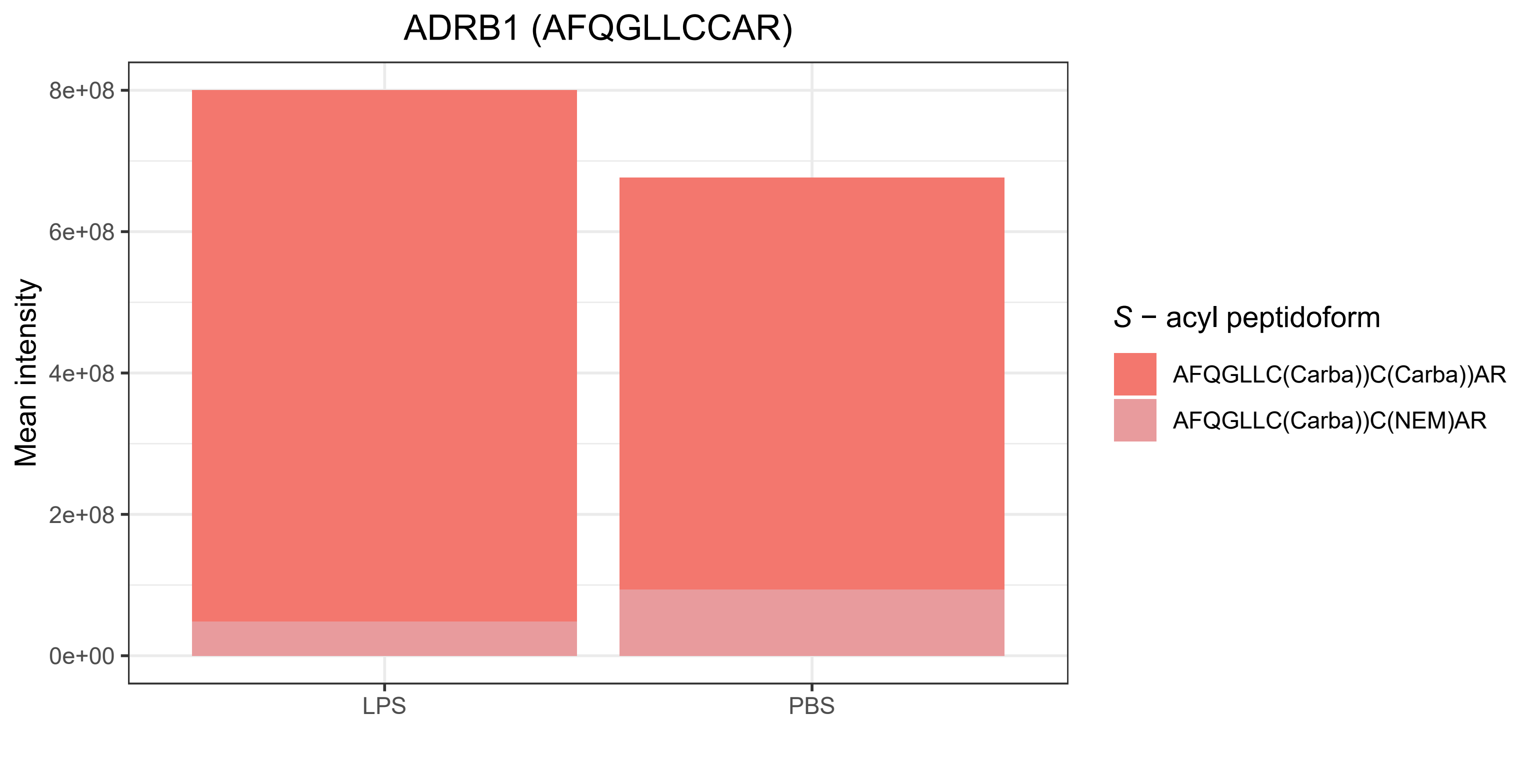
ADRB1 (AFQGLLCCAR)
Mean intensity
8e+08
6e+08
4e+08
2e+08
0e+00
LPS
PBS
S − acyl peptidoform
AFQGLLC(Carba))C(Carba))AR
AFQGLLC(Carba))C(NEM)AR

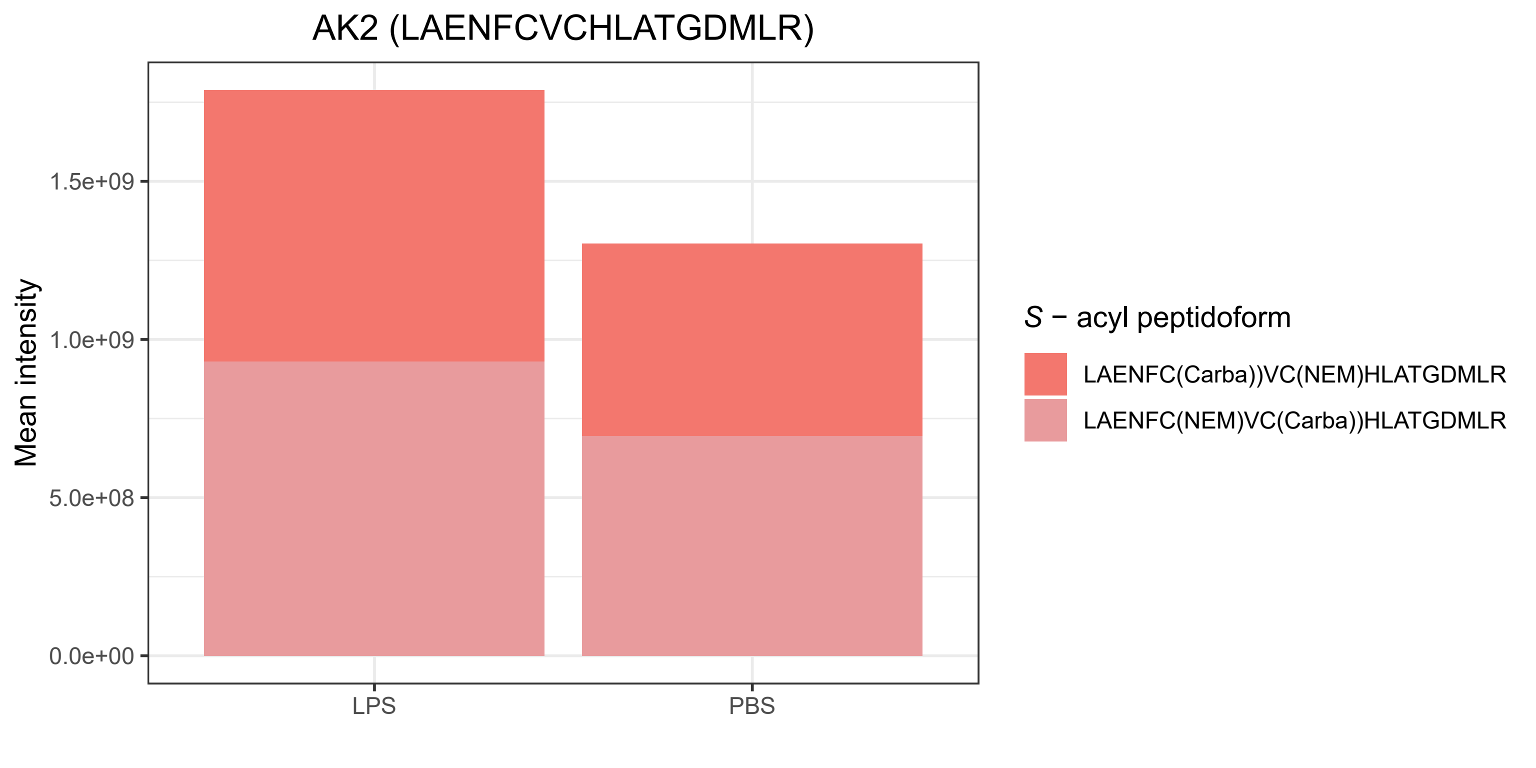

AK2 (LAENFCVCHLATGDMLR)
Mean intensity
1.5e+09
1.0e+09
5.0e+08
0.0e+00
LPS
PBS
S − acyl peptidoform
LAENFC(Carba))VC(NEM)HLATGDMLR
LAENFC(NEM)VC(Carba))HLATGDMLR

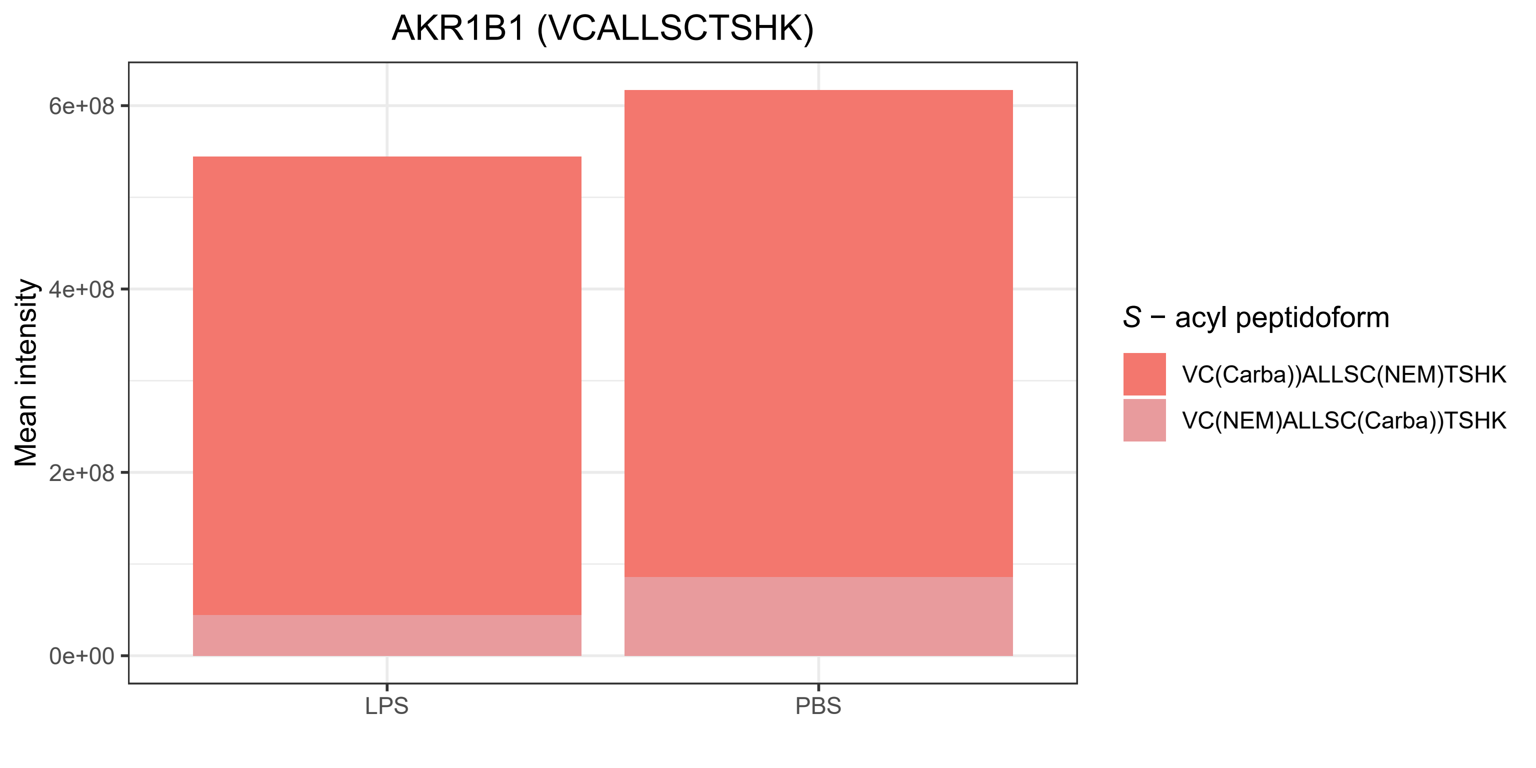

AKR1B1 (VCALLSCTSHK)
Mean intensity
6e+08
4e+08
2e+08
0e+00
LPS
PBS
S − acyl peptidoform
VC(Carba))ALLSC(NEM)TSHK
VC(NEM)ALLSC(Carba))TSHK

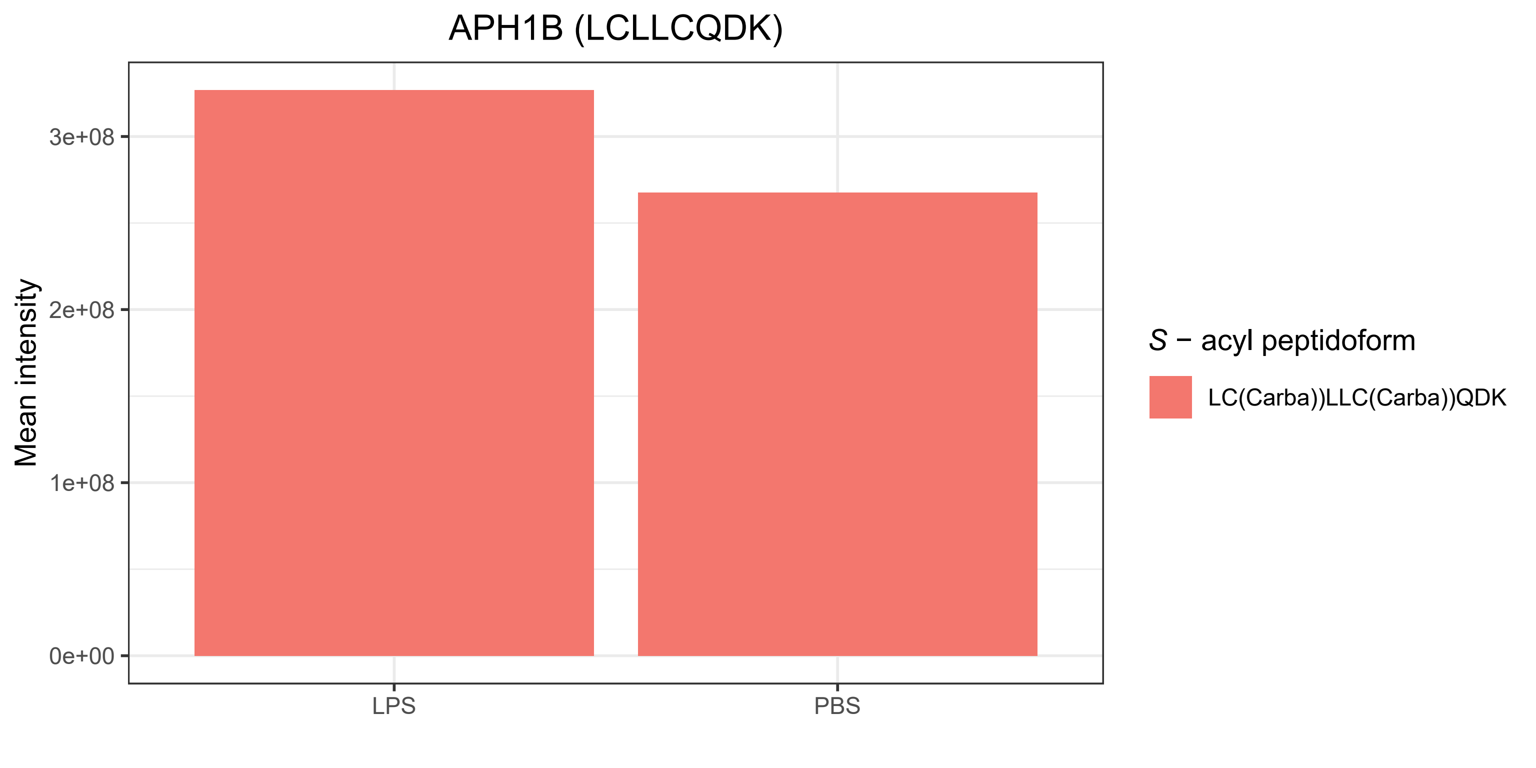
APH1B (LCLLCQDK)
Mean intensity
3e+08
2e+08
1e+08
0e+00
LPS
PBS
S − acyl peptidoform
LC(Carba))LLC(Carba))QDK

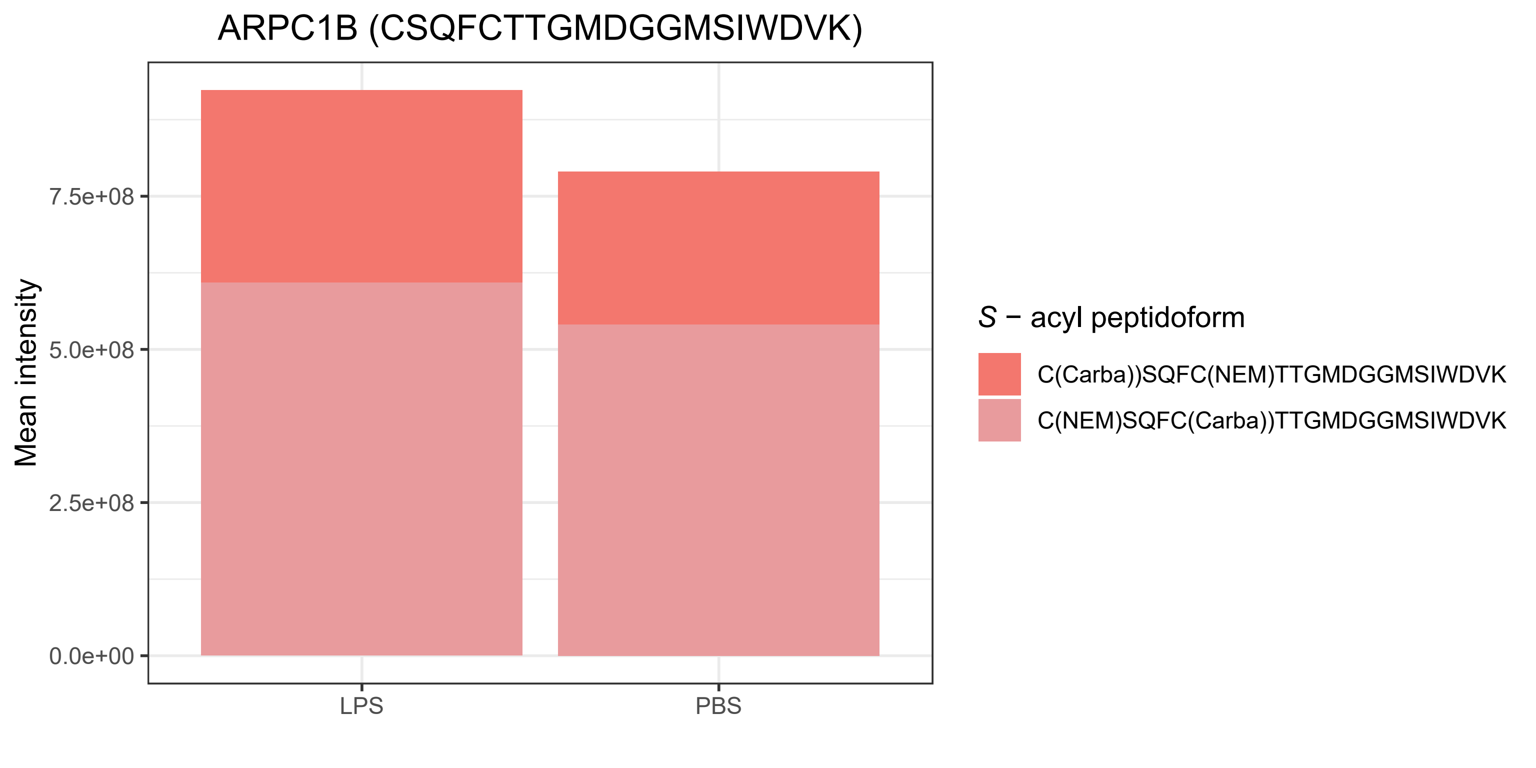
ARPC1B (CSQFCTTGMDGGMSIWDVK)
Mean intensity
7.5e+08
5.0e+08
2.5e+08
0.0e+00
LPS
PBS
S − acyl peptidoform
C(Carba))SQFC(NEM)TTGMDGGMSIWDVK
C(NEM)SQFC(Carba))TTGMDGGMSIWDVK

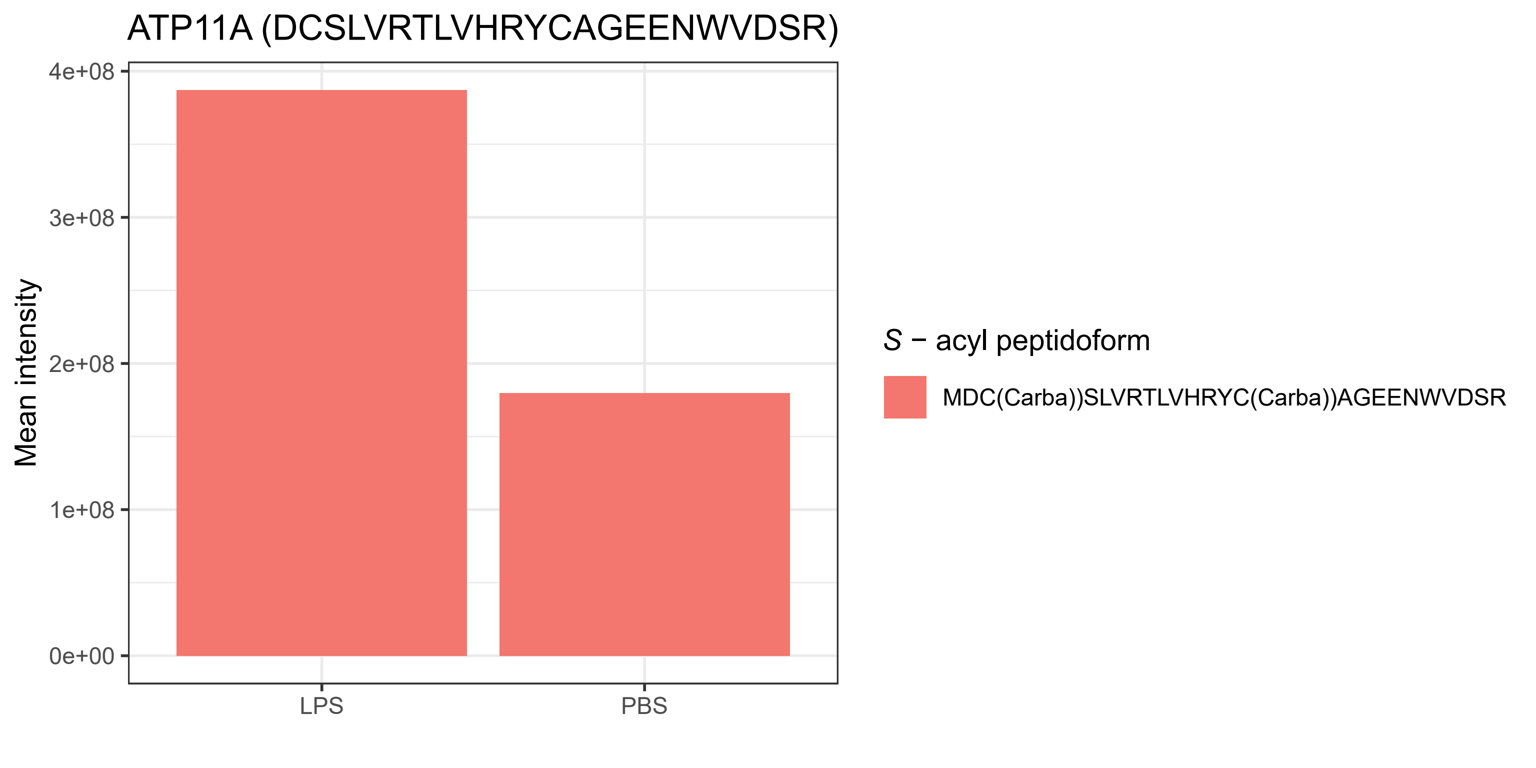
ATP11A (DCSLVRTLVHRYCAGEENWVDSR)
Mean intensity
4e+08
3e+08
2e+08
1e+08
0e+00
LPS
PBS
S − acyl peptidoform
MDC(Carba))SLVRTLVHRYC(Carba))AGEENWVDSR

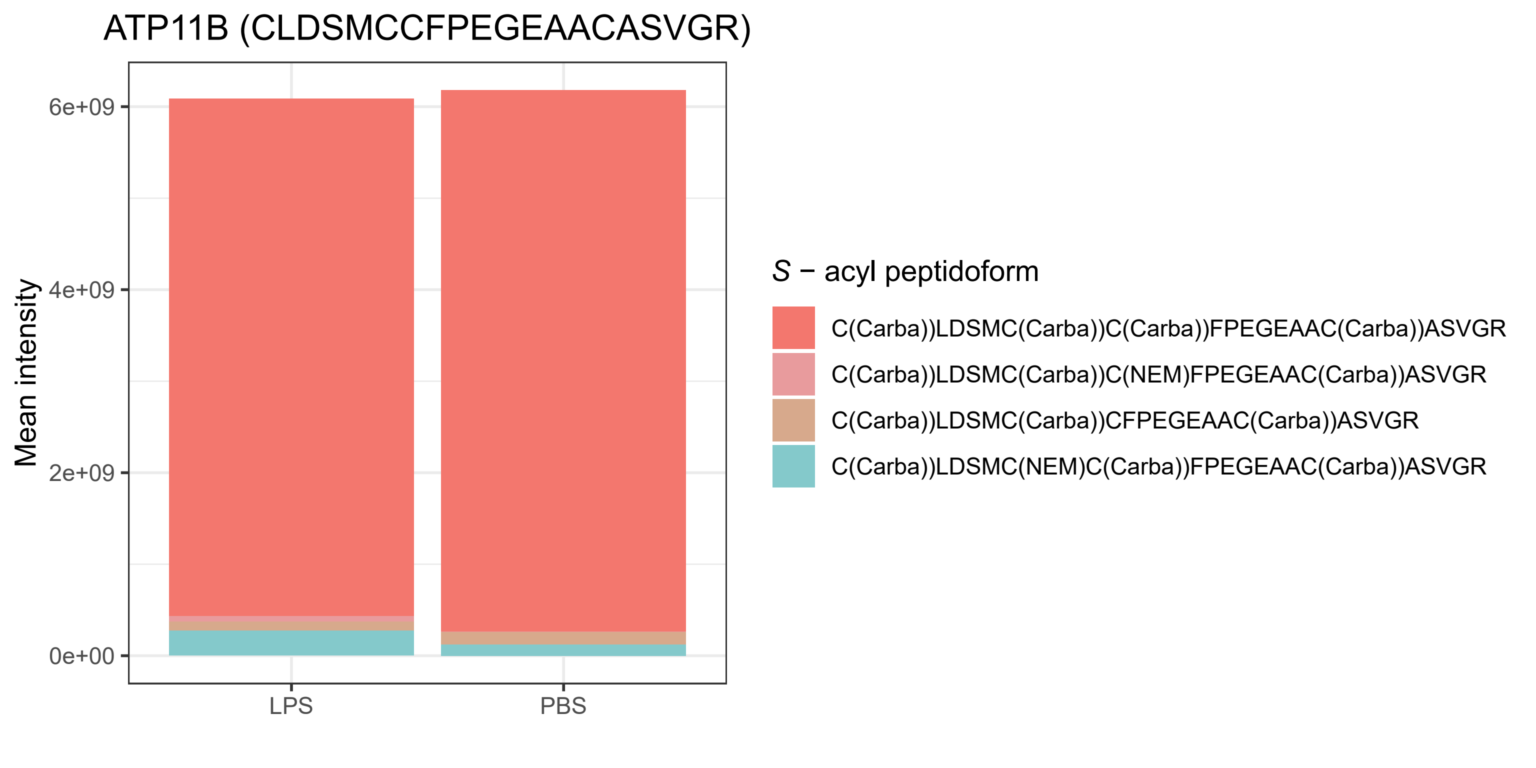
ATP11B (CLDSMCCFPEGEAACASVGR)
Mean intensity
6e+09
4e+09
2e+09
0e+00
LPS
PBS
S − acyl peptidoform
C(Carba))LDSMC(Carba))C(Carba))FPEGEAAC(Carba))ASVGR
C(Carba))LDSMC(Carba))C(NEM)FPEGEAAC(Carba))ASVGR
C(Carba))LDSMC(Carba))CFPEGEAAC(Carba))ASVGR
C(Carba))LDSMC(NEM)C(Carba))FPEGEAAC(Carba))ASVGR

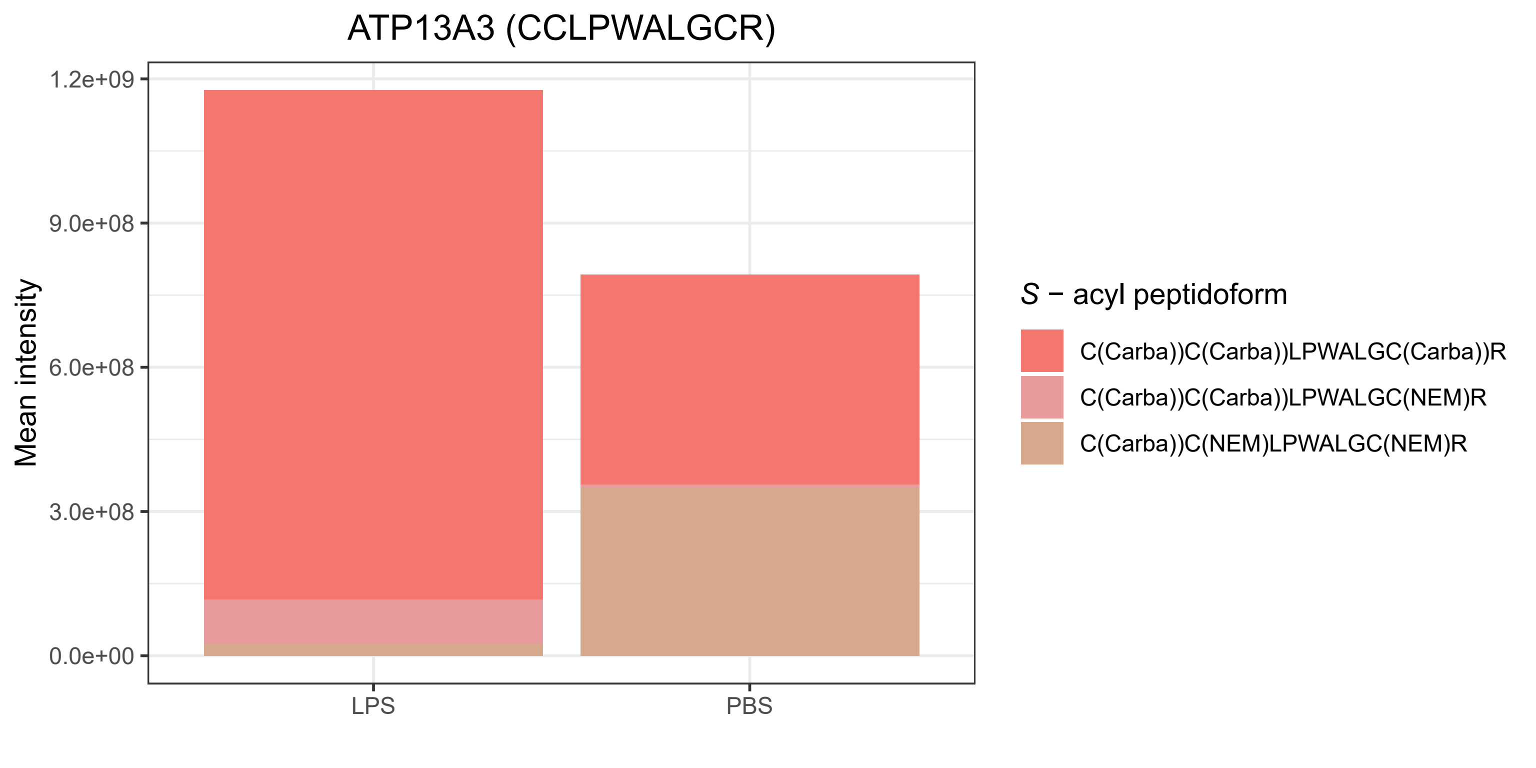
ATP13A3 (CCLPWALGCR)
Mean intensity
1.2e+09
9.0e+08
6.0e+08
3.0e+08
0.0e+00
LPS
PBS
S − acyl peptidoform
C(Carba))C(Carba))LPWALGC(Carba))R
C(Carba))C(Carba))LPWALGC(NEM)R
C(Carba))C(NEM)LPWALGC(NEM)R

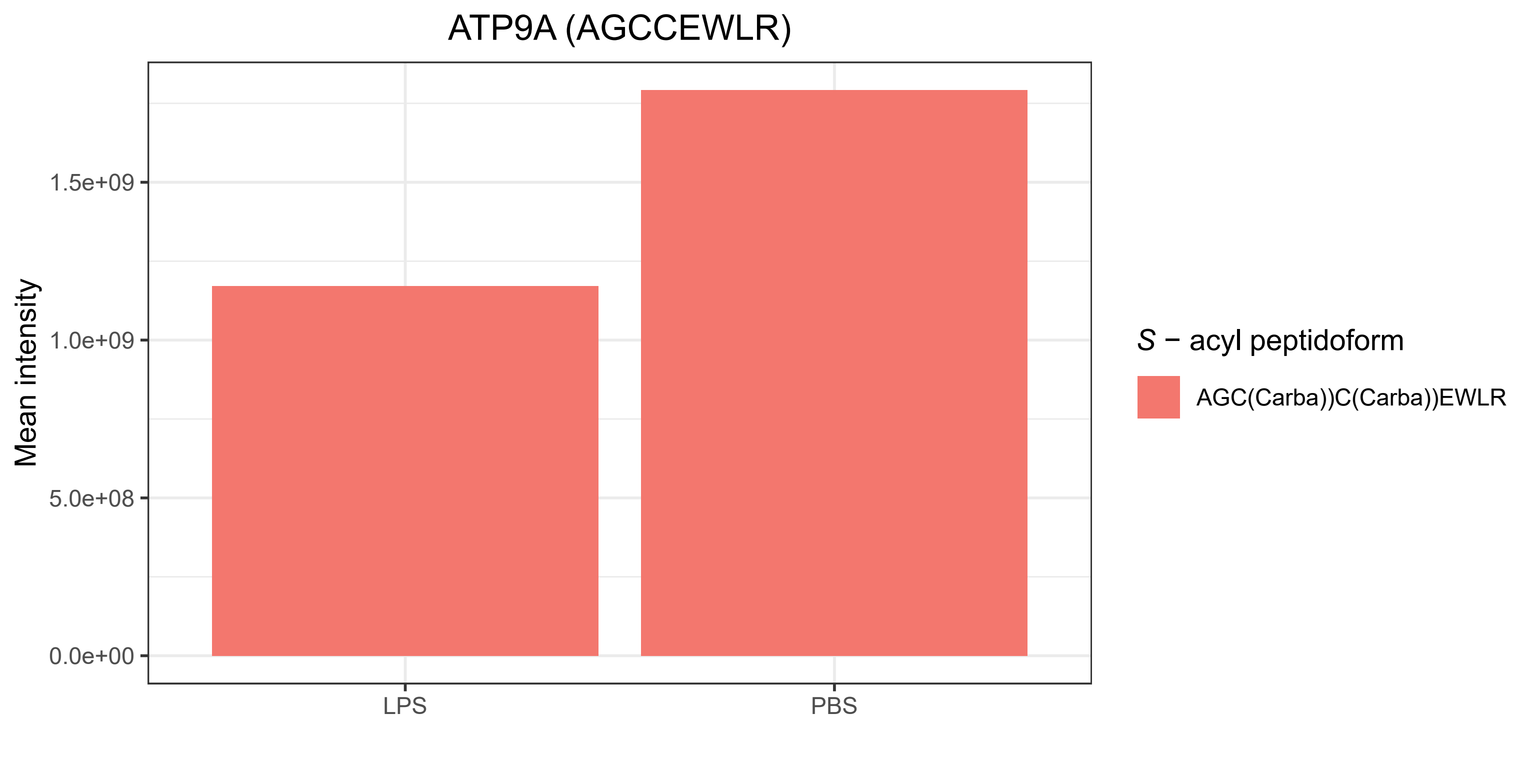

ATP9A (AGCCEWLR)
Mean intensity
1.5e+09
1.0e+09
5.0e+08
0.0e+00
LPS
PBS
S − acyl peptidoform
AGC(Carba))C(Carba))EWLR

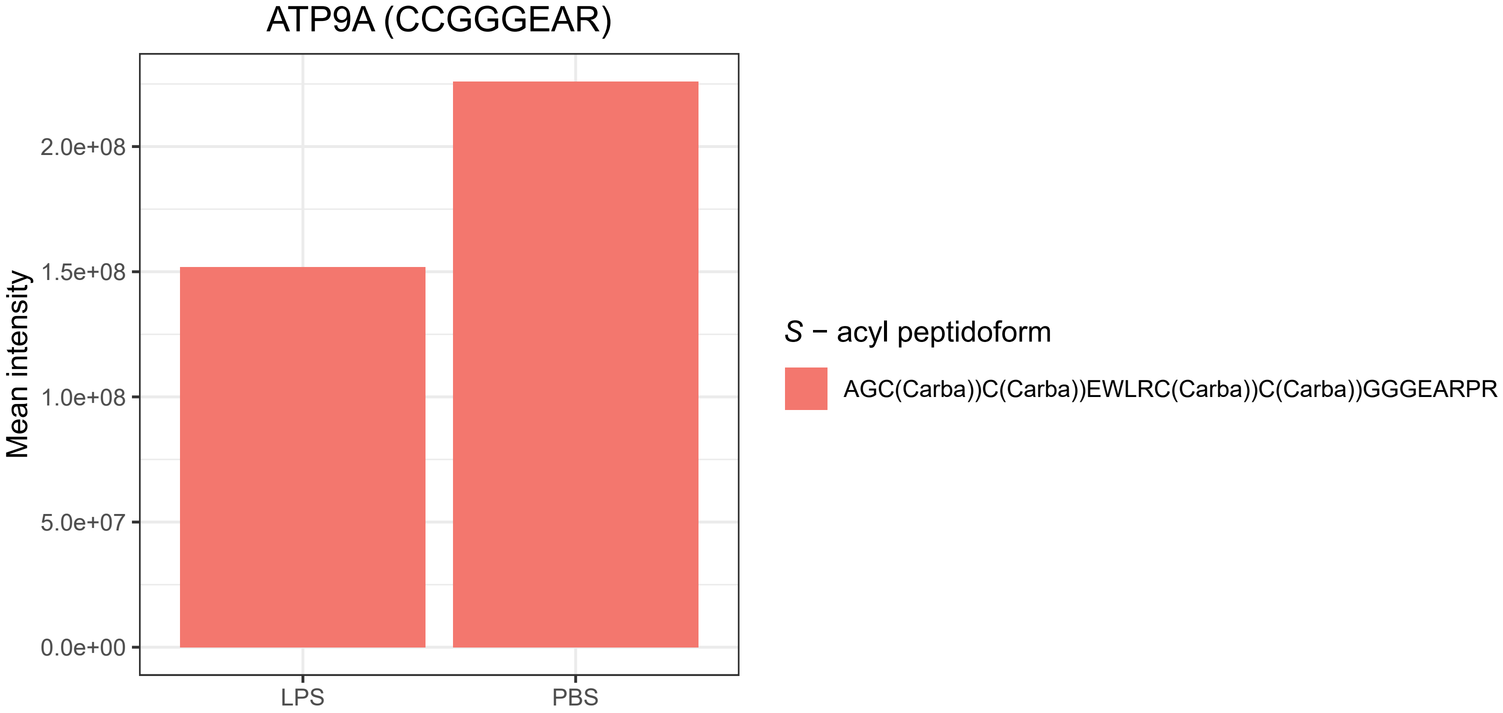
ATP9A (CCGGGEAR)
Mean intensity
2.0e+08
1.5e+08
1.0e+08
5.0e+07
0.0e+00
LPS
PBS
S − acyl peptidoform
AGC(Carba))C(Carba))EWLRC(Carba))C(Carba))GGGEARPR

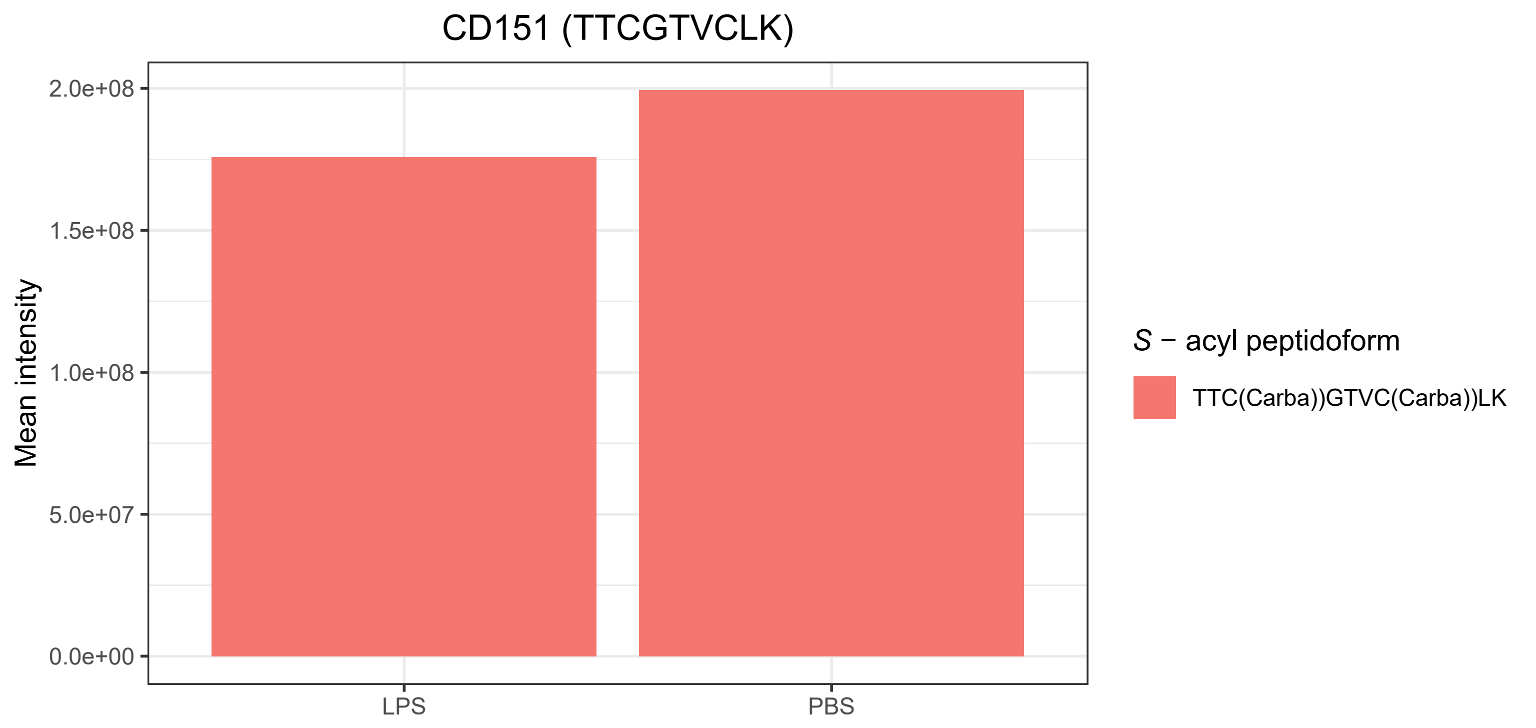
CD151 (TTCGTVCLK)
Mean intensity
2.0e+08
1.5e+08
1.0e+08
5.0e+07
0.0e+00
LPS
PBS
S − acyl peptidoform
TTC(Carba))GTVC(Carba))LK

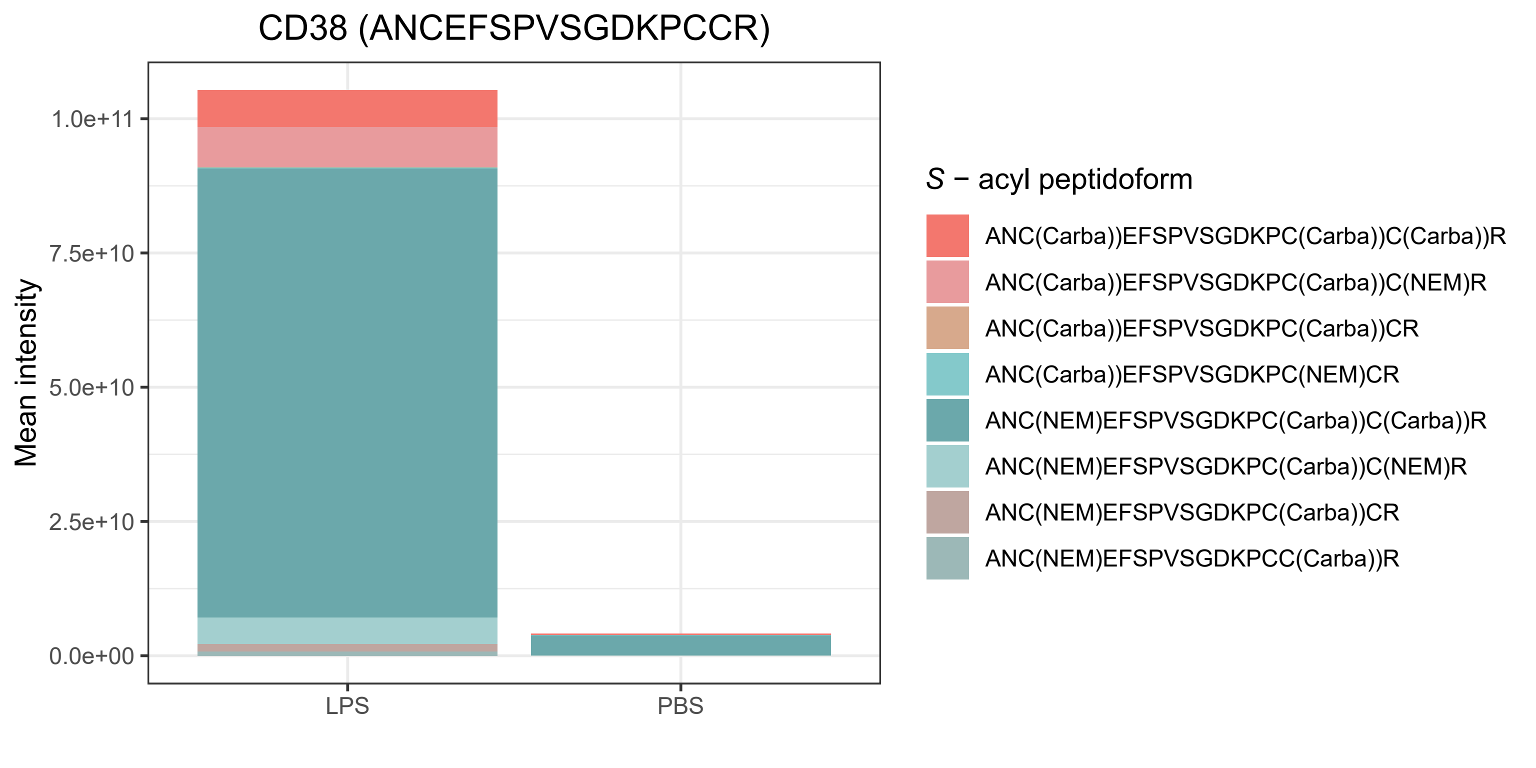

CD38 (ANCEFSPVSGDKPCCR)
Mean intensity
1.0e+11
7.5e+10
5.0e+10
2.5e+10
0.0e+00
LPS
PBS
S − acyl peptidoform
ANC(Carba))EFSPVSGDKPC(Carba))C(Carba))R
ANC(Carba))EFSPVSGDKPC(Carba))C(NEM)R
ANC(Carba))EFSPVSGDKPC(Carba))CR
ANC(Carba))EFSPVSGDKPC(NEM)CR
ANC(NEM)EFSPVSGDKPC(Carba))C(Carba))R
ANC(NEM)EFSPVSGDKPC(Carba))C(NEM)R
ANC(NEM)EFSPVSGDKPC(Carba))CR
ANC(NEM)EFSPVSGDKPCC(Carba))R

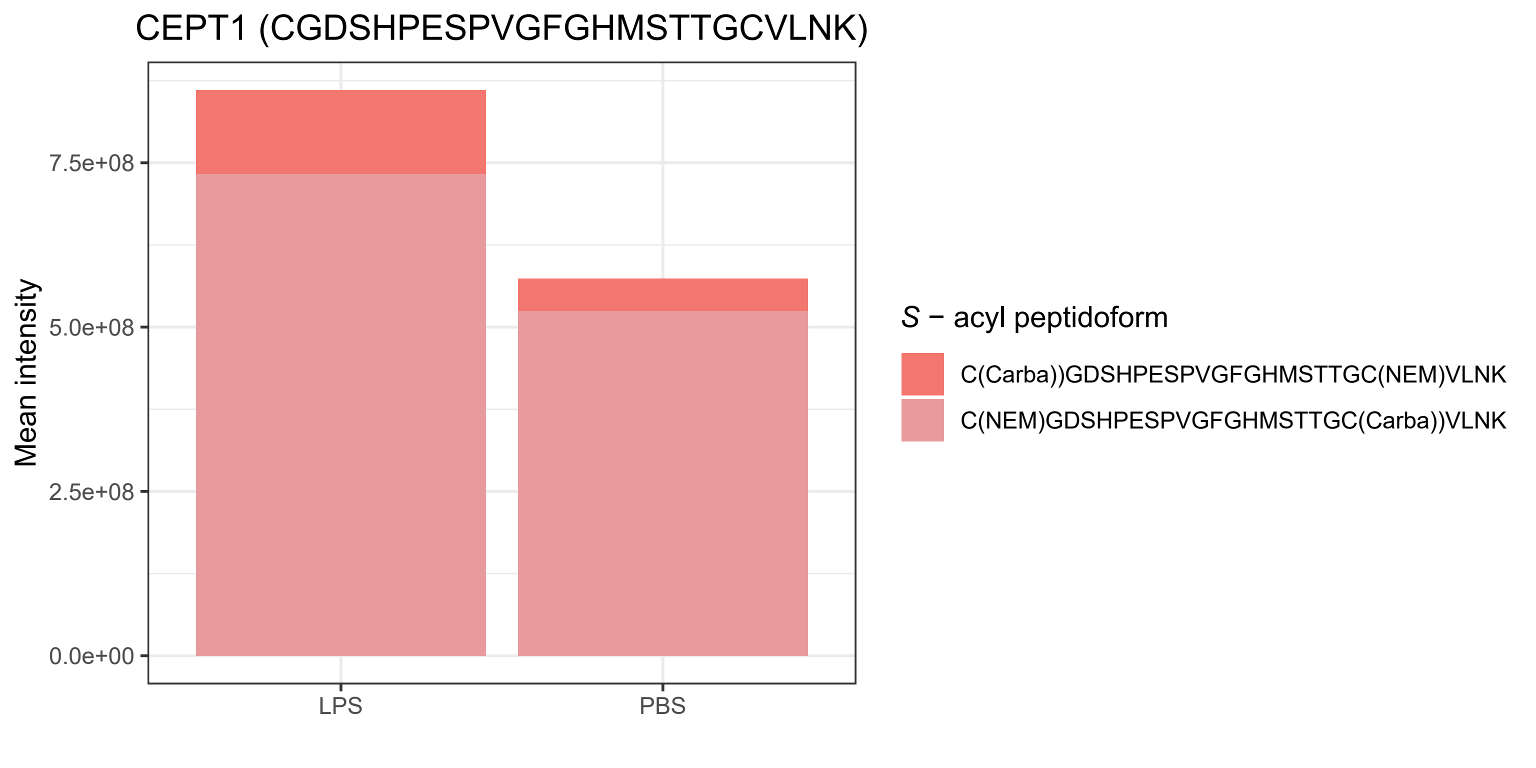

CEPT1 (CGDSHPESPVGFGHMSTTGCVLNK)
Mean intensity
7.5e+08
5.0e+08
2.5e+08
0.0e+00
LPS
PBS
S − acyl peptidoform
C(Carba))GDSHPESPVGFGHMSTTGC(NEM)VLNK
C(NEM)GDSHPESPVGFGHMSTTGC(Carba))VLNK

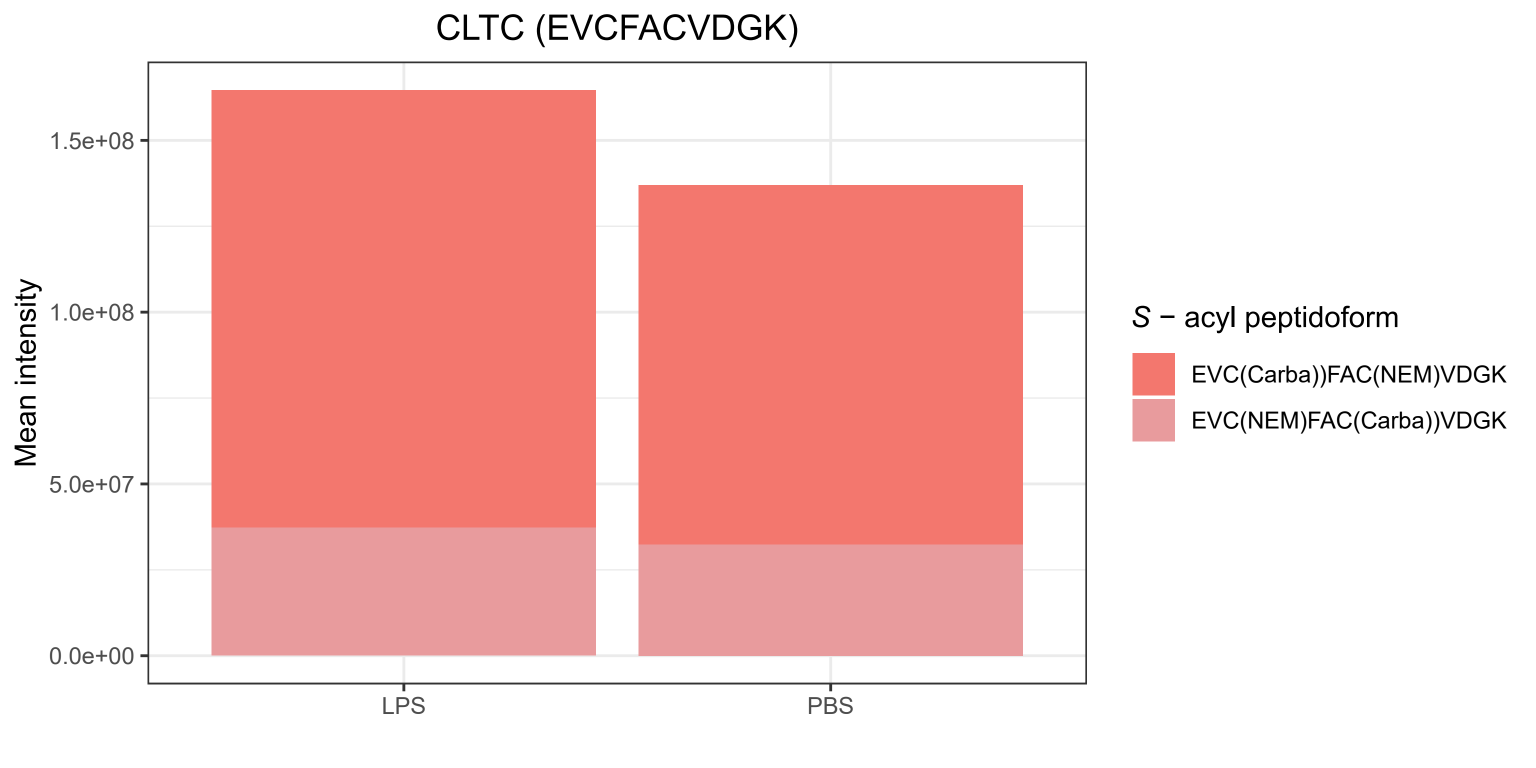

CLTC (EVCFACVDGK)
Mean intensity
1.5e+08
1.0e+08
5.0e+07
0.0e+00
LPS
PBS
S − acyl peptidoform
EVC(Carba))FAC(NEM)VDGK
EVC(NEM)FAC(Carba))VDGK

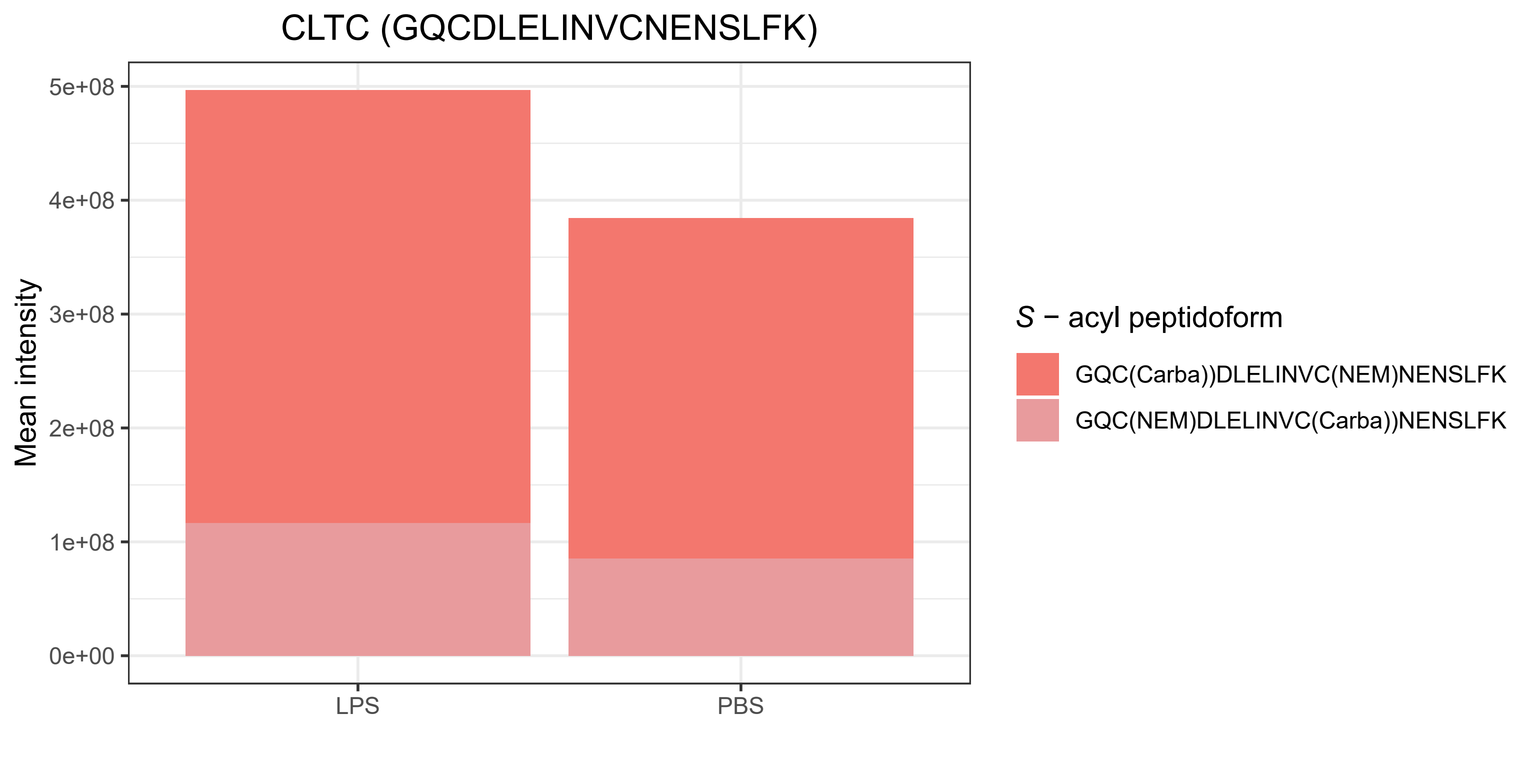

CLTC (GQCDLELINVCNENSLFK)
Mean intensity
5e+08
4e+08
3e+08
2e+08
1e+08
0e+00
LPS
PBS
S − acyl peptidoform
GQC(Carba))DLELINVC(NEM)NENSLFK
GQC(NEM)DLELINVC(Carba))NENSLFK

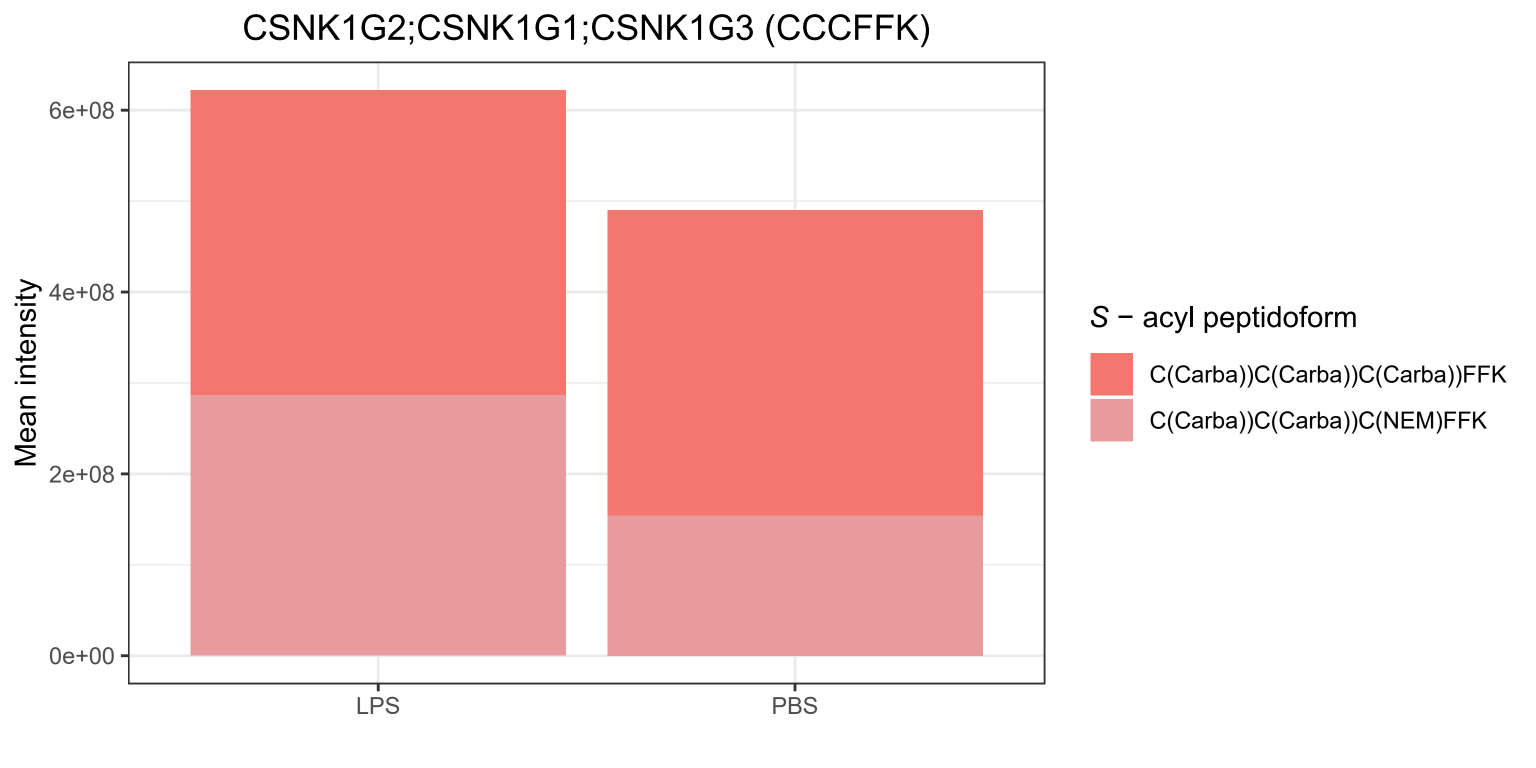
CSNK1G2;CSNK1G1;CSNK1G3 (CCCFFK)
Mean intensity
6e+08
4e+08
2e+08
0e+00
LPS
PBS
S − acyl peptidoform
C(Carba))C(Carba))C(Carba))FFK
C(Carba))C(Carba))C(NEM)FFK

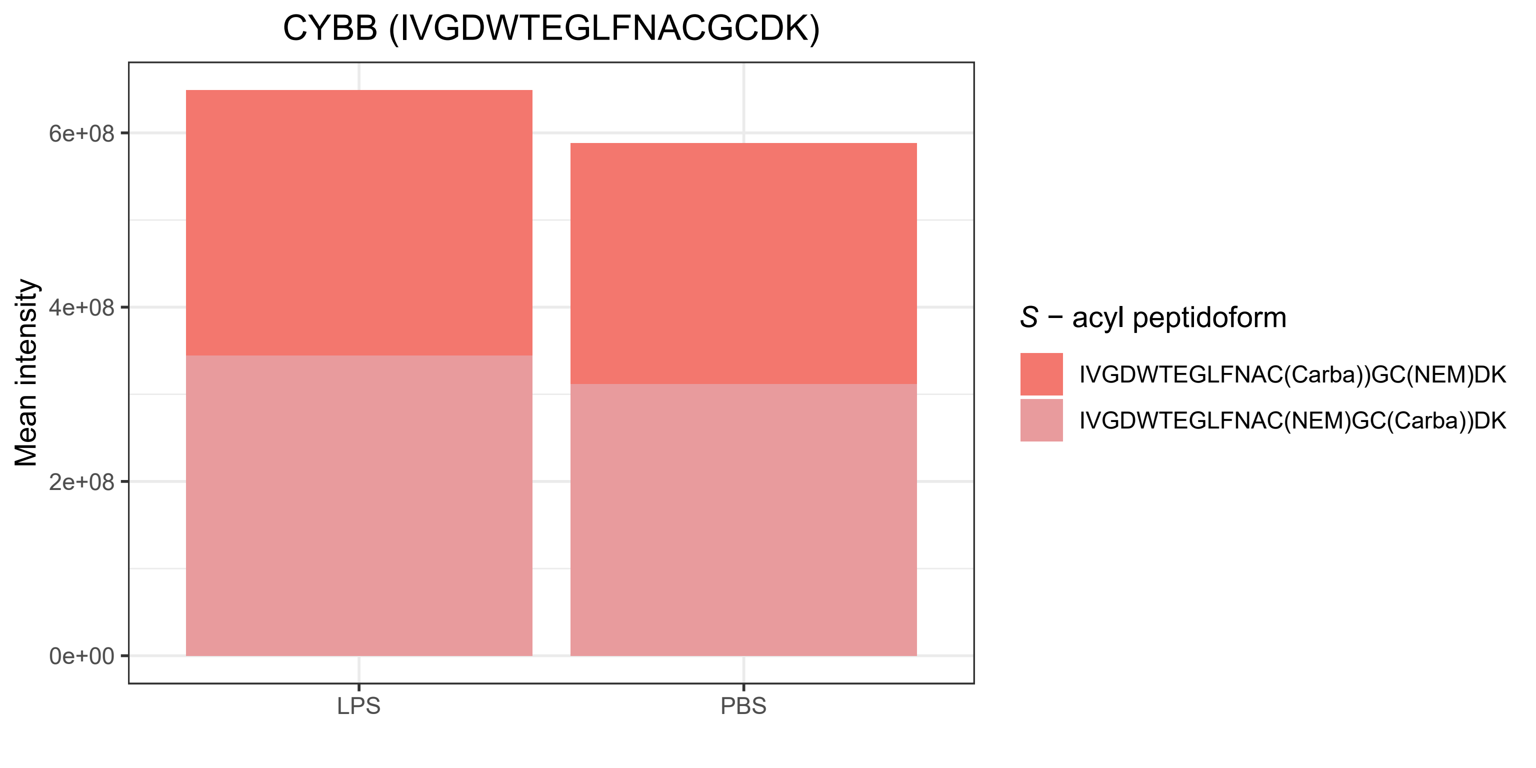
CYBB (IVGDWTEGLFNACGCDK)
Mean intensity
6e+08
4e+08
2e+08
0e+00
LPS
PBS
S − acyl peptidoform
IVGDWTEGLFNAC(Carba))GC(NEM)DK
IVGDWTEGLFNAC(NEM)GC(Carba))DK

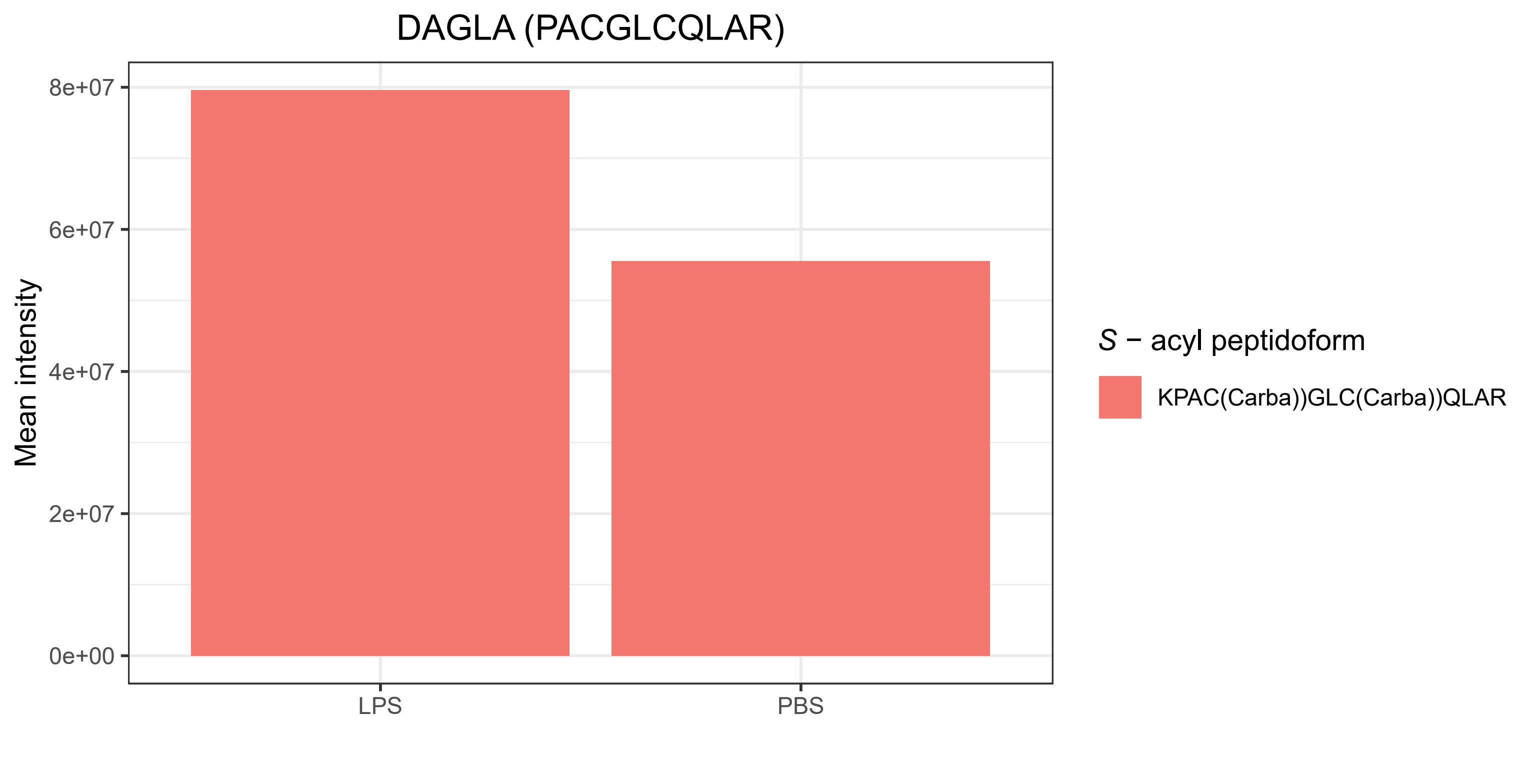
DAGLA (PACGLCQLAR)
Mean intensity
8e+07
6e+07
4e+07
2e+07
0e+00
LPS
PBS
S − acyl peptidoform
KPAC(Carba))GLC(Carba))QLAR

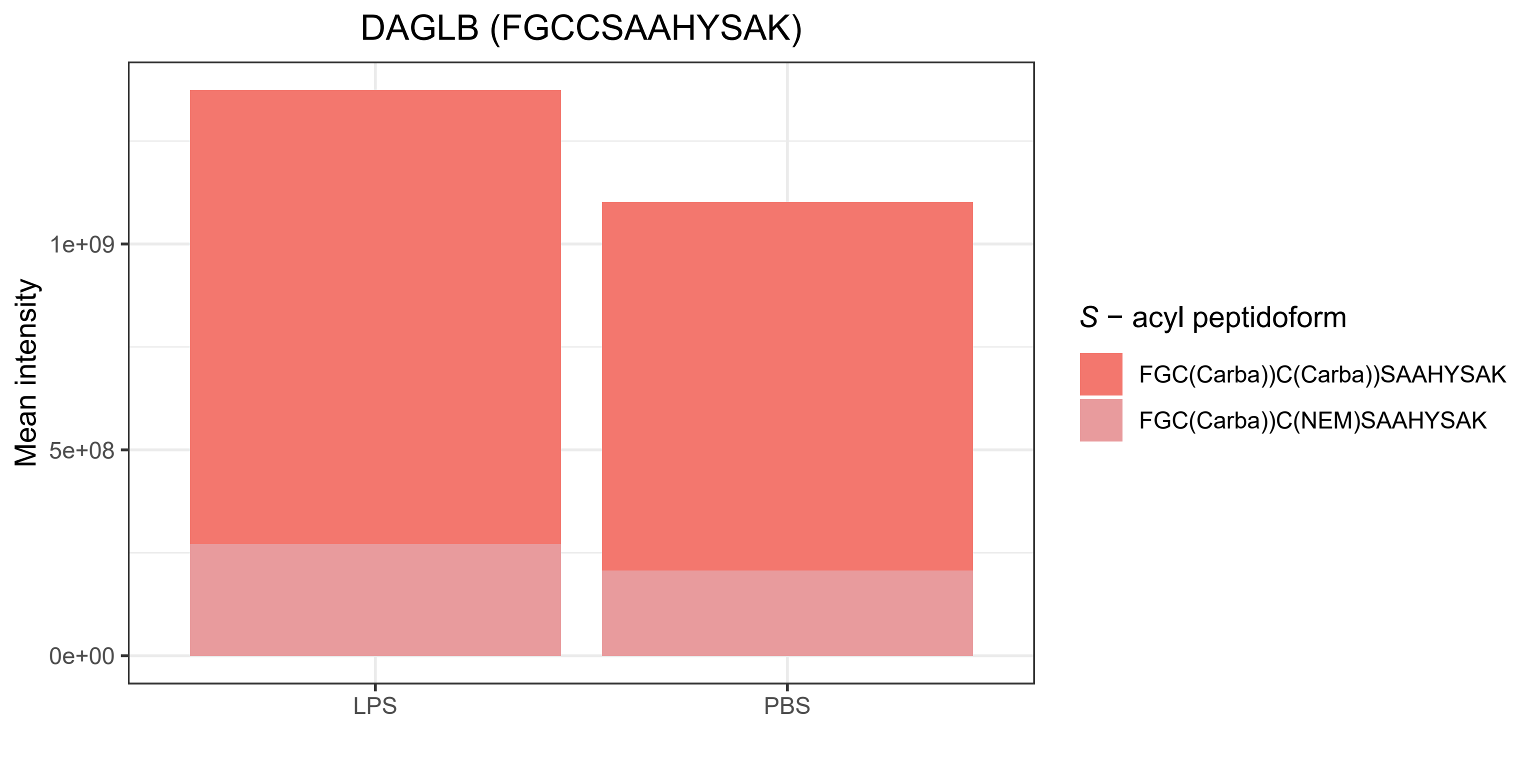
DAGLB (FGCCSAAHYSAK)
Mean intensity
1e+09
5e+08
0e+00
LPS
PBS
S − acyl peptidoform
FGC(Carba))C(Carba))SAAHYSAK
FGC(Carba))C(NEM)SAAHYSAK

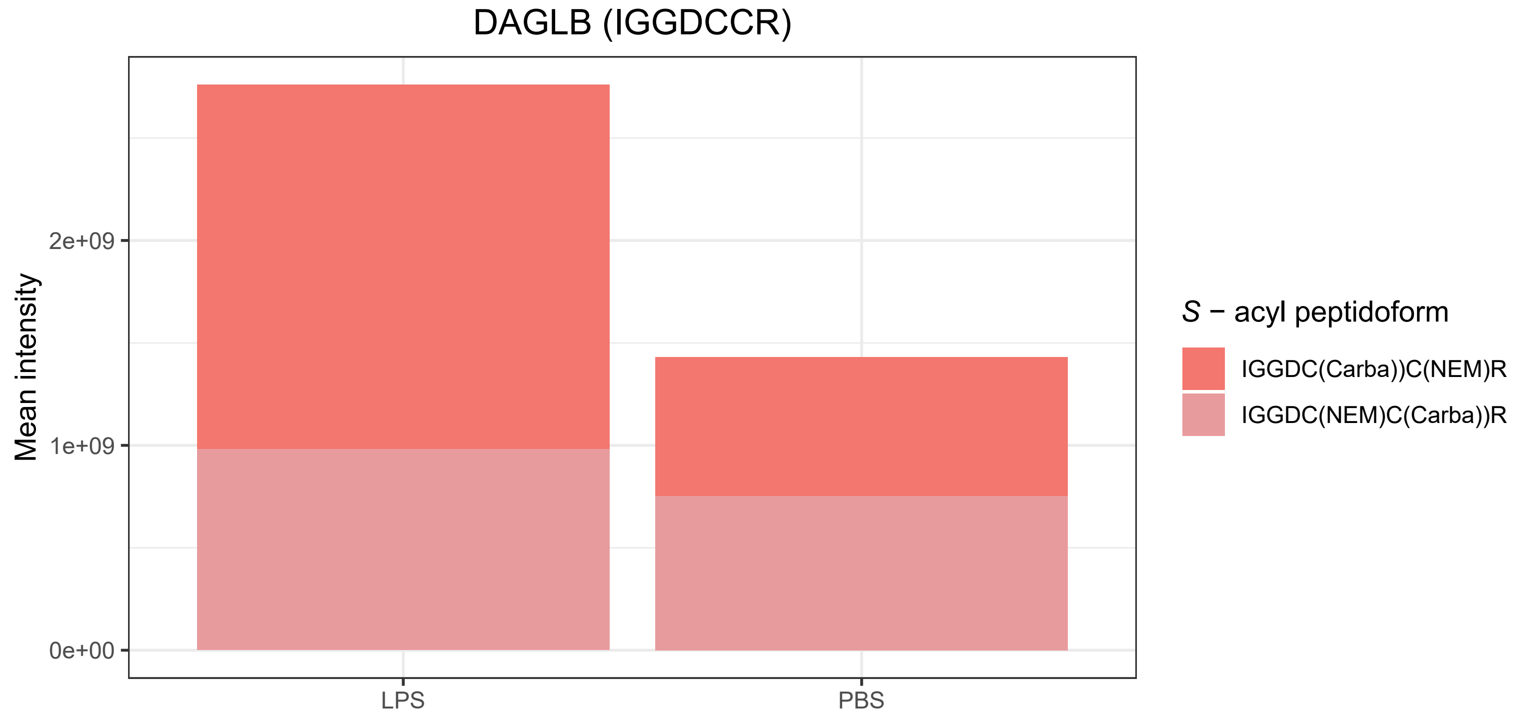
DAGLB (IGGDCCR)
Mean intensity
2e+09
1e+09
0e+00
LPS
PBS
S − acyl peptidoform
IGGDC(Carba))C(NEM)R
IGGDC(NEM)C(Carba))R

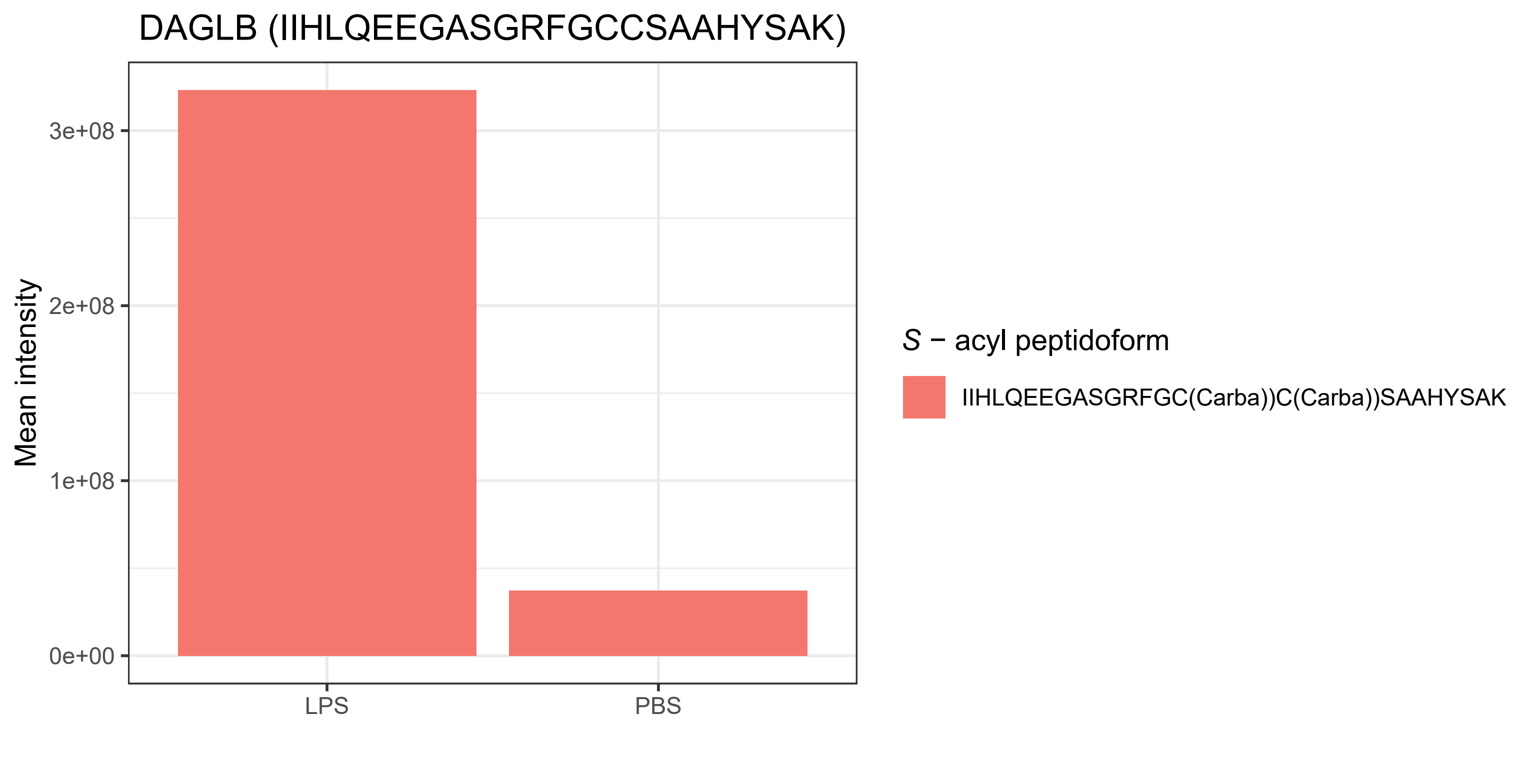
DAGLB (IIHLQEEGASGRFGCCSAAHYSAK)
Mean intensity
3e+08
2e+08
1e+08
0e+00
LPS
PBS
S − acyl peptidoform
IIHLQEEGASGRFGC(Carba))C(Carba))SAAHYSAK

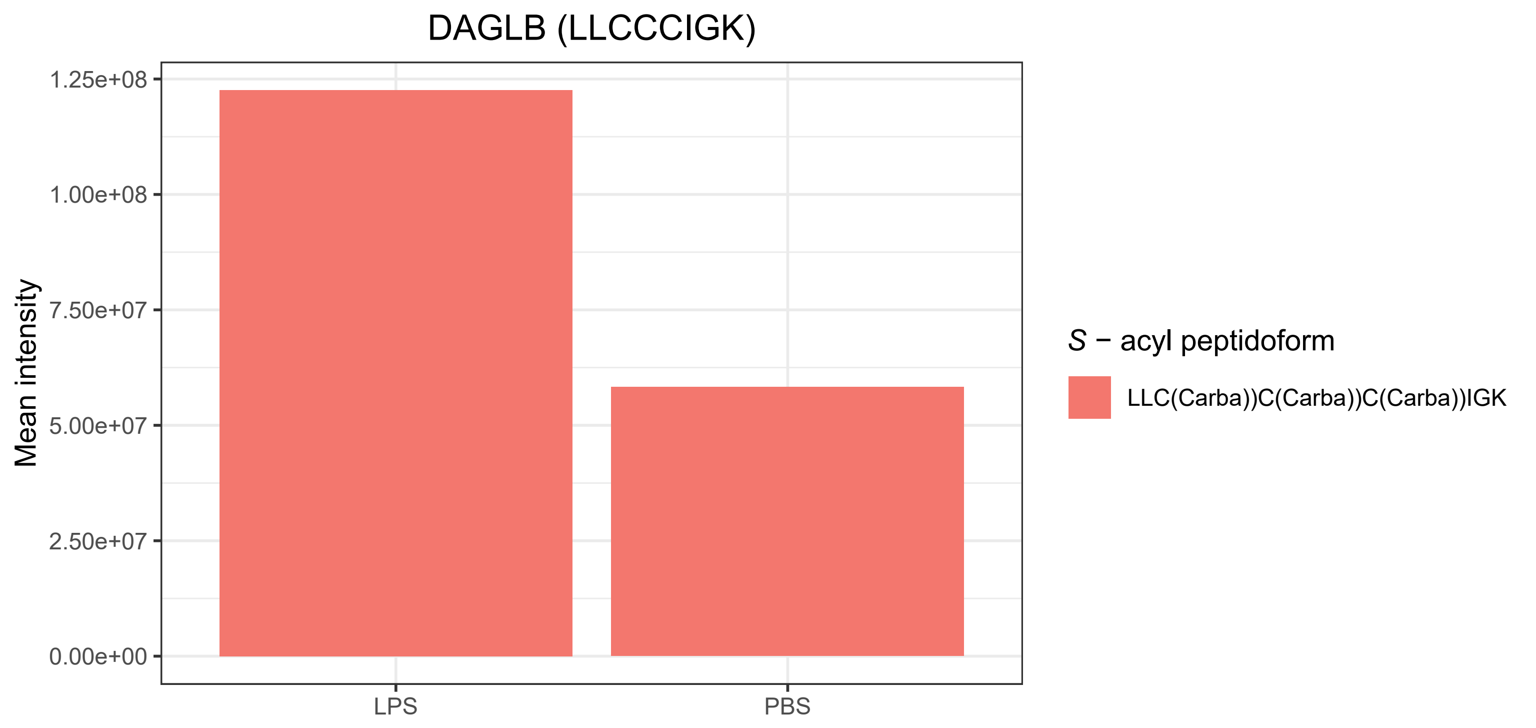
DAGLB (LLCCCIGK)
Mean intensity
1.25e+08
1.00e+08
7.50e+07
5.00e+07
2.50e+07
0.00e+00
LPS
PBS
S − acyl peptidoform
LLC(Carba))C(Carba))C(Carba))IGK

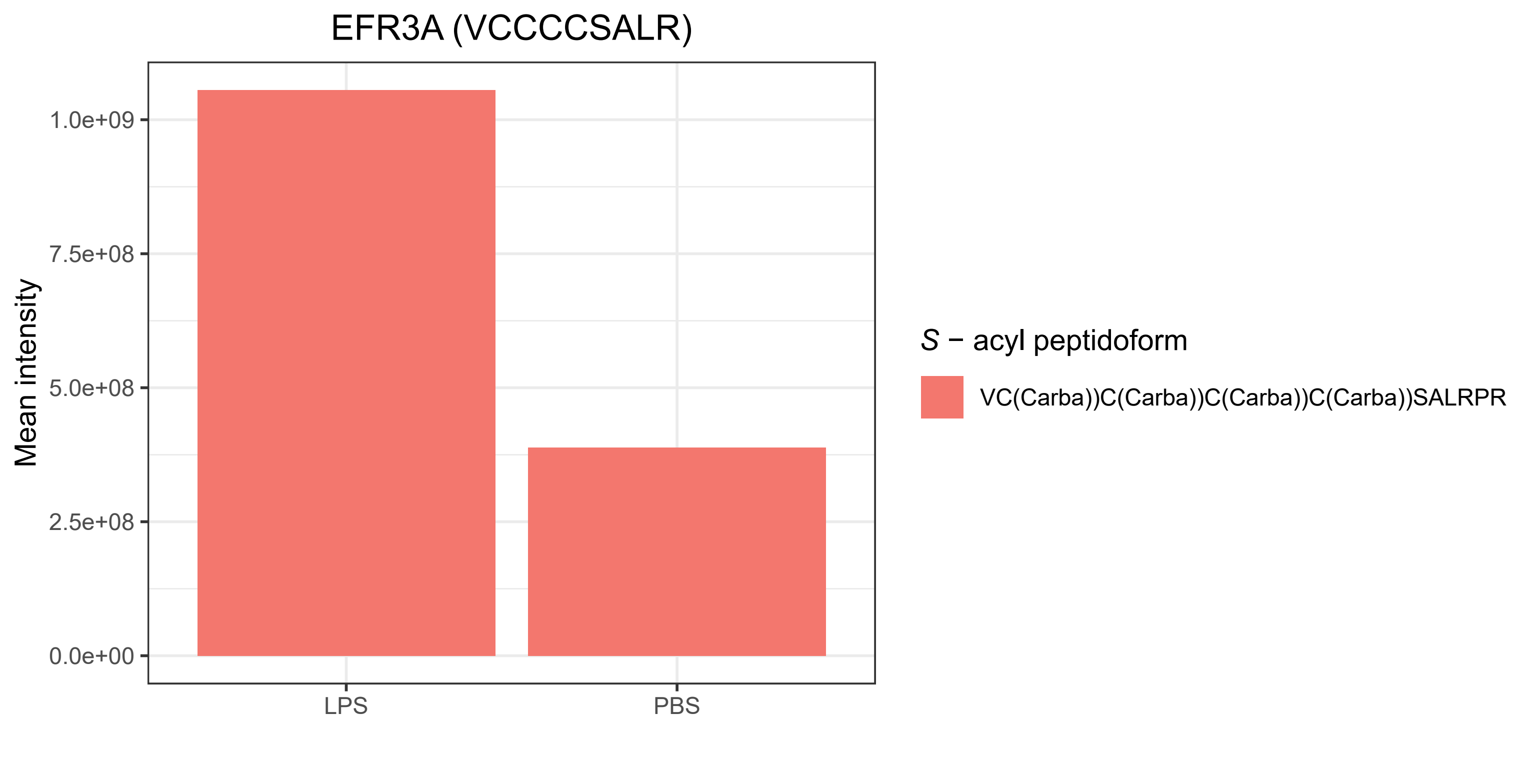

EFR3A (VCCCCSALR)
Mean intensity
1.0e+09
7.5e+08
5.0e+08
2.5e+08
0.0e+00
LPS
PBS
S − acyl peptidoform
VC(Carba))C(Carba))C(Carba))C(Carba))SALRPR

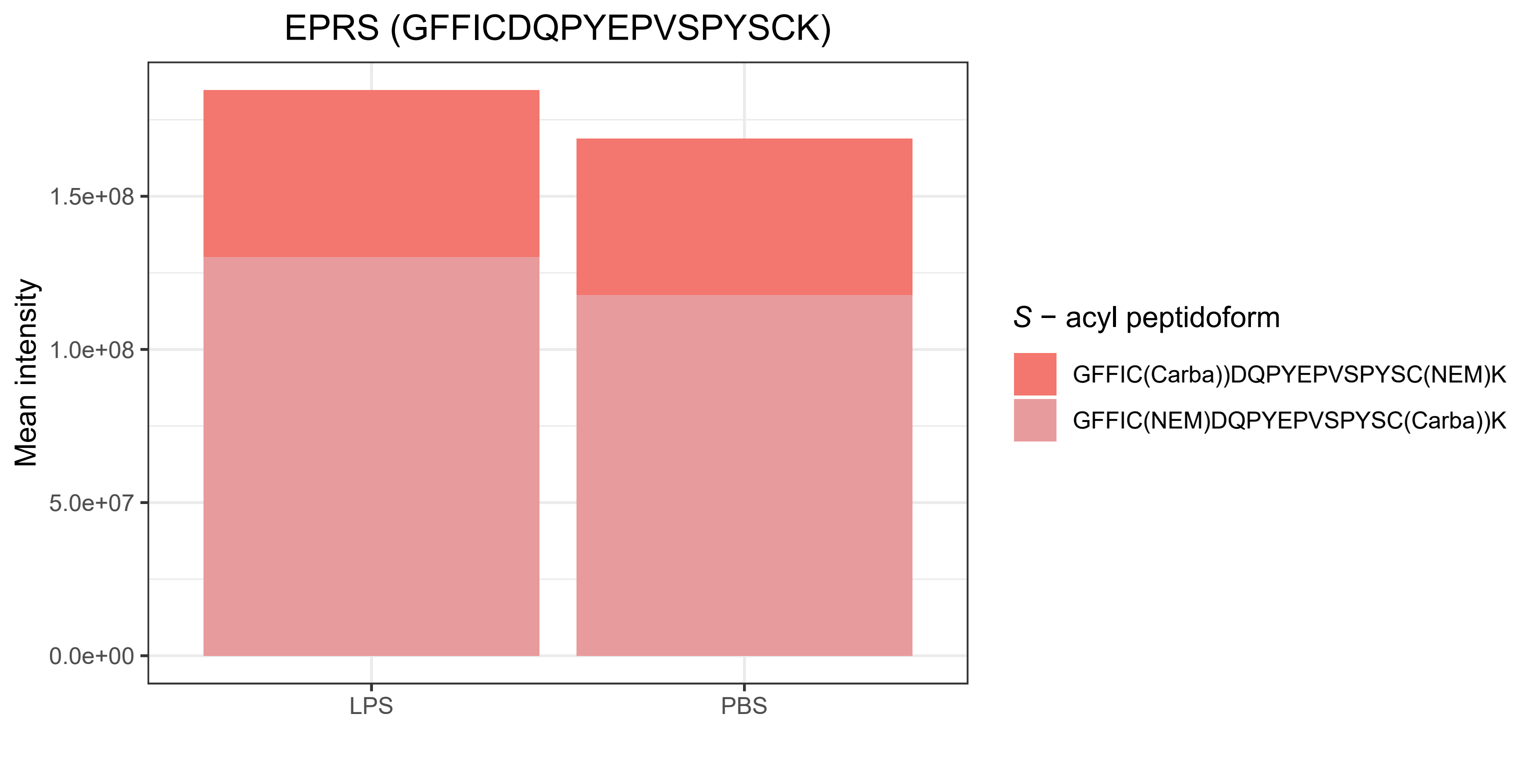

EPRS (GFFICDQPYEPVSPYSCK)
Mean intensity
1.5e+08
1.0e+08
5.0e+07
0.0e+00
LPS
PBS
S − acyl peptidoform
GFFIC(Carba))DQPYEPVSPYSC(NEM)K
GFFIC(NEM)DQPYEPVSPYSC(Carba))K

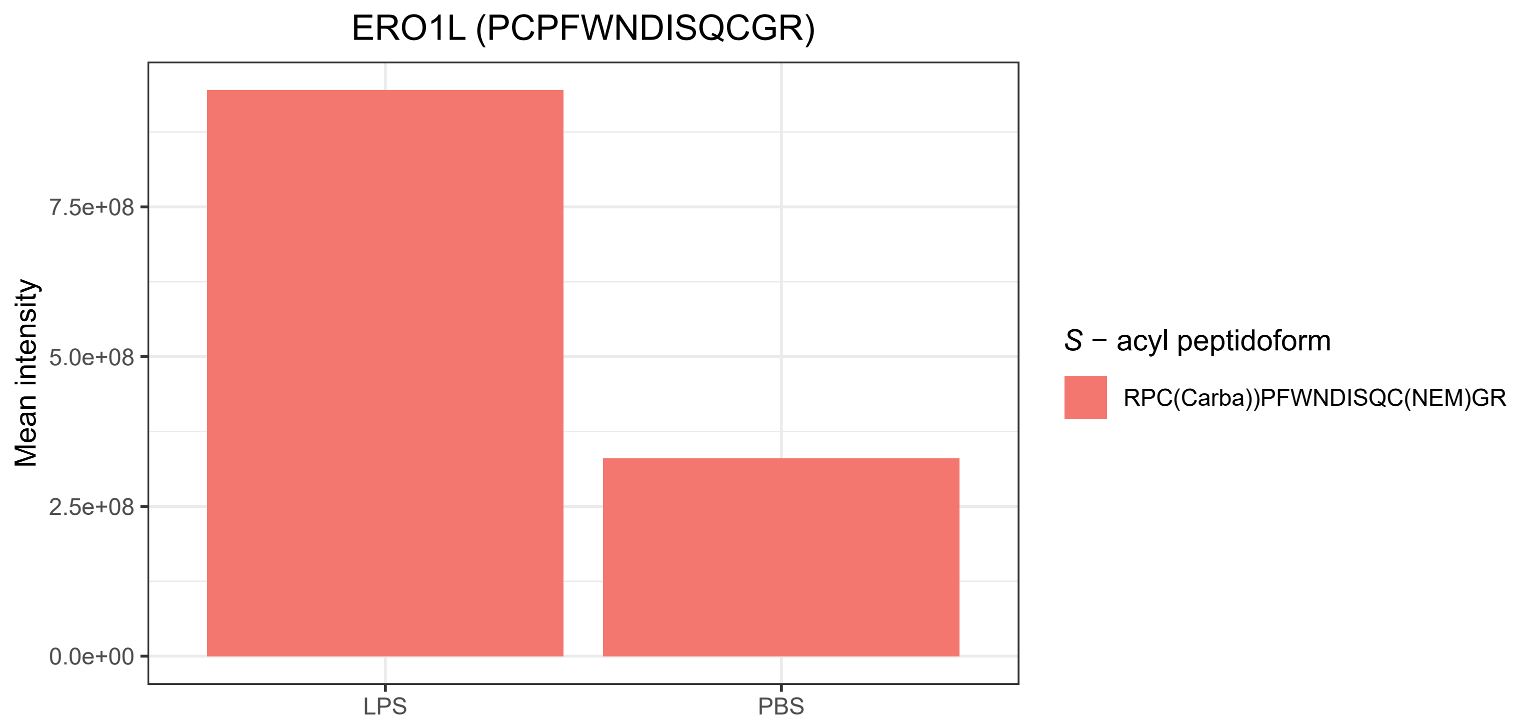

ERO1L (PCPFWNDISQCGR)
Mean intensity
7.5e+08
5.0e+08
2.5e+08
0.0e+00
LPS
PBS
S − acyl peptidoform
RPC(Carba))PFWNDISQC(NEM)GR

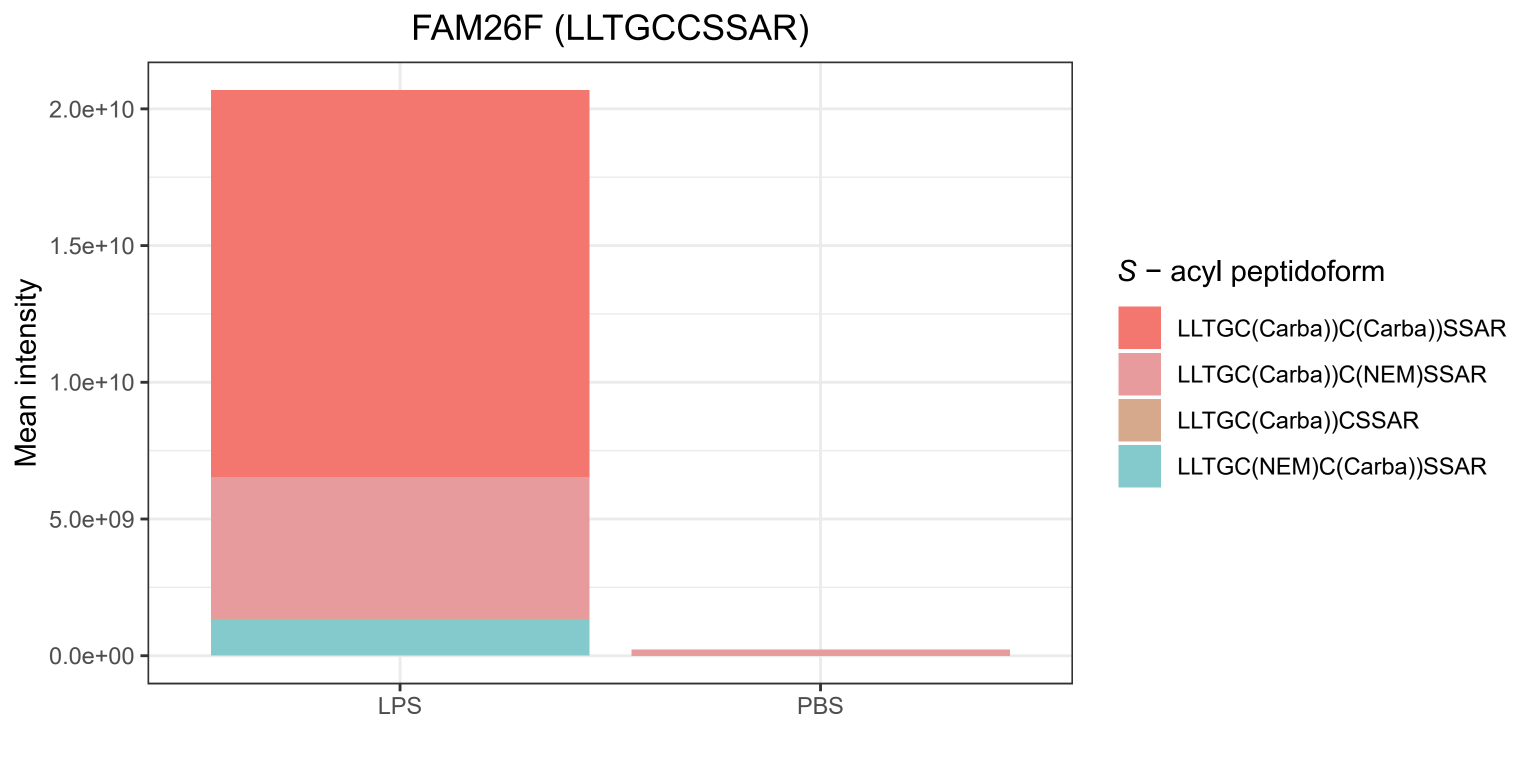

FAM26F (LLTGCCSSAR)
Mean intensity
2.0e+10
1.5e+10
1.0e+10
5.0e+09
0.0e+00
LPS
PBS
S − acyl peptidoform
LLTGC(Carba))C(Carba))SSAR
LLTGC(Carba))C(NEM)SSAR
LLTGC(Carba))CSSAR
LLTGC(NEM)C(Carba))SSAR

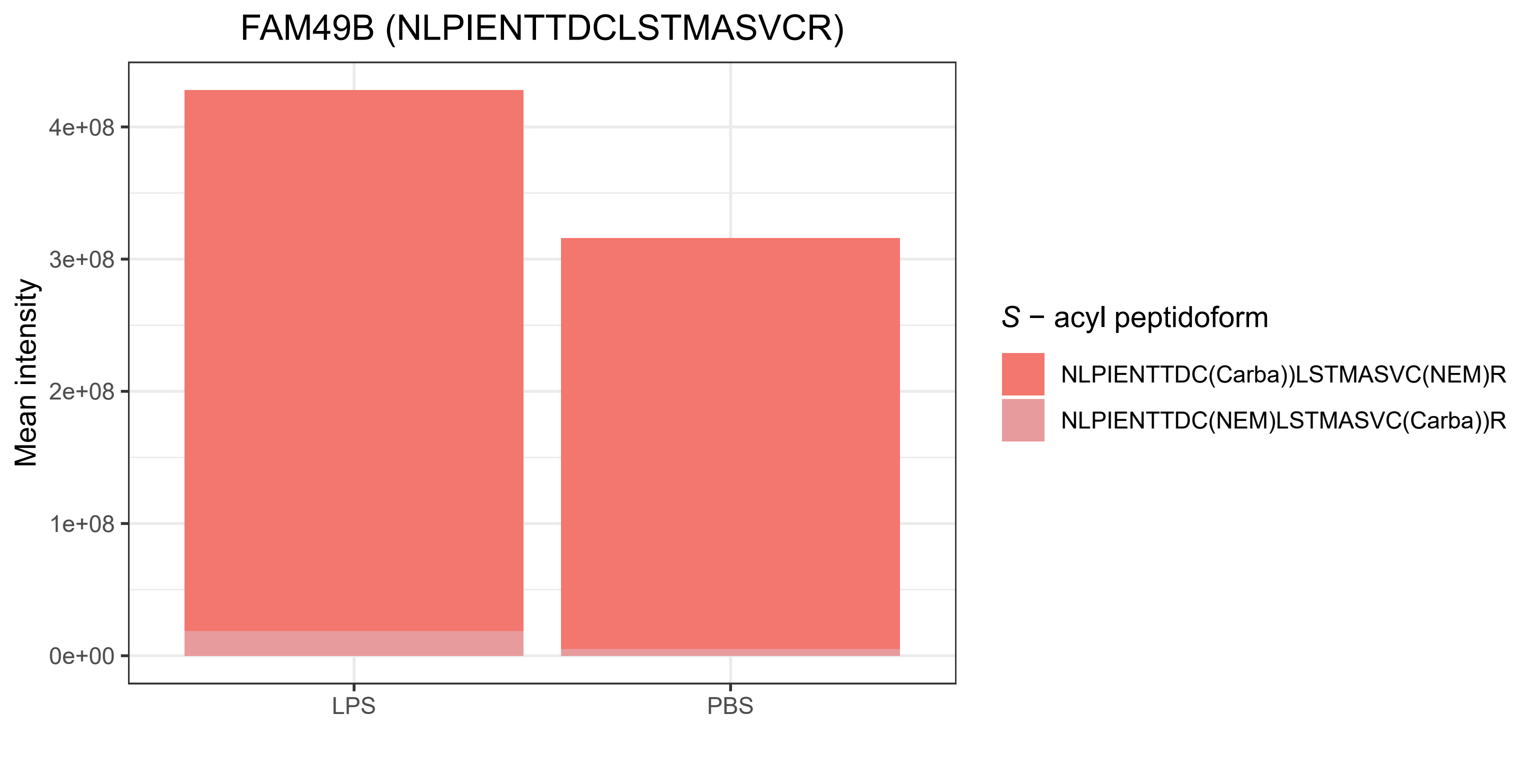
FAM49B (NLPIENTTDCLSTMASVCR)
Mean intensity
4e+08
3e+08
2e+08
1e+08
0e+00
LPS
PBS
S − acyl peptidoform
NLPIENTTDC(Carba))LSTMASVC(NEM)R
NLPIENTTDC(NEM)LSTMASVC(Carba))R

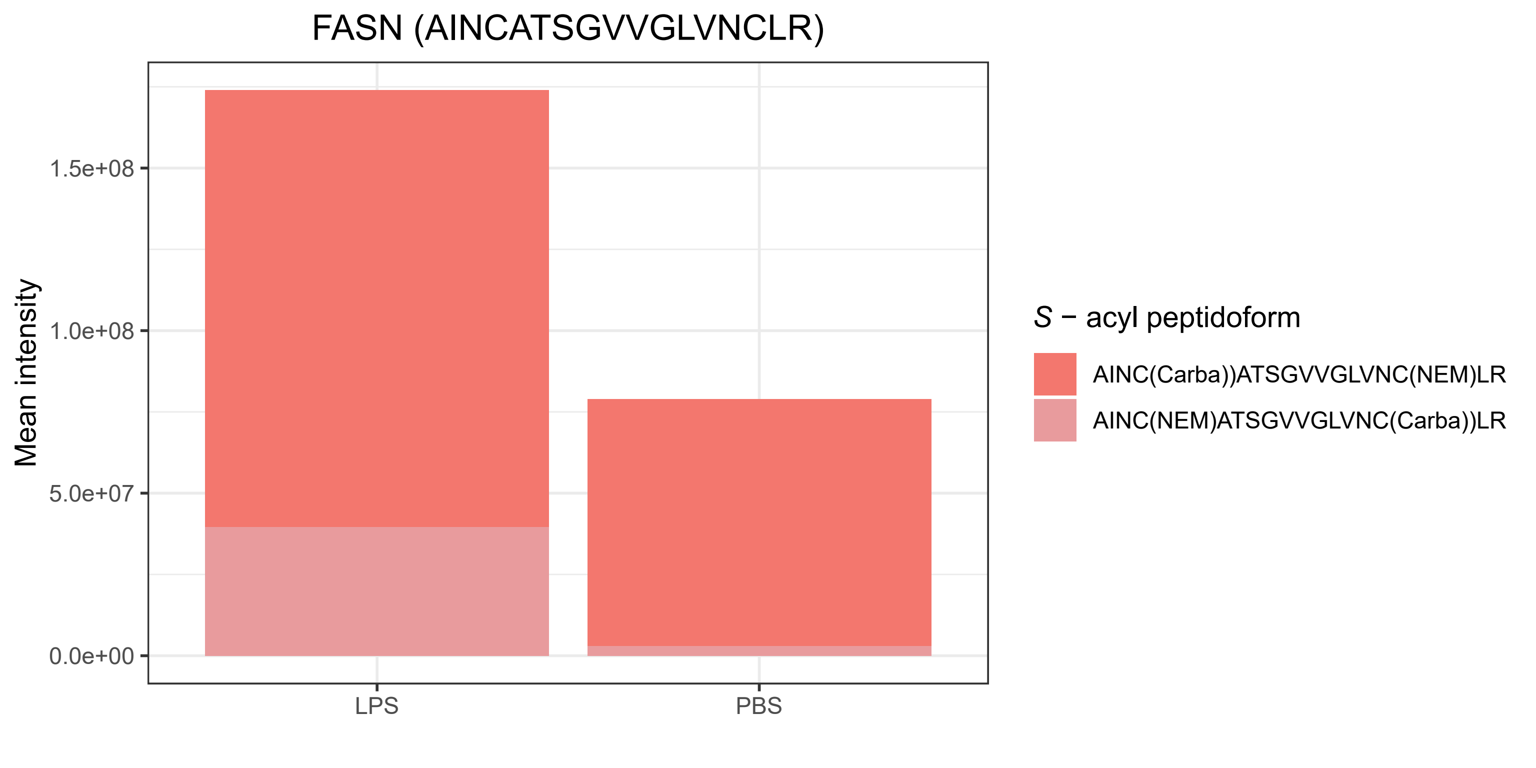
FASN (AINCATSGVVGLVNCLR)
Mean intensity
1.5e+08
1.0e+08
5.0e+07
0.0e+00
LPS
PBS
S − acyl peptidoform
AINC(Carba))ATSGVVGLVNC(NEM)LR
AINC(NEM)ATSGVVGLVNC(Carba))LR

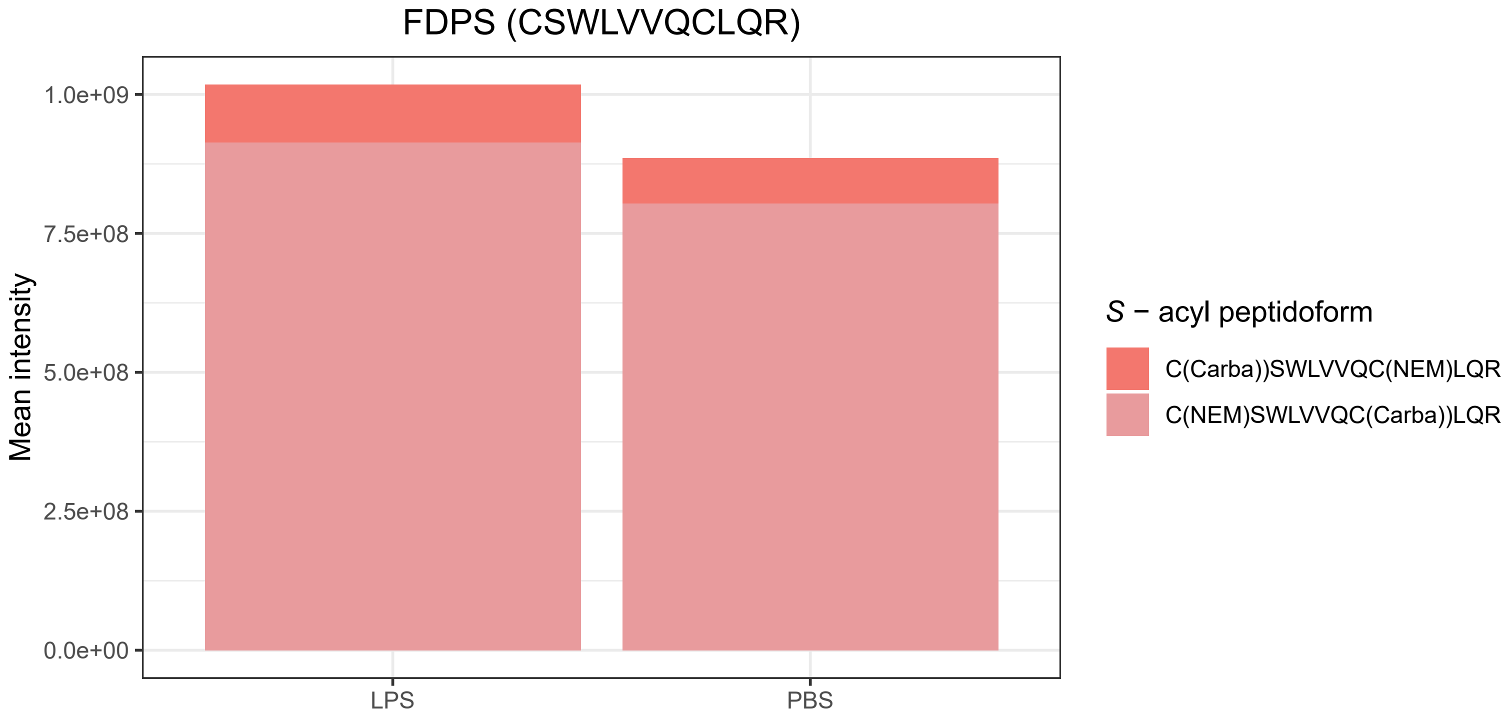
FDPS (CSWLVVQCLQR)
Mean intensity
1.0e+09
7.5e+08
5.0e+08
2.5e+08
0.0e+00
LPS
PBS
S − acyl peptidoform
C(Carba))SWLVVQC(NEM)LQR
C(NEM)SWLVVQC(Carba))LQR

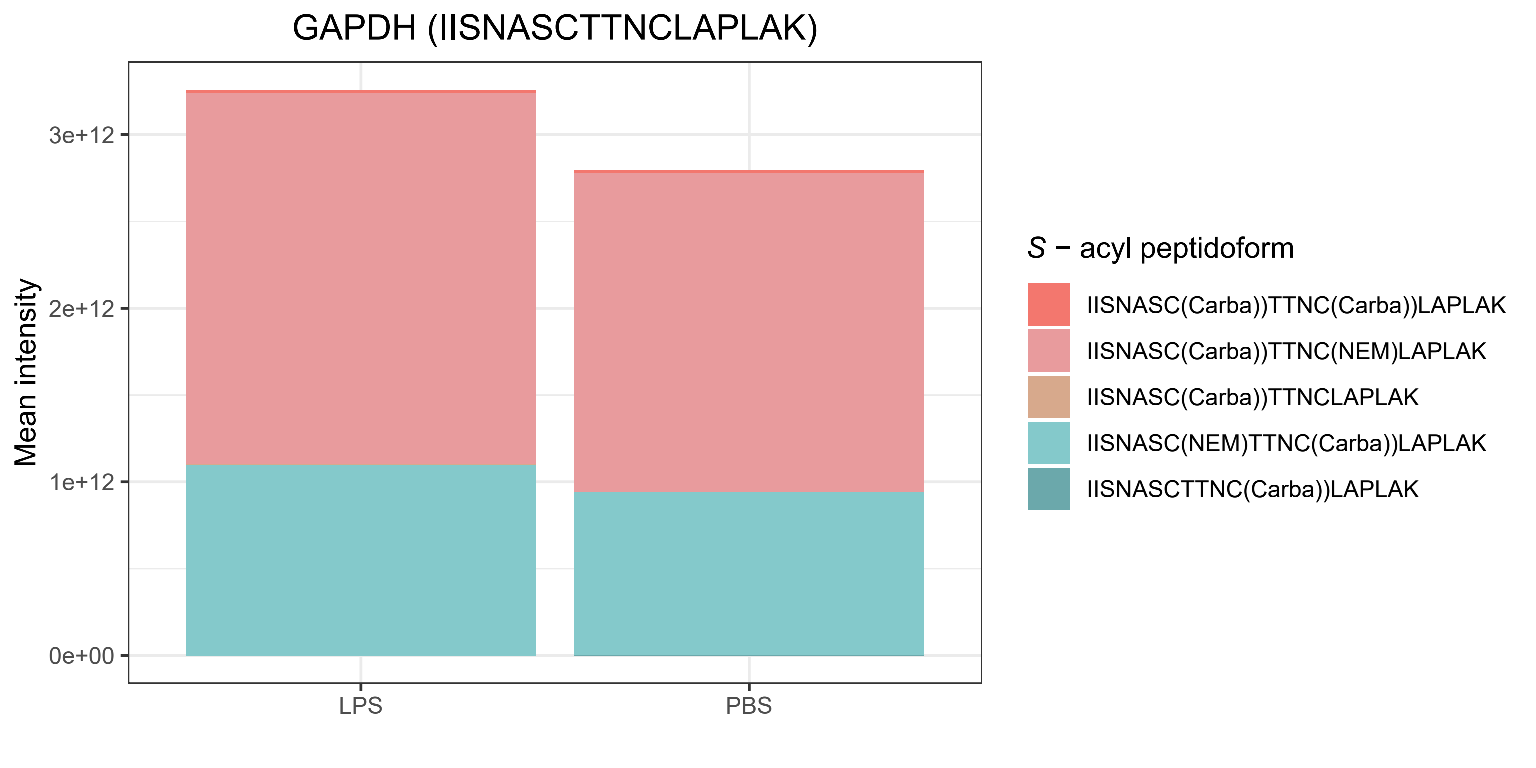

GAPDH (IISNASCTTNCLAPLAK)
Mean intensity
3e+12
2e+12
1e+12
0e+00
LPS
PBS
S − acyl peptidoform
IISNASC(Carba))TTNC(Carba))LAPLAK
IISNASC(Carba))TTNC(NEM)LAPLAK
IISNASC(Carba))TTNCLAPLAK
IISNASC(NEM)TTNC(Carba))LAPLAK
IISNASCTTNC(Carba))LAPLAK

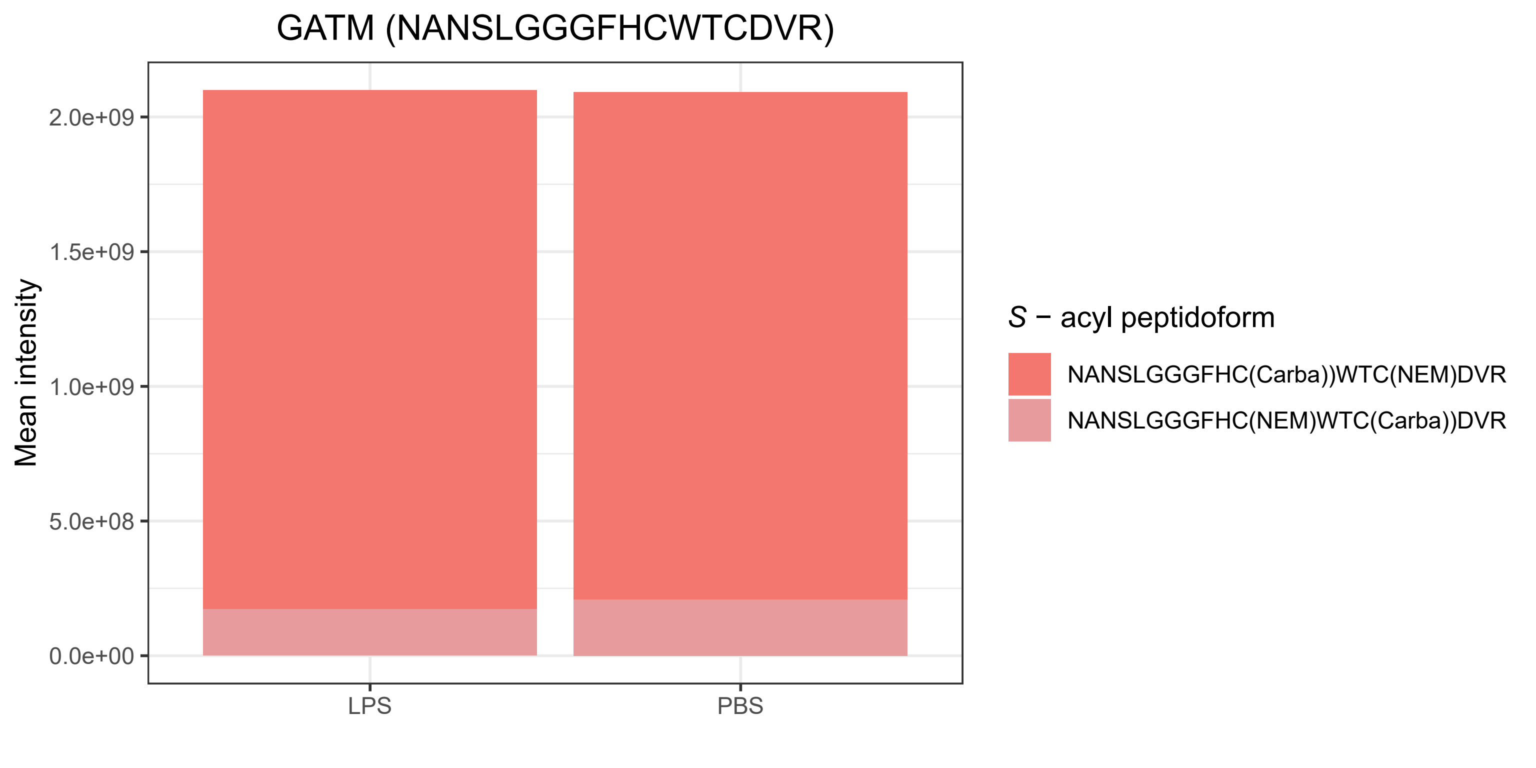

GATM (NANSLGGGFHCWTCDVR)
Mean intensity
2.0e+09
1.5e+09
1.0e+09
5.0e+08
0.0e+00
LPS
PBS
S − acyl peptidoform
NANSLGGGFHC(Carba))WTC(NEM)DVR
NANSLGGGFHC(NEM)WTC(Carba))DVR

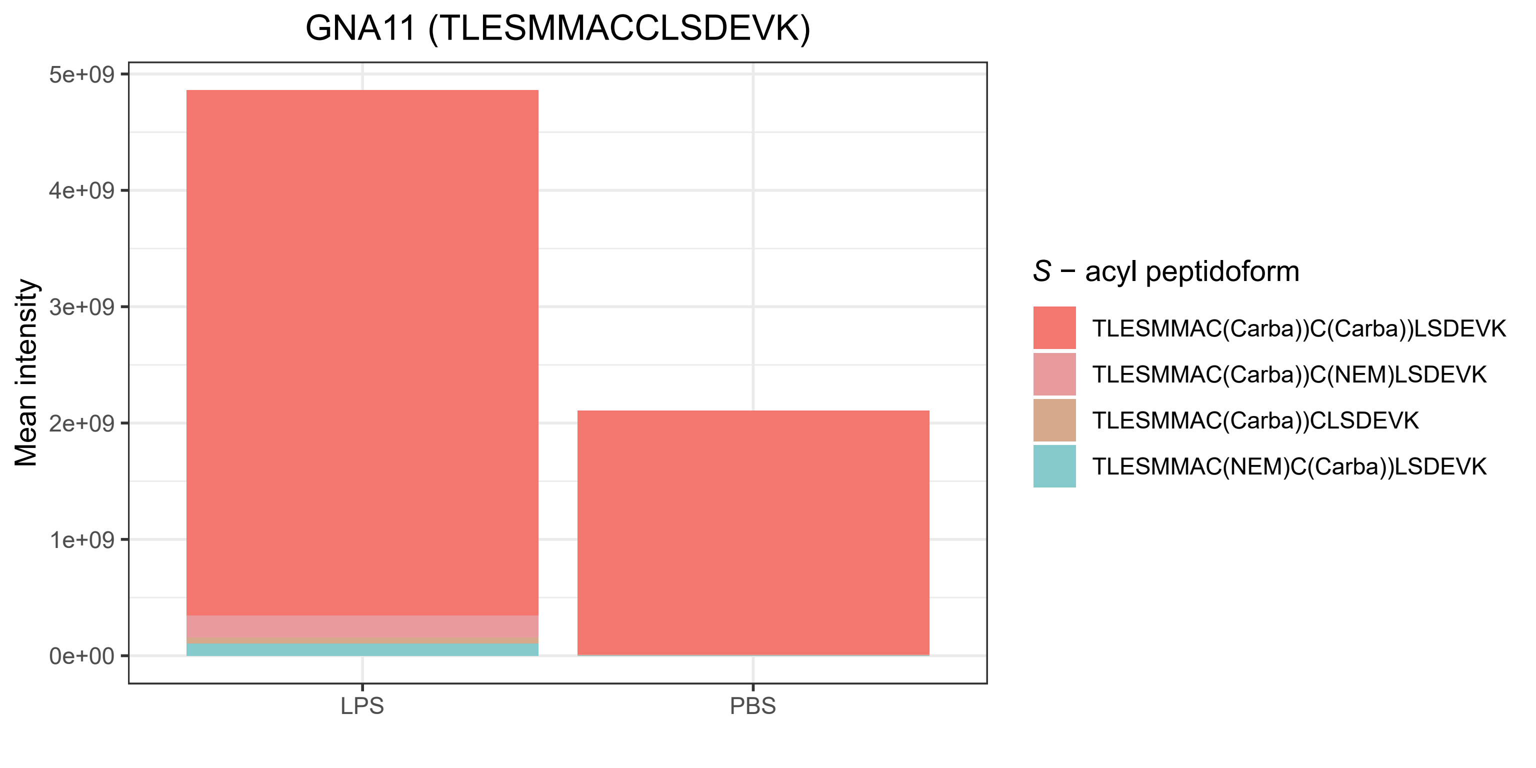

GNA11 (TLESMMACCLSDEVK)
Mean intensity
5e+09
4e+09
3e+09
2e+09
1e+09
0e+00
LPS
PBS
S − acyl peptidoform
TLESMMAC(Carba))C(Carba))LSDEVK
TLESMMAC(Carba))C(NEM)LSDEVK
TLESMMAC(Carba))CLSDEVK
TLESMMAC(NEM)C(Carba))LSDEVK

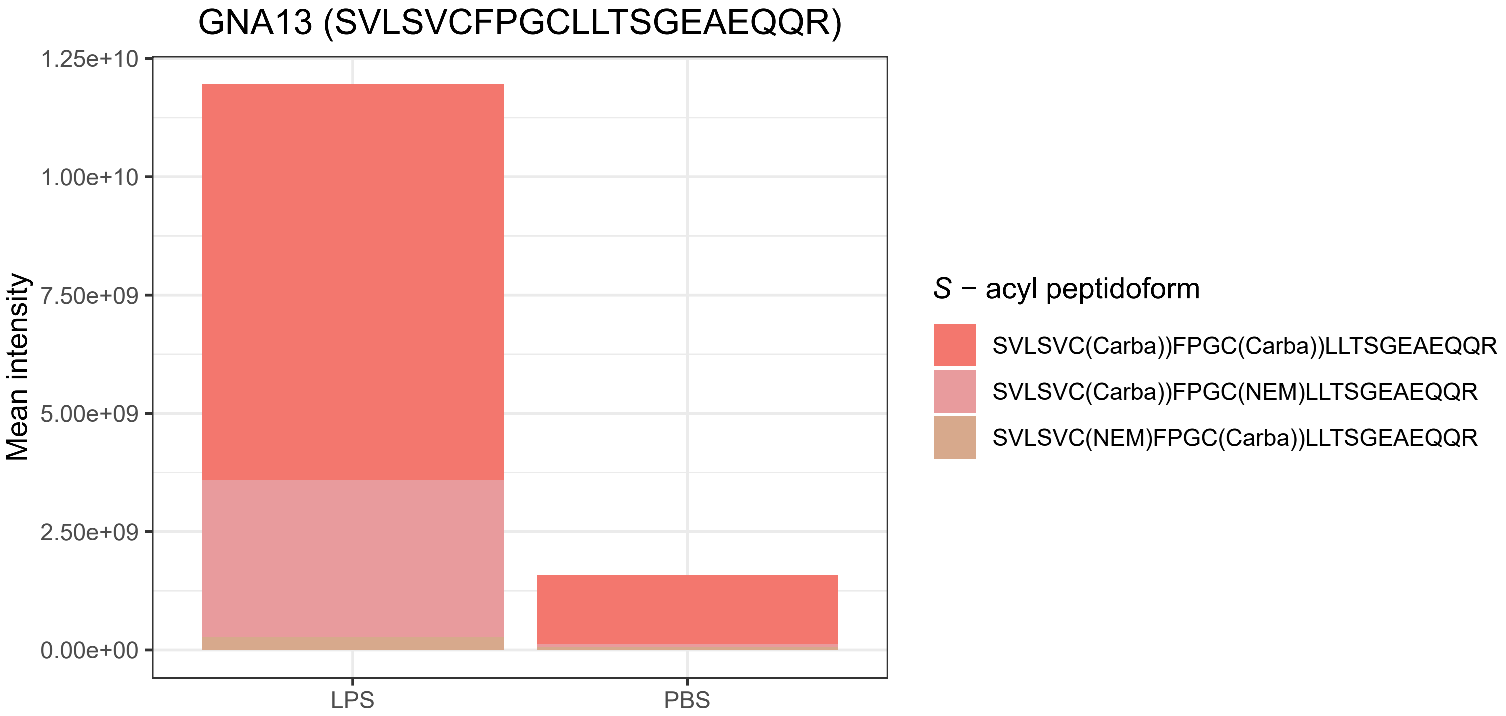
GNA13 (SVLSVCFPGCLLTSGEAEQQR)
Mean intensity
1.25e+10
1.00e+10
7.50e+09
5.00e+09
2.50e+09
0.00e+00
LPS
PBS
S − acyl peptidoform
SVLSVC(Carba))FPGC(Carba))LLTSGEAEQQR
SVLSVC(Carba))FPGC(NEM)LLTSGEAEQQR
SVLSVC(NEM)FPGC(Carba))LLTSGEAEQQR

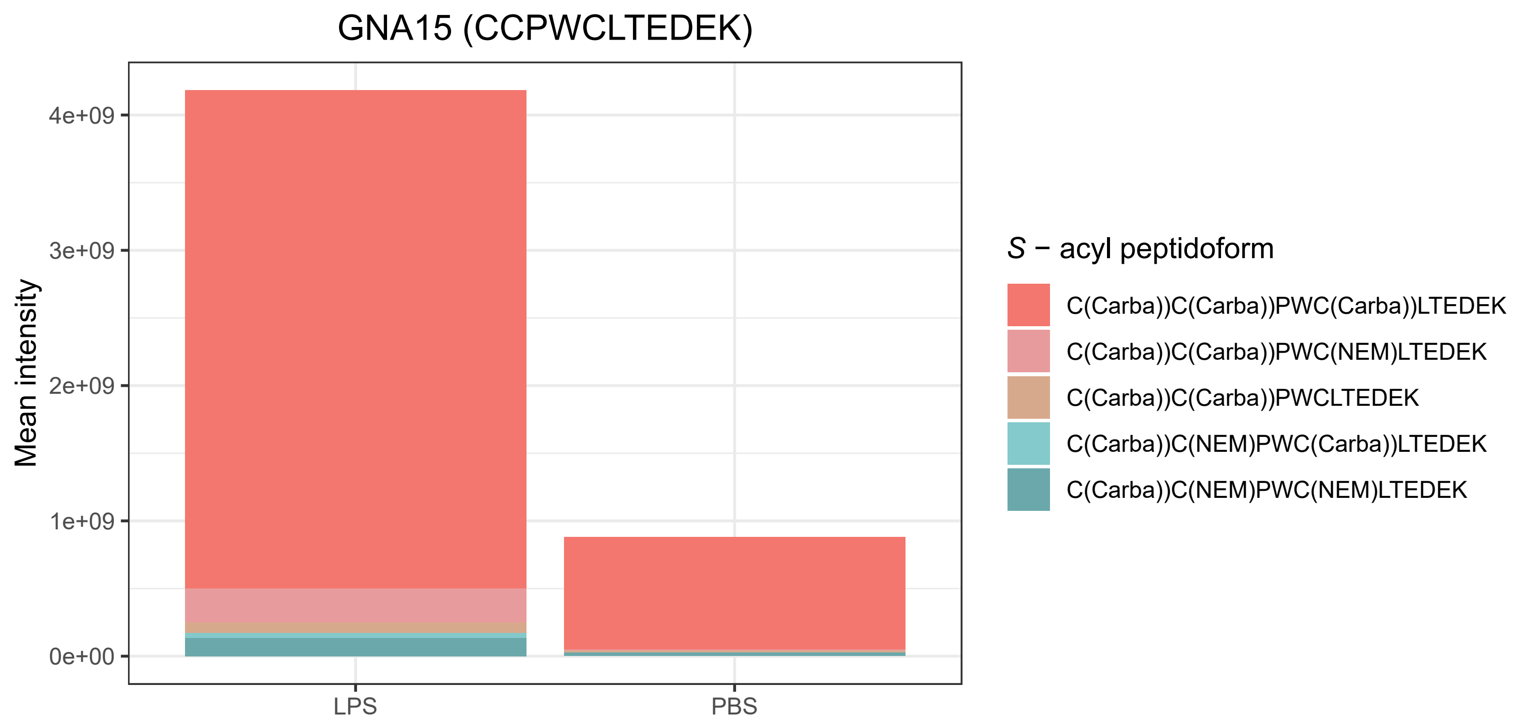

GNA15 (CCPWCLTEDEK)
Mean intensity
4e+09
3e+09
2e+09
1e+09
0e+00
LPS
PBS
S − acyl peptidoform
C(Carba))C(Carba))PWC(Carba))LTEDEK
C(Carba))C(Carba))PWC(NEM)LTEDEK
C(Carba))C(Carba))PWCLTEDEK
C(Carba))C(NEM)PWC(Carba))LTEDEK
C(Carba))C(NEM)PWC(NEM)LTEDEK

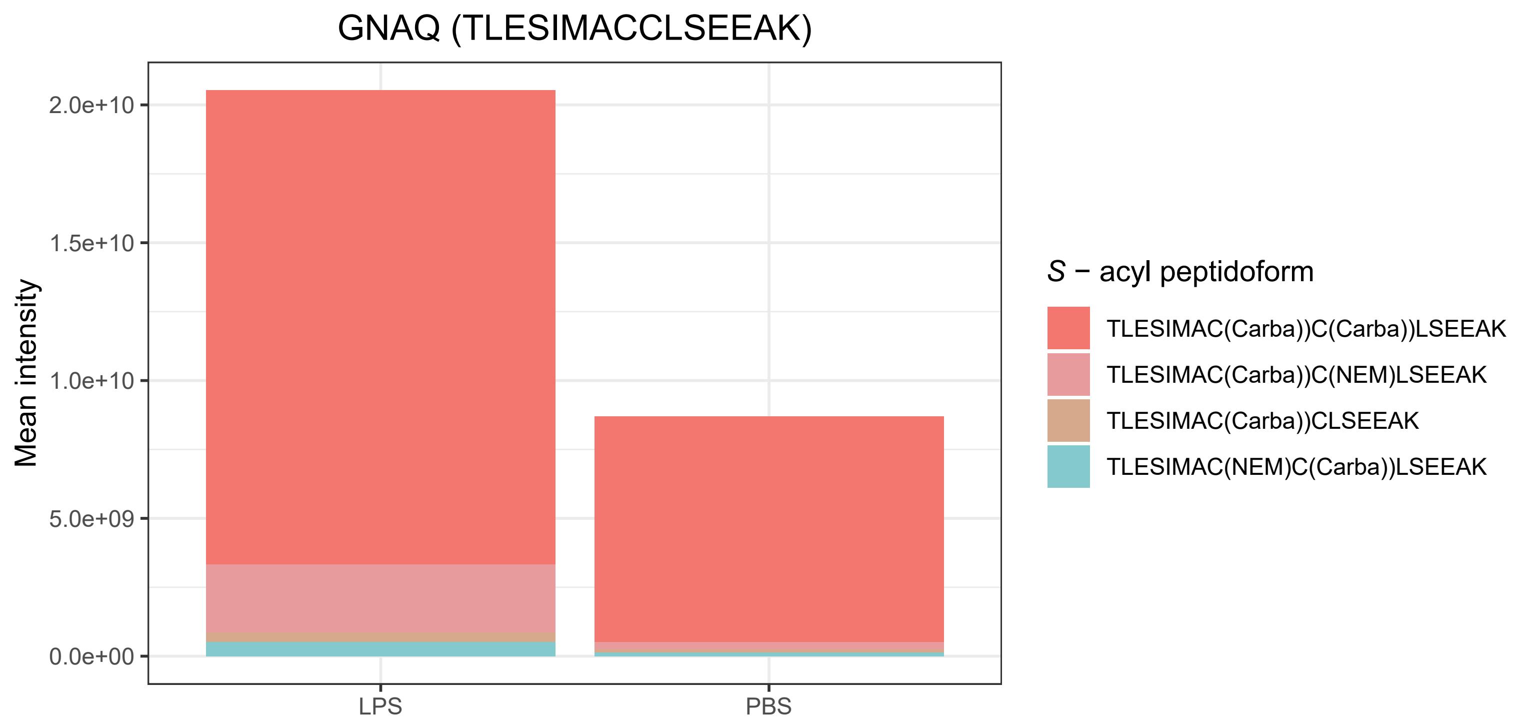

GNAQ (TLESIMACCLSEEAK)
Mean intensity
2.0e+10
1.5e+10
1.0e+10
5.0e+09
0.0e+00
LPS
PBS
S − acyl peptidoform
TLESIMAC(Carba))C(Carba))LSEEAK
TLESIMAC(Carba))C(NEM)LSEEAK
TLESIMAC(Carba))CLSEEAK
TLESIMAC(NEM)C(Carba))LSEEAK

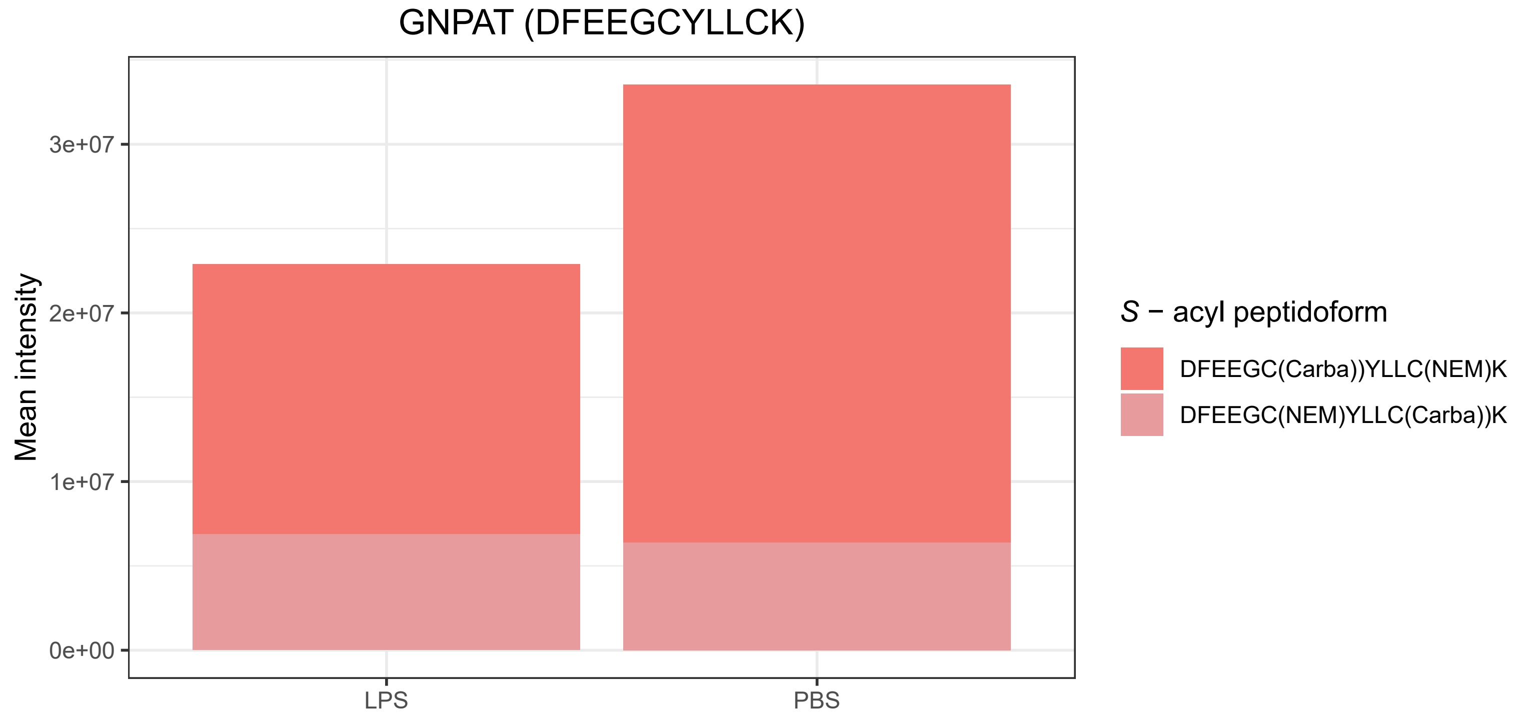
GNPAT (DFEEGCYLLCK)
Mean intensity
3e+07
2e+07
1e+07
0e+00
LPS
PBS
S − acyl peptidoform
DFEEGC(Carba))YLLC(NEM)K
DFEEGC(NEM)YLLC(Carba))K

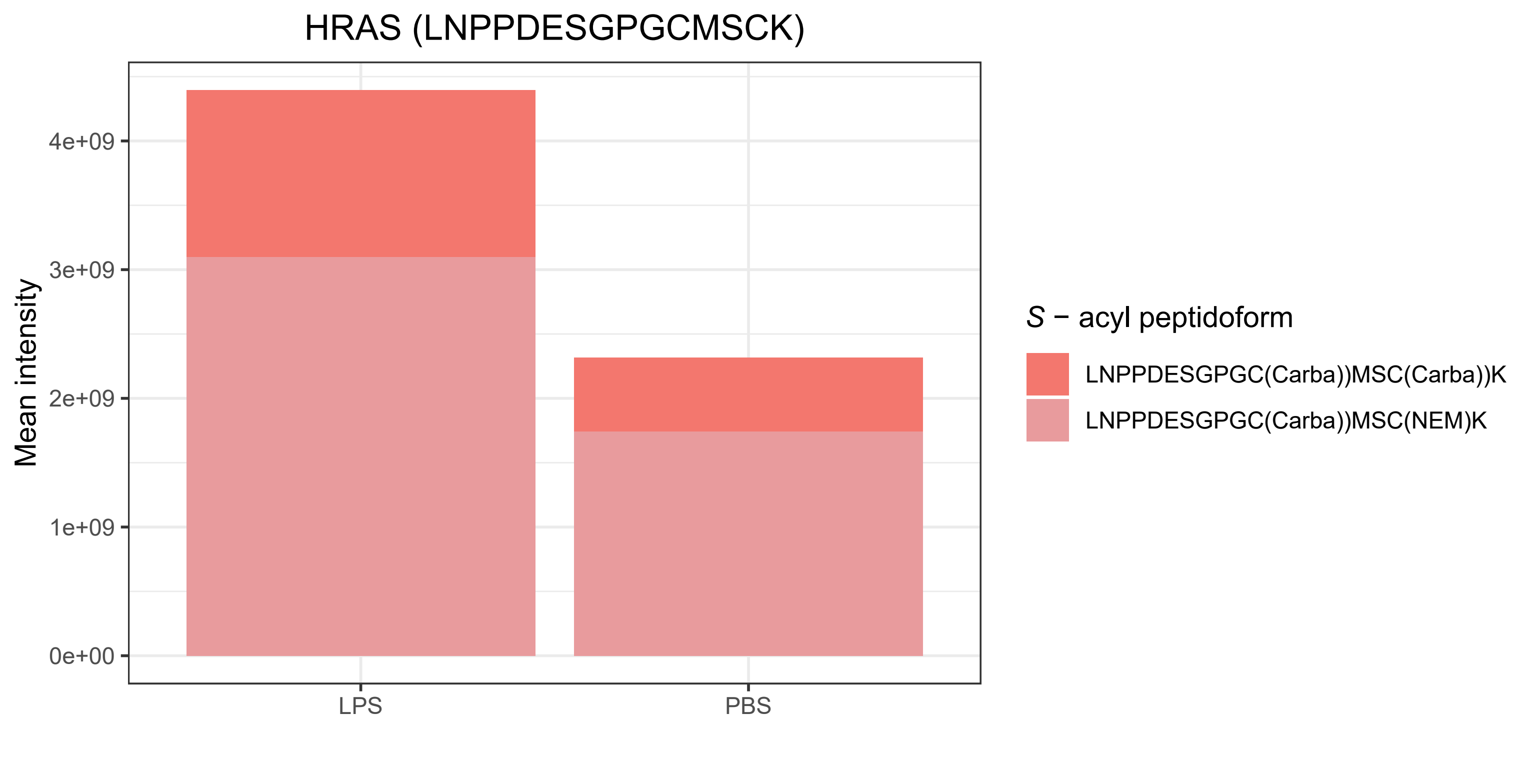

HRAS (LNPPDESGPGCMSCK)
Mean intensity
4e+09
3e+09
2e+09
1e+09
0e+00
LPS
PBS
S − acyl peptidoform
LNPPDESGPGC(Carba))MSC(Carba))K
LNPPDESGPGC(Carba))MSC(NEM)K

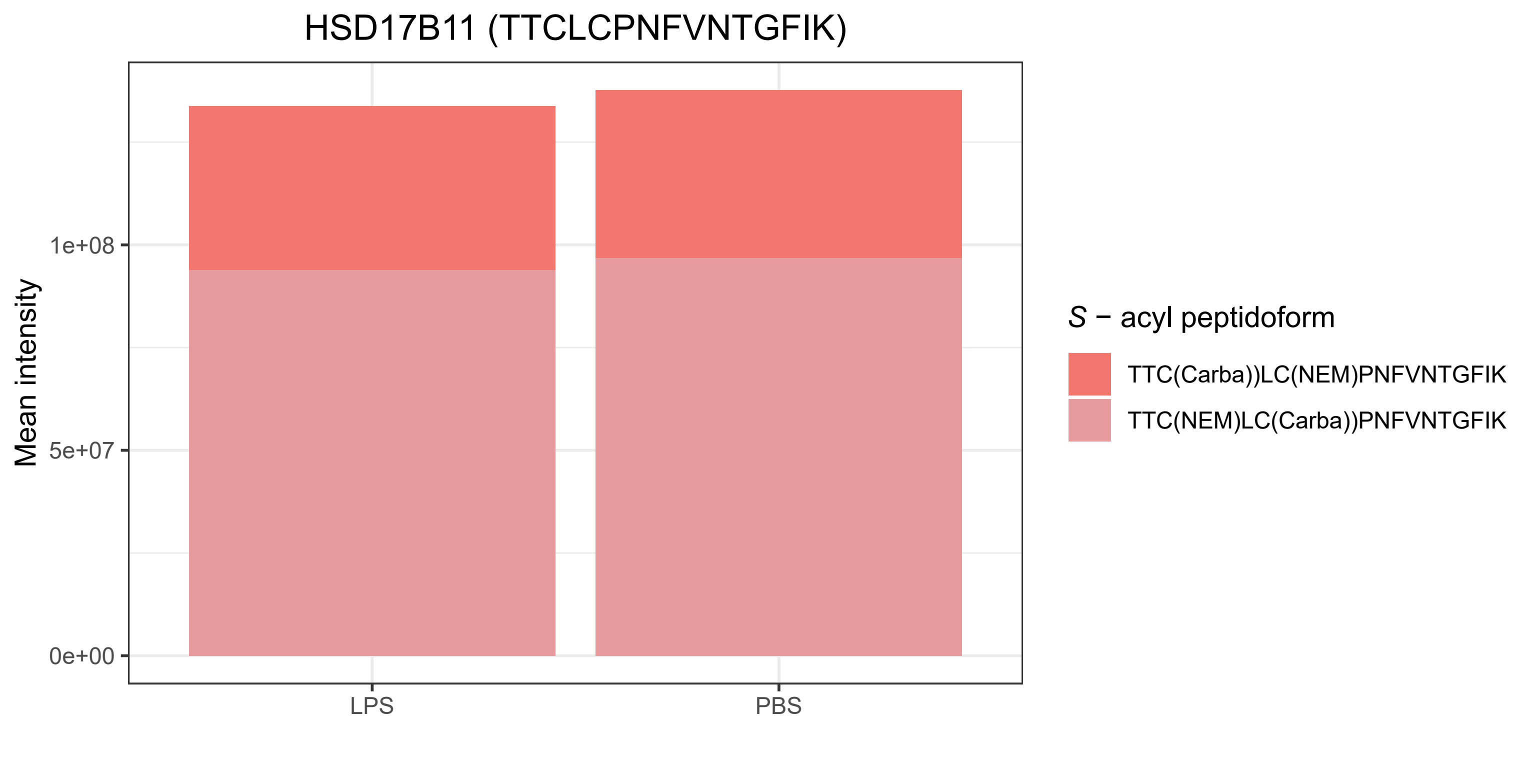
HSD17B11 (TTCLCPNFVNTGFIK)
Mean intensity
1e+08
5e+07
0e+00
LPS
PBS
S − acyl peptidoform
TTC(Carba))LC(NEM)PNFVNTGFIK
TTC(NEM)LC(Carba))PNFVNTGFIK

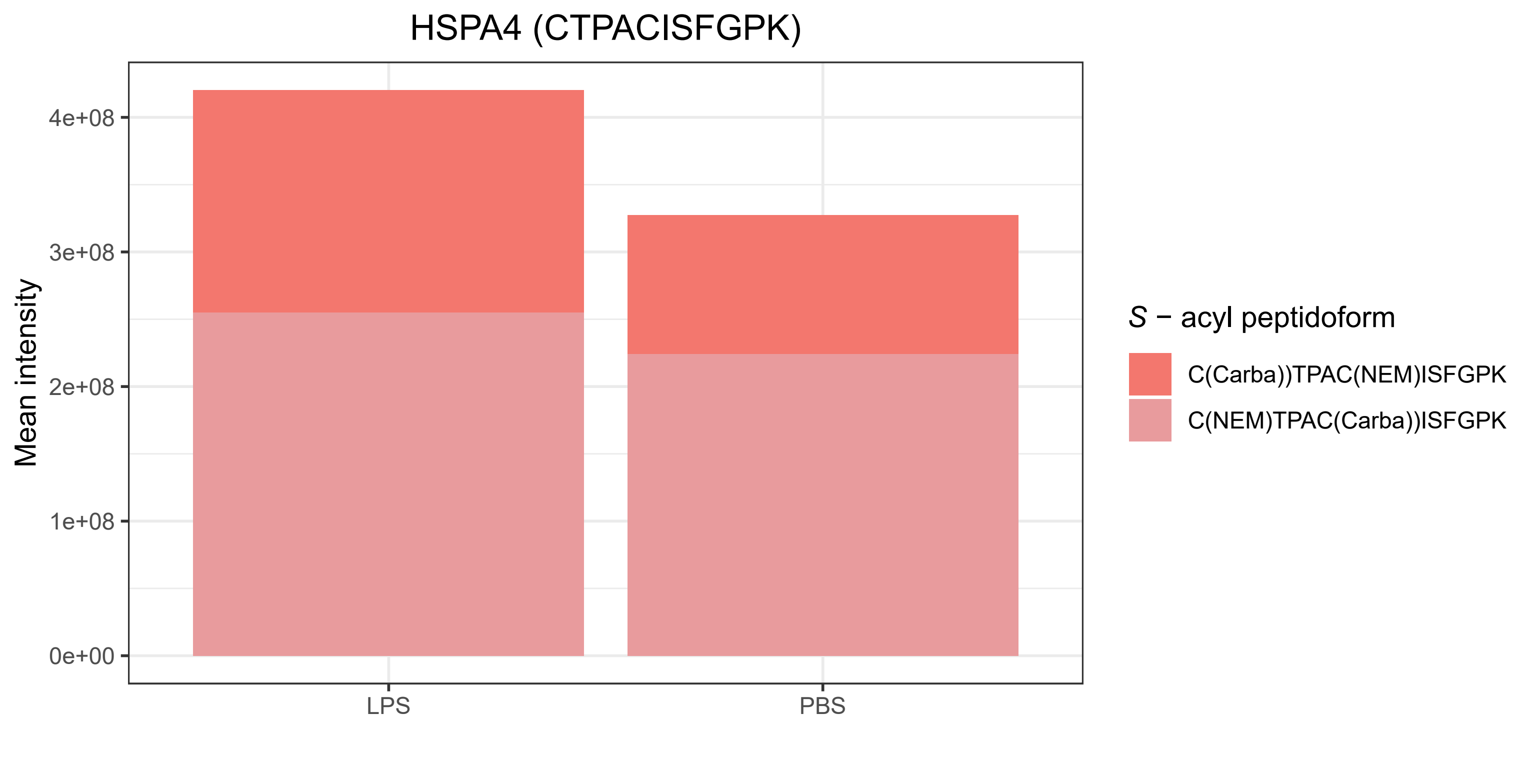
HSPA4 (CTPACISFGPK)
Mean intensity
4e+08
3e+08
2e+08
1e+08
0e+00
LPS
PBS
S − acyl peptidoform
C(Carba))TPAC(NEM)ISFGPK
C(NEM)TPAC(Carba))ISFGPK

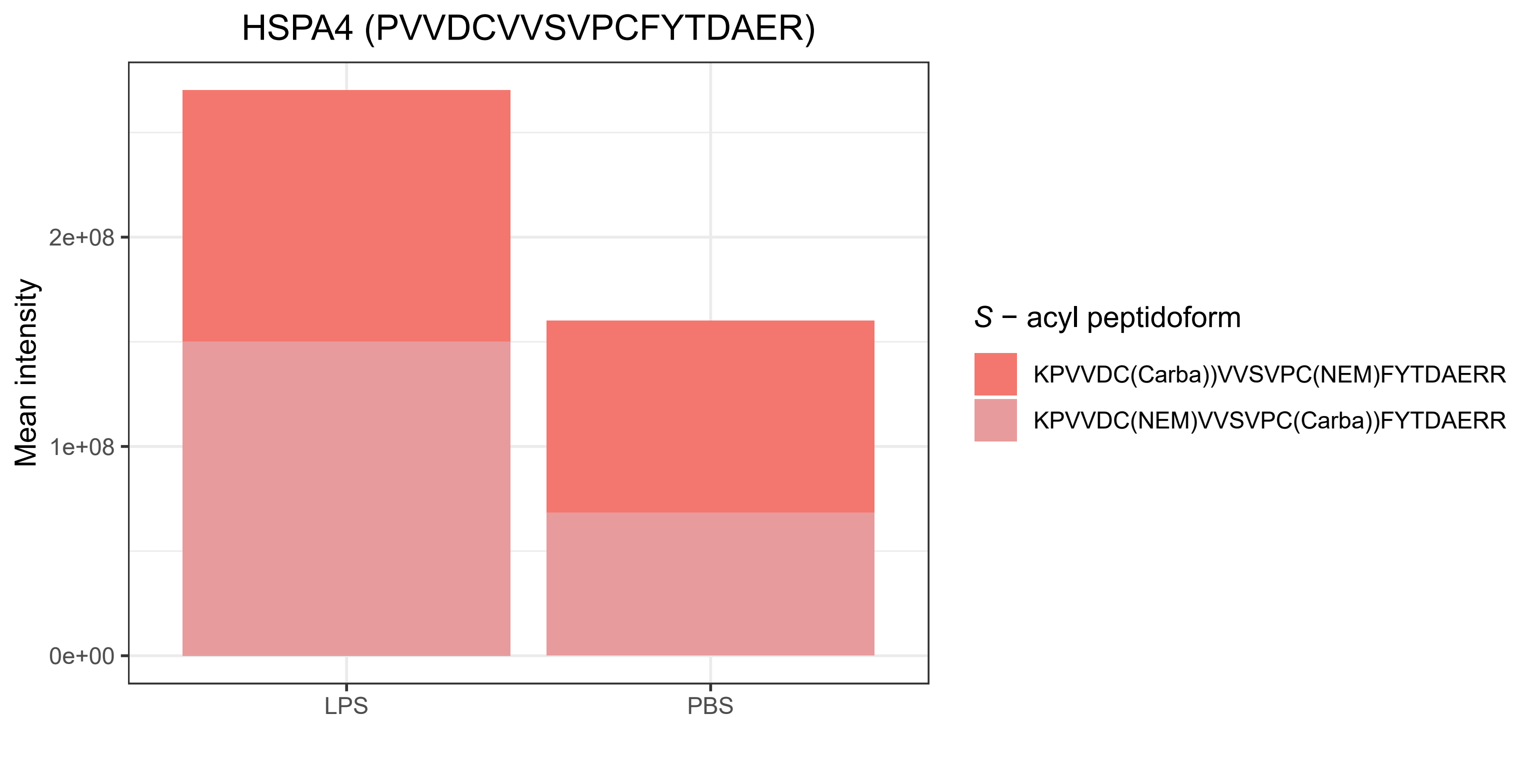
HSPA4 (PVVDCVVSVPCFYTDAER)
Mean intensity
2e+08
1e+08
0e+00
LPS
PBS
S − acyl peptidoform
KPVVDC(Carba))VVSVPC(NEM)FYTDAERR
KPVVDC(NEM)VVSVPC(Carba))FYTDAERR

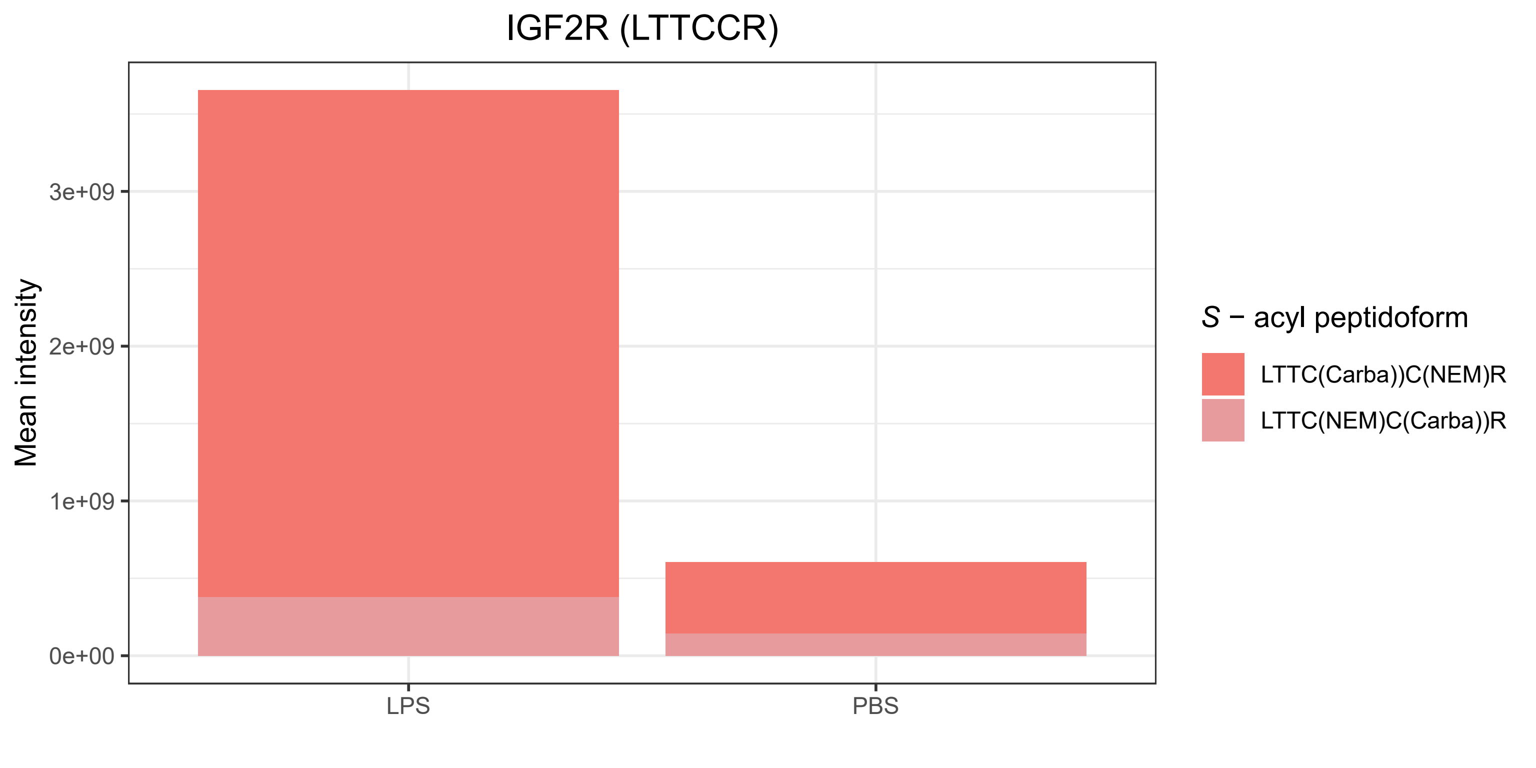

IGF2R (LTTCCR)
Mean intensity
3e+09
2e+09
1e+09
0e+00
LPS
PBS
S − acyl peptidoform
LTTC(Carba))C(NEM)R
LTTC(NEM)C(Carba))R

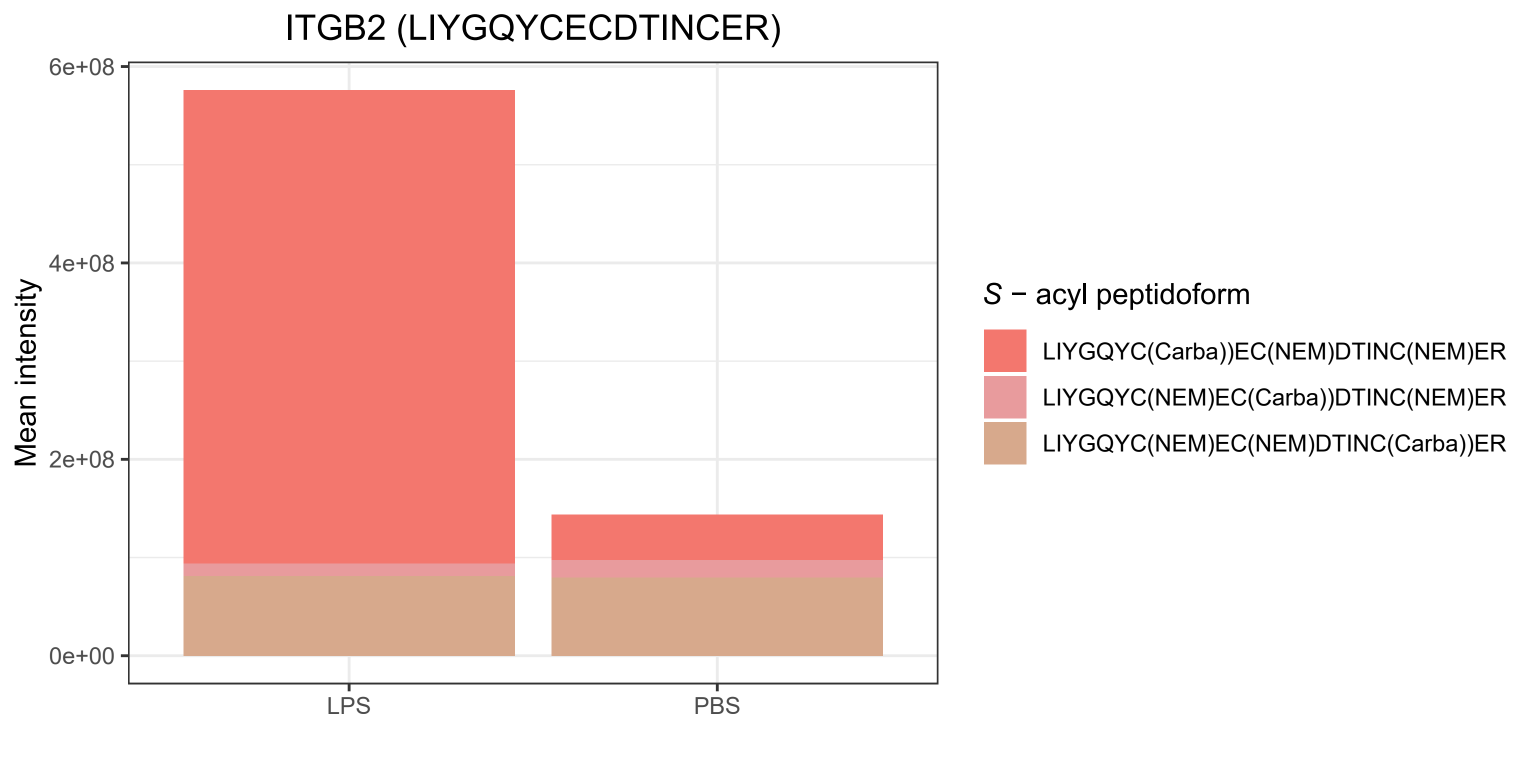

ITGB2 (LIYGQYCECDTINCER)
Mean intensity
6e+08
4e+08
2e+08
0e+00
LPS
PBS
S − acyl peptidoform
LIYGQYC(Carba))EC(NEM)DTINC(NEM)ER
LIYGQYC(NEM)EC(Carba))DTINC(NEM)ER
LIYGQYC(NEM)EC(NEM)DTINC(Carba))ER

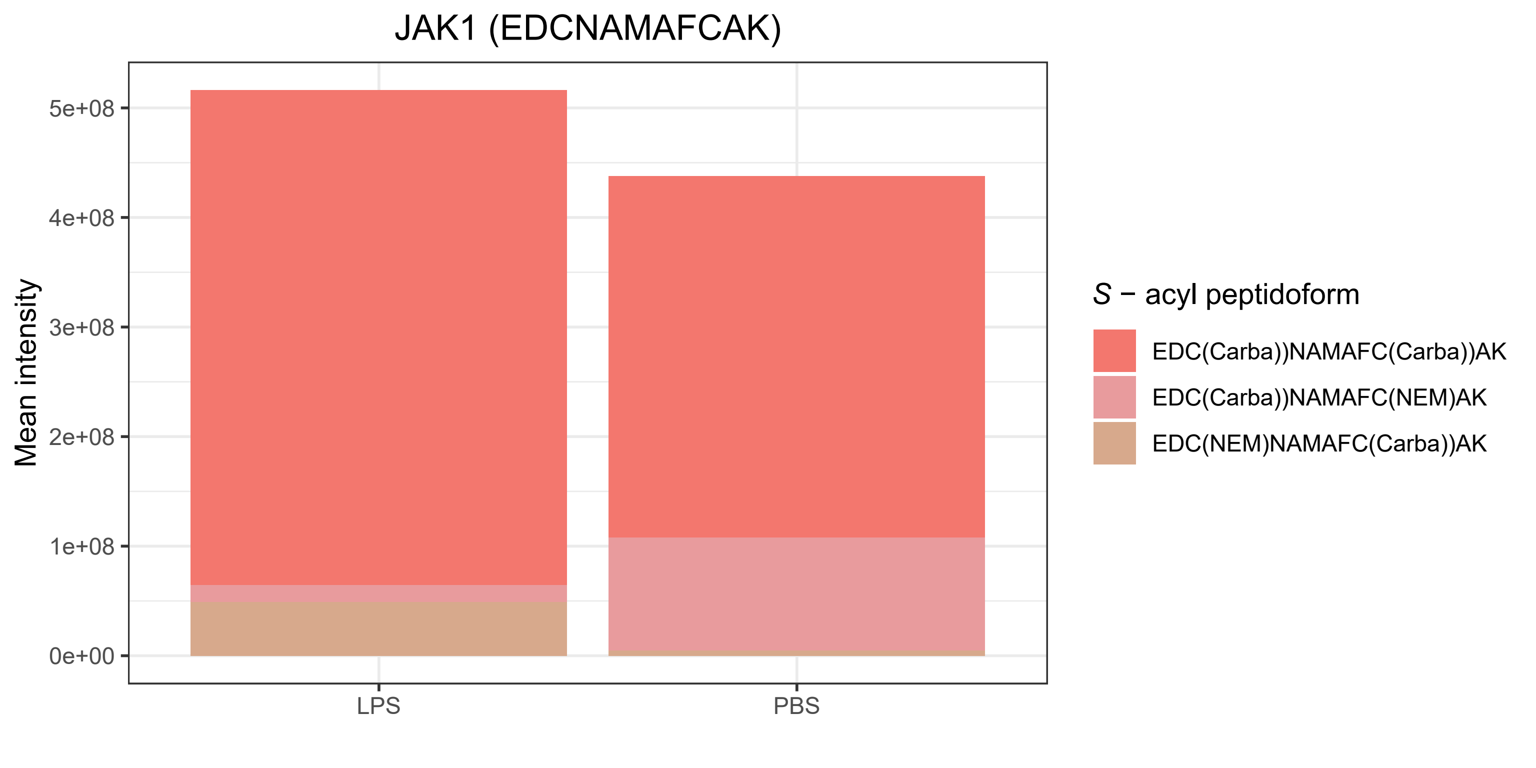
JAK1 (EDCNAMAFCAK)
Mean intensity
5e+08
4e+08
3e+08
2e+08
1e+08
0e+00
LPS
PBS
S − acyl peptidoform
EDC(Carba))NAMAFC(Carba))AK
EDC(Carba))NAMAFC(NEM)AK
EDC(NEM)NAMAFC(Carba))AK

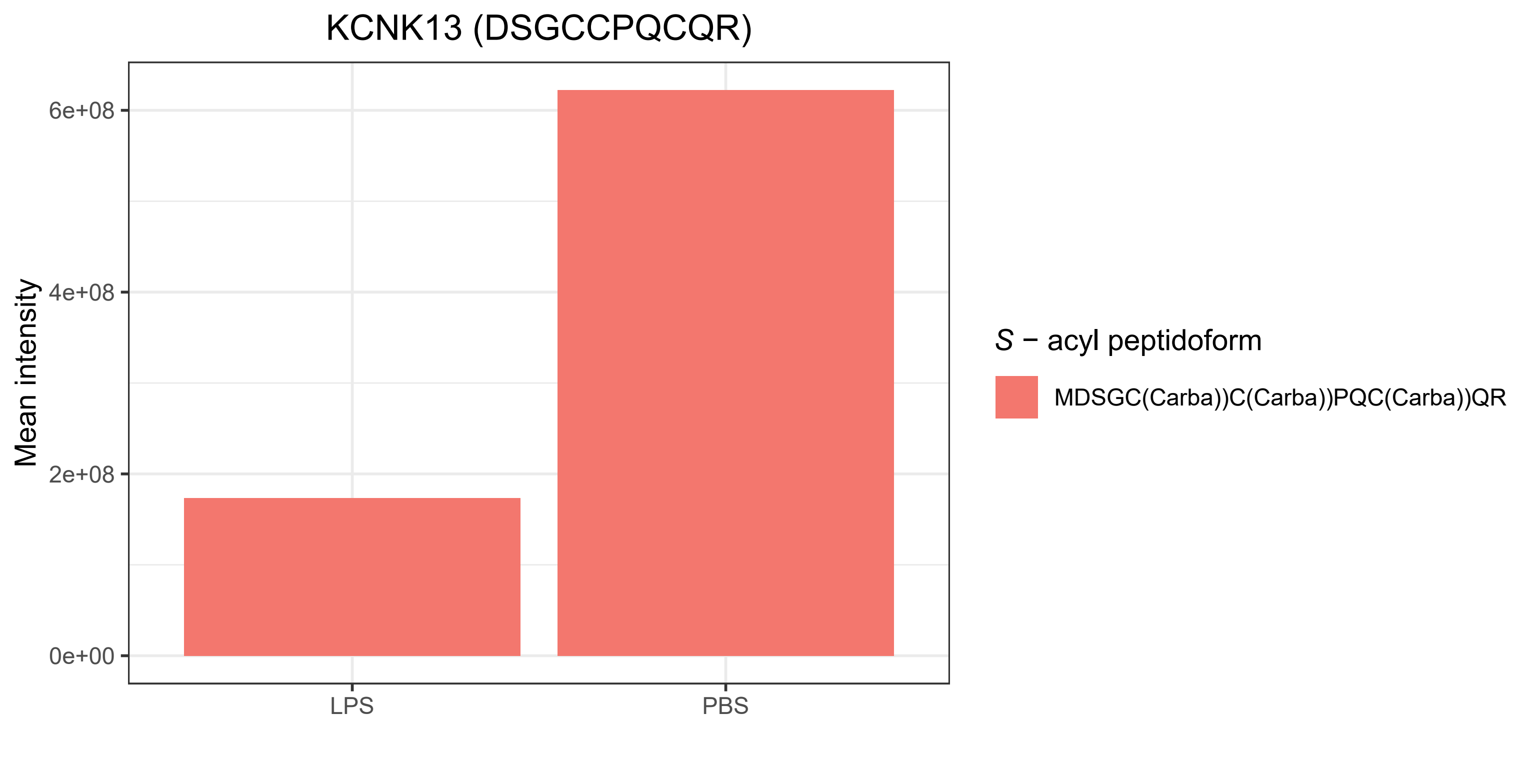
KCNK13 (DSGCCPQCQR)
Mean intensity
6e+08
4e+08
2e+08
0e+00
LPS
PBS
S − acyl peptidoform
MDSGC(Carba))C(Carba))PQC(Carba))QR

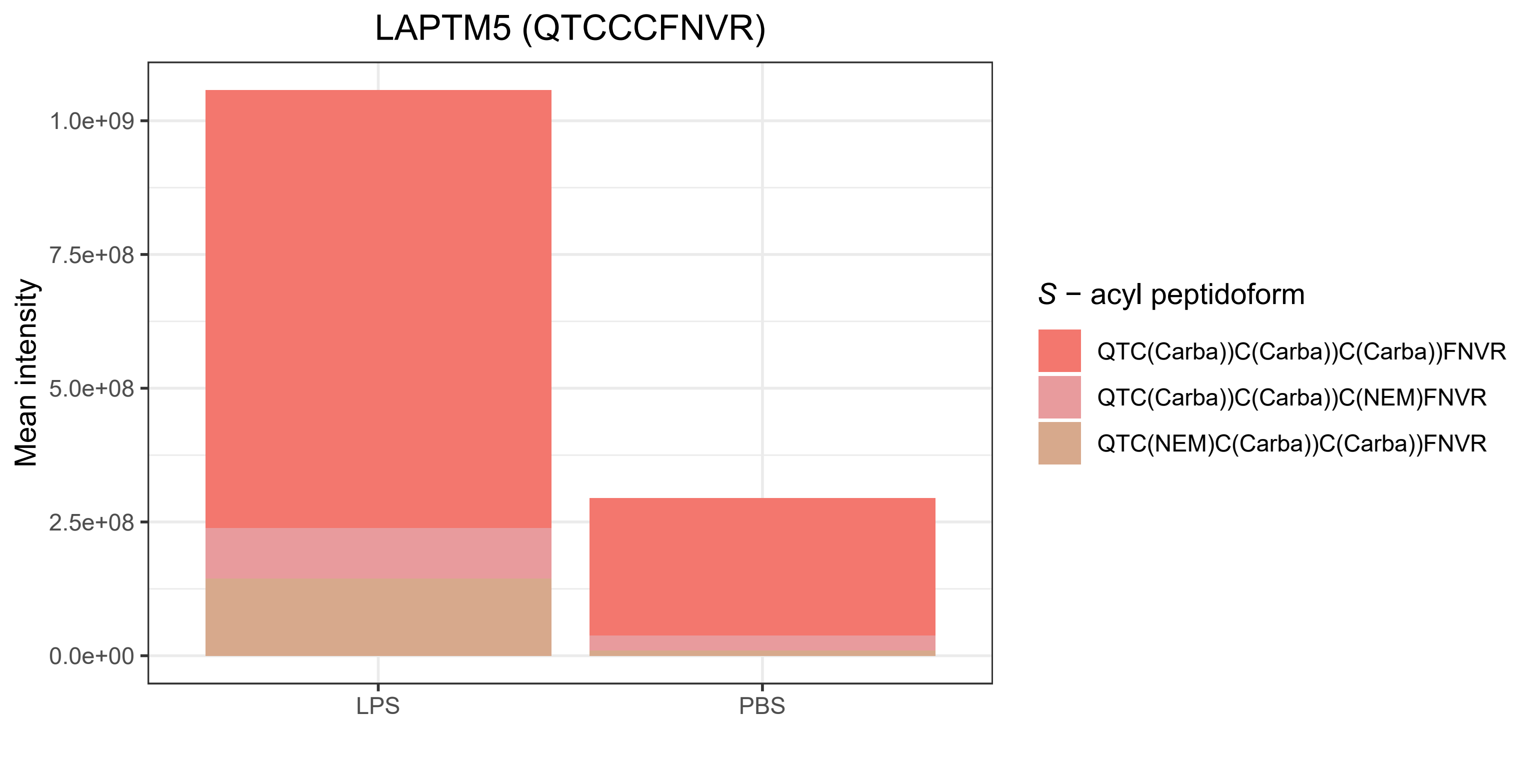

LAPTM5 (QTCCCFNVR)
Mean intensity
1.0e+09
7.5e+08
5.0e+08
2.5e+08
0.0e+00
LPS
PBS
S − acyl peptidoform
QTC(Carba))C(Carba))C(Carba))FNVR
QTC(Carba))C(Carba))C(NEM)FNVR
QTC(NEM)C(Carba))C(Carba))FNVR

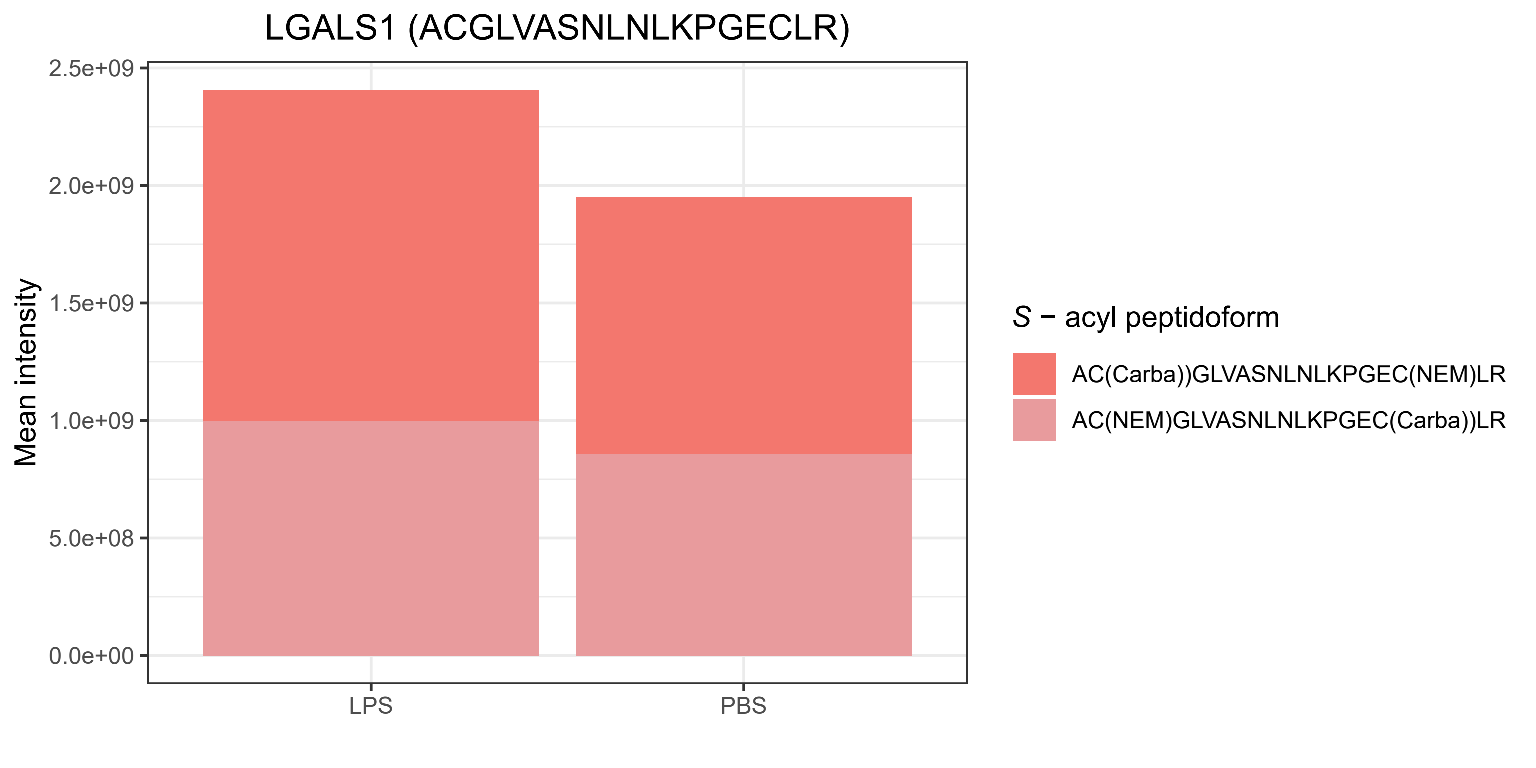

LGALS1 (ACGLVASNLNLKPGECLR)
Mean intensity
2.5e+09
2.0e+09
1.5e+09
1.0e+09
5.0e+08
0.0e+00
LPS
PBS
S − acyl peptidoform
AC(Carba))GLVASNLNLKPGEC(NEM)LR
AC(NEM)GLVASNLNLKPGEC(Carba))LR

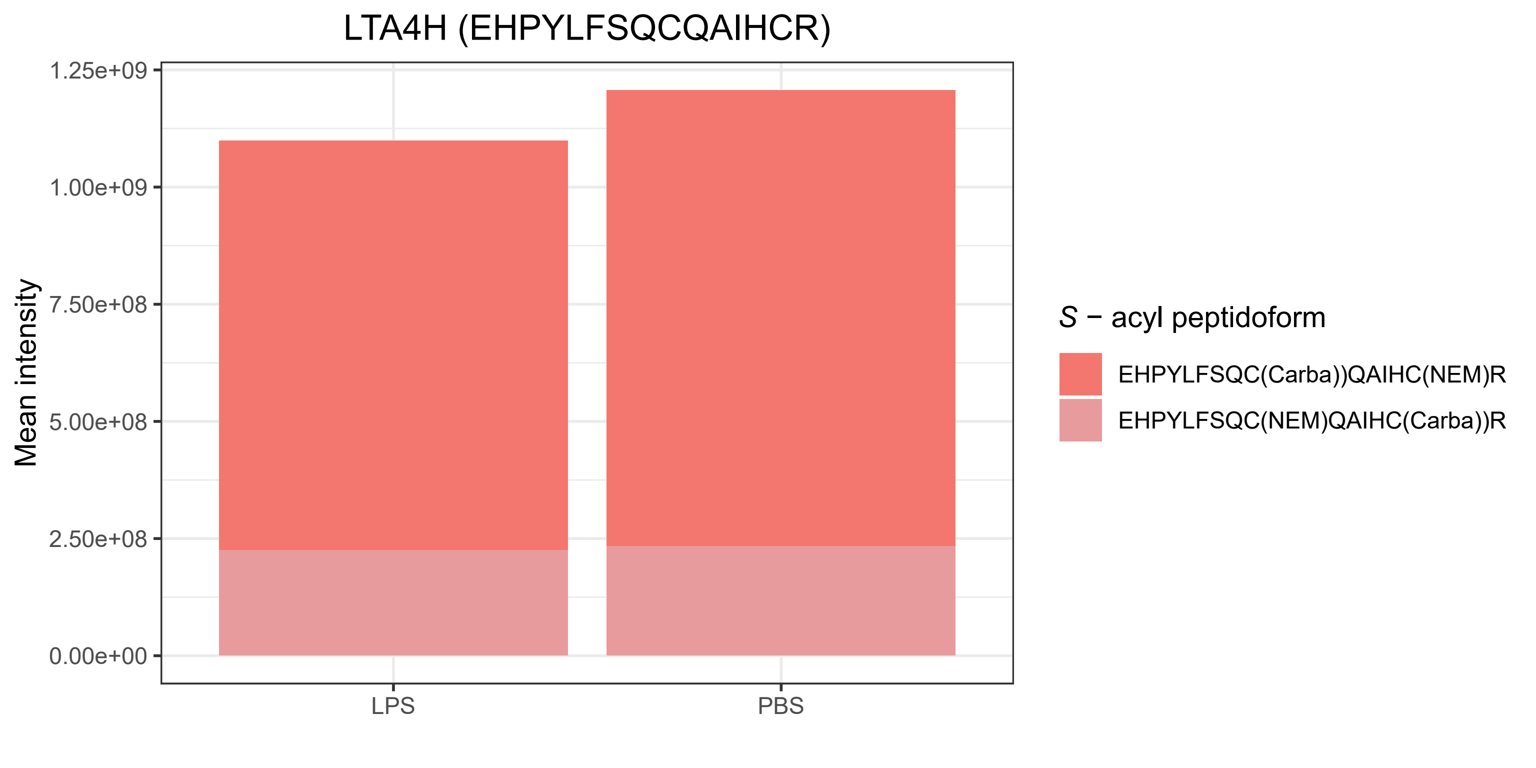
LTA4H (EHPYLFSQCQAIHCR)
Mean intensity
1.25e+09
1.00e+09
7.50e+08
5.00e+08
2.50e+08
0.00e+00
LPS
PBS
S − acyl peptidoform
EHPYLFSQC(Carba))QAIHC(NEM)R
EHPYLFSQC(NEM)QAIHC(Carba))R

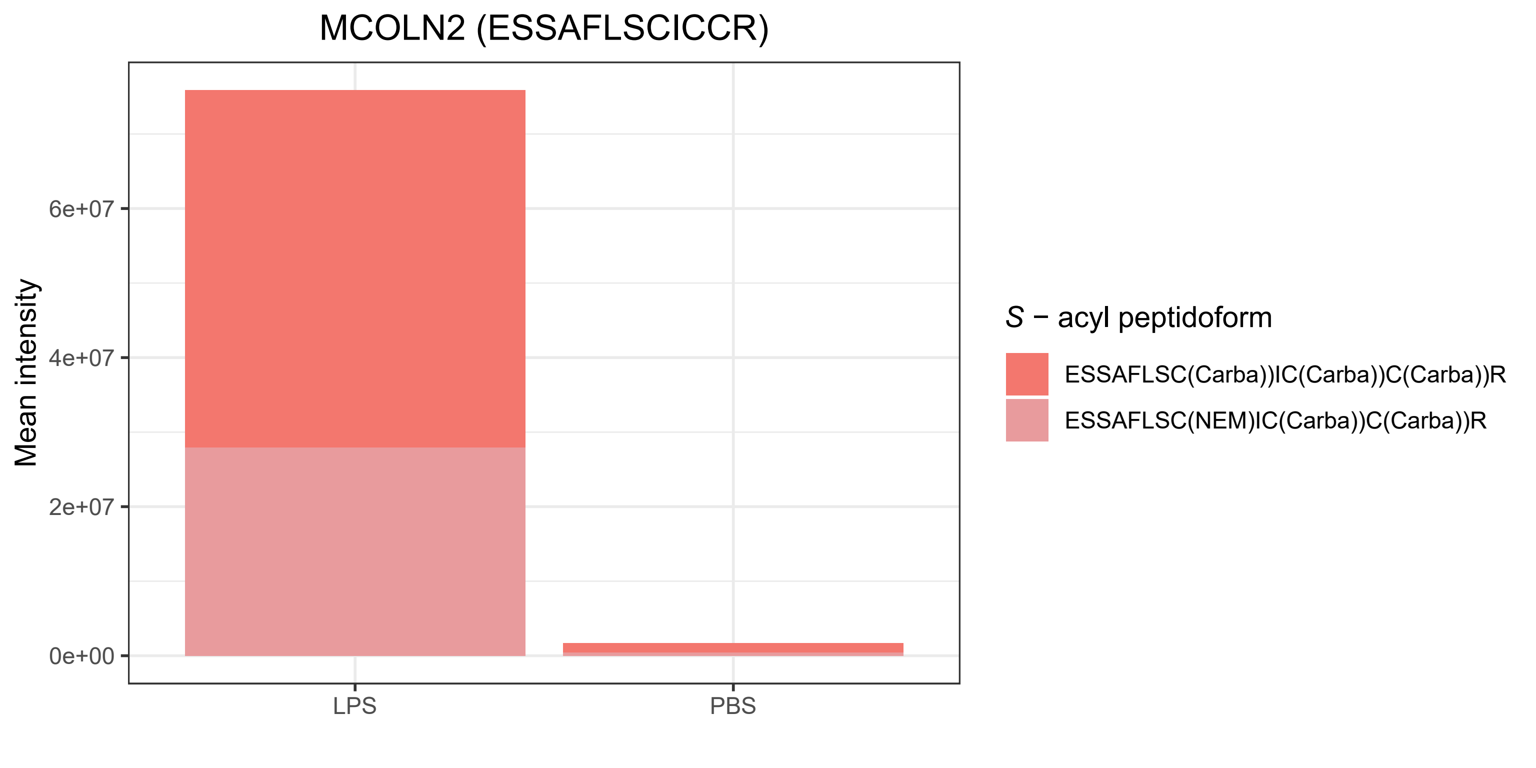

MCOLN2 (ESSAFLSCICCR)
Mean intensity
6e+07
4e+07
2e+07
0e+00
LPS
PBS
S − acyl peptidoform
ESSAFLSC(Carba))IC(Carba))C(Carba))R
ESSAFLSC(NEM)IC(Carba))C(Carba))R

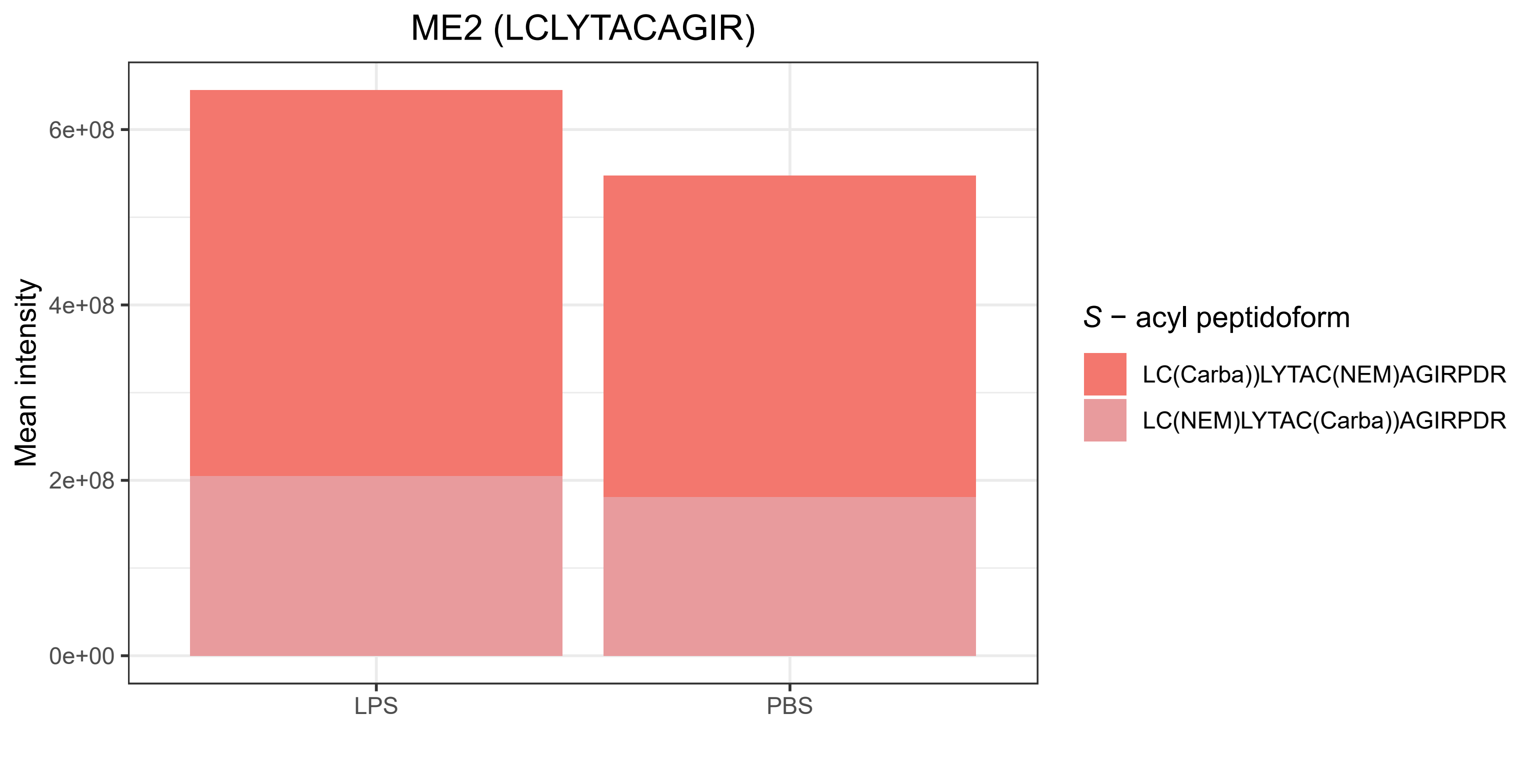
ME2 (LCLYTACAGIR)
Mean intensity
6e+08
4e+08
2e+08
0e+00
LPS
PBS
S − acyl peptidoform
LC(Carba))LYTAC(NEM)AGIRPDR
LC(NEM)LYTAC(Carba))AGIRPDR

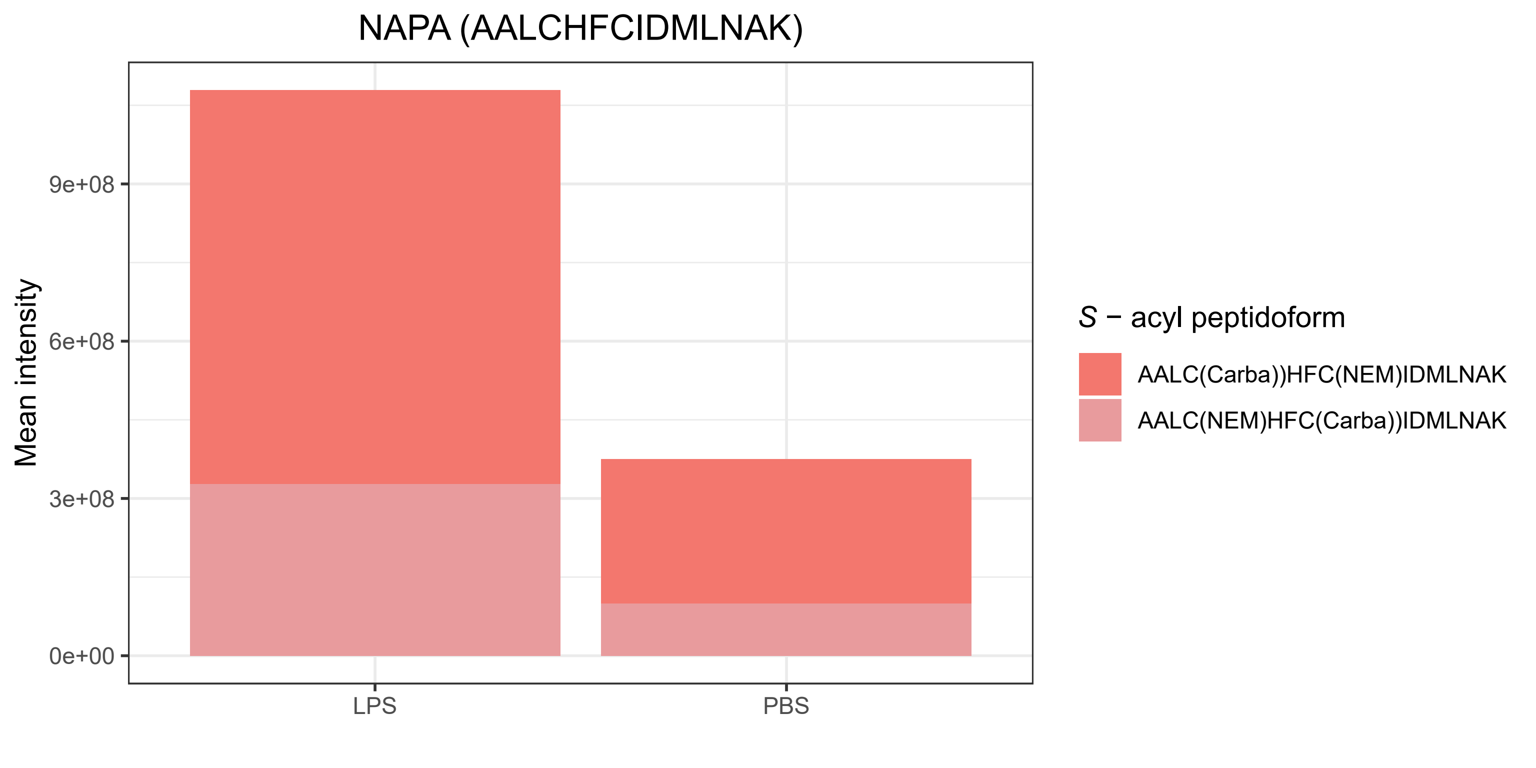

NAPA (AALCHFCIDMLNAK)
Mean intensity
9e+08
6e+08
3e+08
0e+00
LPS
PBS
S − acyl peptidoform
AALC(Carba))HFC(NEM)IDMLNAK
AALC(NEM)HFC(Carba))IDMLNAK

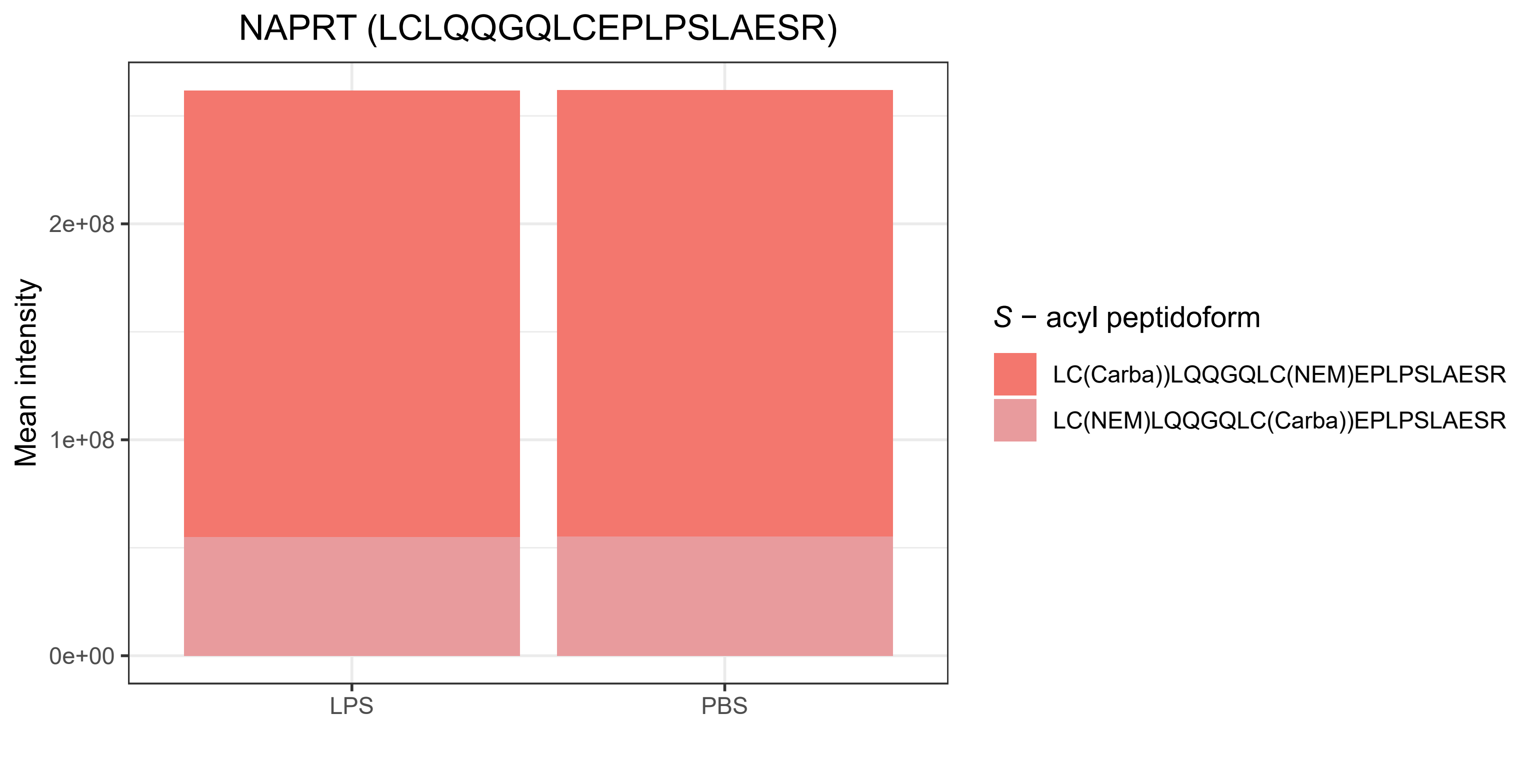

NAPRT (LCLQQGQLCEPLPSLAESR)
Mean intensity
2e+08
1e+08
0e+00
LPS
PBS
S − acyl peptidoform
LC(Carba))LQQGQLC(NEM)EPLPSLAESR
LC(NEM)LQQGQLC(Carba))EPLPSLAESR

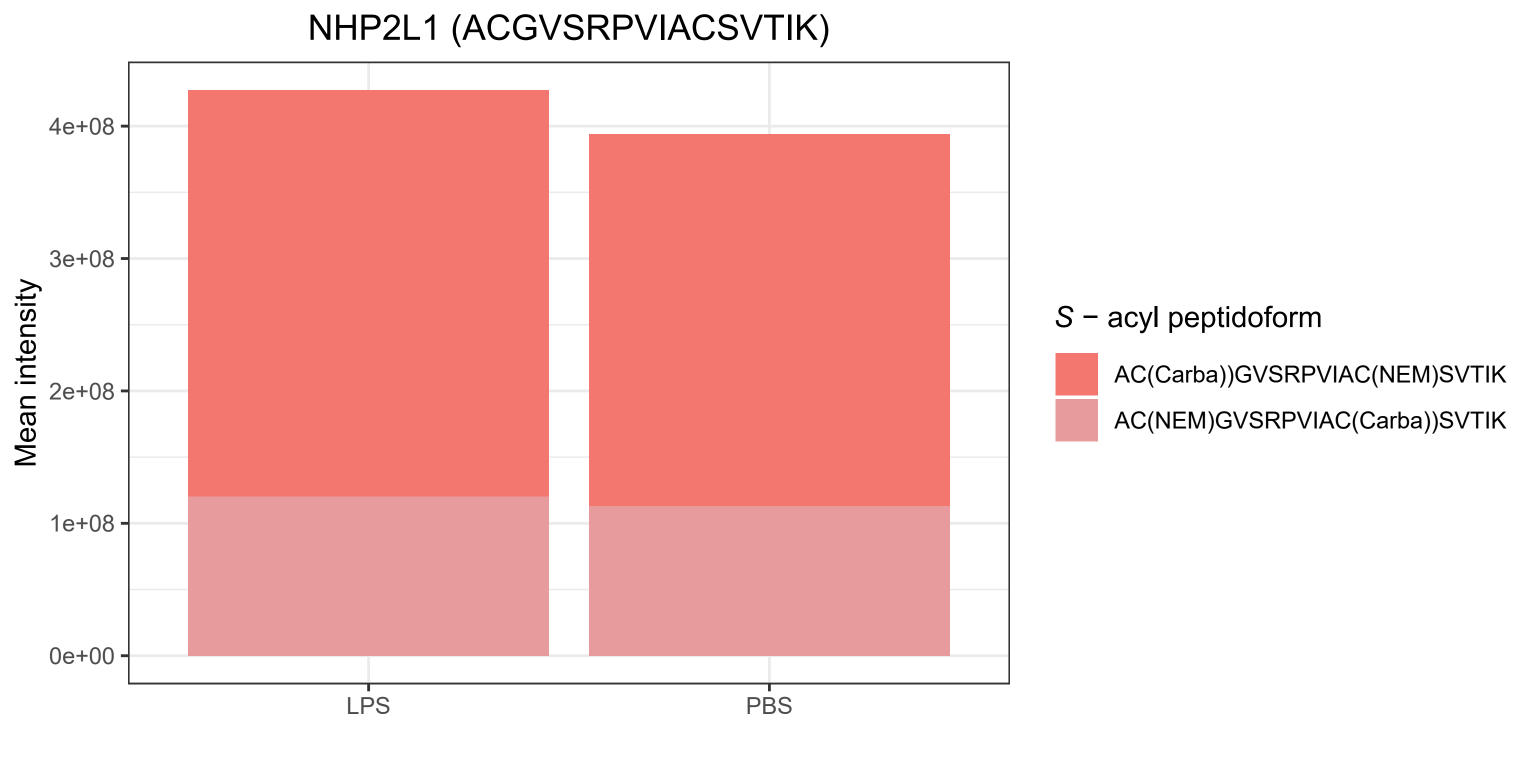
NHP2L1 (ACGVSRPVIACSVTIK)
Mean intensity
4e+08
3e+08
2e+08
1e+08
0e+00
LPS
PBS
S − acyl peptidoform
AC(Carba))GVSRPVIAC(NEM)SVTIK
AC(NEM)GVSRPVIAC(Carba))SVTIK

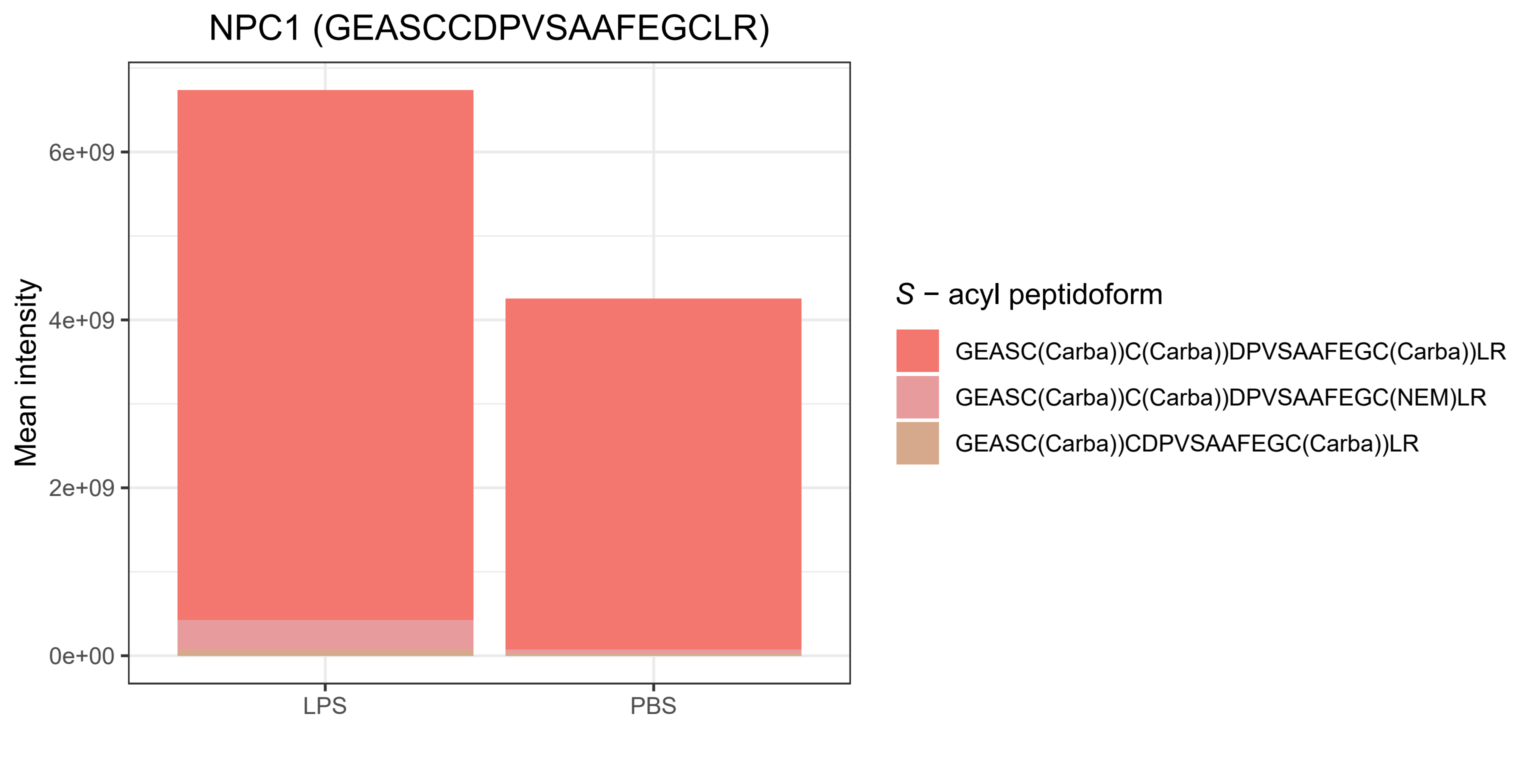
NPC1 (GEASCCDPVSAAFEGCLR)
Mean intensity
6e+09
4e+09
2e+09
0e+00
LPS
PBS
S − acyl peptidoform
GEASC(Carba))C(Carba))DPVSAAFEGC(Carba))LR
GEASC(Carba))C(Carba))DPVSAAFEGC(NEM)LR
GEASC(Carba))CDPVSAAFEGC(Carba))LR

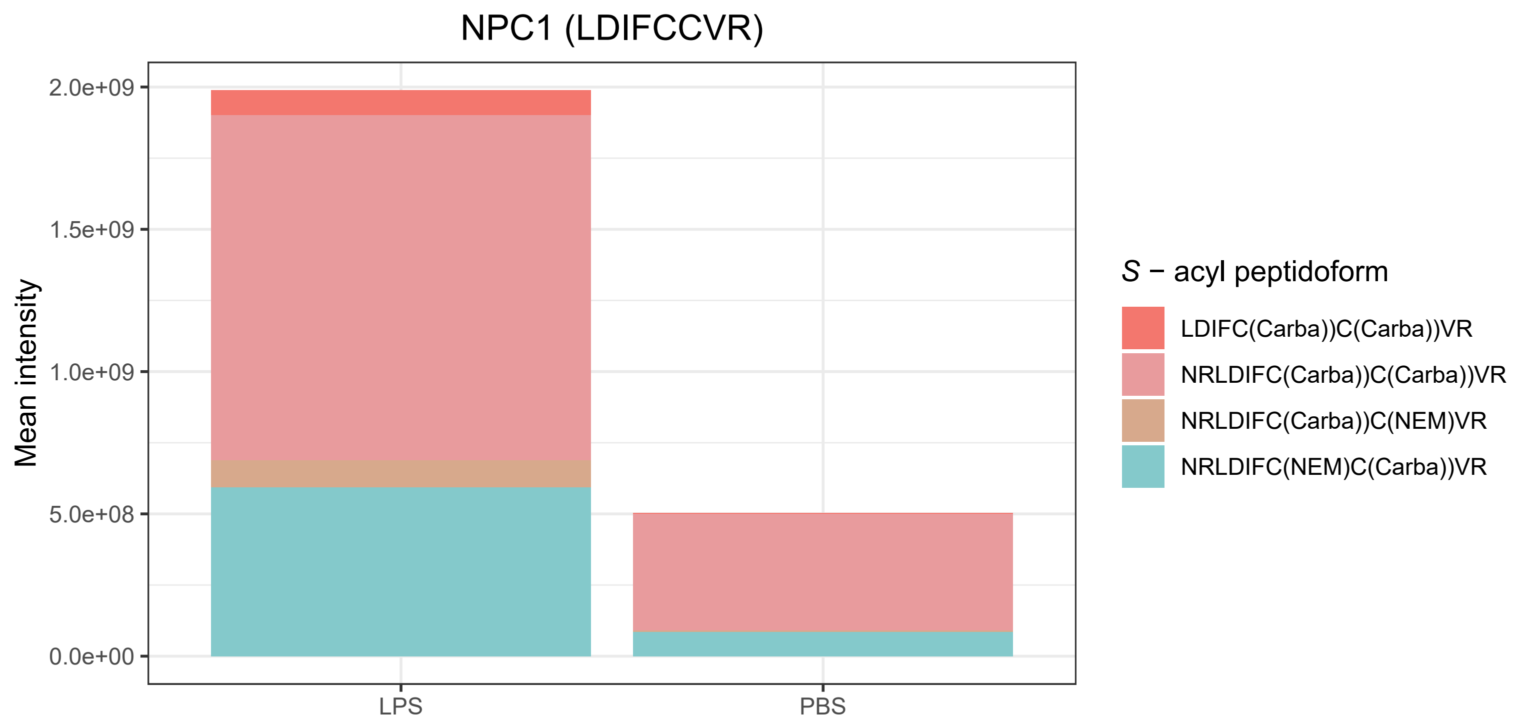
NPC1 (LDIFCCVR)
Mean intensity
2.0e+09
1.5e+09
1.0e+09
5.0e+08
0.0e+00
LPS
PBS
S − acyl peptidoform
LDIFC(Carba))C(Carba))VR
NRLDIFC(Carba))C(Carba))VR
NRLDIFC(Carba))C(NEM)VR
NRLDIFC(NEM)C(Carba))VR

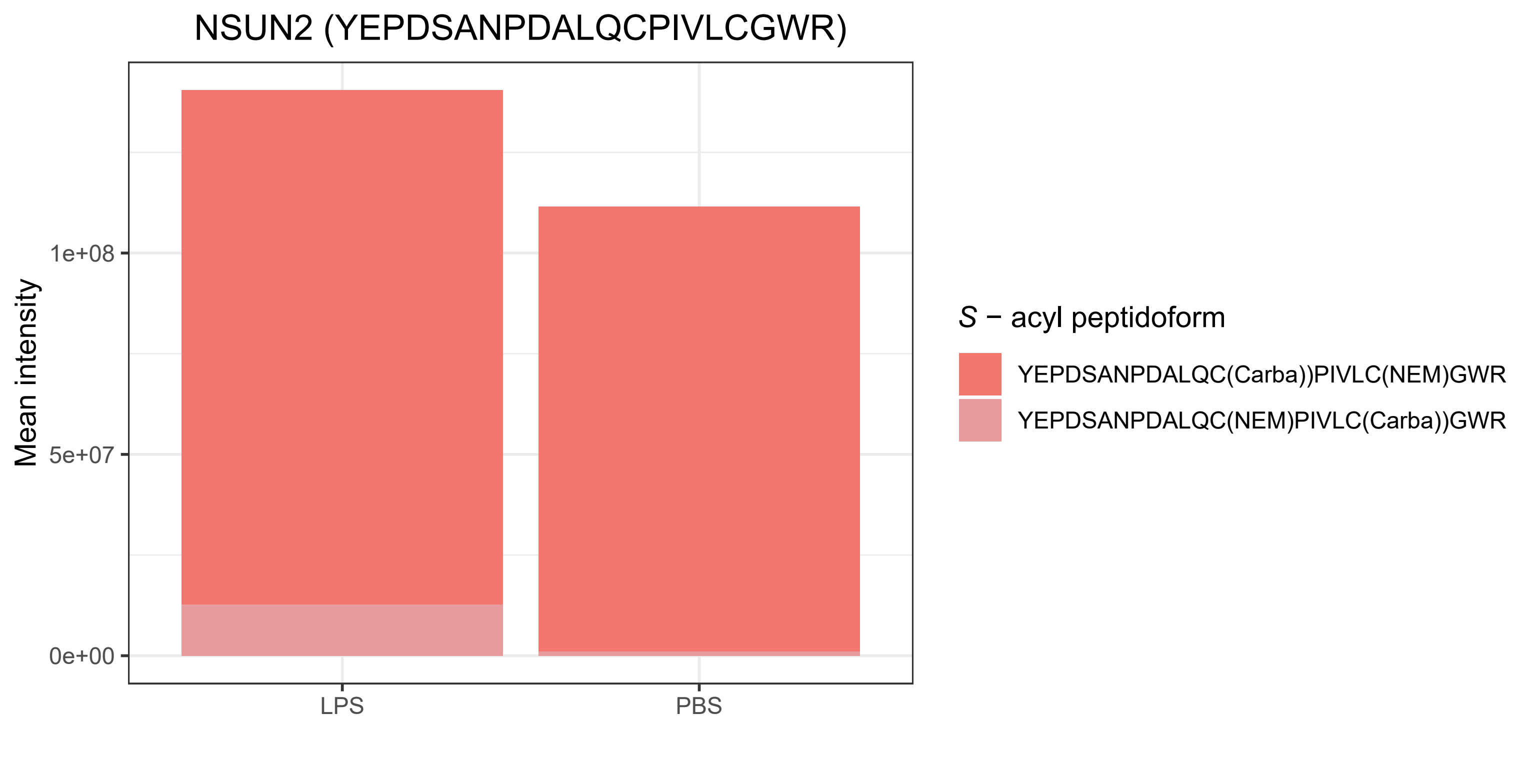

NSUN2 (YEPDSANPDALQCPIVLCGWR)
Mean intensity
1e+08
5e+07
0e+00
LPS
PBS
S − acyl peptidoform
YEPDSANPDALQC(Carba))PIVLC(NEM)GWR
YEPDSANPDALQC(NEM)PIVLC(Carba))GWR

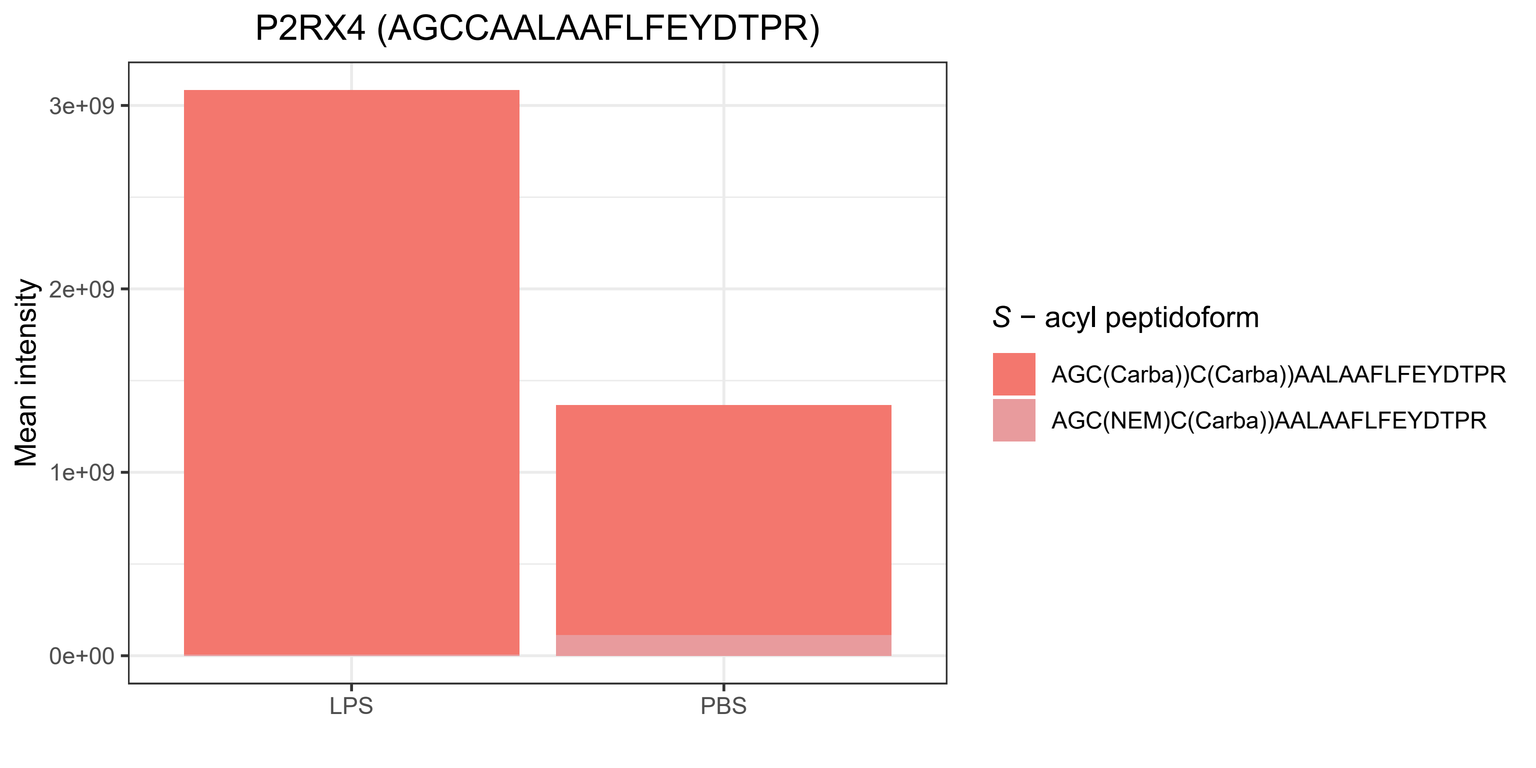
P2RX4 (AGCCAALAAFLFEYDTPR)
Mean intensity
3e+09
2e+09
1e+09
0e+00
LPS
PBS
S − acyl peptidoform
AGC(Carba))C(Carba))AALAAFLFEYDTPR
AGC(NEM)C(Carba))AALAAFLFEYDTPR

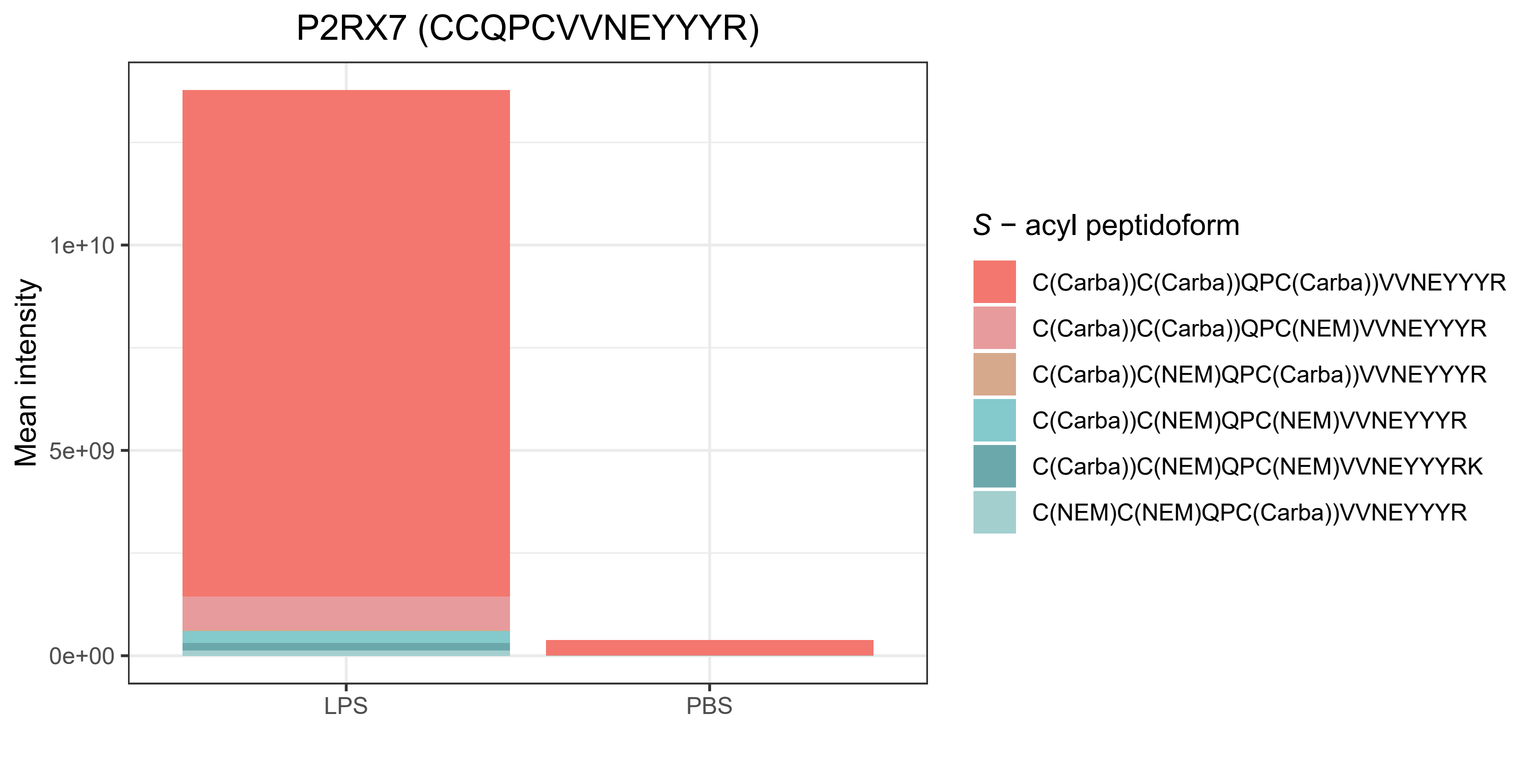

P2RX7 (CCQPCVVNEYYYR)
Mean intensity
1e+10
5e+09
0e+00
LPS
PBS
S − acyl peptidoform
C(Carba))C(Carba))QPC(Carba))VVNEYYYR
C(Carba))C(Carba))QPC(NEM)VVNEYYYR
C(Carba))C(NEM)QPC(Carba))VVNEYYYR
C(Carba))C(NEM)QPC(NEM)VVNEYYYR
C(Carba))C(NEM)QPC(NEM)VVNEYYYRK
C(NEM)C(NEM)QPC(Carba))VVNEYYYR

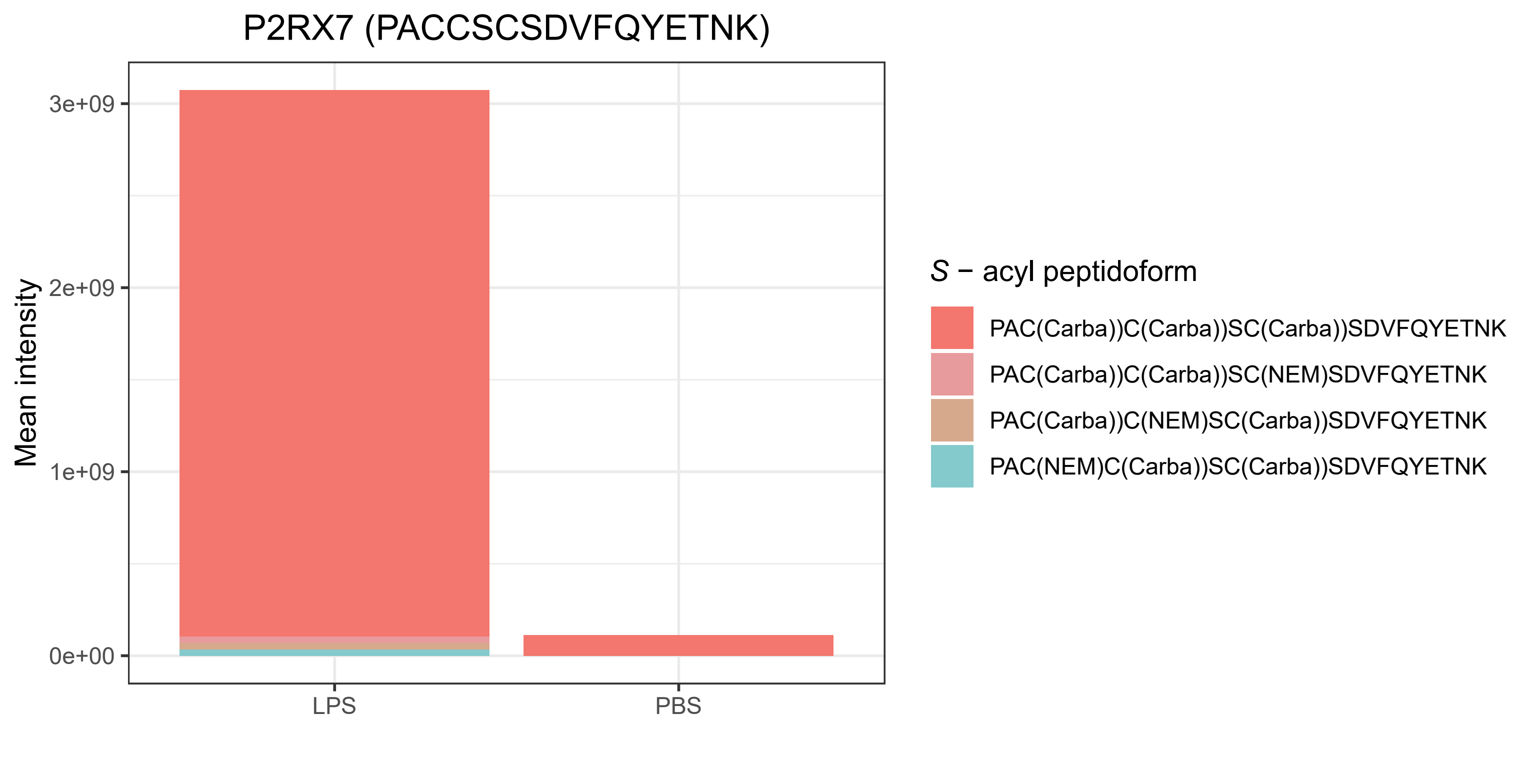
P2RX7 (PACCSCSDVFQYETNK)
Mean intensity
3e+09
2e+09
1e+09
0e+00
LPS
PBS
S − acyl peptidoform
PAC(Carba))C(Carba))SC(Carba))SDVFQYETNK
PAC(Carba))C(Carba))SC(NEM)SDVFQYETNK
PAC(Carba))C(NEM)SC(Carba))SDVFQYETNK
PAC(NEM)C(Carba))SC(Carba))SDVFQYETNK

PCNXL3 (EVEAITEGVEEDEGCCCCEPGHLPR)

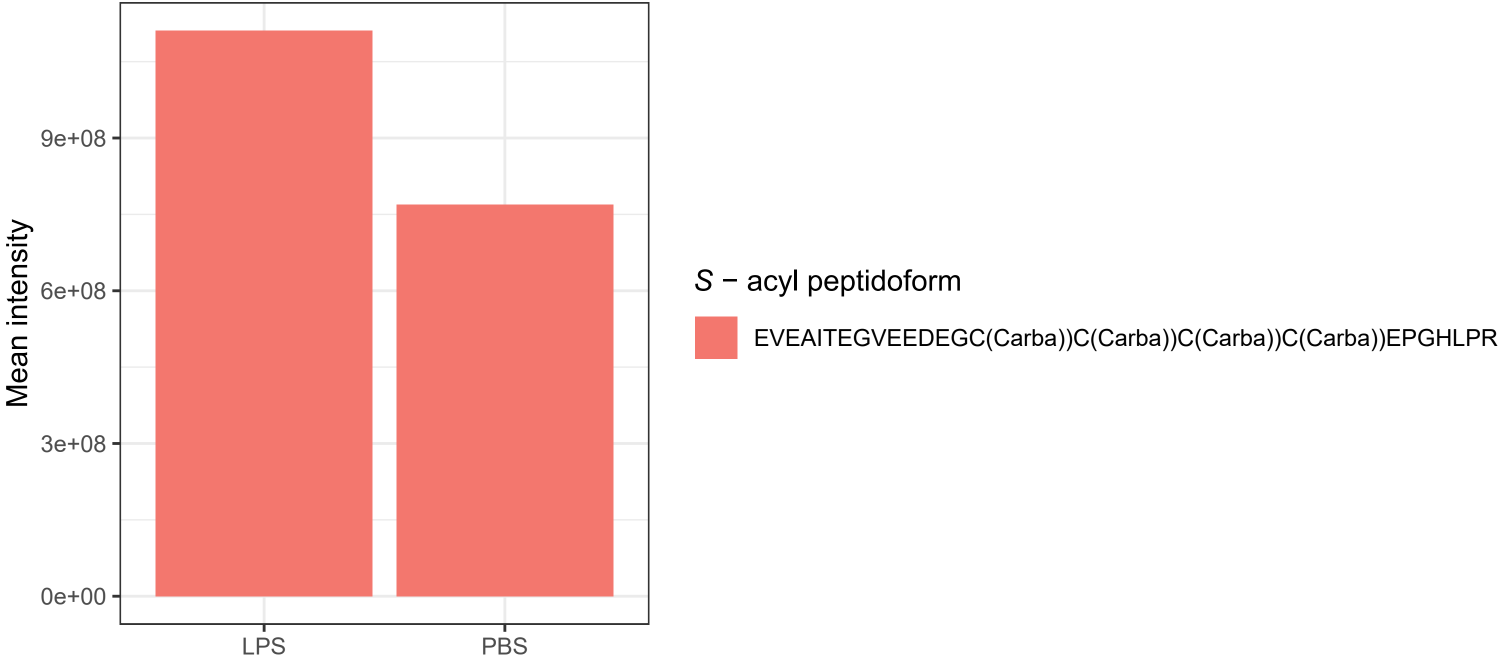

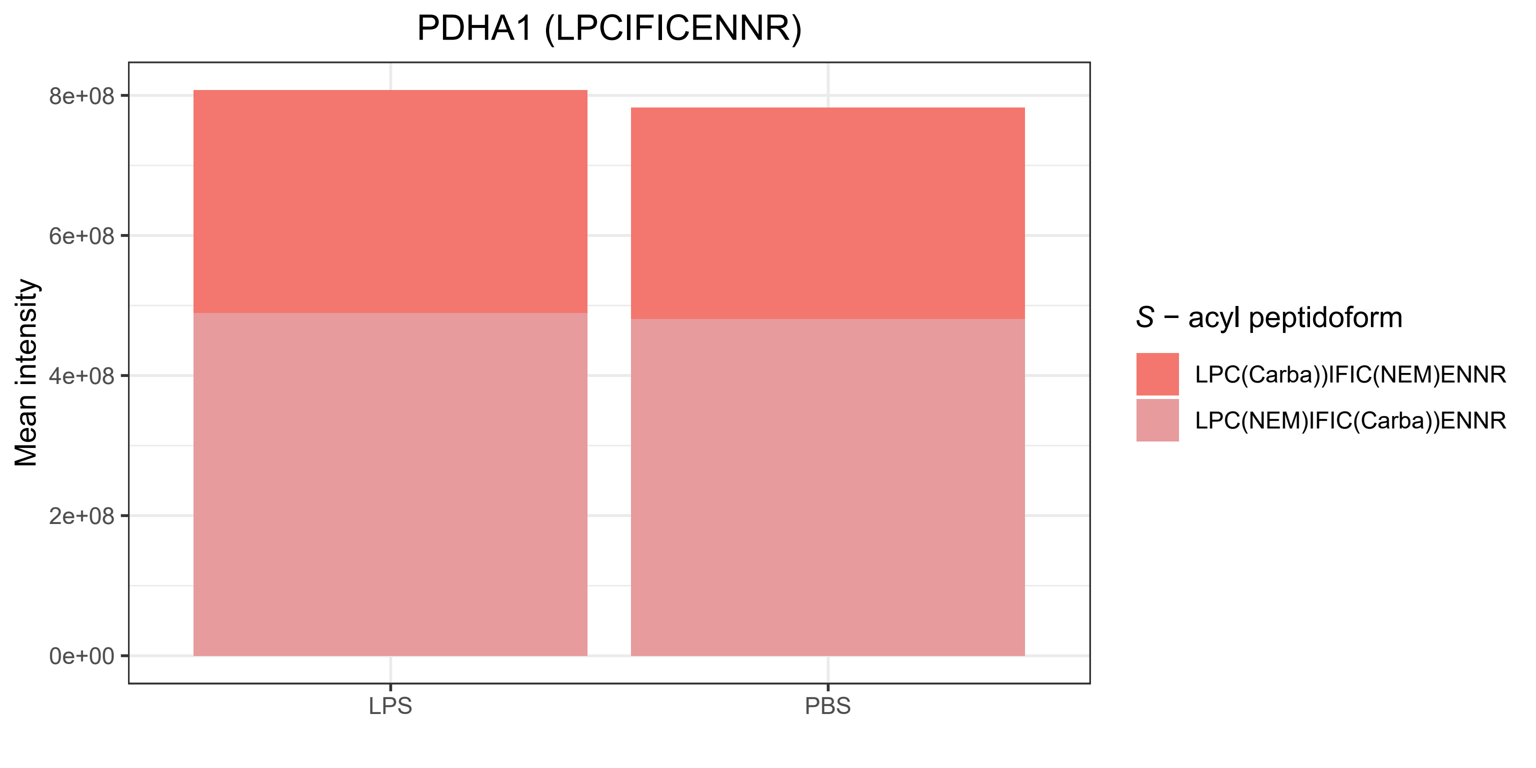
PDHA1 (LPCIFICENNR)
Mean intensity
8e+08
6e+08
4e+08
2e+08
0e+00
LPS
PBS
S − acyl peptidoform
LPC(Carba))IFIC(NEM)ENNR
LPC(NEM)IFIC(Carba))ENNR

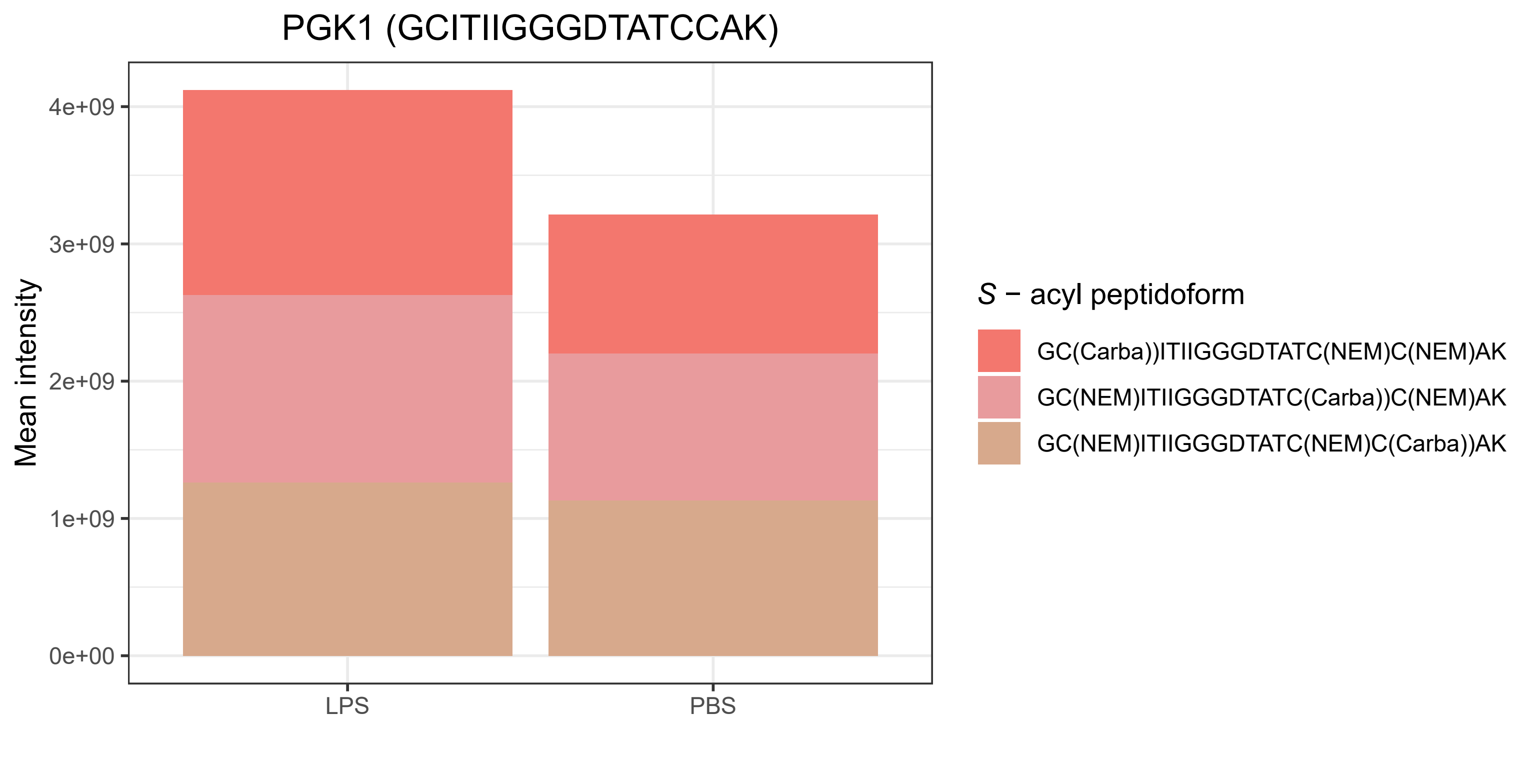

PGK1 (GCITIIGGGDTATCCAK)
Mean intensity
4e+09
3e+09
2e+09
1e+09
0e+00
LPS
PBS
S − acyl peptidoform
GC(Carba))ITIIGGGDTATC(NEM)C(NEM)AK
GC(NEM)ITIIGGGDTATC(Carba))C(NEM)AK
GC(NEM)ITIIGGGDTATC(NEM)C(Carba))AK

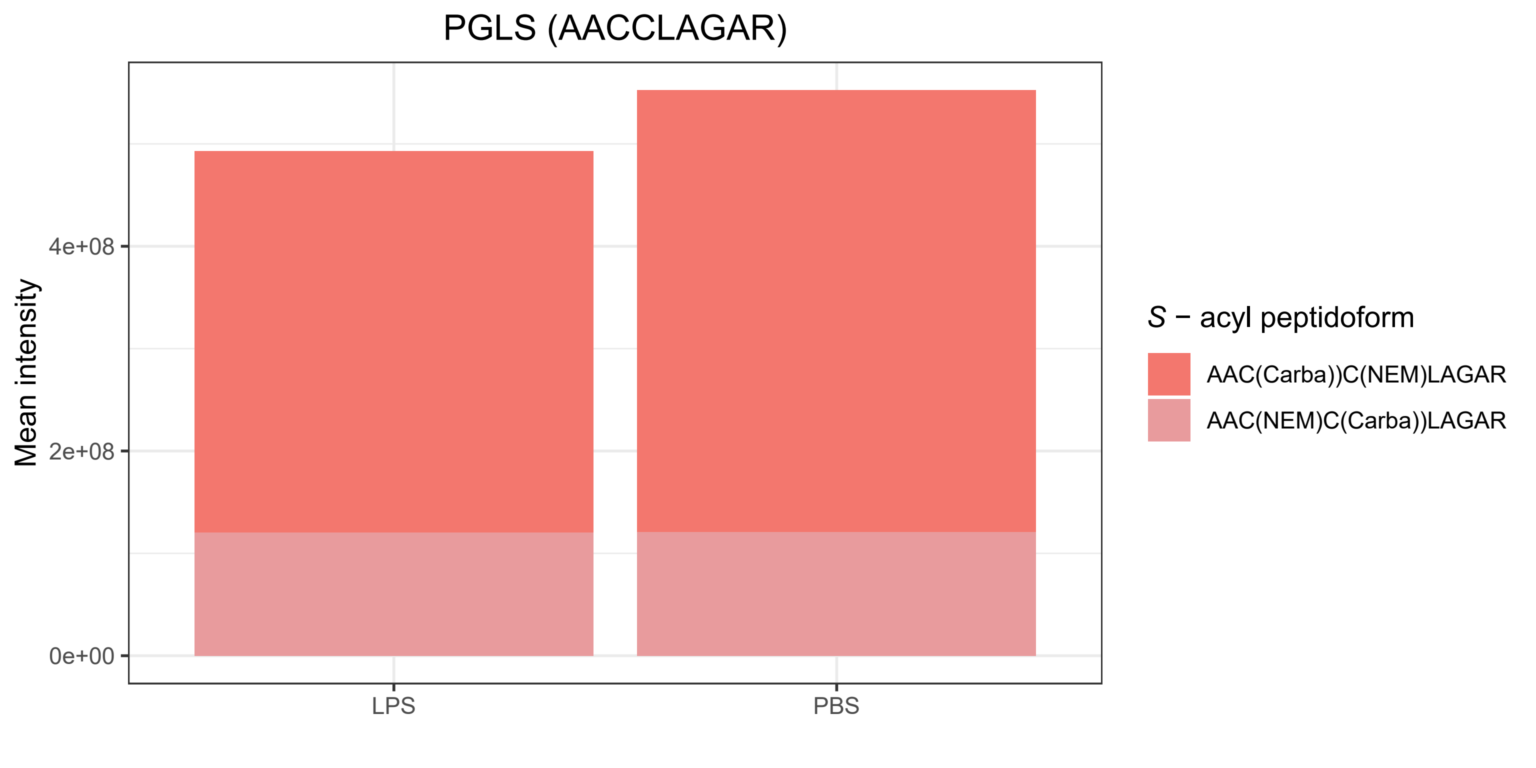

PGLS (AACCLAGAR)
Mean intensity
4e+08
2e+08
0e+00
LPS
PBS
S − acyl peptidoform
AAC(Carba))C(NEM)LAGAR
AAC(NEM)C(Carba))LAGAR

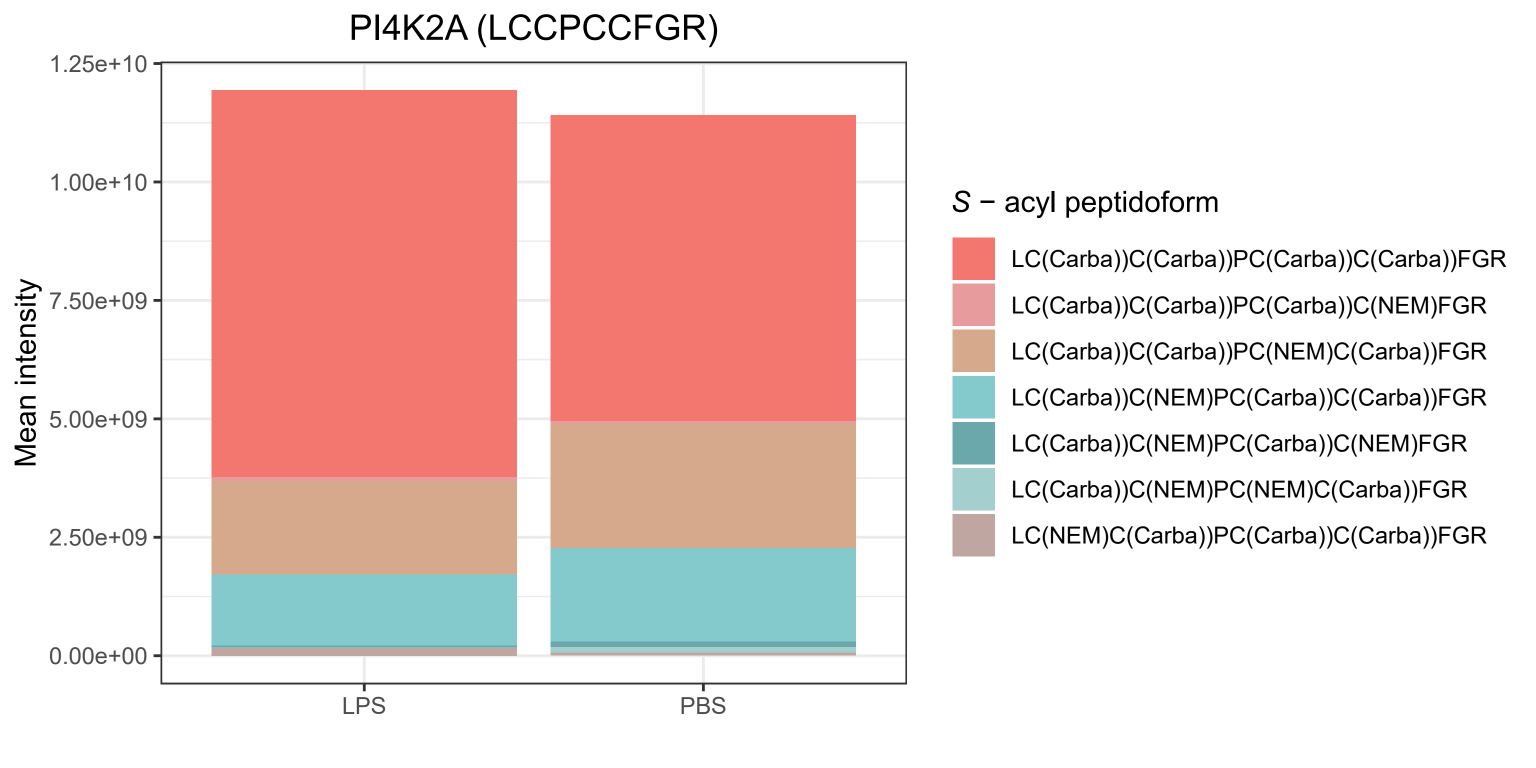
PI4K2A (LCCPCCFGR)
Mean intensity
1.25e+10
1.00e+10
7.50e+09
5.00e+09
2.50e+09
0.00e+00
LPS
PBS
S − acyl peptidoform
LC(Carba))C(Carba))PC(Carba))C(Carba))FGR
LC(Carba))C(Carba))PC(Carba))C(NEM)FGR
LC(Carba))C(Carba))PC(NEM)C(Carba))FGR
LC(Carba))C(NEM)PC(Carba))C(Carba))FGR
LC(Carba))C(NEM)PC(Carba))C(NEM)FGR
LC(Carba))C(NEM)PC(NEM)C(Carba))FGR
LC(NEM)C(Carba))PC(Carba))C(Carba))FGR

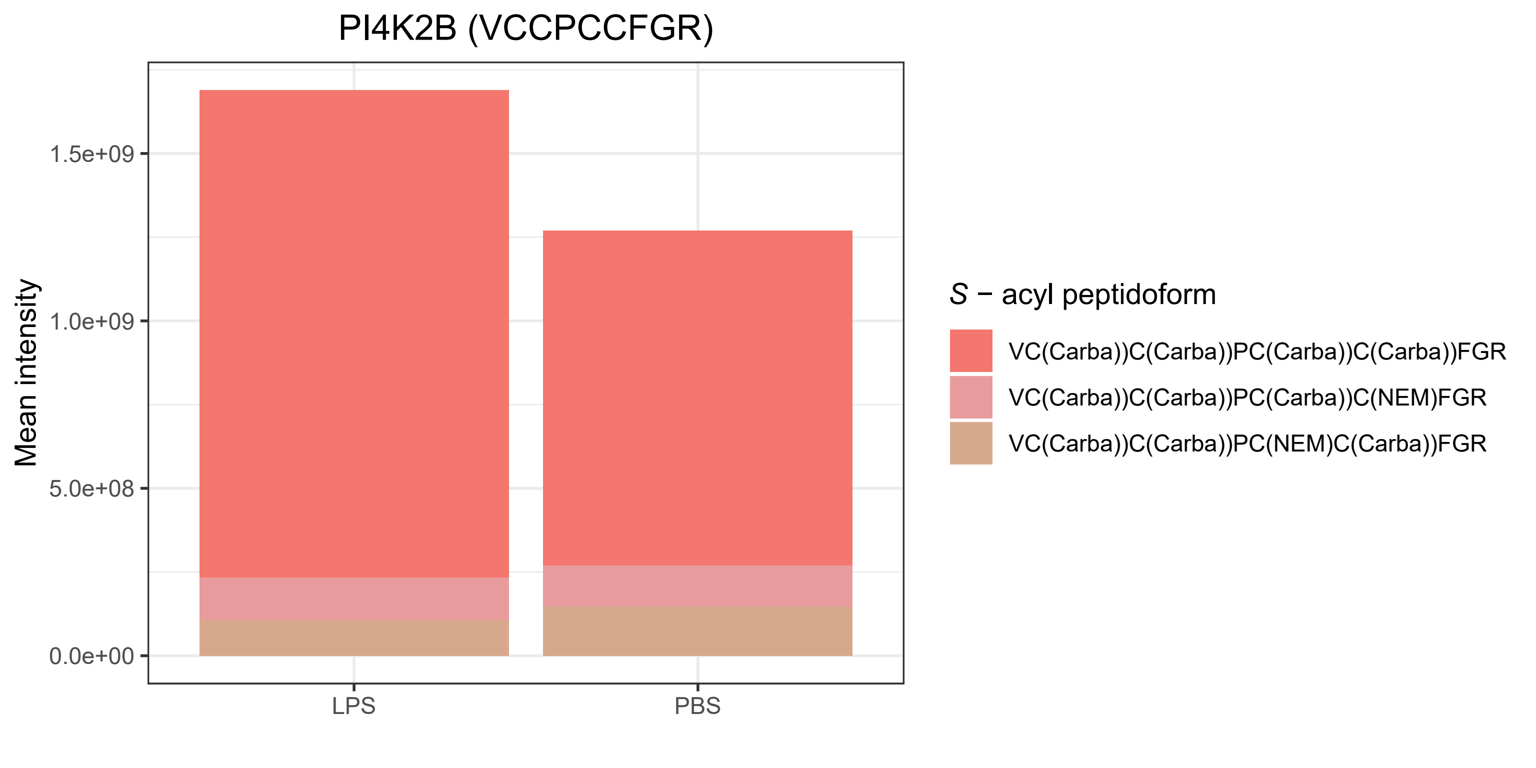

PI4K2B (VCCPCCFGR)
Mean intensity
1.5e+09
1.0e+09
5.0e+08
0.0e+00
LPS
PBS
S − acyl peptidoform
VC(Carba))C(Carba))PC(Carba))C(Carba))FGR
VC(Carba))C(Carba))PC(Carba))C(NEM)FGR
VC(Carba))C(Carba))PC(NEM)C(Carba))FGR

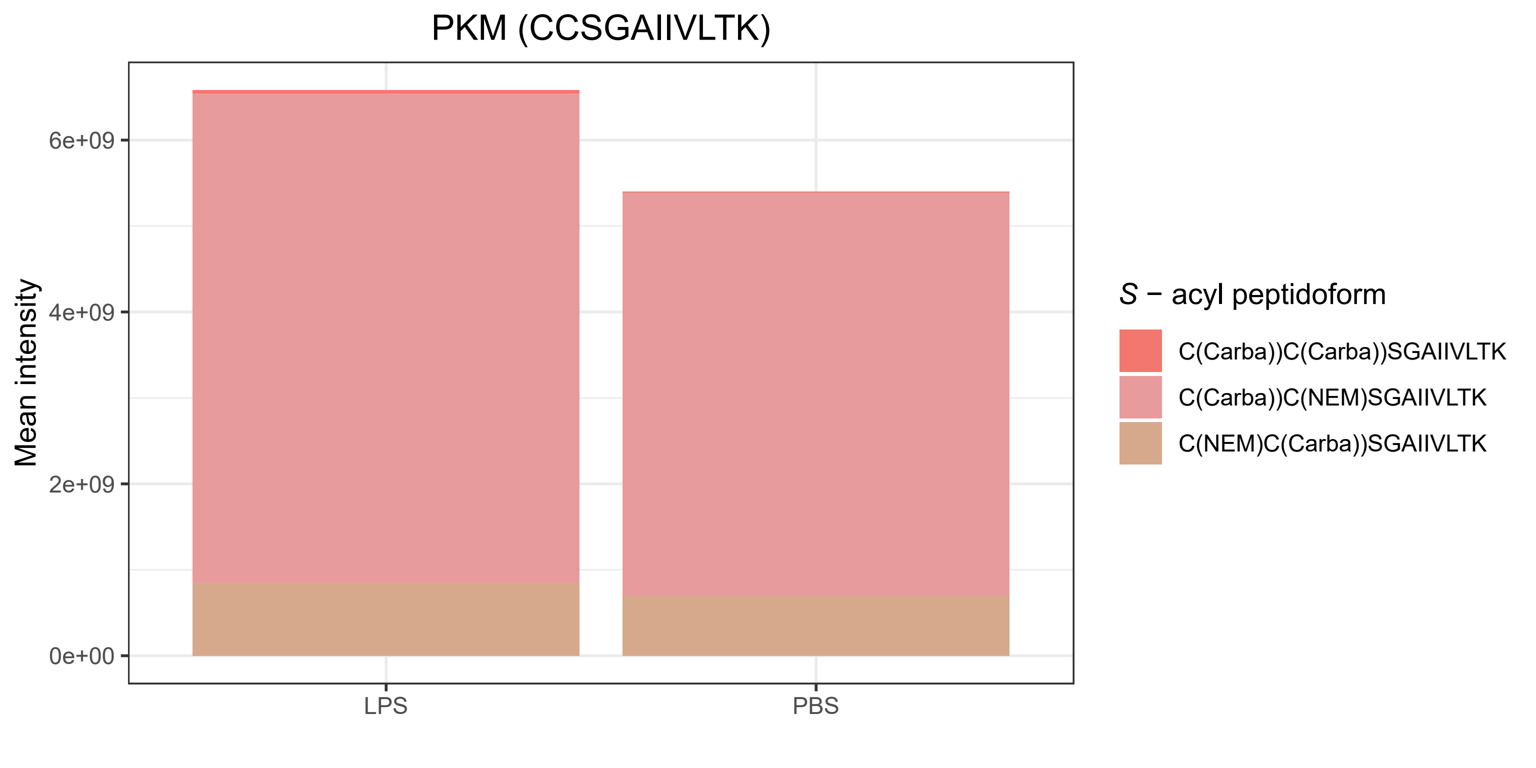
PKM (CCSGAIIVLTK)
Mean intensity
6e+09
4e+09
2e+09
0e+00
LPS
PBS
S − acyl peptidoform
C(Carba))C(Carba))SGAIIVLTK
C(Carba))C(NEM)SGAIIVLTK
C(NEM)C(Carba))SGAIIVLTK

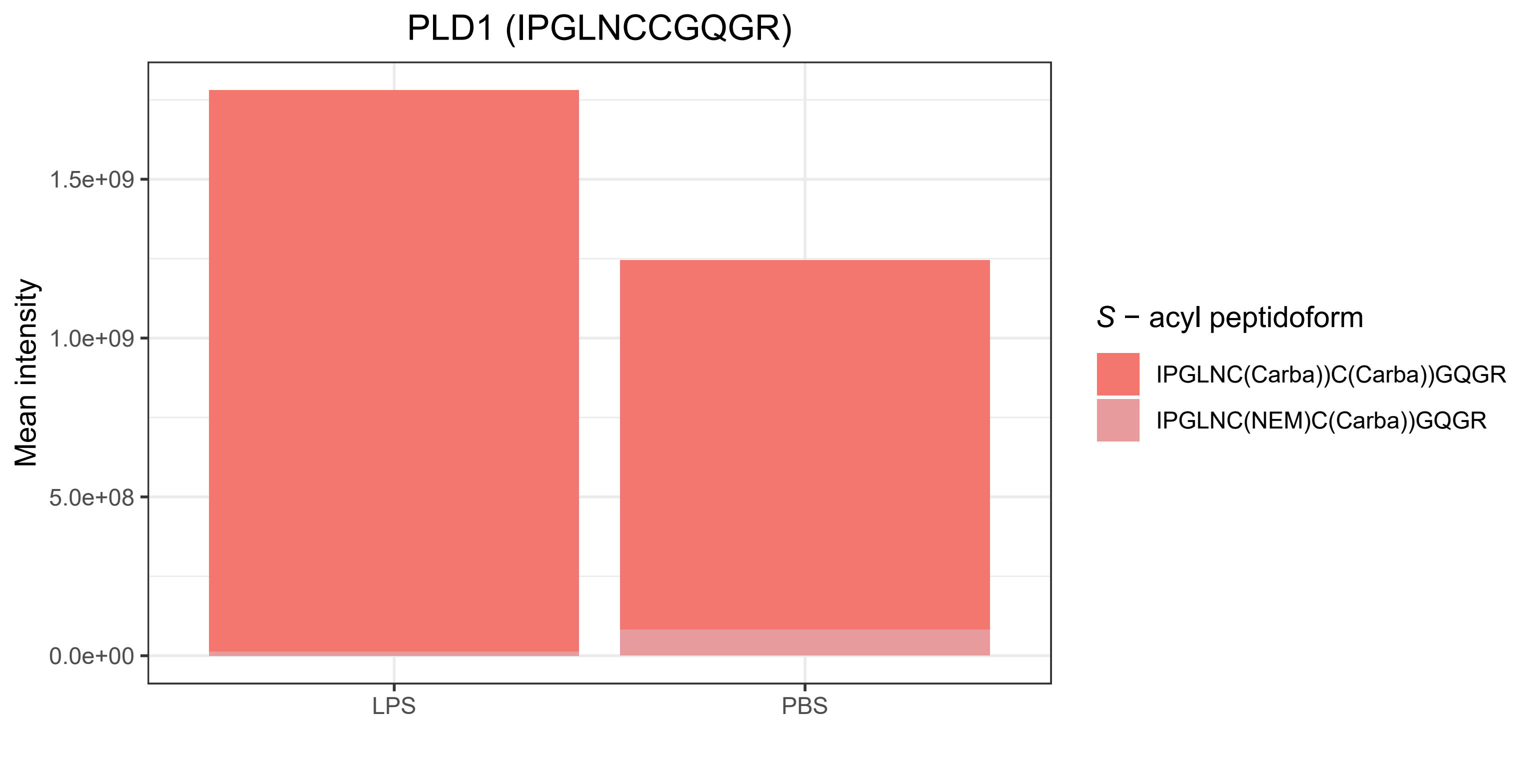

PLD1 (IPGLNCCGQGR)
Mean intensity
1.5e+09
1.0e+09
5.0e+08
0.0e+00
LPS
PBS
S − acyl peptidoform
IPGLNC(Carba))C(Carba))GQGR
IPGLNC(NEM)C(Carba))GQGR

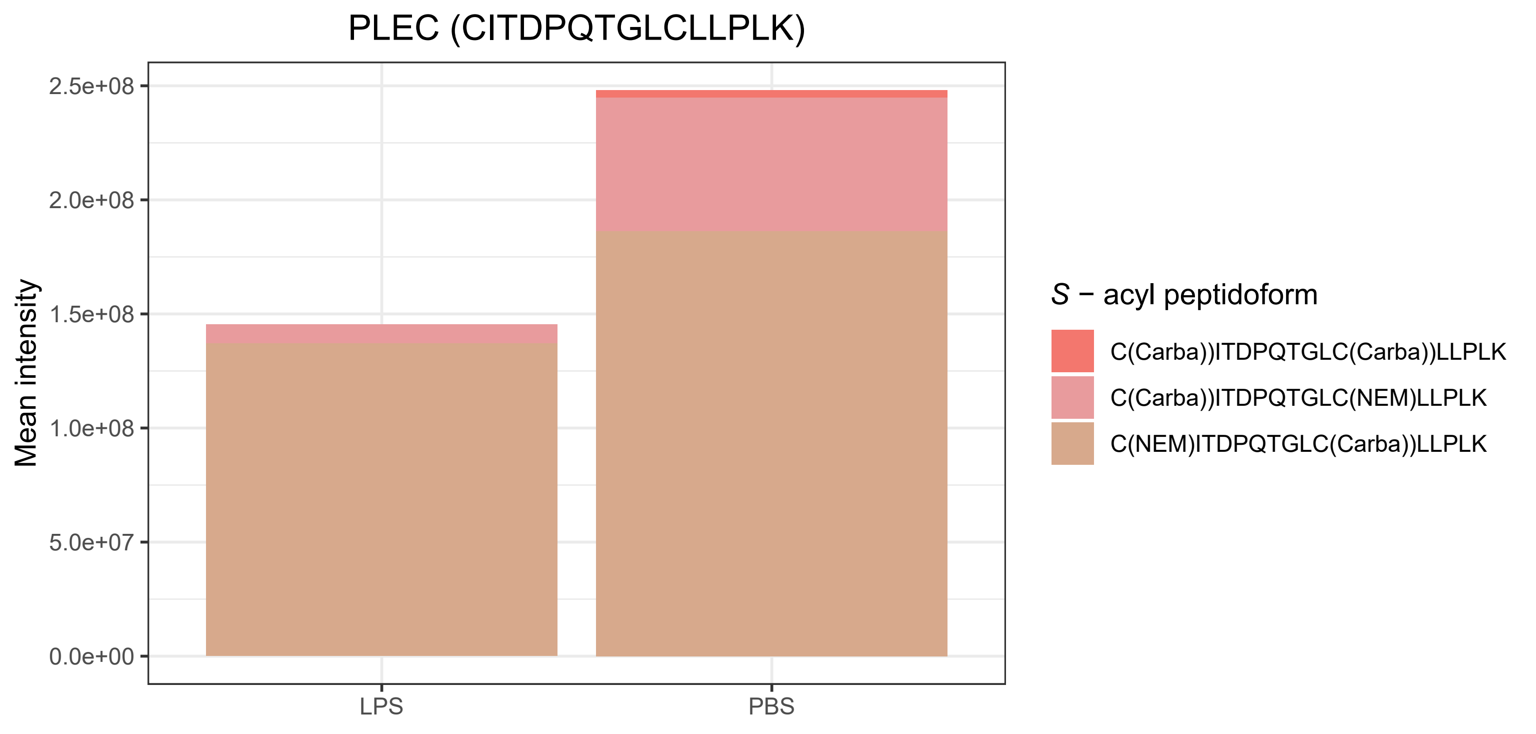
PLEC (CITDPQTGLCLLPLK)
Mean intensity
2.5e+08
2.0e+08
1.5e+08
1.0e+08
5.0e+07
0.0e+00
LPS
PBS
S − acyl peptidoform
C(Carba))ITDPQTGLC(Carba))LLPLK
C(Carba))ITDPQTGLC(NEM)LLPLK
C(NEM)ITDPQTGLC(Carba))LLPLK

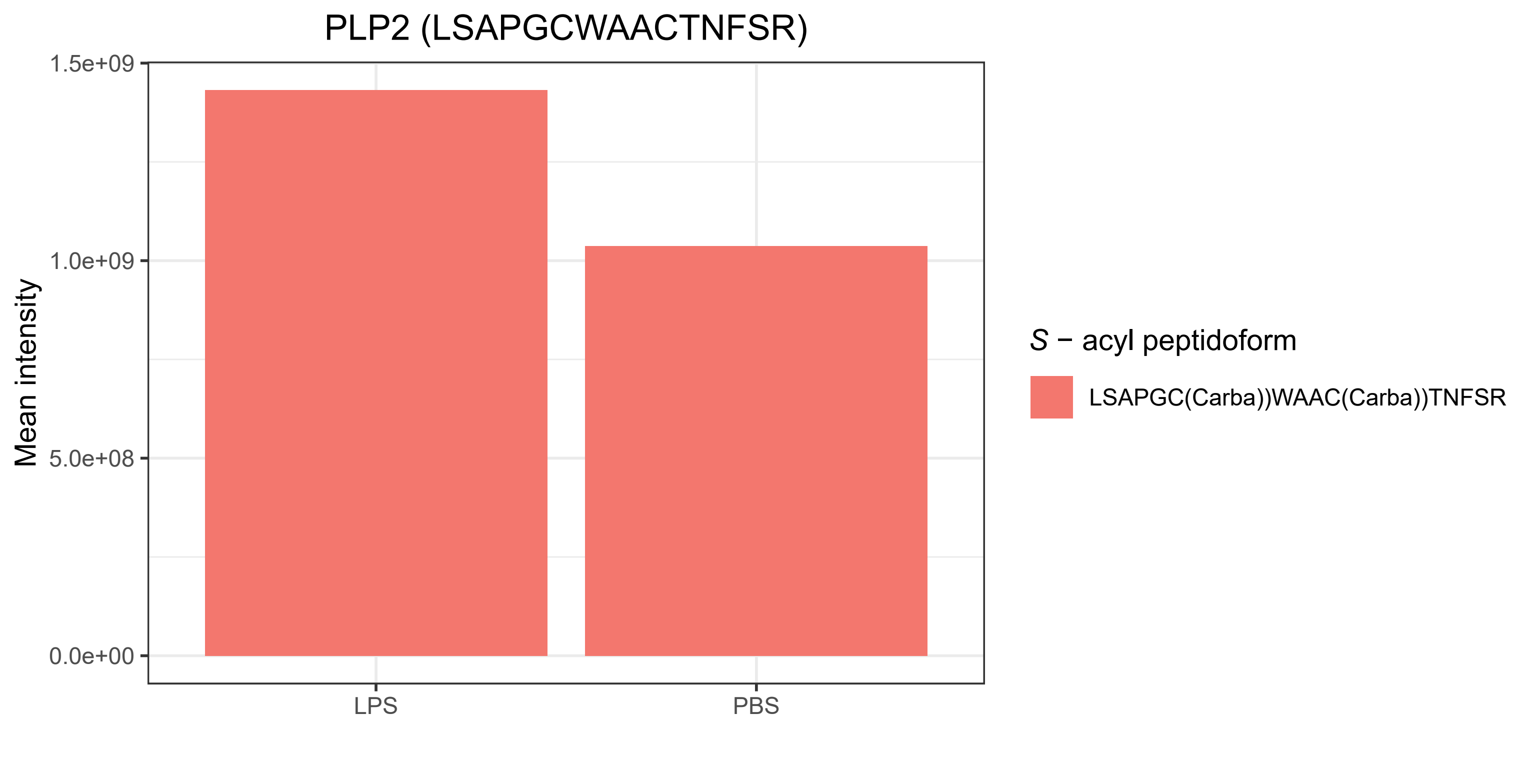
PLP2 (LSAPGCWAACTNFSR)
1.5e+09
1.0e+09
5.0e+08
0.0e+00
Mean intensity
LPS
PBS
S − acyl peptidoform
LSAPGC(Carba))WAAC(Carba))TNFSR

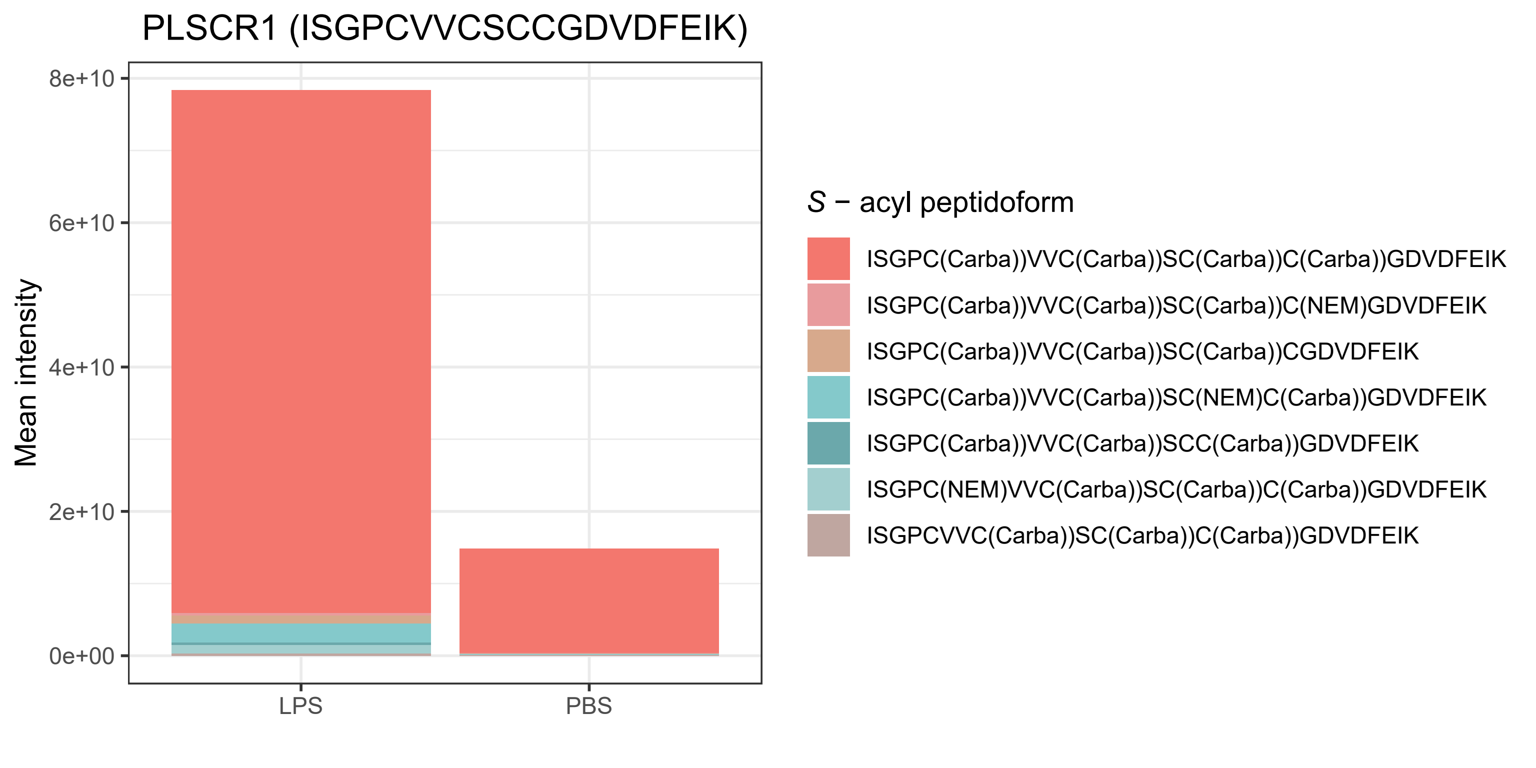

PLSCR1 (ISGPCVVCSCCGDVDFEIK)
Mean intensity
8e+10
6e+10
4e+10
2e+10
0e+00
LPS
PBS
S − acyl peptidoform
ISGPC(Carba))VVC(Carba))SC(Carba))C(Carba))GDVDFEIK
ISGPC(Carba))VVC(Carba))SC(Carba))C(NEM)GDVDFEIK
ISGPC(Carba))VVC(Carba))SC(Carba))CGDVDFEIK
ISGPC(Carba))VVC(Carba))SC(NEM)C(Carba))GDVDFEIK
ISGPC(Carba))VVC(Carba))SCC(Carba))GDVDFEIK
ISGPC(NEM)VVC(Carba))SC(Carba))C(Carba))GDVDFEIK
ISGPCVVC(Carba))SC(Carba))C(Carba))GDVDFEIK

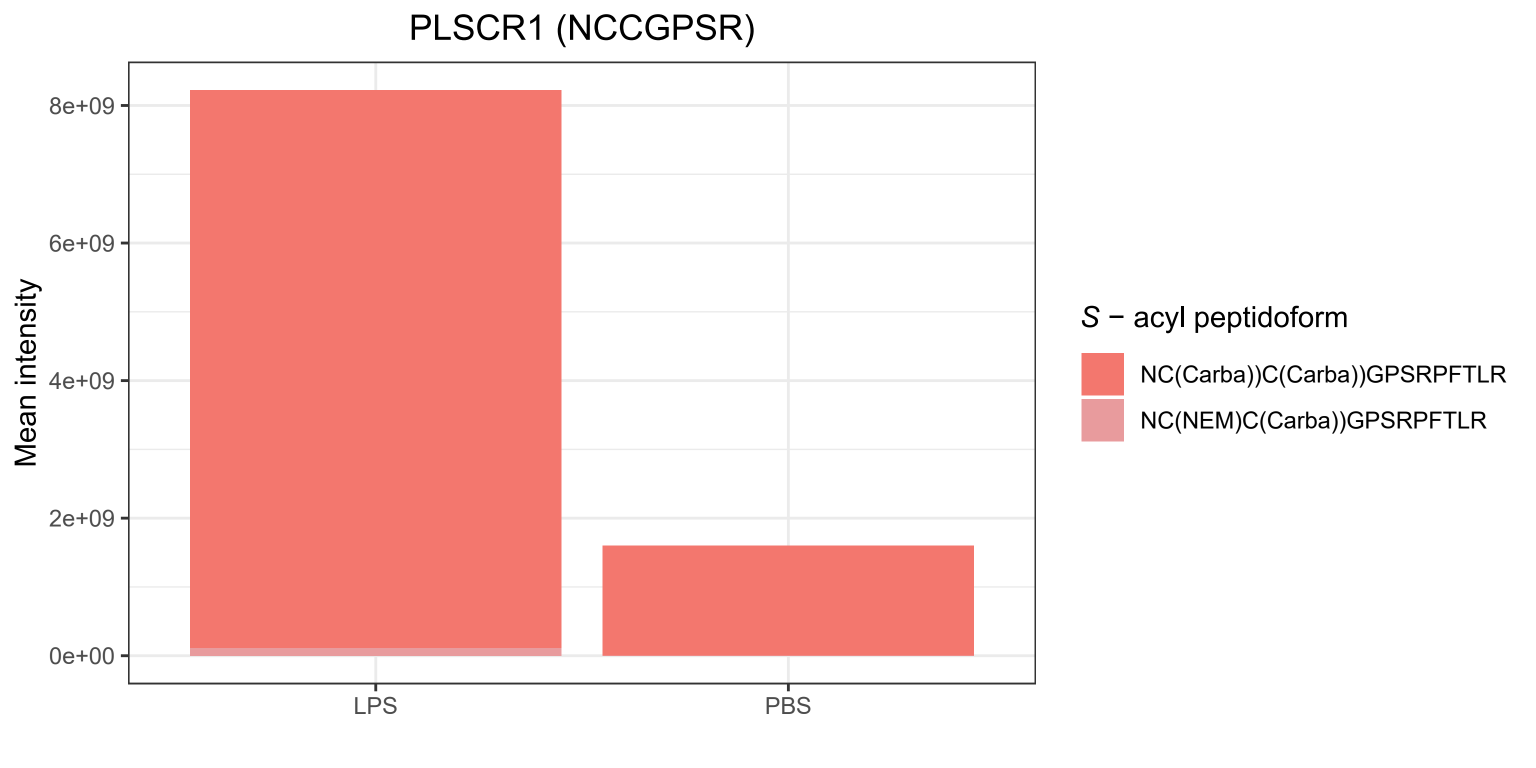

PLSCR1 (NCCGPSR)
Mean intensity
8e+09
6e+09
4e+09
2e+09
0e+00
LPS
PBS
S − acyl peptidoform
NC(Carba))C(Carba))GPSRPFTLR
NC(NEM)C(Carba))GPSRPFTLR

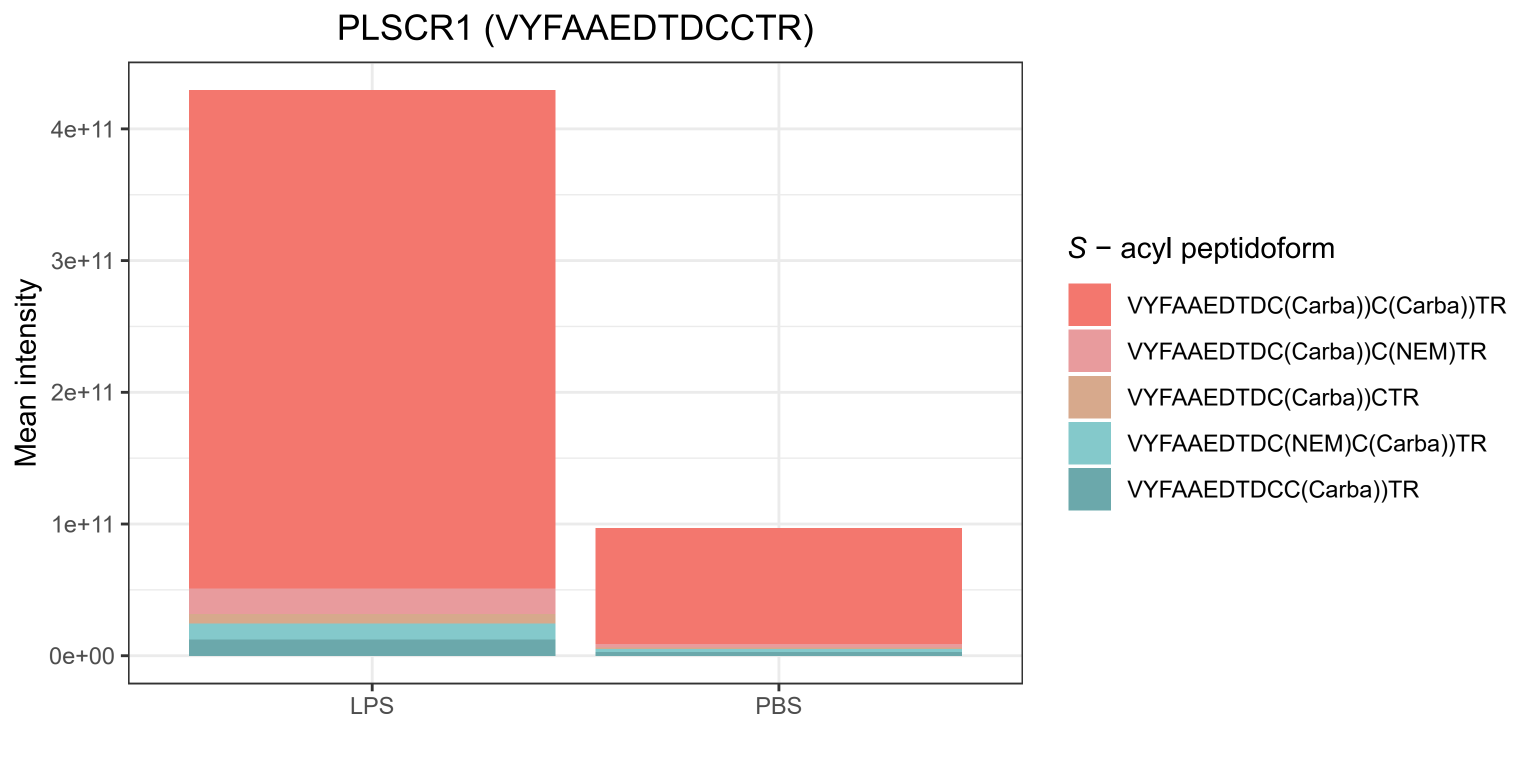

PLSCR1 (VYFAAEDTDCCTR)
Mean intensity
4e+11
3e+11
2e+11
1e+11
0e+00
LPS
PBS
S − acyl peptidoform
VYFAAEDTDC(Carba))C(Carba))TR
VYFAAEDTDC(Carba))C(NEM)TR
VYFAAEDTDC(Carba))CTR
VYFAAEDTDC(NEM)C(Carba))TR
VYFAAEDTDCC(Carba))TR

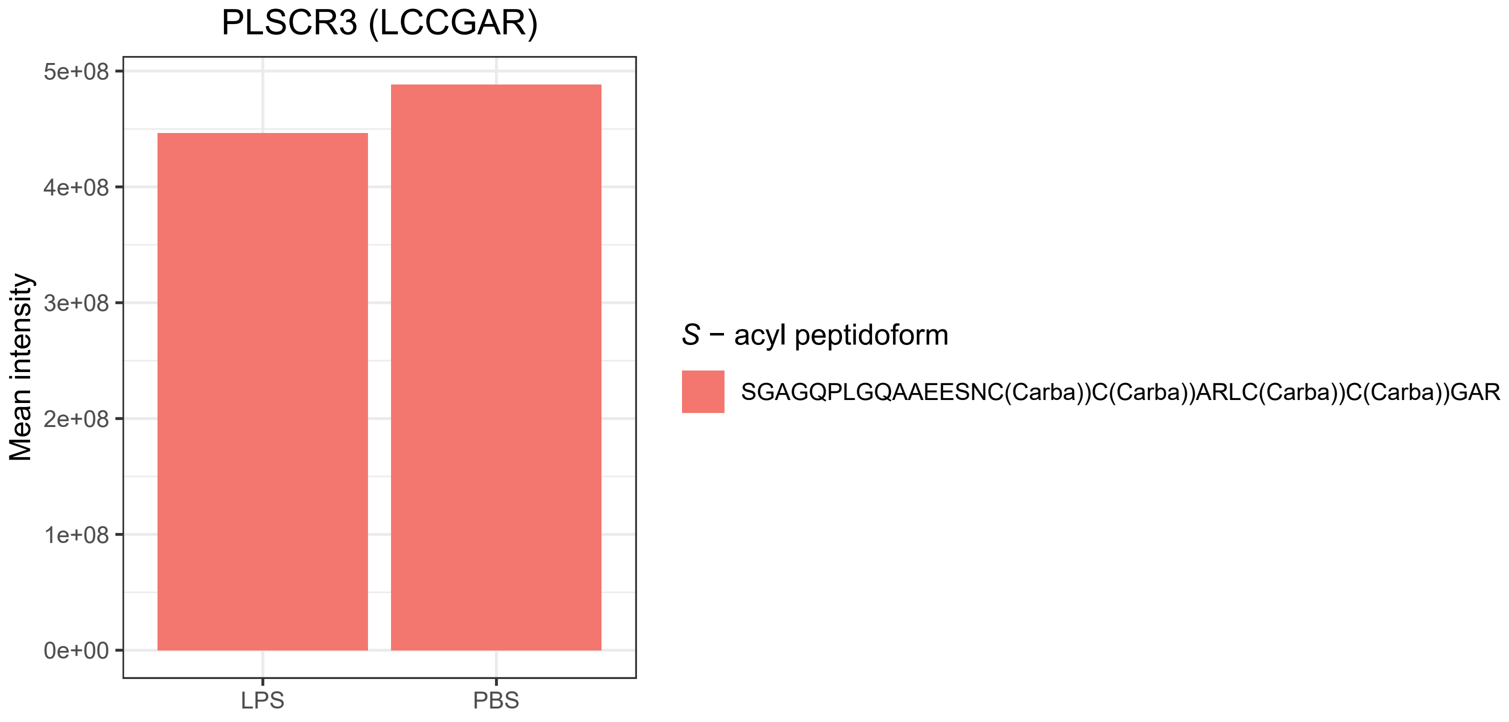

PLSCR3 (LCCGAR)
Mean intensity
5e+08
4e+08
3e+08
2e+08
1e+08
0e+00
LPS
PBS
S − acyl peptidoform
SGAGQPLGQAAEESNC(Carba))C(Carba))ARLC(Carba))C(Carba))GAR

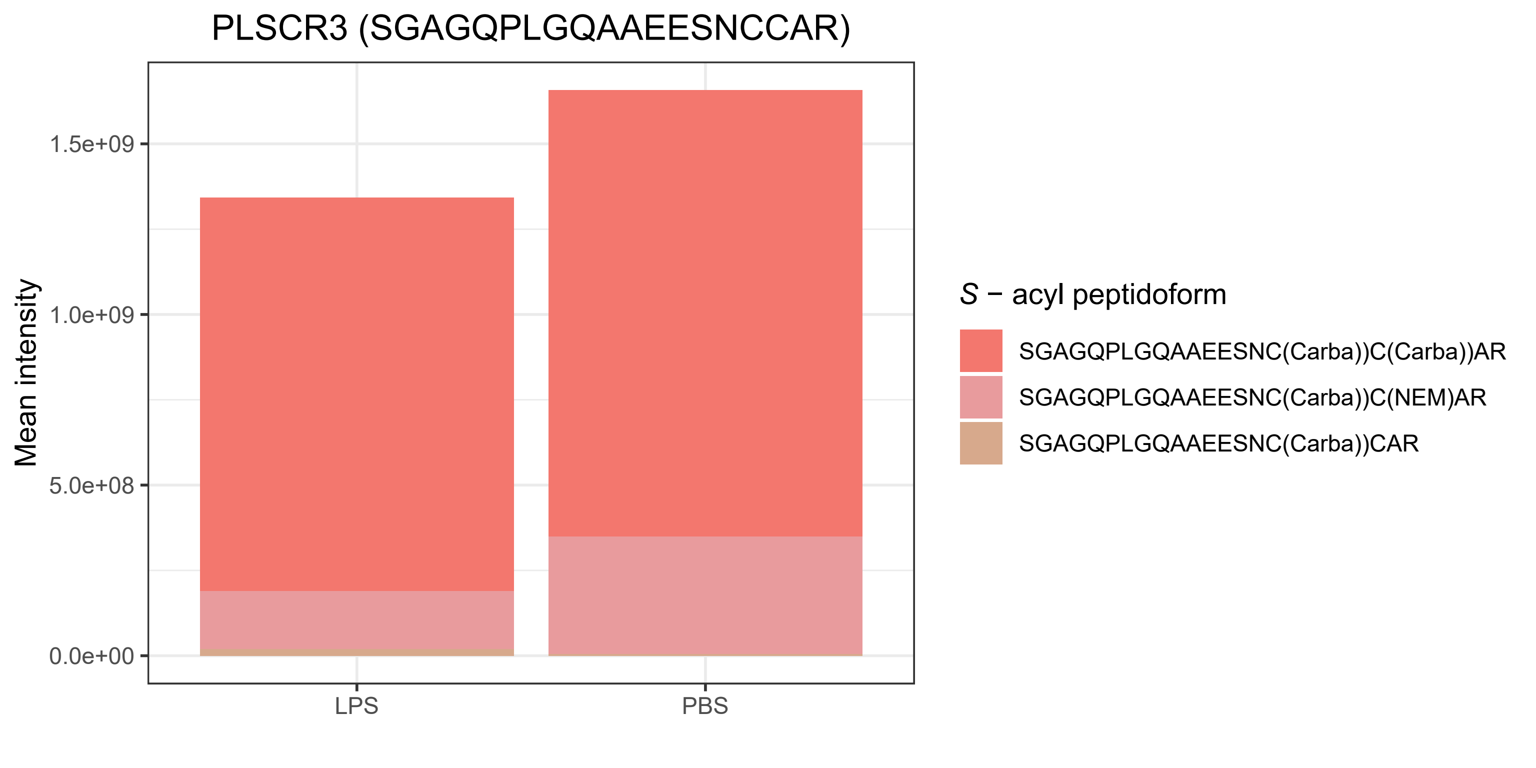

PLSCR3 (SGAGQPLGQAAEESNCCAR)
Mean intensity
1.5e+09
1.0e+09
5.0e+08
0.0e+00
LPS
PBS
S − acyl peptidoform
SGAGQPLGQAAEESNC(Carba))C(Carba))AR
SGAGQPLGQAAEESNC(Carba))C(NEM)AR
SGAGQPLGQAAEESNC(Carba))CAR

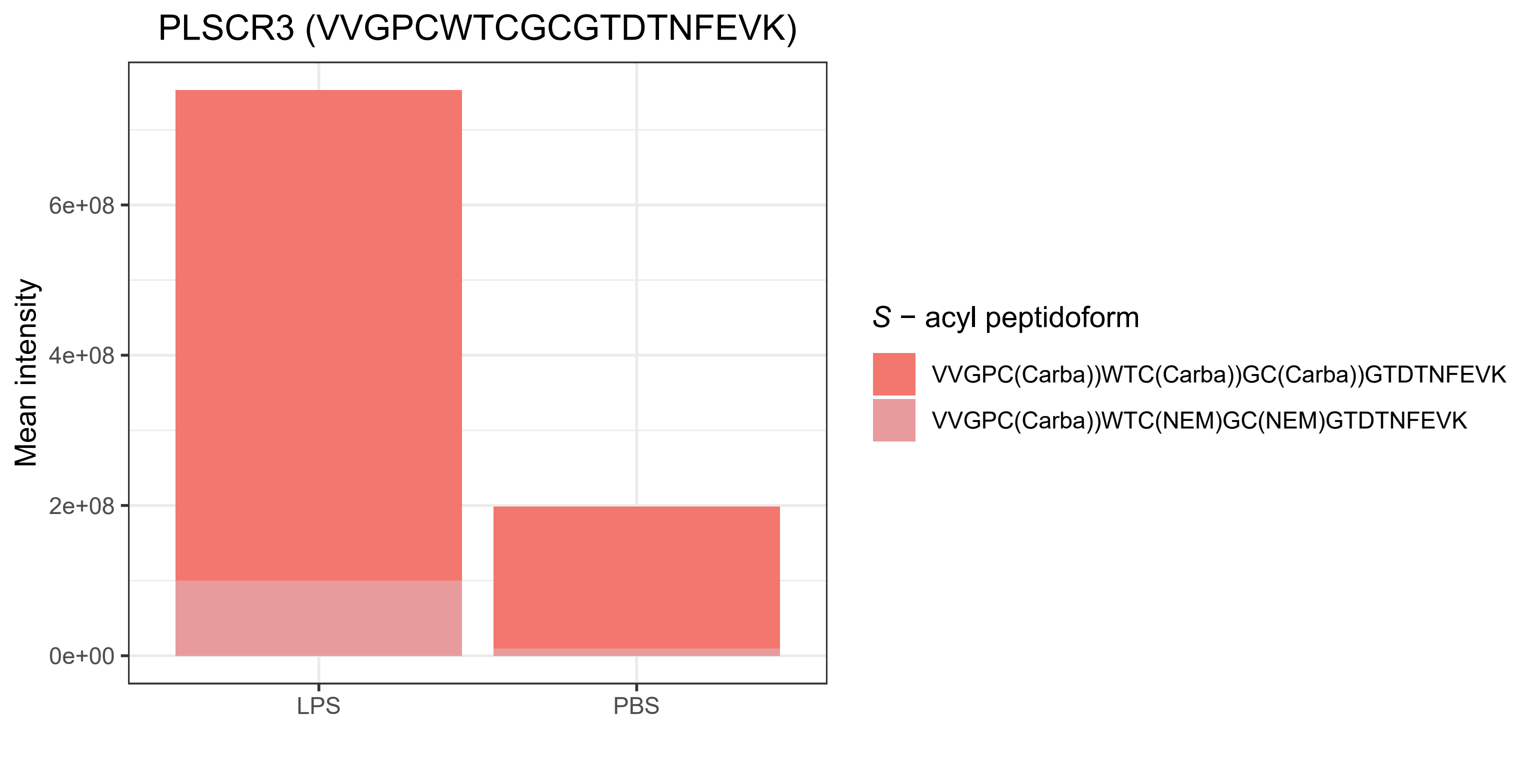

PLSCR3 (VVGPCWTCGCGTDTNFEVK)
Mean intensity
6e+08
4e+08
2e+08
0e+00
LPS
PBS
S − acyl peptidoform
VVGPC(Carba))WTC(Carba))GC(Carba))GTDTNFEVK
VVGPC(Carba))WTC(NEM)GC(NEM)GTDTNFEVK

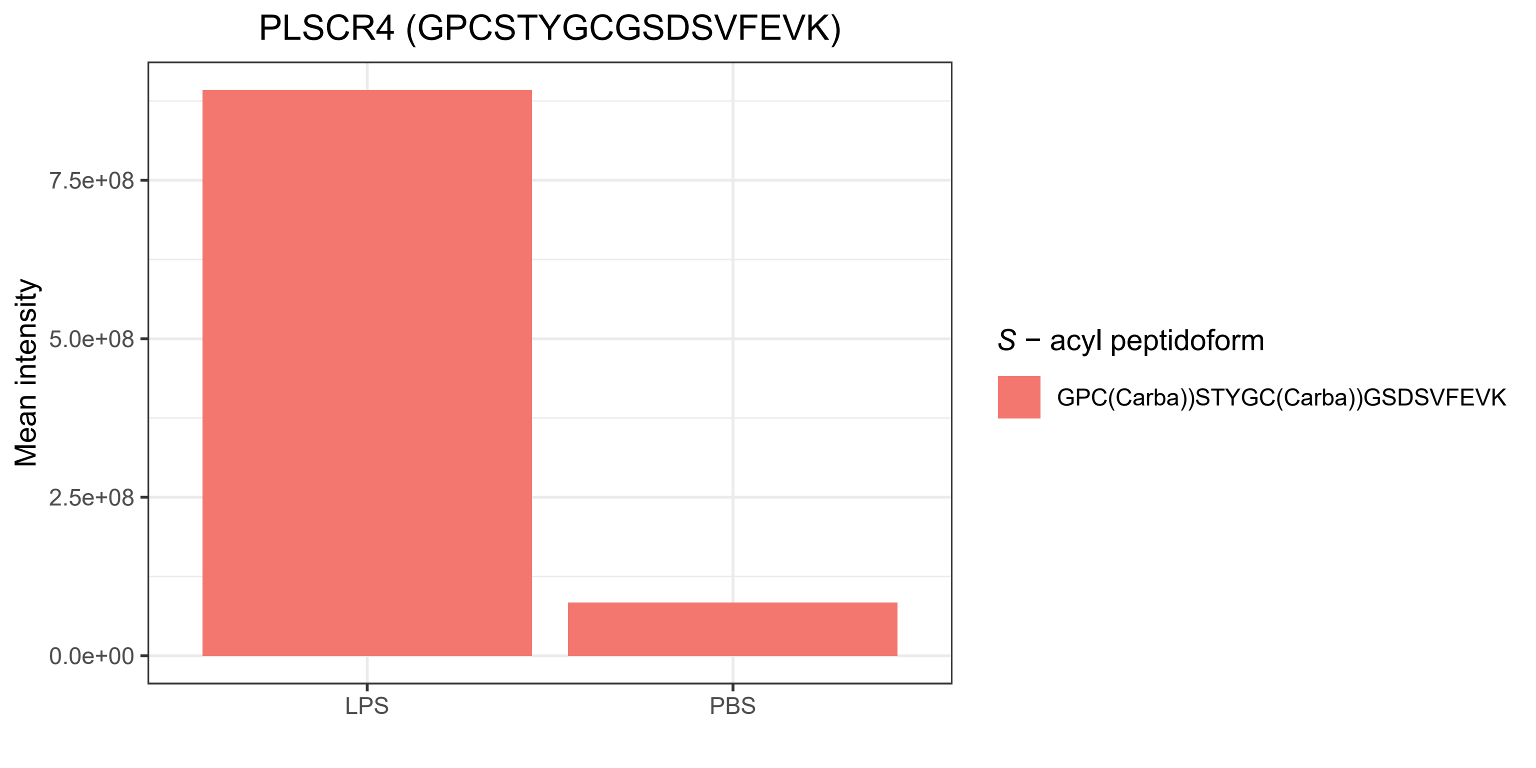
PLSCR4 (GPCSTYGCGSDSVFEVK)
Mean intensity
7.5e+08
5.0e+08
2.5e+08
0.0e+00
LPS
PBS
S − acyl peptidoform
GPC(Carba))STYGC(Carba))GSDSVFEVK

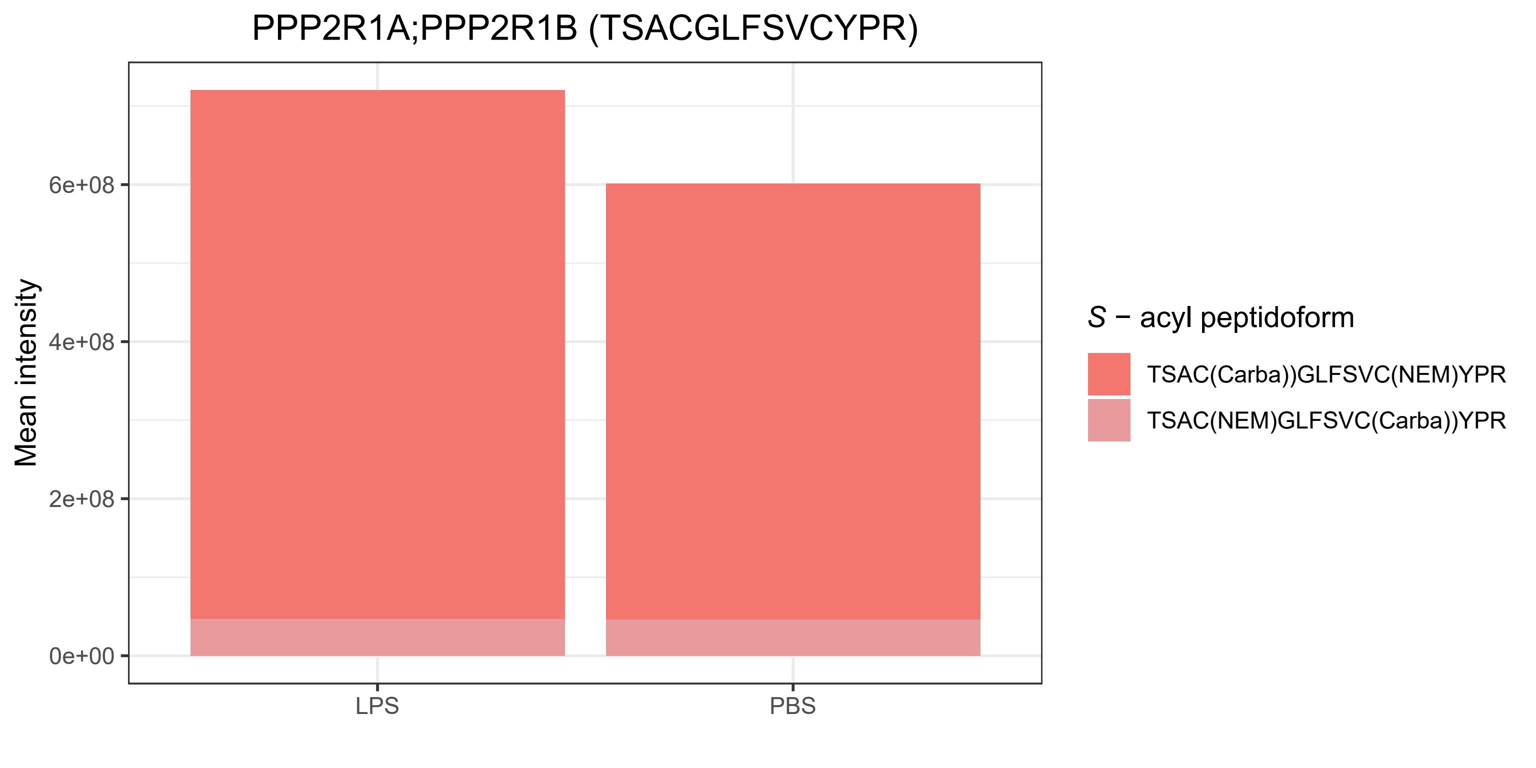

PPP2R1A;PPP2R1B (TSACGLFSVCYPR)
Mean intensity
6e+08
4e+08
2e+08
0e+00
LPS
PBS
S − acyl peptidoform
TSAC(Carba))GLFSVC(NEM)YPR
TSAC(NEM)GLFSVC(Carba))YPR

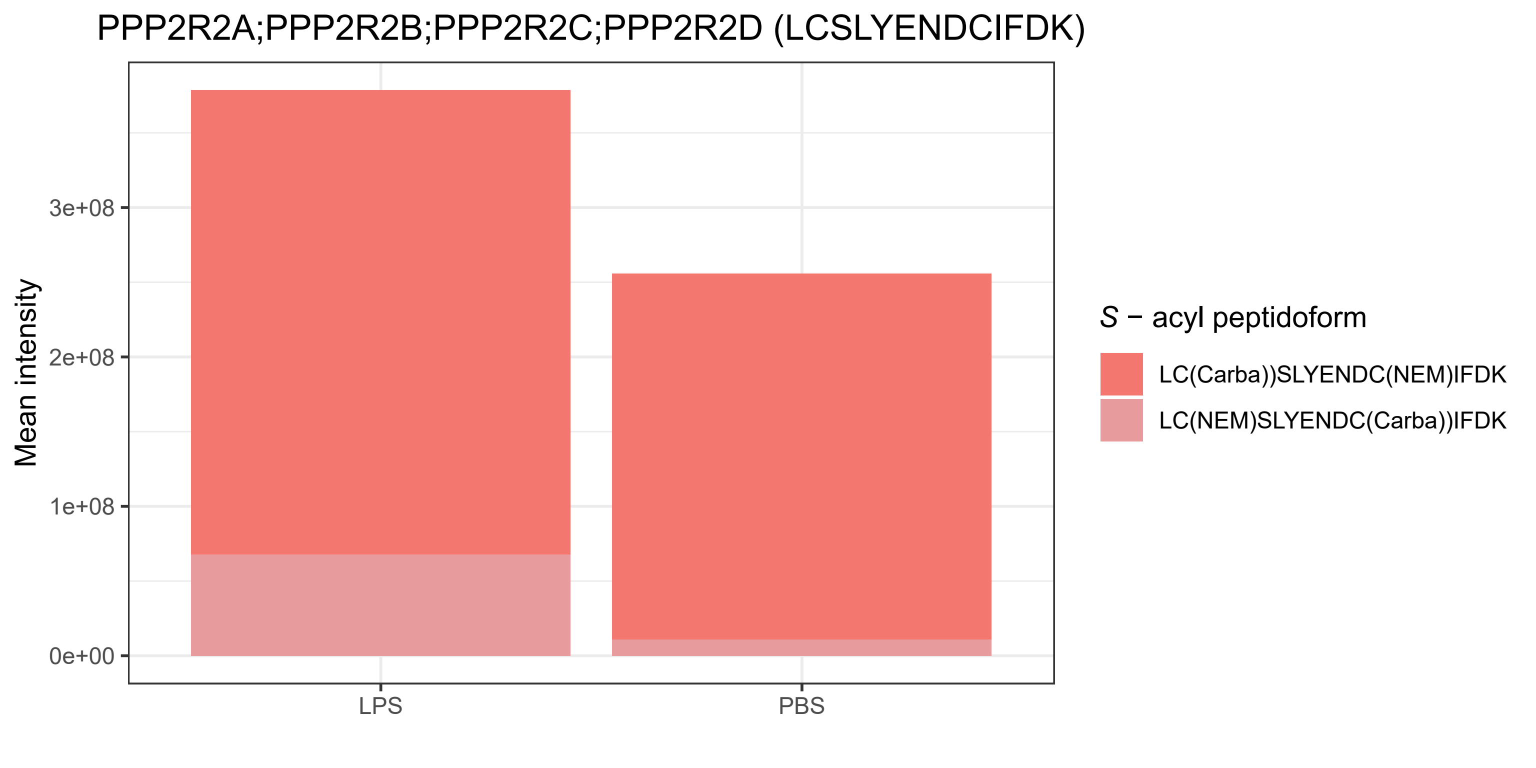

PPP2R2A;PPP2R2B;PPP2R2C;PPP2R2D (LCSLYENDCIFDK)
Mean intensity
3e+08
2e+08
1e+08
0e+00
LPS
PBS
S − acyl peptidoform
LC(Carba))SLYENDC(NEM)IFDK
LC(NEM)SLYENDC(Carba))IFDK

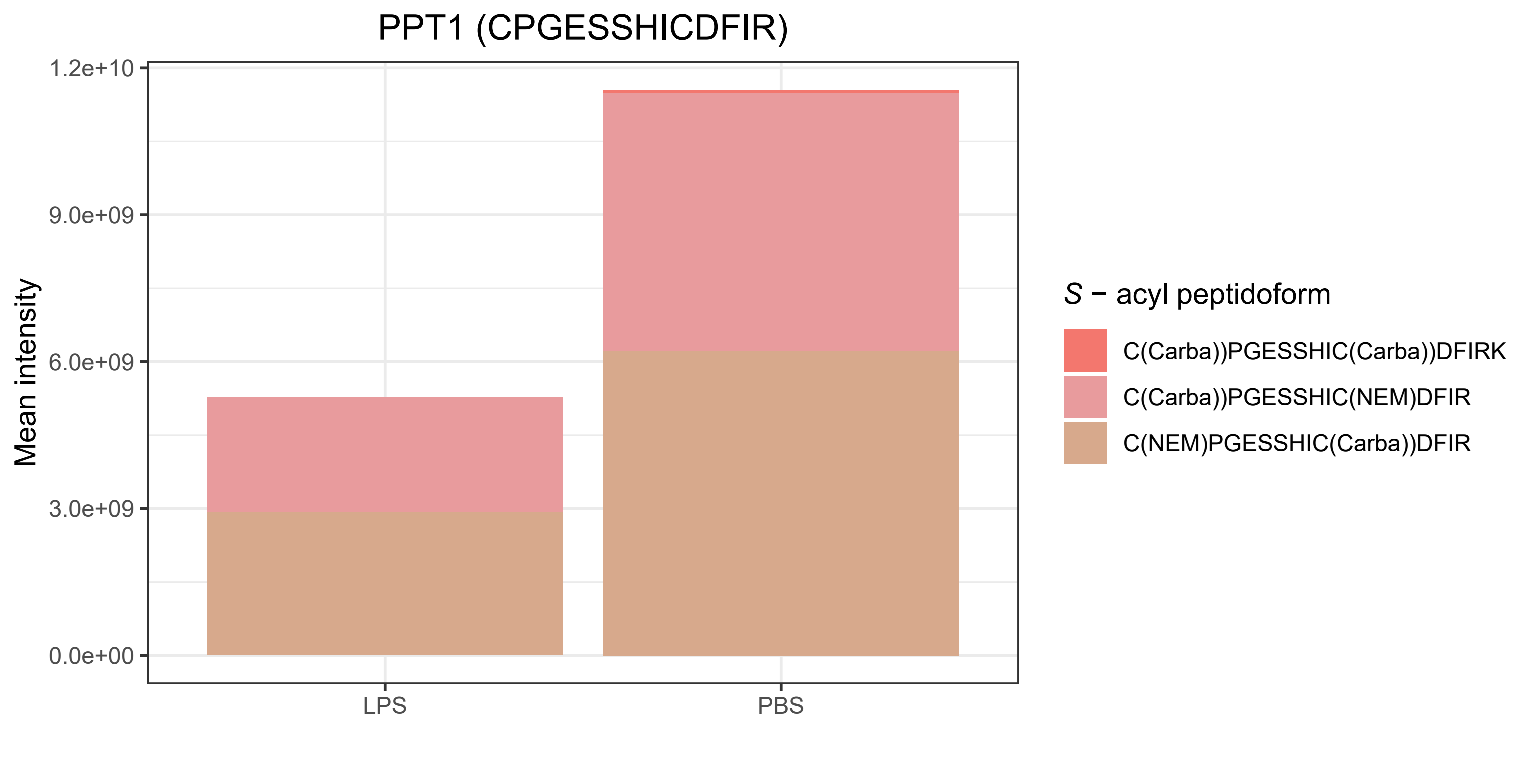

PPT1 (CPGESSHICDFIR)
Mean intensity
1.2e+10
9.0e+09
6.0e+09
3.0e+09
0.0e+00
LPS
PBS
S − acyl peptidoform
C(Carba))PGESSHIC(Carba))DFIRK
C(Carba))PGESSHIC(NEM)DFIR
C(NEM)PGESSHIC(Carba))DFIR

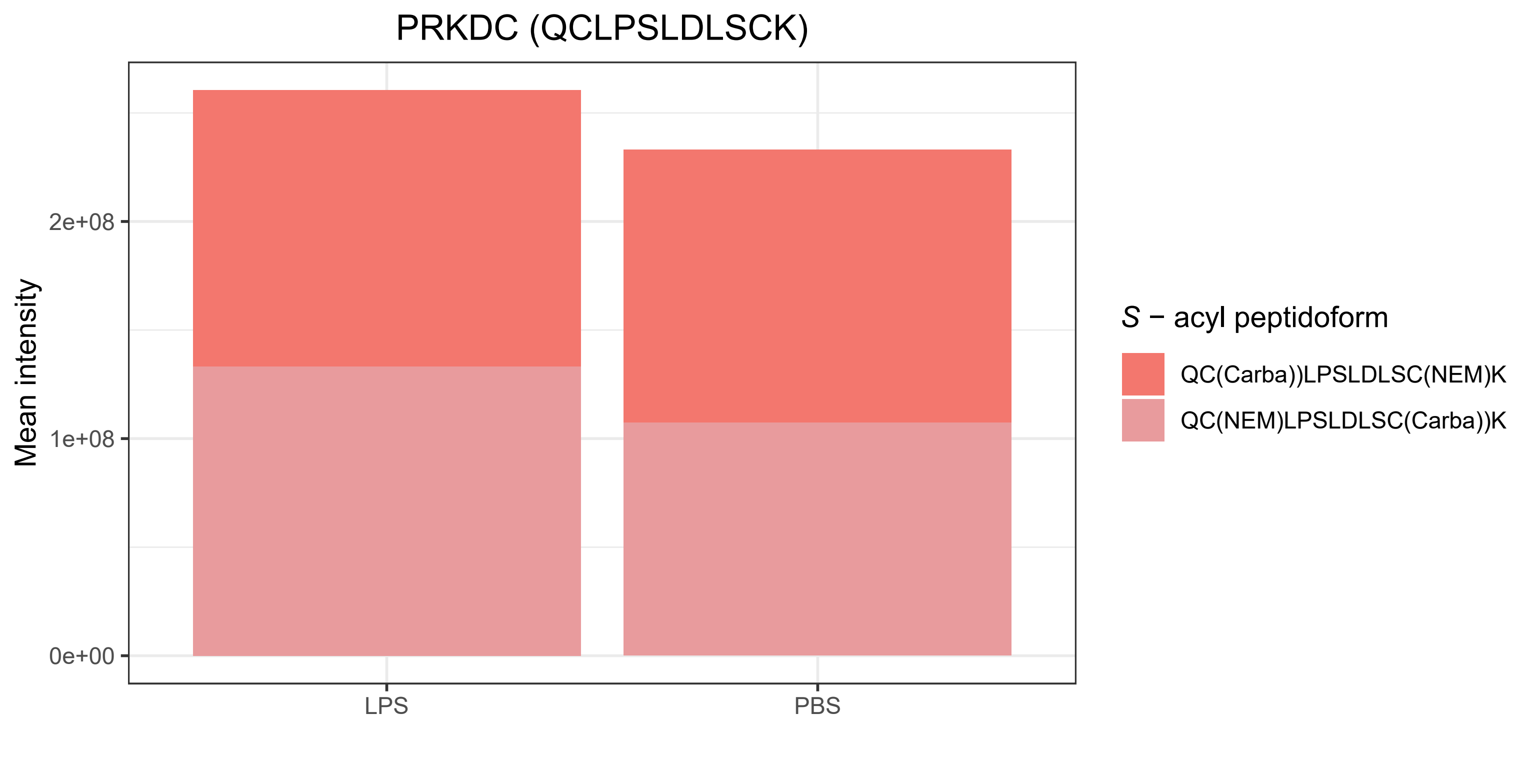

PRKDC (QCLPSLDLSCK)
Mean intensity
2e+08
1e+08
0e+00
LPS
PBS
S − acyl peptidoform
QC(Carba))LPSLDLSC(NEM)K
QC(NEM)LPSLDLSC(Carba))K

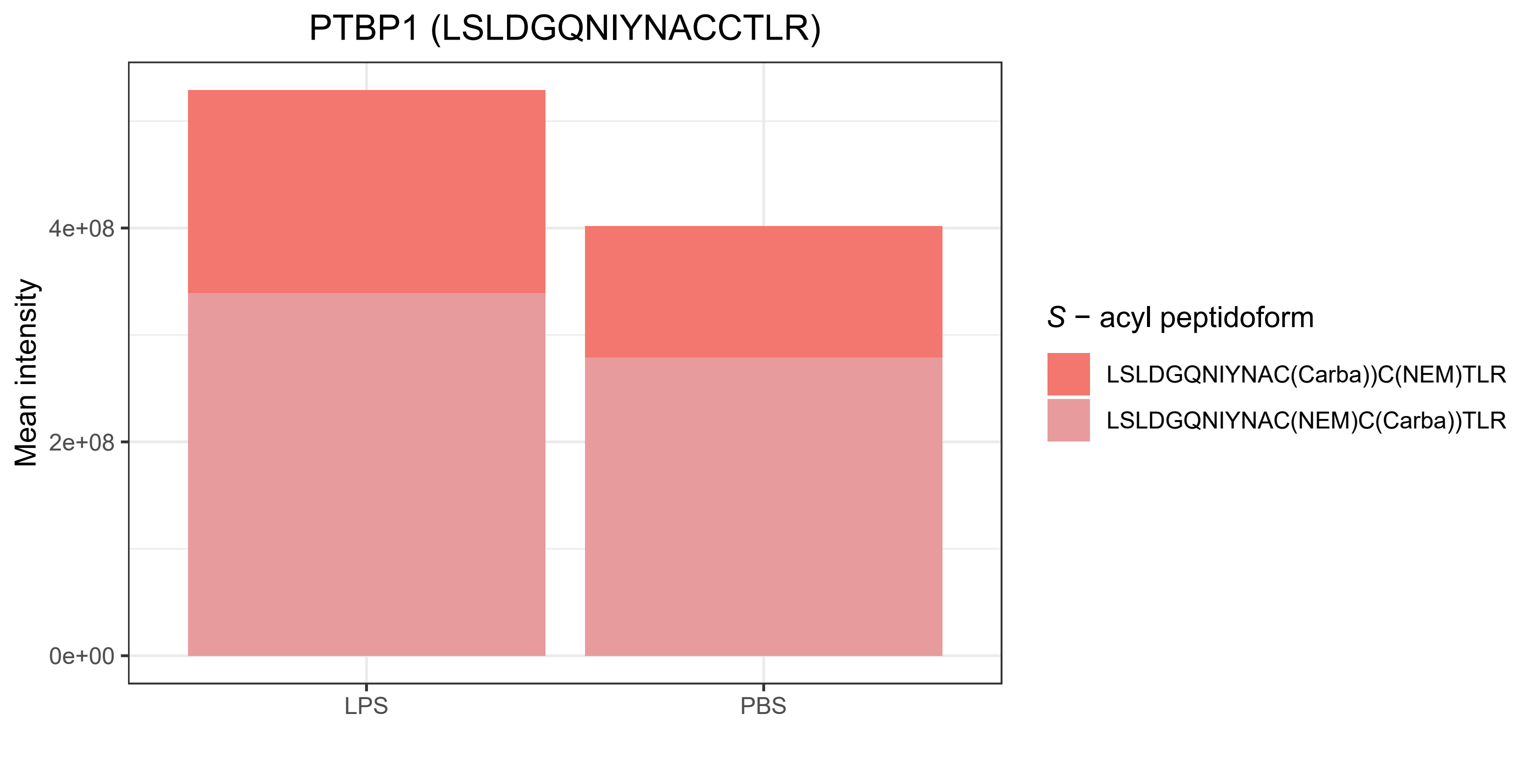

PTBP1 (LSLDGQNIYNACCTLR)
Mean intensity
4e+08
2e+08
0e+00
LPS
PBS
S − acyl peptidoform
LSLDGQNIYNAC(Carba))C(NEM)TLR
LSLDGQNIYNAC(NEM)C(Carba))TLR

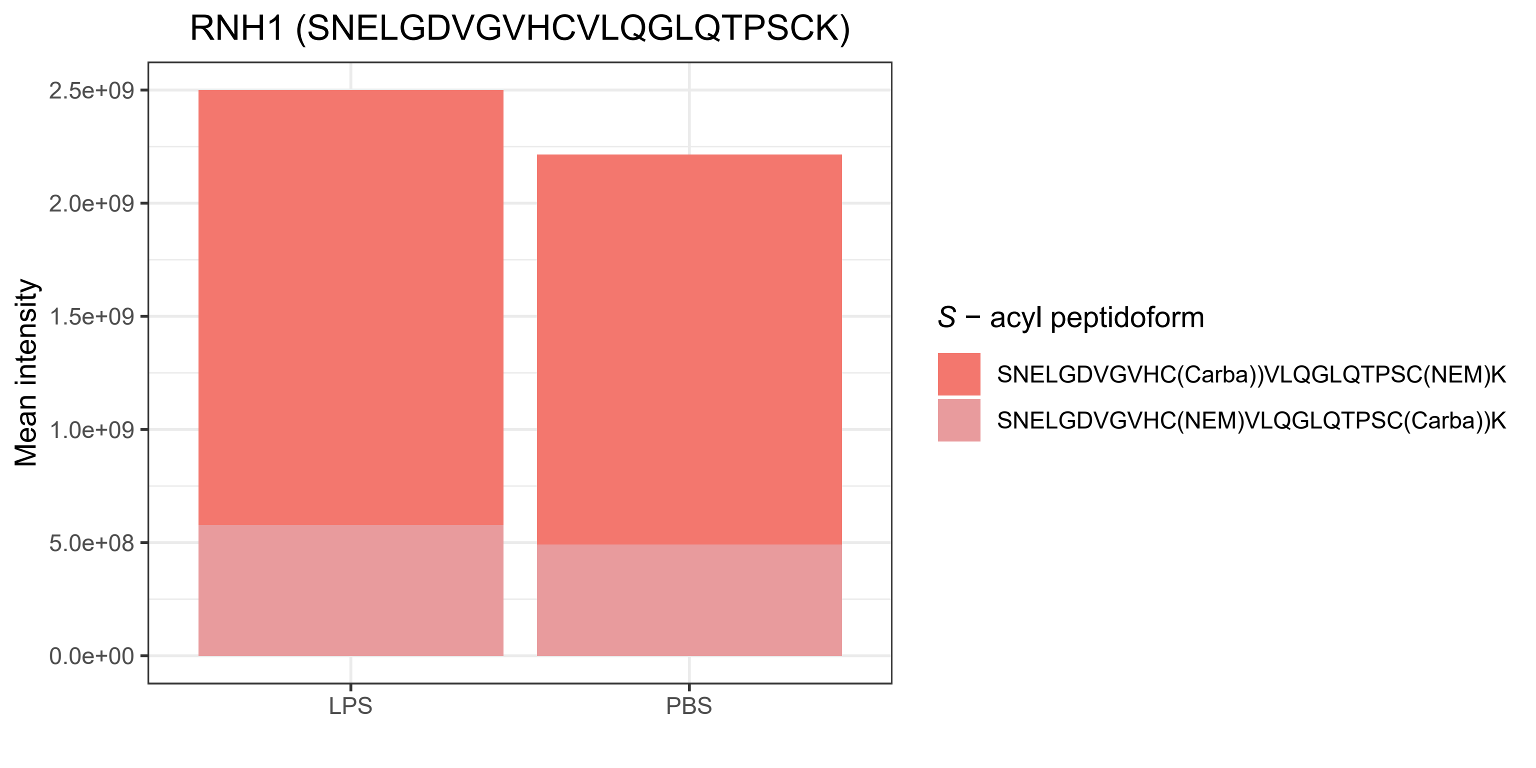
RNH1 (SNELGDVGVHCVLQGLQTPSCK)
Mean intensity
2.5e+09
2.0e+09
1.5e+09
1.0e+09
5.0e+08
0.0e+00
LPS
PBS
S − acyl peptidoform
SNELGDVGVHC(Carba))VLQGLQTPSC(NEM)K
SNELGDVGVHC(NEM)VLQGLQTPSC(Carba))K

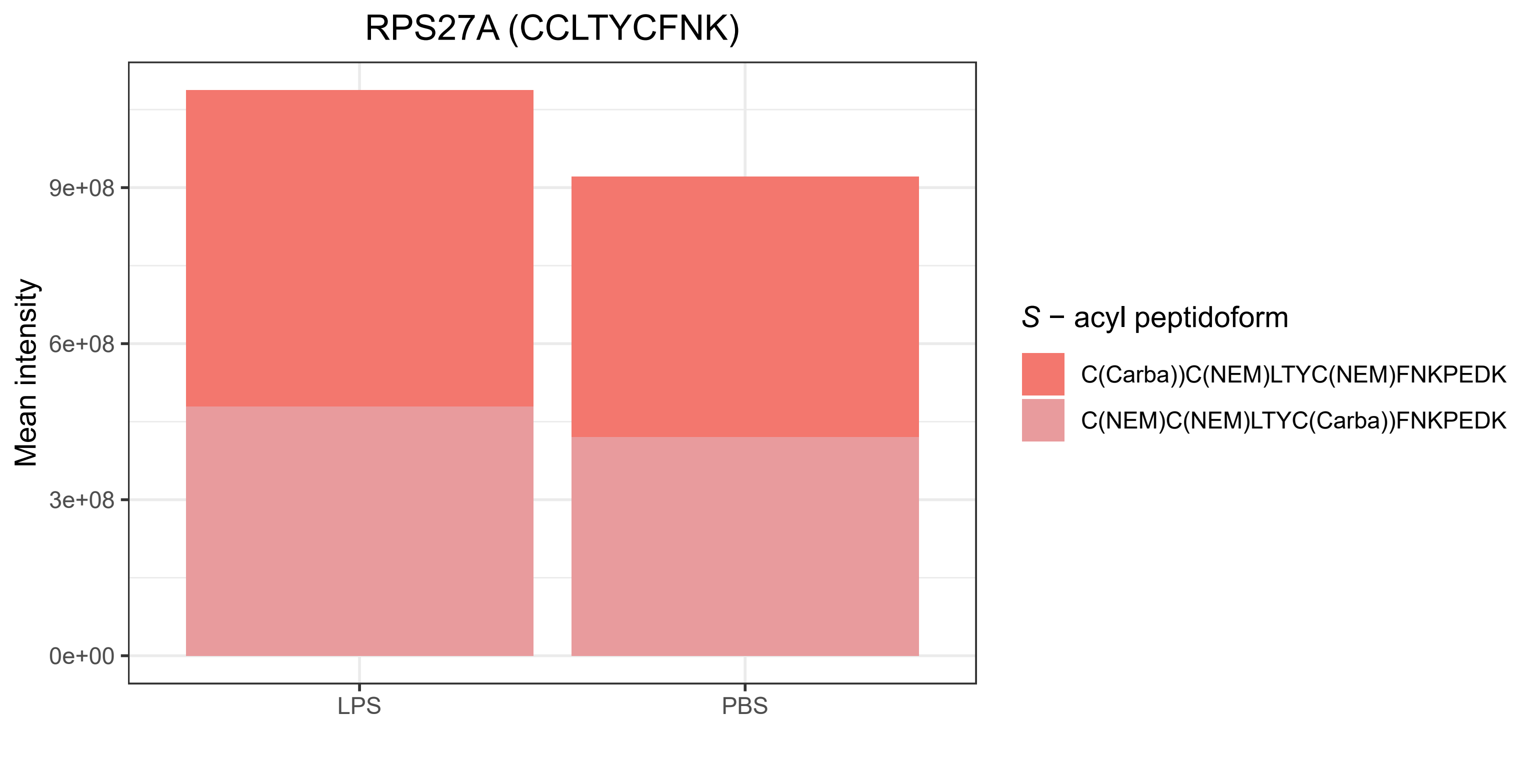
RPS27A (CCLTYCFNK)
Mean intensity
9e+08
6e+08
3e+08
0e+00
LPS
PBS
S − acyl peptidoform
C(Carba))C(NEM)LTYC(NEM)FNKPEDK
C(NEM)C(NEM)LTYC(Carba))FNKPEDK

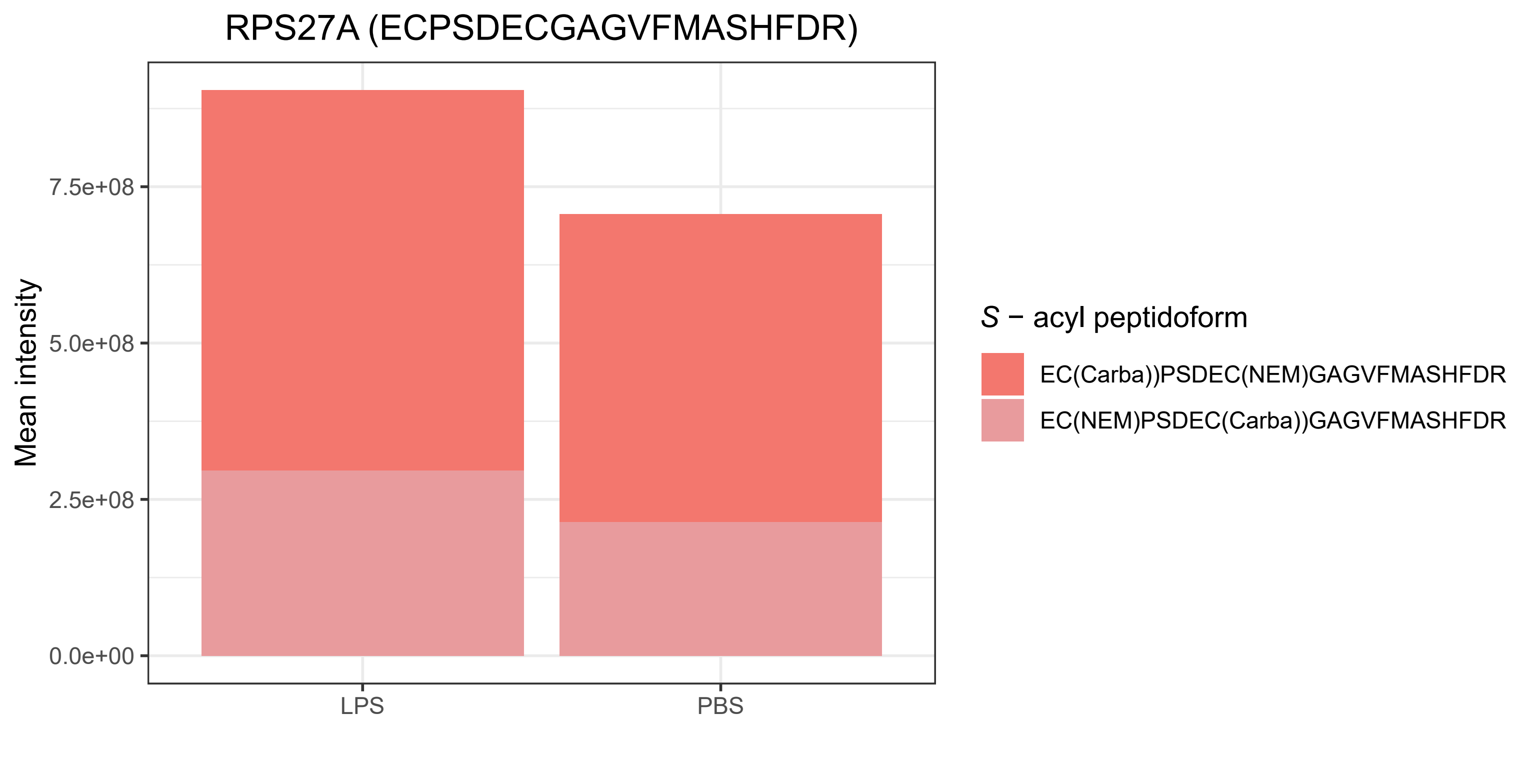

RPS27A (ECPSDECGAGVFMASHFDR)
Mean intensity
7.5e+08
5.0e+08
2.5e+08
0.0e+00
LPS
PBS
S − acyl peptidoform
EC(Carba))PSDEC(NEM)GAGVFMASHFDR
EC(NEM)PSDEC(Carba))GAGVFMASHFDR

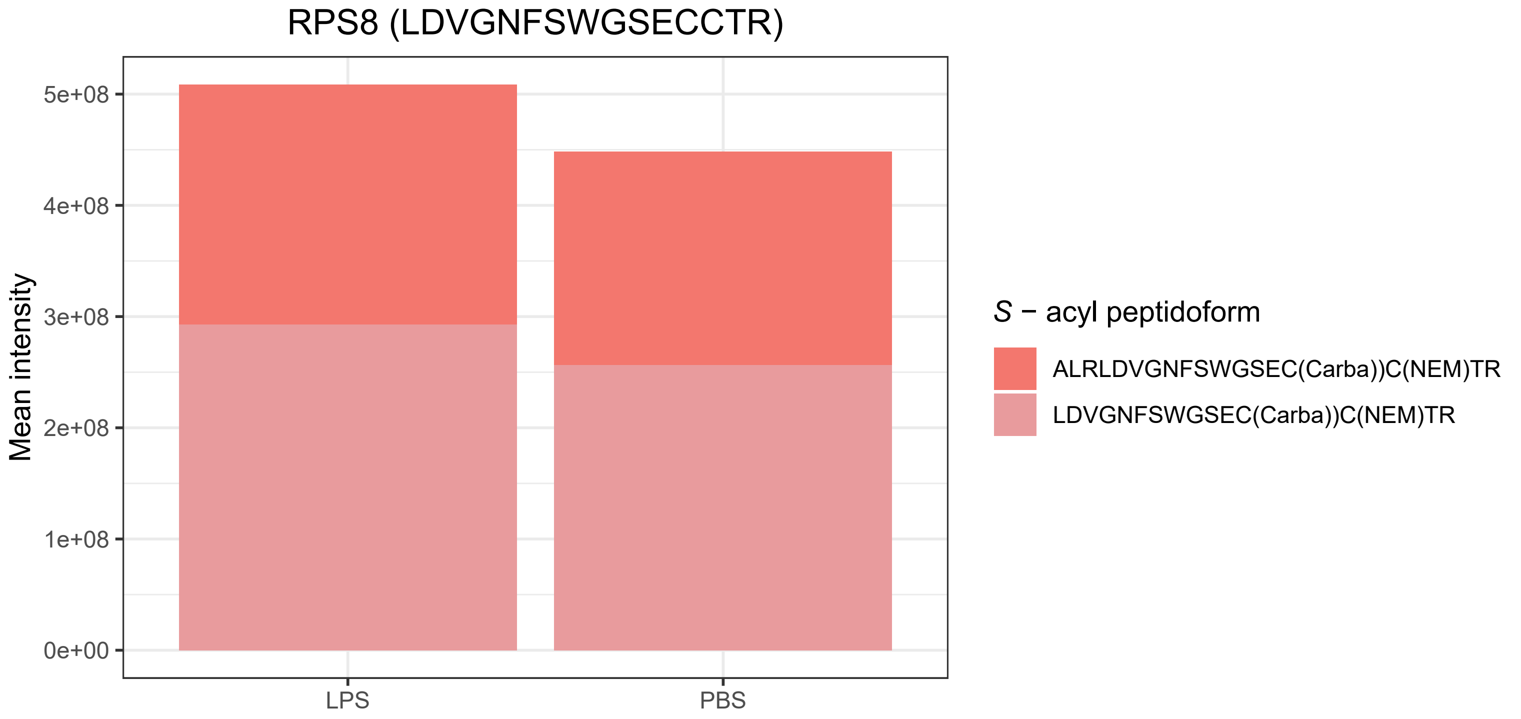
RPS8 (LDVGNFSWGSECCTR)
Mean intensity
5e+08
4e+08
3e+08
2e+08
1e+08
0e+00
LPS
PBS
S − acyl peptidoform
ALRLDVGNFSWGSEC(Carba))C(NEM)TR
LDVGNFSWGSEC(Carba))C(NEM)TR

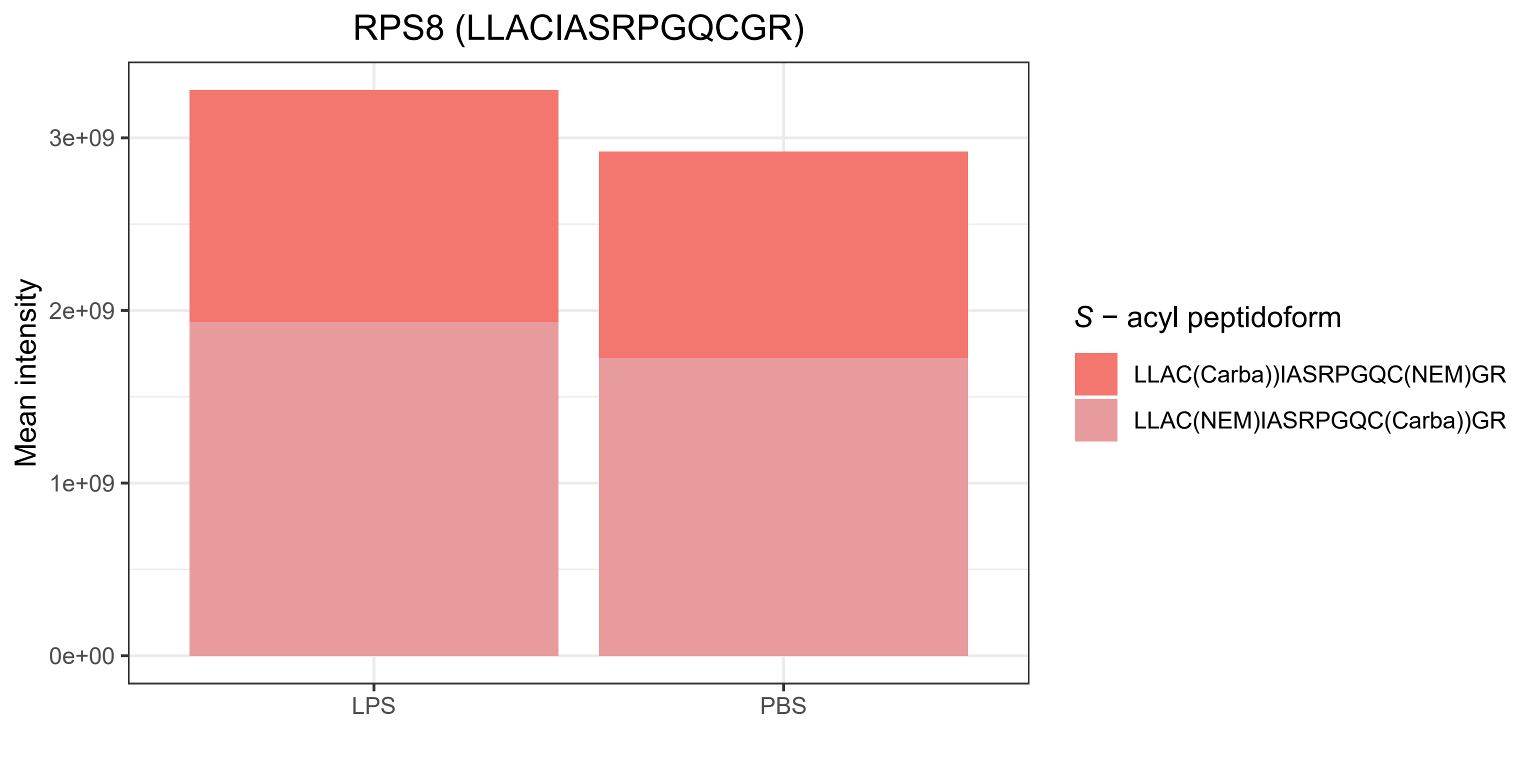

RPS8 (LLACIASRPGQCGR)
Mean intensity
3e+09
2e+09
1e+09
0e+00
LPS
PBS
S − acyl peptidoform
LLAC(Carba))IASRPGQC(NEM)GR
LLAC(NEM)IASRPGQC(Carba))GR

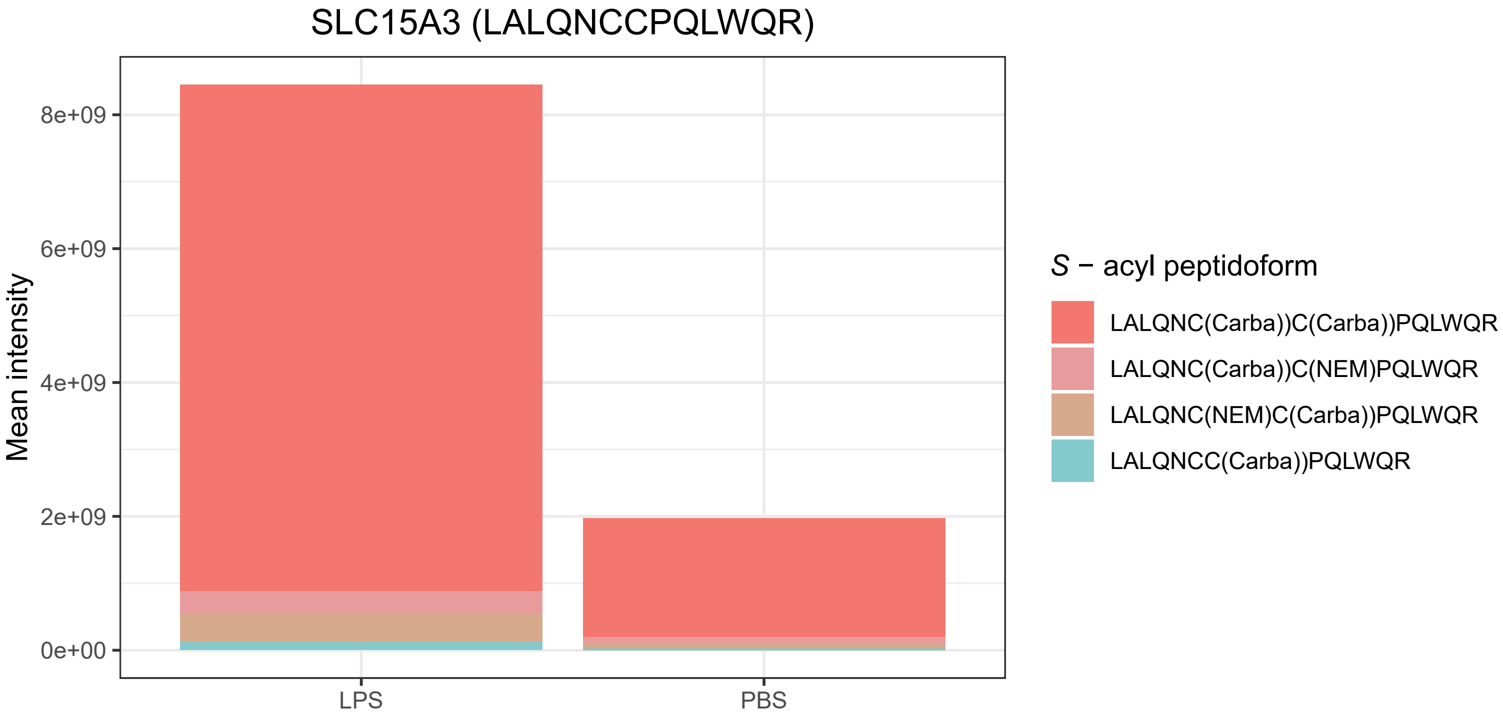
SLC15A3 (LALQNCCPQLWQR)
Mean intensity
8e+09
6e+09
4e+09
2e+09
0e+00
LPS
PBS
S − acyl peptidoform
LALQNC(Carba))C(Carba))PQLWQR
LALQNC(Carba))C(NEM)PQLWQR
LALQNC(NEM)C(Carba))PQLWQR
LALQNCC(Carba))PQLWQR

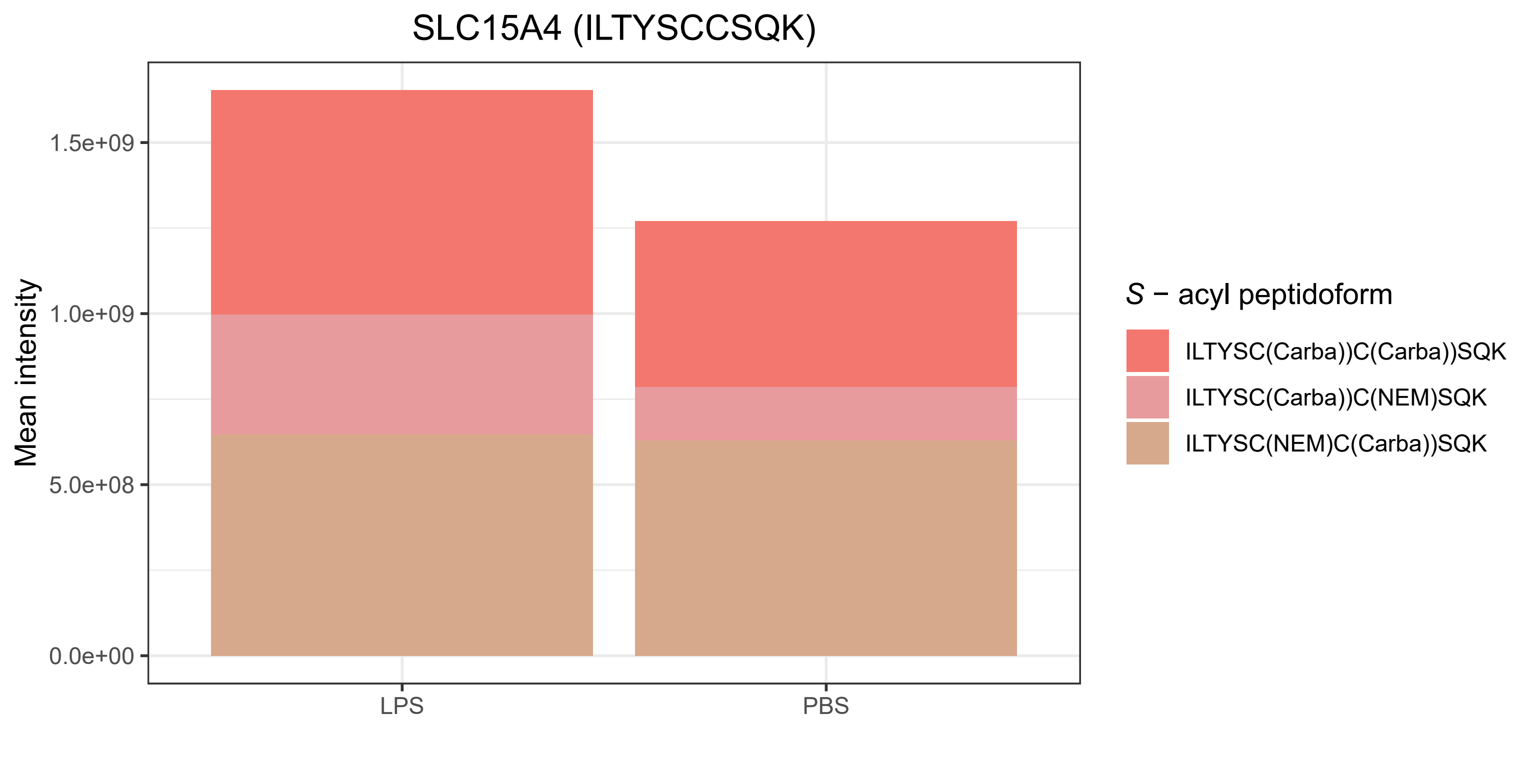
SLC15A4 (ILTYSCCSQK)
Mean intensity
1.5e+09
1.0e+09
5.0e+08
0.0e+00
LPS
PBS
S − acyl peptidoform
ILTYSC(Carba))C(Carba))SQK
ILTYSC(Carba))C(NEM)SQK
ILTYSC(NEM)C(Carba))SQK

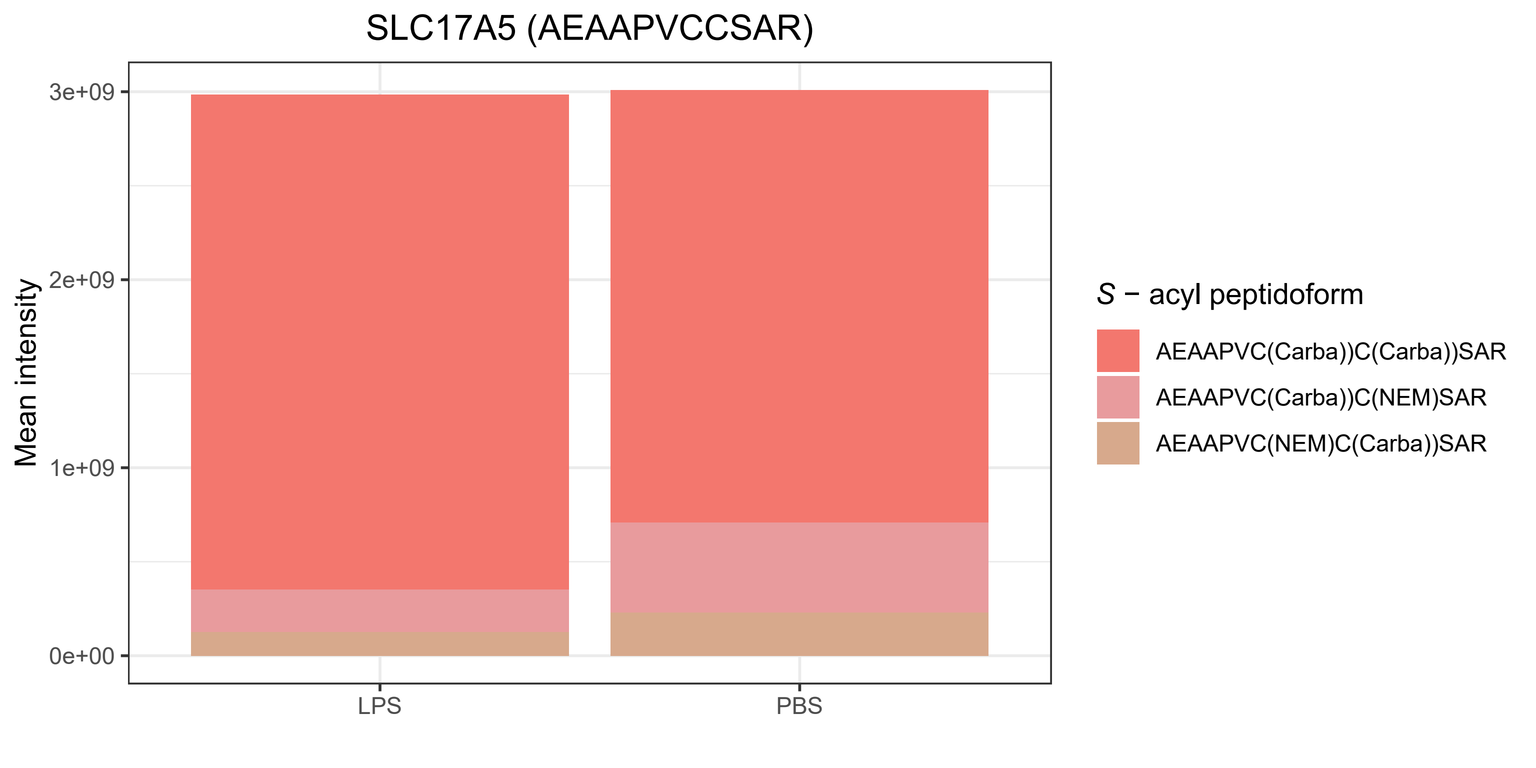
SLC17A5 (AEAAPVCCSAR)
3e+09
2e+09
1e+09
0e+00
Mean intensity
LPS
PBS
S − acyl peptidoform
AEAAPVC(Carba))C(Carba))SAR
AEAAPVC(Carba))C(NEM)SAR
AEAAPVC(NEM)C(Carba))SAR

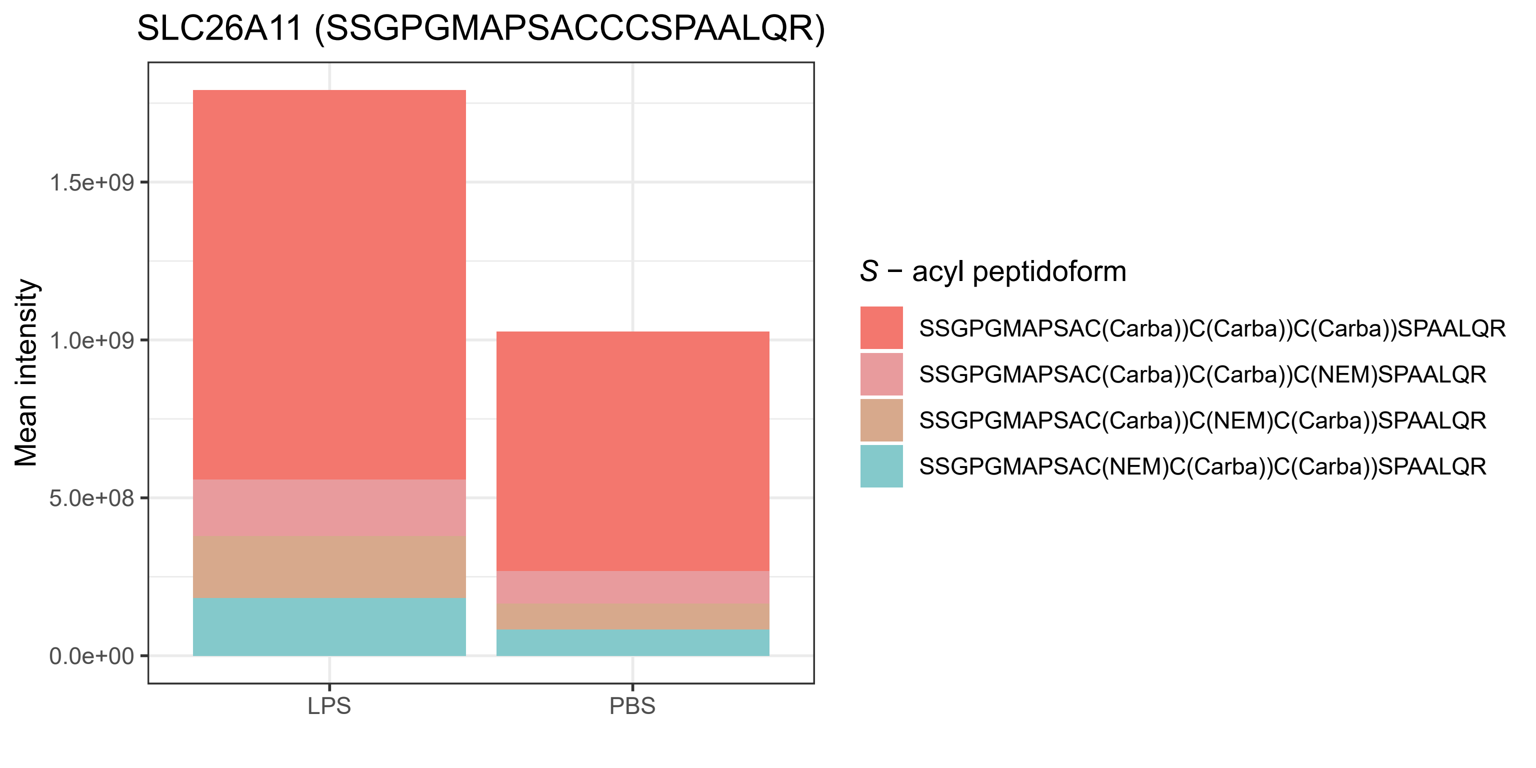
SLC26A11 (SSGPGMAPSACCCSPAALQR)
Mean intensity
1.5e+09
1.0e+09
5.0e+08
0.0e+00
LPS
PBS
S − acyl peptidoform
SSGPGMAPSAC(Carba))C(Carba))C(Carba))SPAALQR
SSGPGMAPSAC(Carba))C(Carba))C(NEM)SPAALQR
SSGPGMAPSAC(Carba))C(NEM)C(Carba))SPAALQR
SSGPGMAPSAC(NEM)C(Carba))C(Carba))SPAALQR

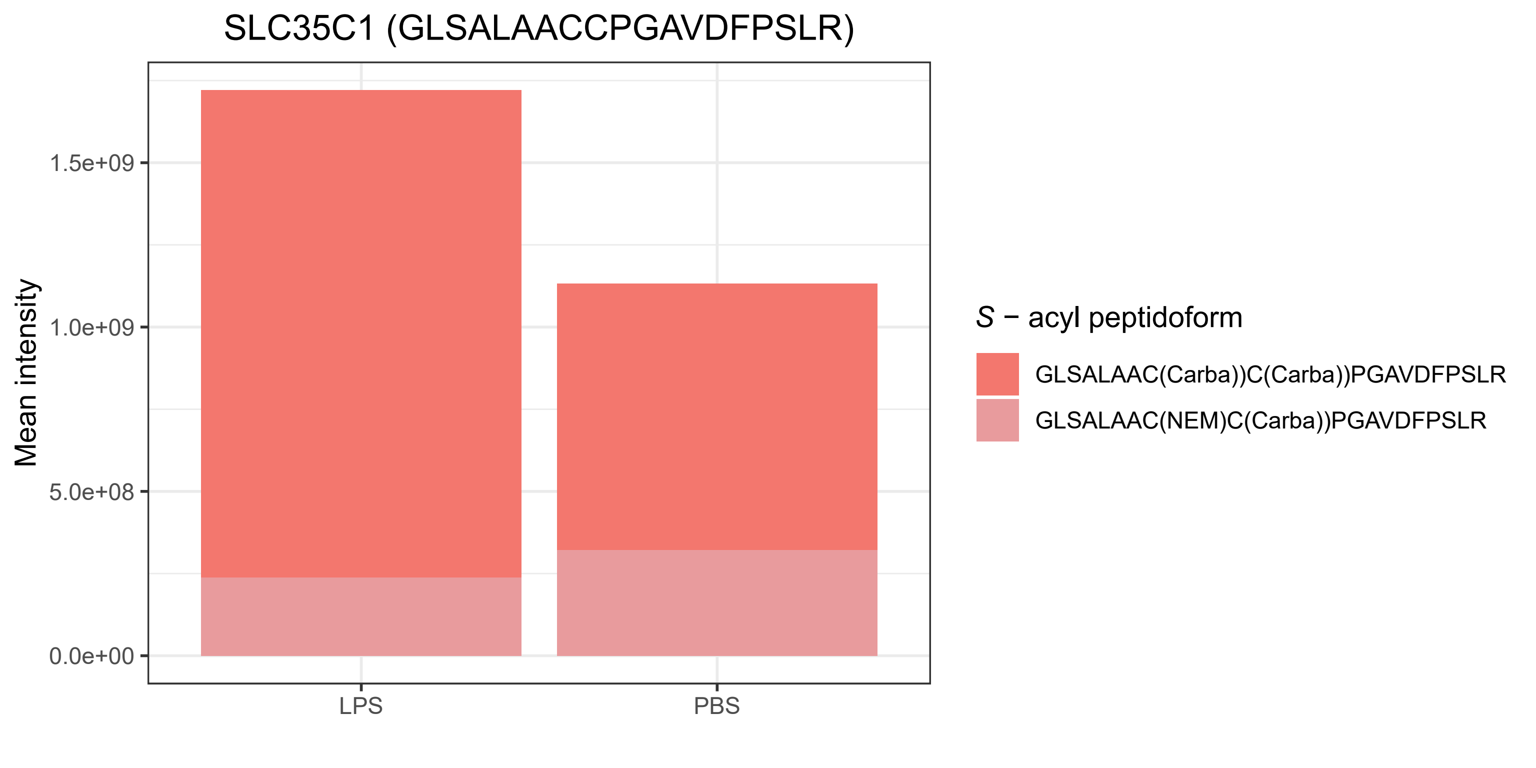
SLC35C1 (GLSALAACCPGAVDFPSLR)
Mean intensity
1.5e+09
1.0e+09
5.0e+08
0.0e+00
LPS
PBS
S − acyl peptidoform
GLSALAAC(Carba))C(Carba))PGAVDFPSLR
GLSALAAC(NEM)C(Carba))PGAVDFPSLR

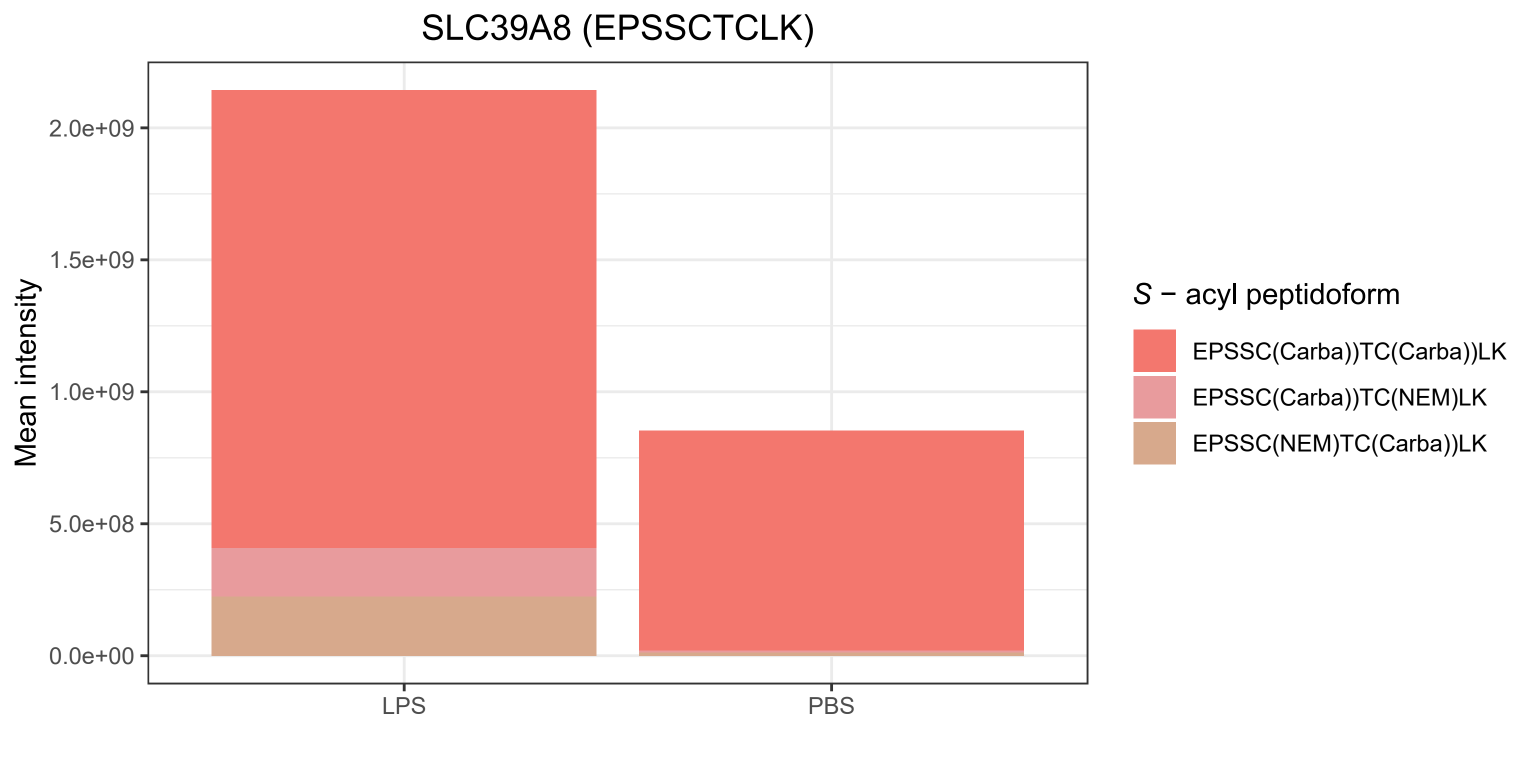

SLC39A8 (EPSSCTCLK)
Mean intensity
2.0e+09
1.5e+09
1.0e+09
5.0e+08
0.0e+00
LPS
PBS
S − acyl peptidoform
EPSSC(Carba))TC(Carba))LK
EPSSC(Carba))TC(NEM)LK
EPSSC(NEM)TC(Carba))LK

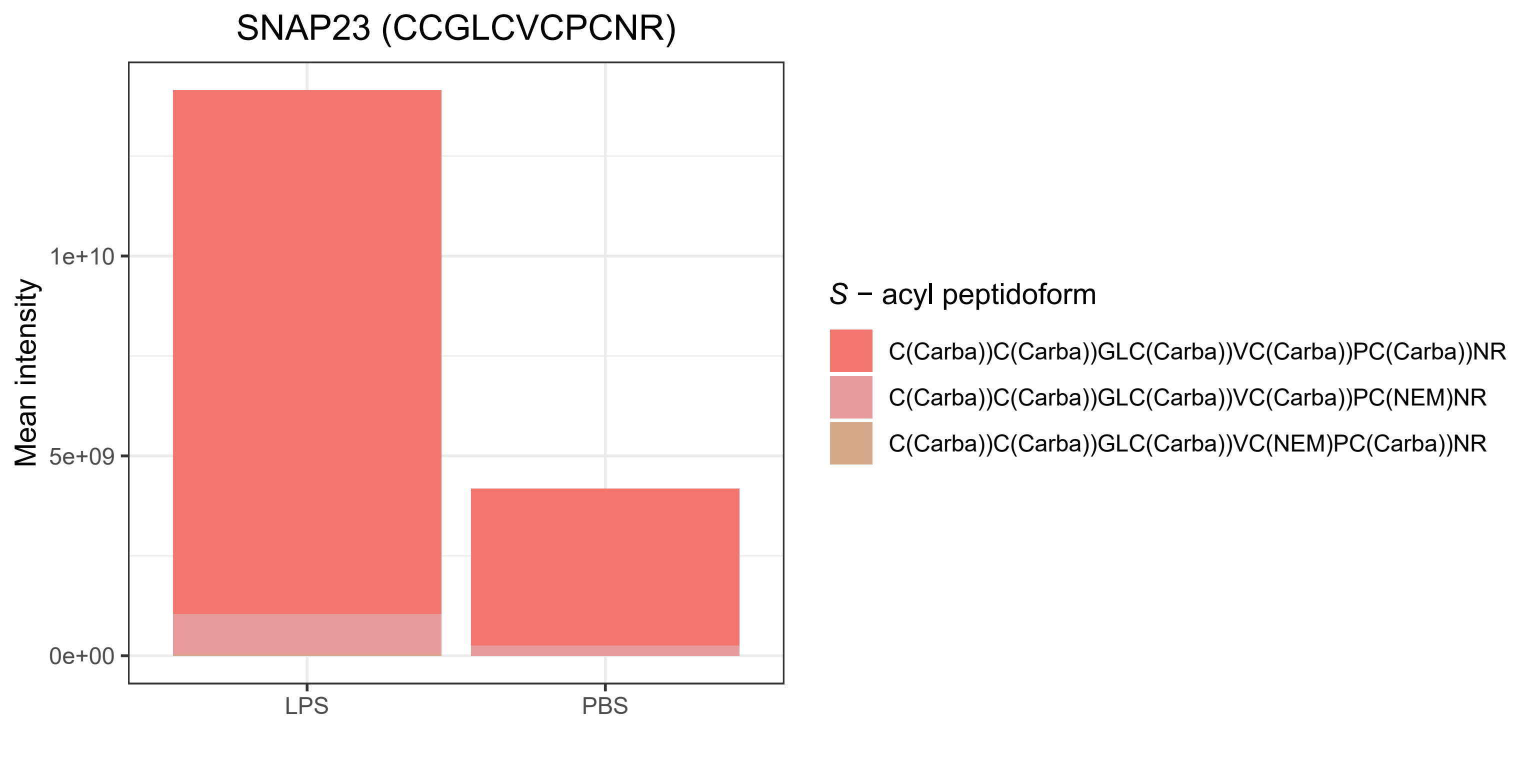
SNAP23 (CCGLCVCPCNR)
Mean intensity
1e+10
5e+09
0e+00
LPS
PBS
S − acyl peptidoform
C(Carba))C(Carba))GLC(Carba))VC(Carba))PC(Carba))NR
C(Carba))C(Carba))GLC(Carba))VC(Carba))PC(NEM)NR
C(Carba))C(Carba))GLC(Carba))VC(NEM)PC(Carba))NR

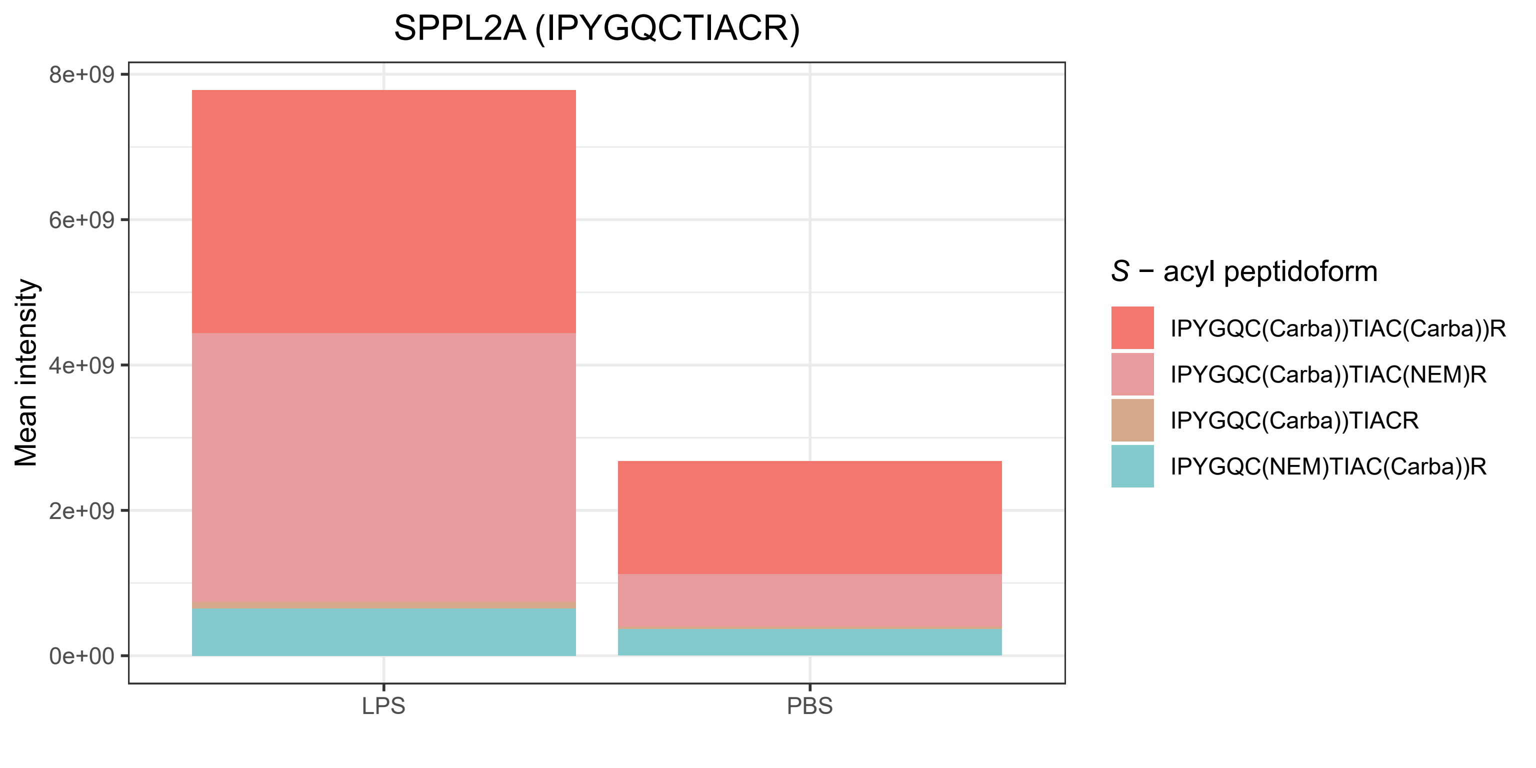
SPPL2A (IPYGQCTIACR)
Mean intensity
8e+09
6e+09
4e+09
2e+09
0e+00
LPS
PBS
S − acyl peptidoform
IPYGQC(Carba))TIAC(Carba))R
IPYGQC(Carba))TIAC(NEM)R
IPYGQC(Carba))TIACR
IPYGQC(NEM)TIAC(Carba))R

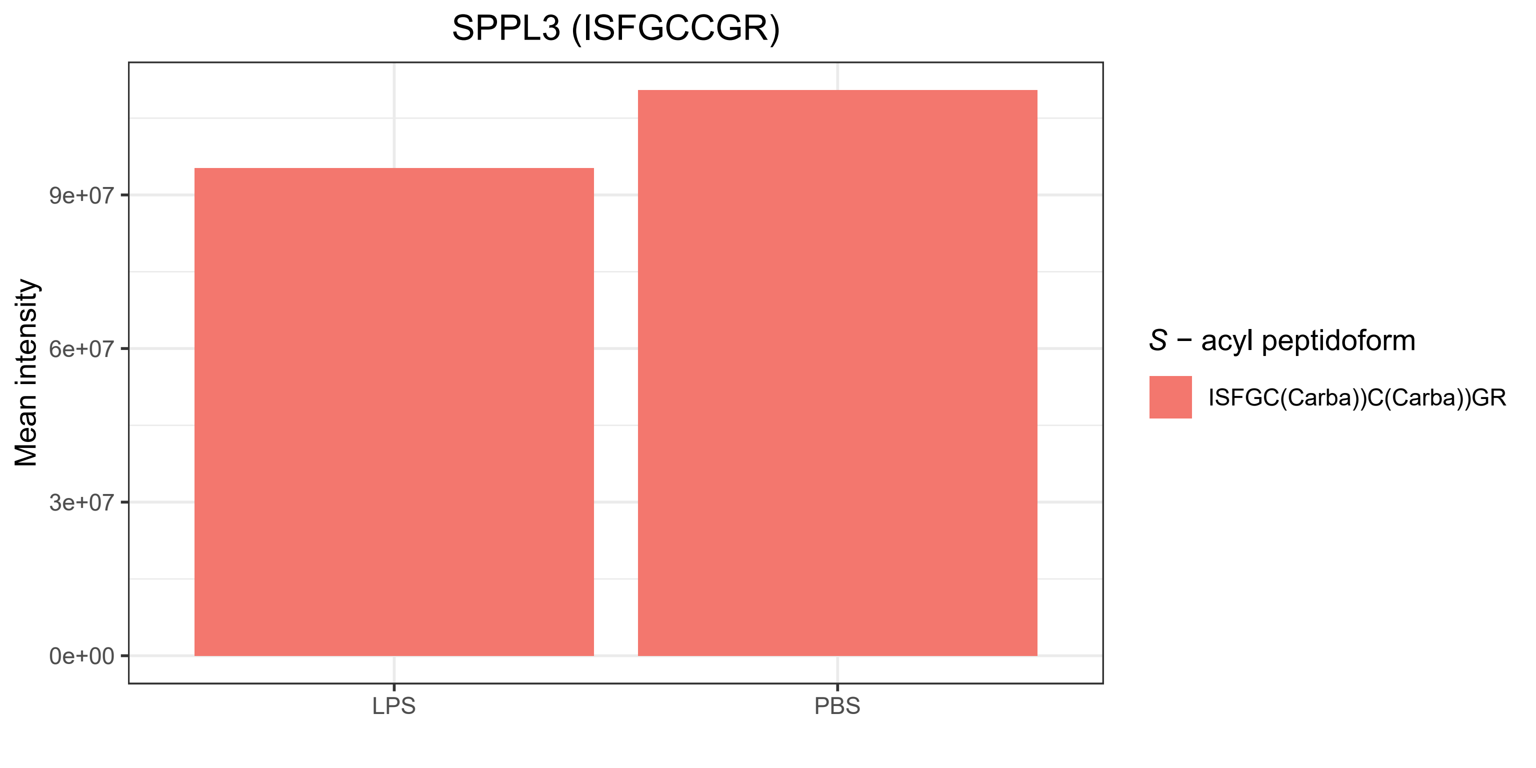

SPPL3 (ISFGCCGR)
Mean intensity
9e+07
6e+07
3e+07
0e+00
LPS
PBS
S − acyl peptidoform
ISFGC(Carba))C(Carba))GR

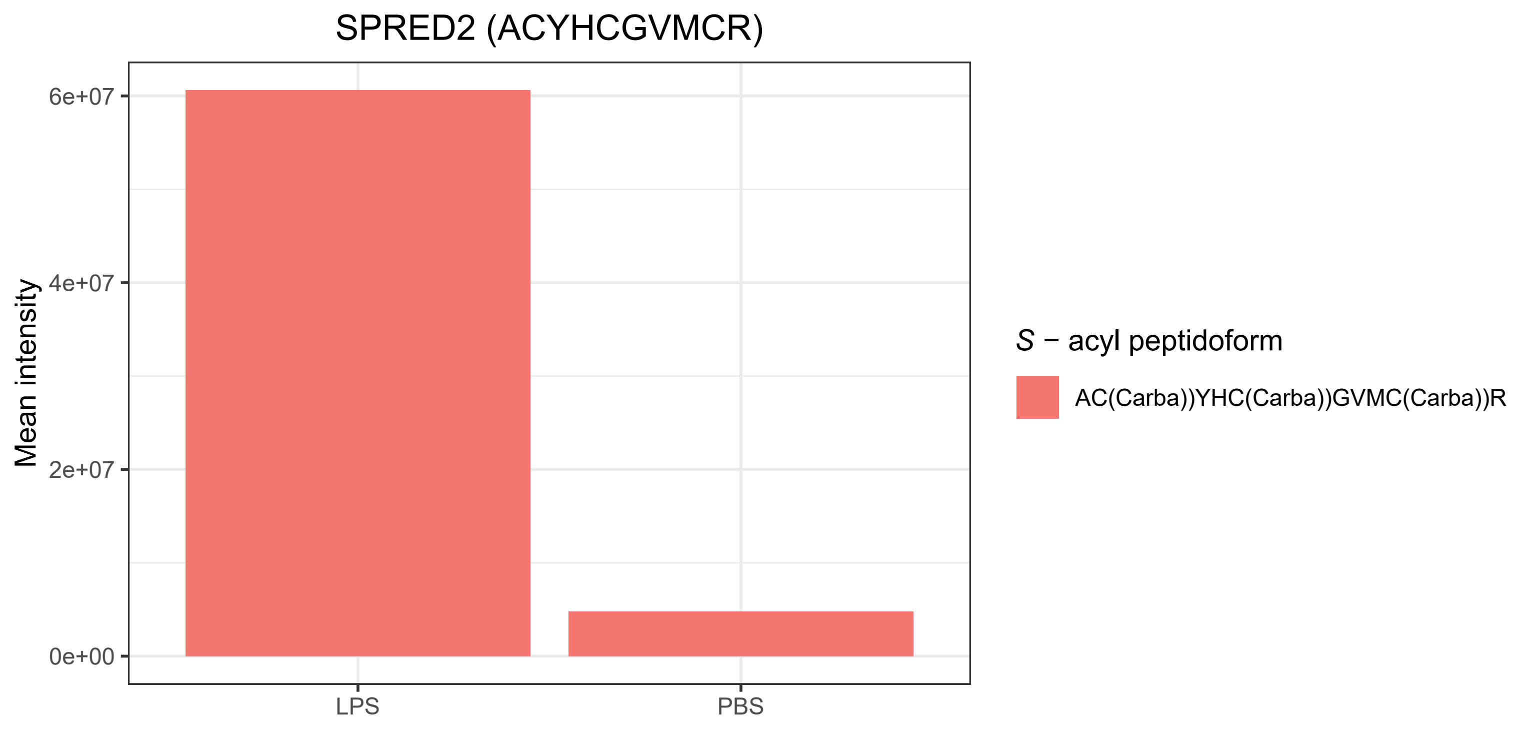

SPRED2 (ACYHCGVMCR)
Mean intensity
6e+07
4e+07
2e+07
0e+00
LPS
PBS
S − acyl peptidoform
AC(Carba))YHC(Carba))GVMC(Carba))R

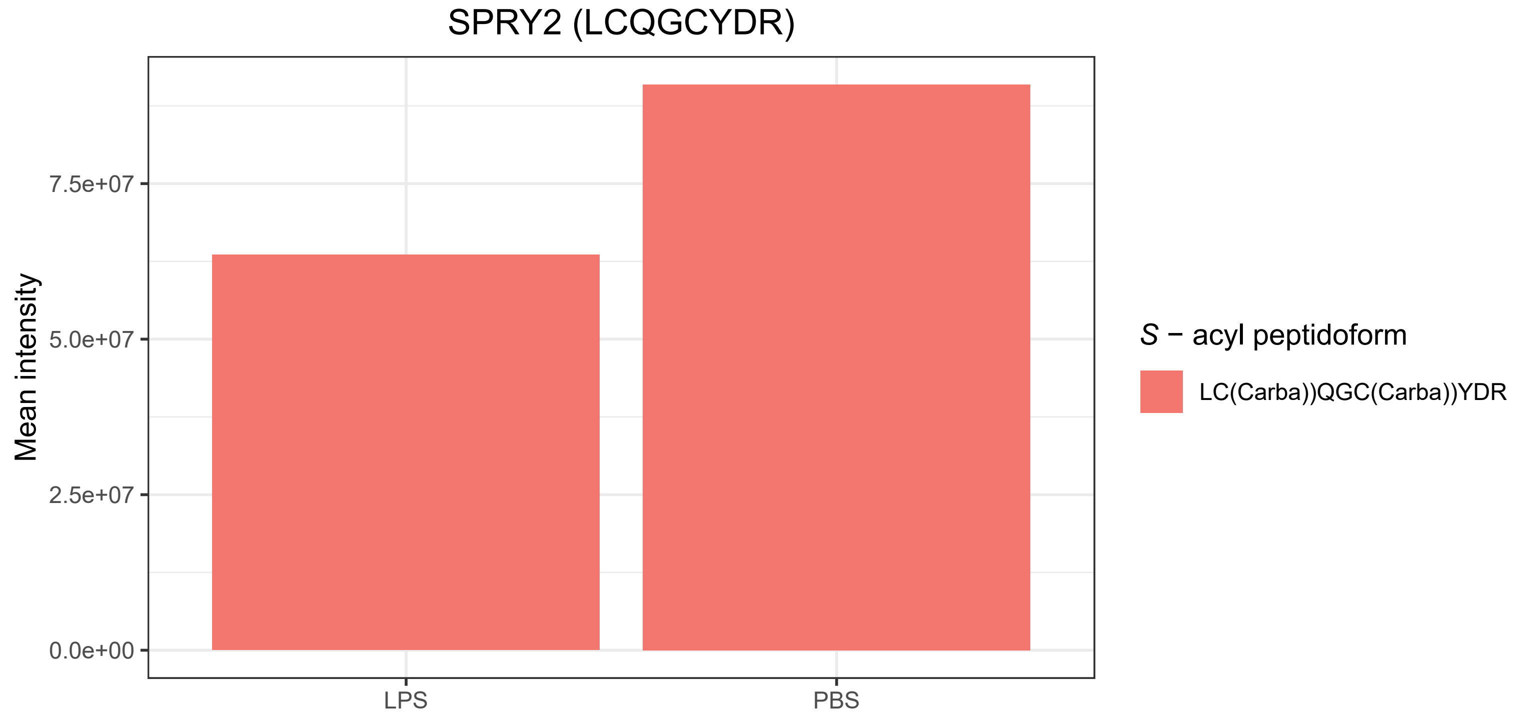
SPRY2 (LCQGCYDR)
Mean intensity
7.5e+07
5.0e+07
2.5e+07
0.0e+00
LPS
PBS
S − acyl peptidoform
LC(Carba))QGC(Carba))YDR

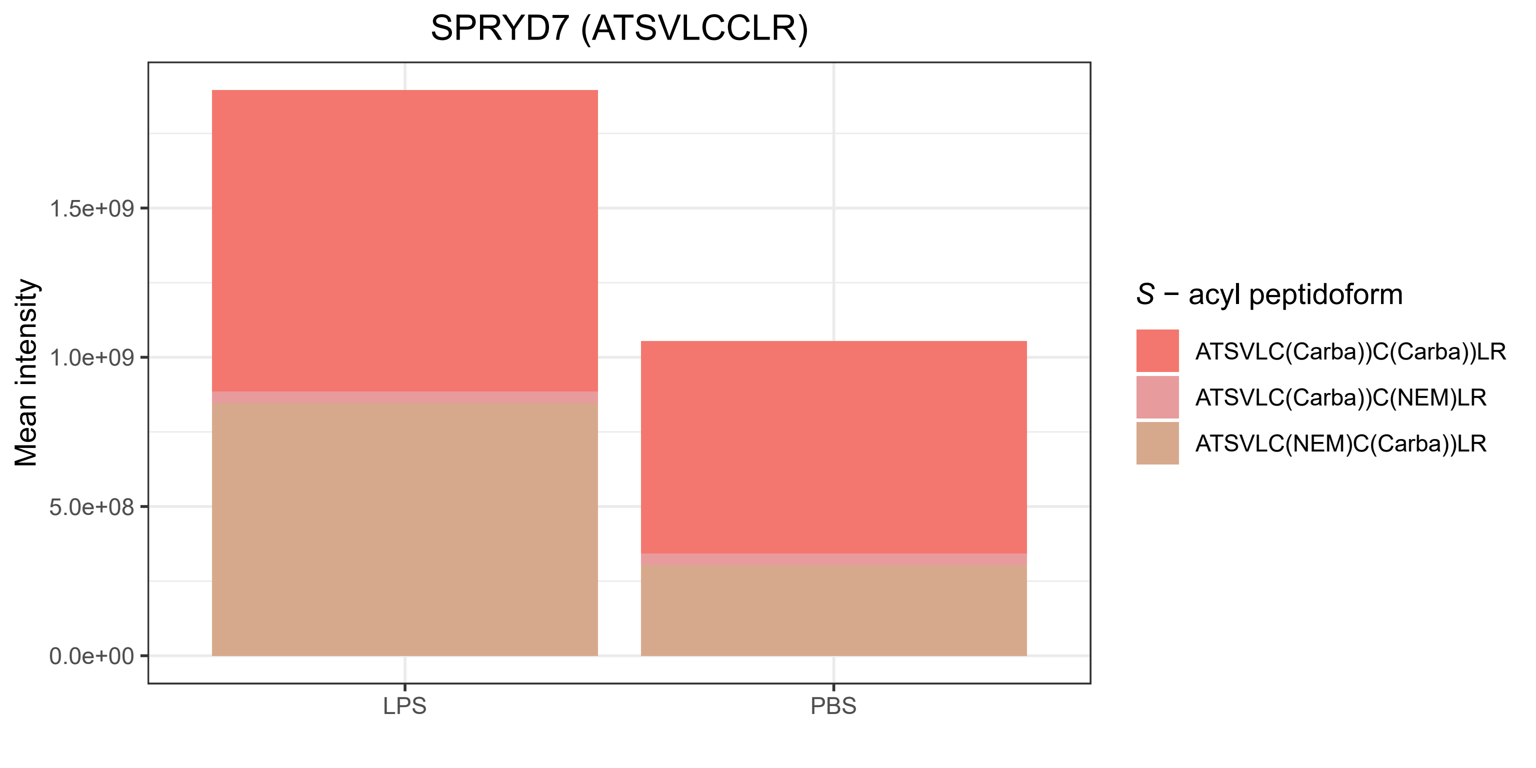

SPRYD7 (ATSVLCCLR)
Mean intensity
1.5e+09
1.0e+09
5.0e+08
0.0e+00
LPS
PBS
S − acyl peptidoform
ATSVLC(Carba))C(Carba))LR
ATSVLC(Carba))C(NEM)LR
ATSVLC(NEM)C(Carba))LR

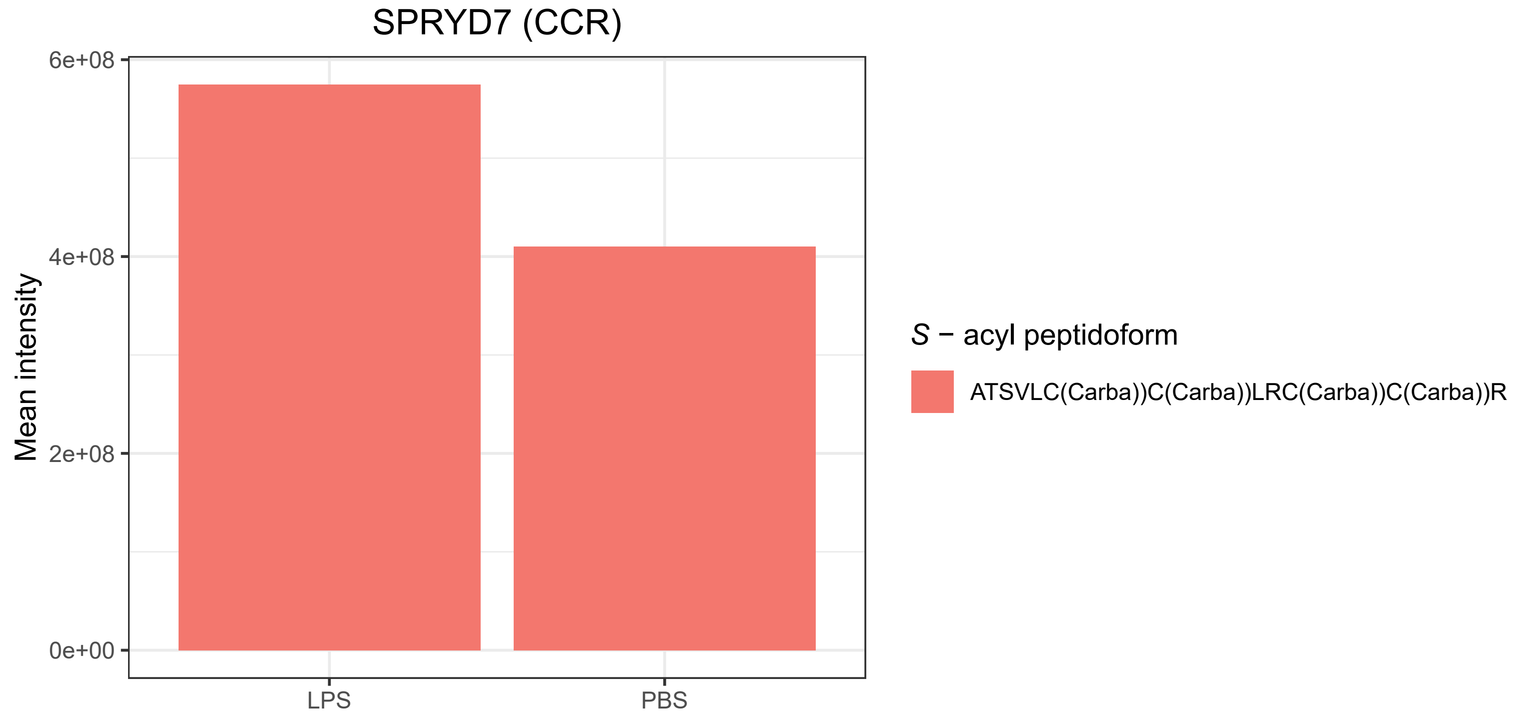

SPRYD7 (CCR)
Mean intensity
6e+08
4e+08
2e+08
0e+00
LPS
PBS
S − acyl peptidoform
ATSVLC(Carba))C(Carba))LRC(Carba))C(Carba))R

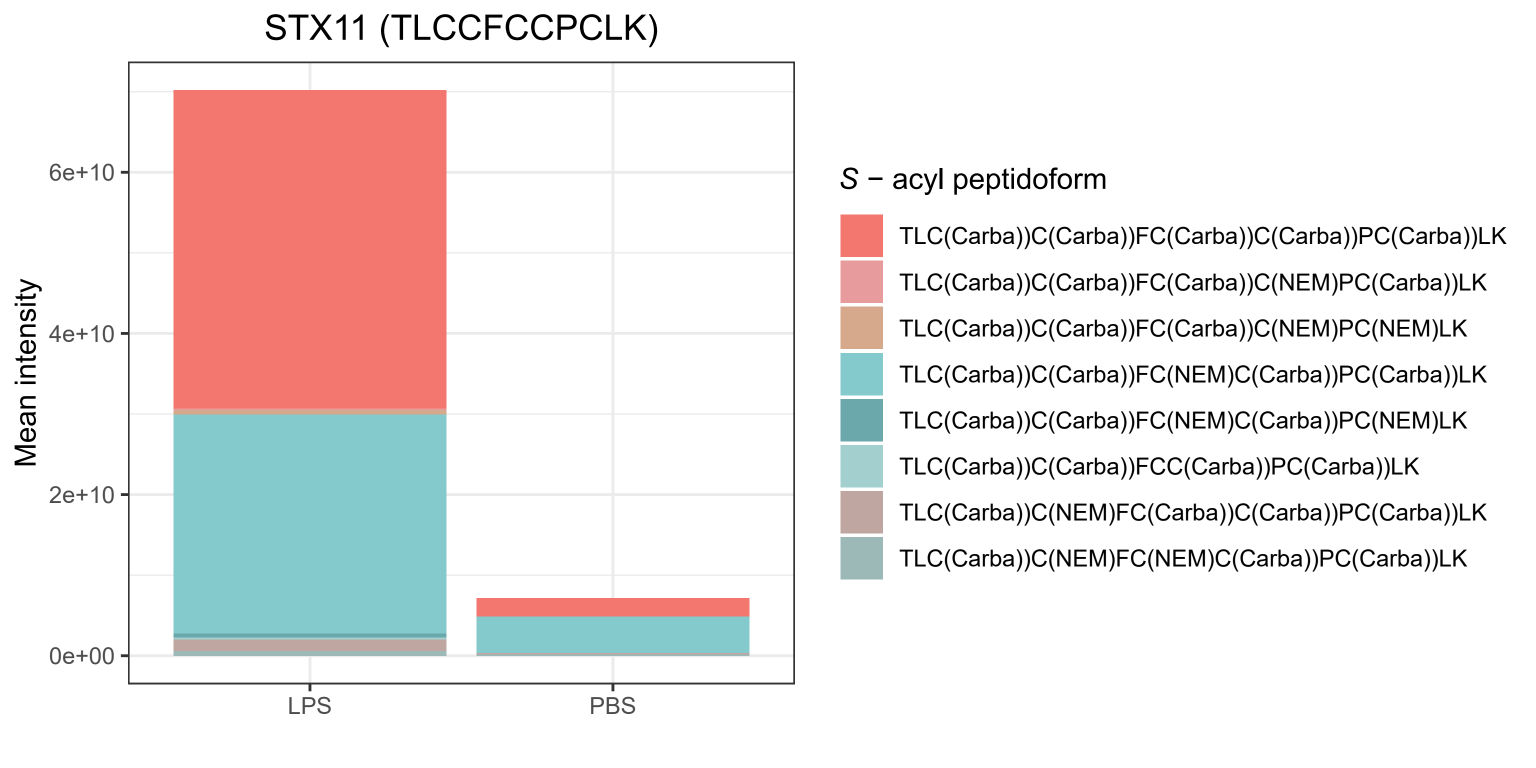
STX11 (TLCCFCCPCLK)
Mean intensity
6e+10
4e+10
2e+10
0e+00
LPS
PBS
S − acyl peptidoform
TLC(Carba))C(Carba))FC(Carba))C(Carba))PC(Carba))LK
TLC(Carba))C(Carba))FC(Carba))C(NEM)PC(Carba))LK
TLC(Carba))C(Carba))FC(Carba))C(NEM)PC(NEM)LK
TLC(Carba))C(Carba))FC(NEM)C(Carba))PC(Carba))LK
TLC(Carba))C(Carba))FC(NEM)C(Carba))PC(NEM)LK
TLC(Carba))C(Carba))FCC(Carba))PC(Carba))LK
TLC(Carba))C(NEM)FC(Carba))C(Carba))PC(Carba))LK
TLC(Carba))C(NEM)FC(NEM)C(Carba))PC(Carba))LK

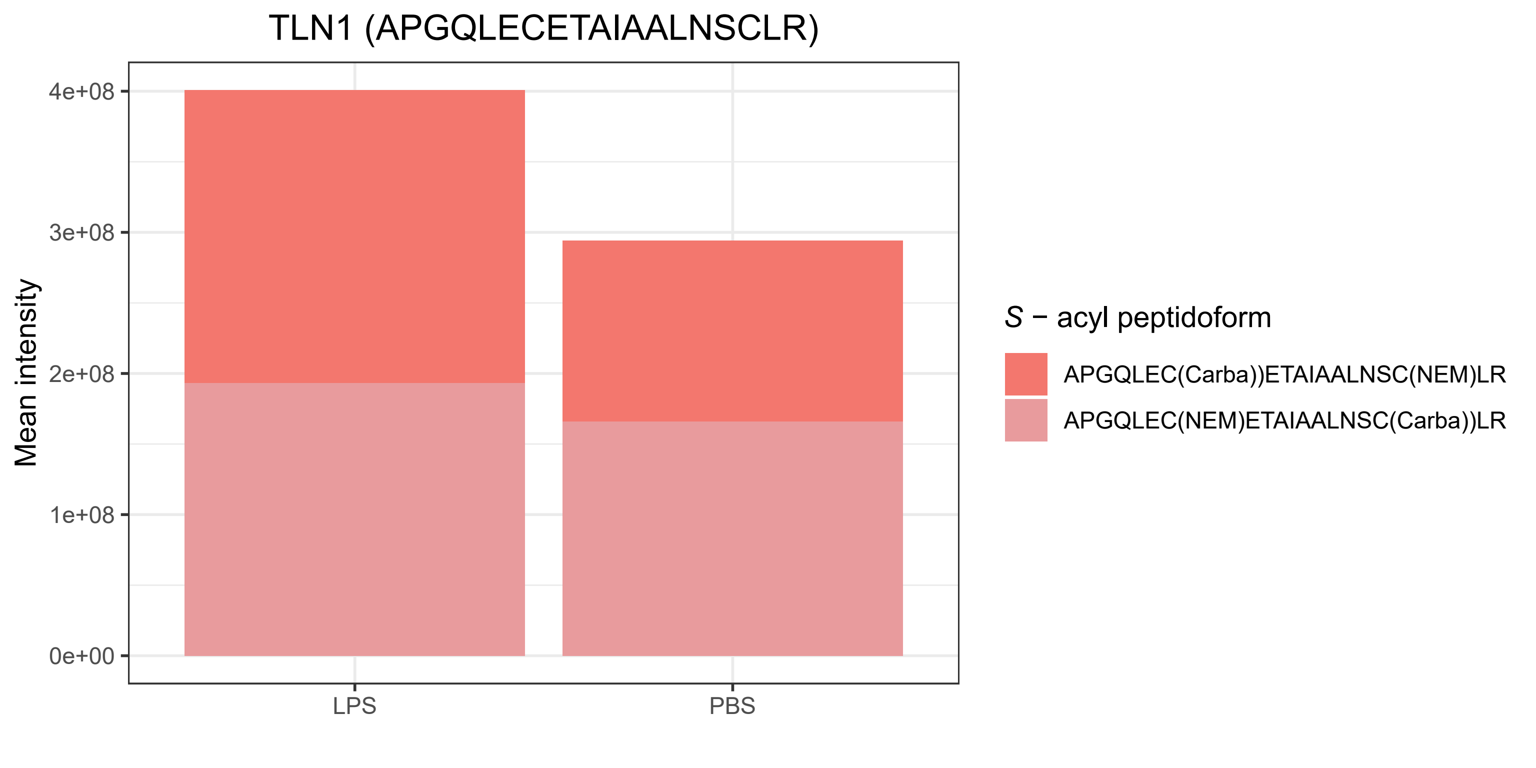

TLN1 (APGQLECETAIAALNSCLR)
Mean intensity
4e+08
3e+08
2e+08
1e+08
0e+00
LPS
PBS
S − acyl peptidoform
APGQLEC(Carba))ETAIAALNSC(NEM)LR
APGQLEC(NEM)ETAIAALNSC(Carba))LR

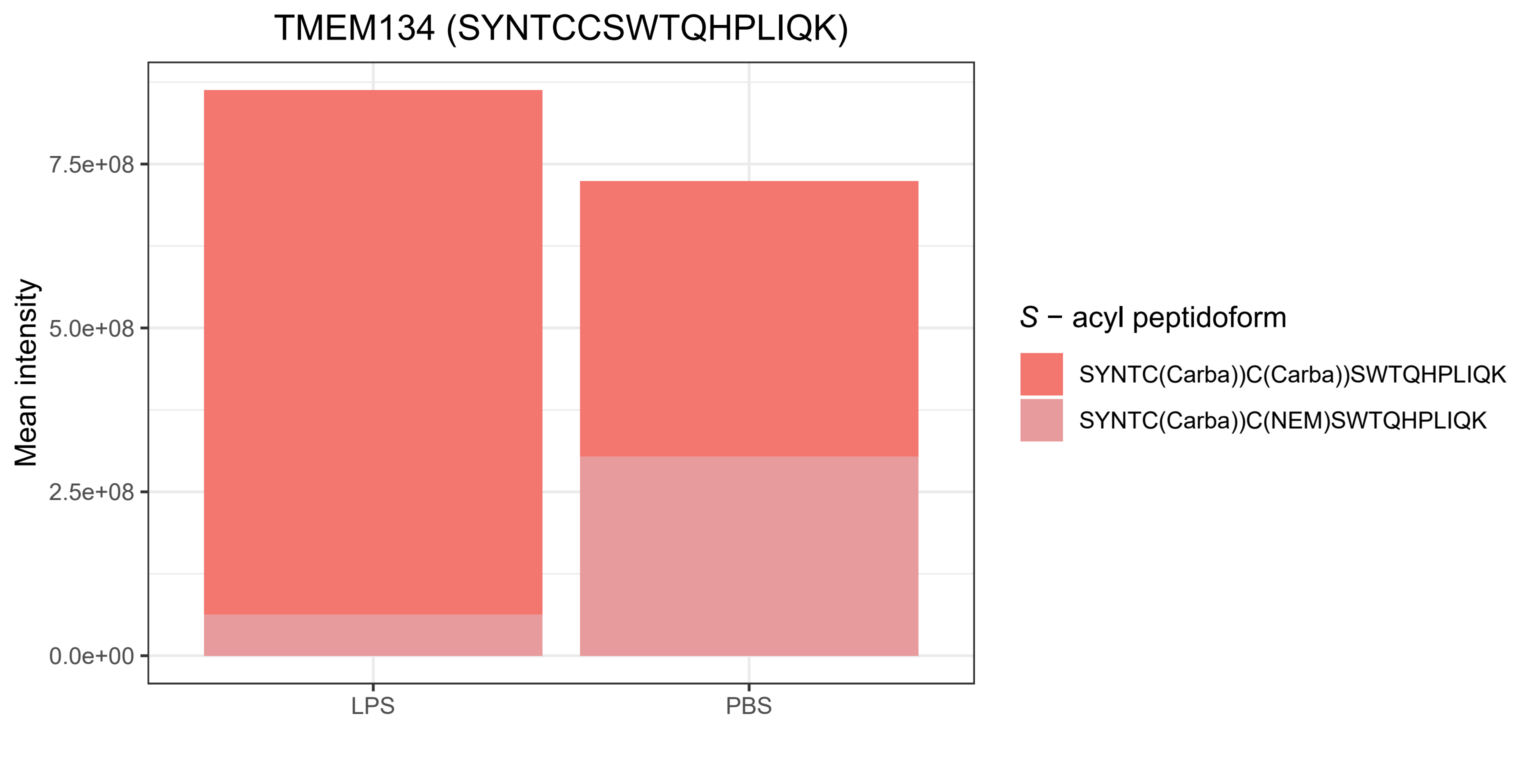
TMEM134 (SYNTCCSWTQHPLIQK)
Mean intensity
7.5e+08
5.0e+08
2.5e+08
0.0e+00
LPS
PBS
S − acyl peptidoform
SYNTC(Carba))C(Carba))SWTQHPLIQK
SYNTC(Carba))C(NEM)SWTQHPLIQK

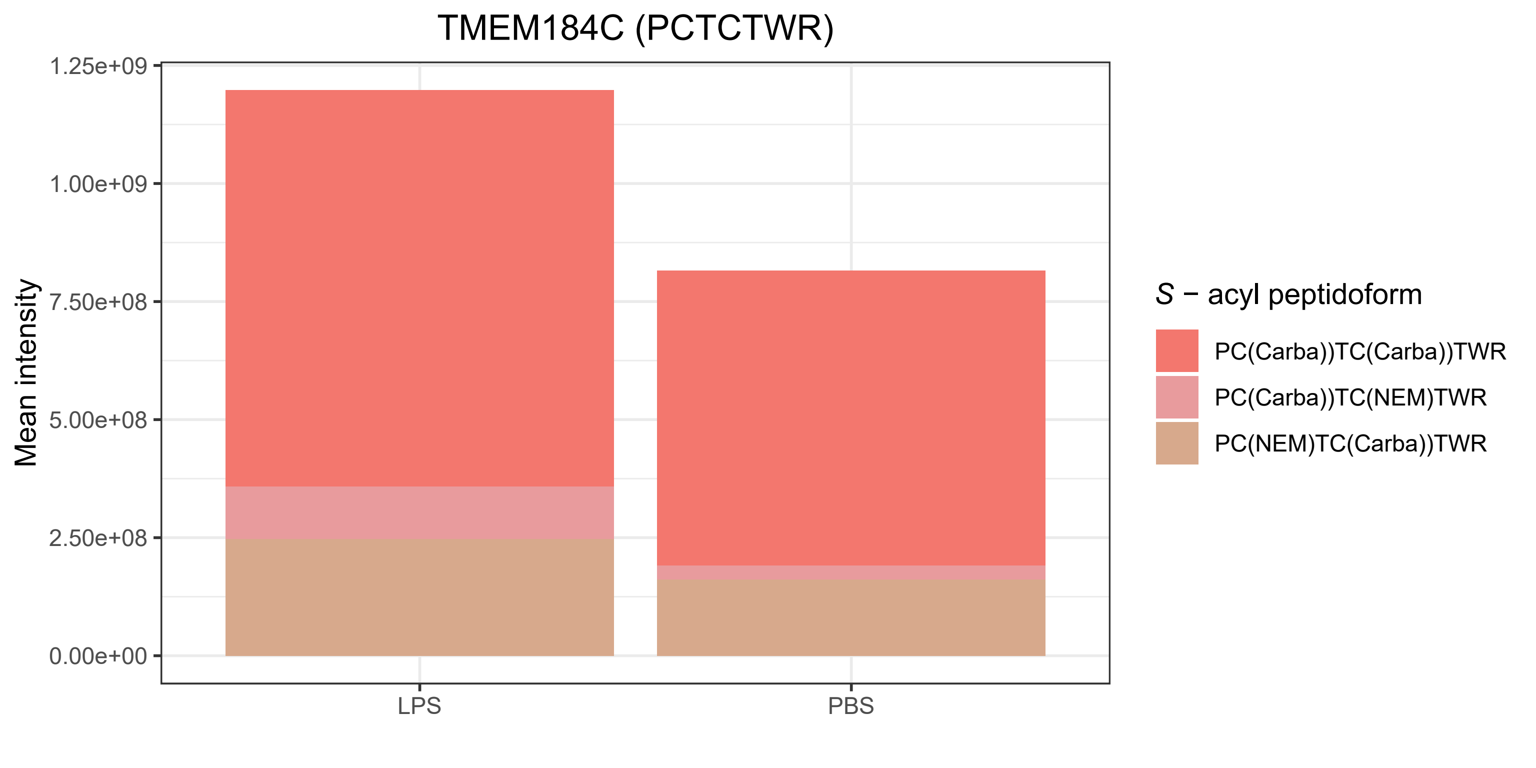
TMEM184C (PCTCTWR)
Mean intensity
1.25e+09
1.00e+09
7.50e+08
5.00e+08
2.50e+08
0.00e+00
LPS
PBS
S − acyl peptidoform
PC(Carba))TC(Carba))TWR
PC(Carba))TC(NEM)TWR
PC(NEM)TC(Carba))TWR

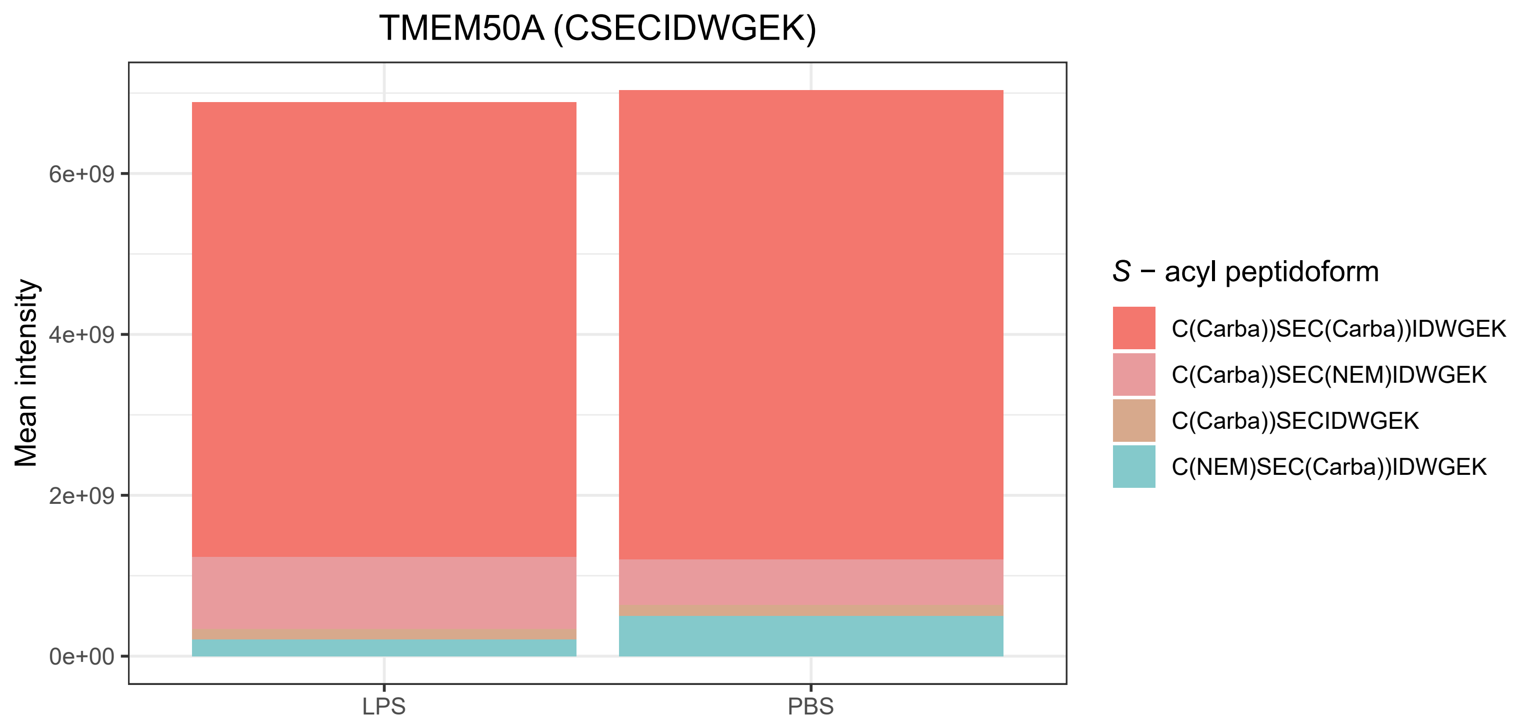
TMEM50A (CSECIDWGEK)
Mean intensity
6e+09
4e+09
2e+09
0e+00
LPS
PBS
S − acyl peptidoform
C(Carba))SEC(Carba))IDWGEK
C(Carba))SEC(NEM)IDWGEK
C(Carba))SECIDWGEK
C(NEM)SEC(Carba))IDWGEK

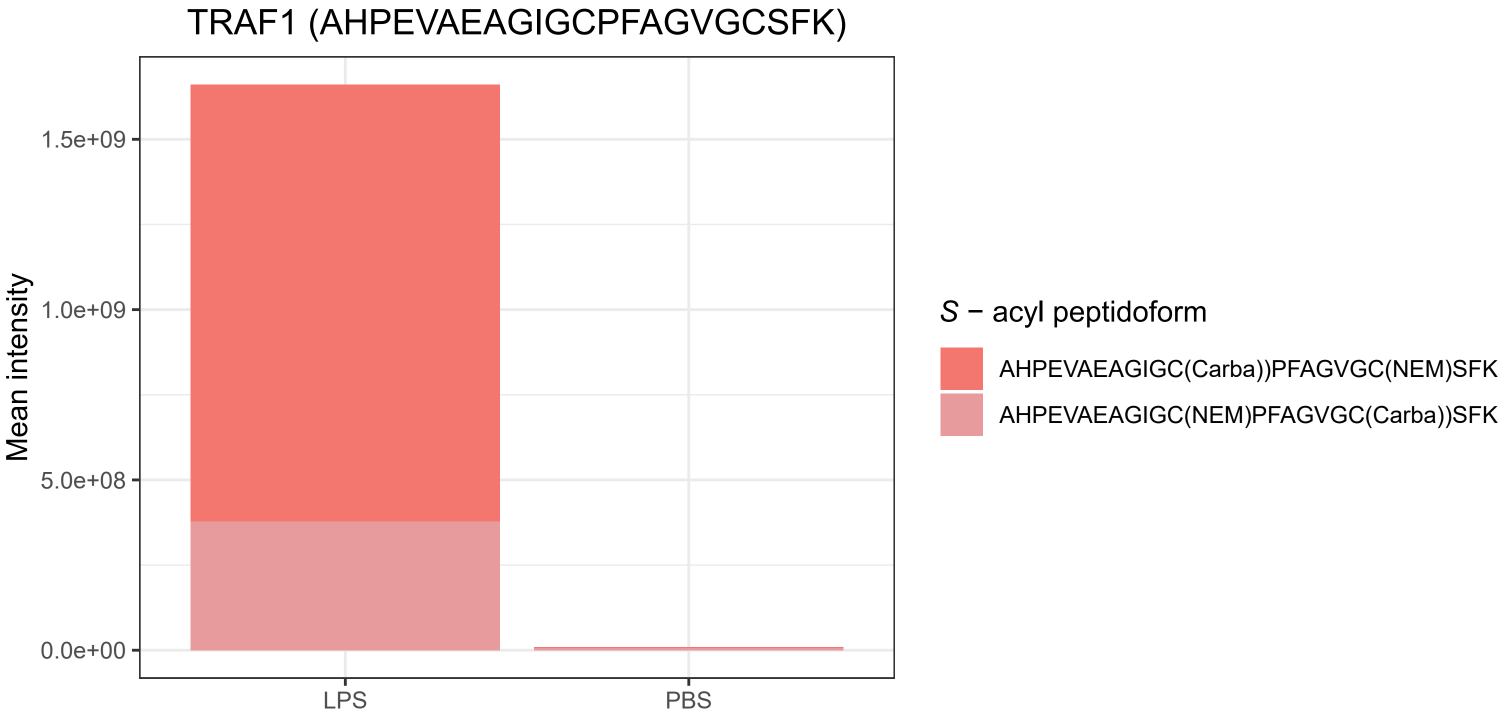

TRAF1 (AHPEVAEAGIGCPFAGVGCSFK)
Mean intensity
1.5e+09
1.0e+09
5.0e+08
0.0e+00
LPS
PBS
S − acyl peptidoform
AHPEVAEAGIGC(Carba))PFAGVGC(NEM)SFK
AHPEVAEAGIGC(NEM)PFAGVGC(Carba))SFK

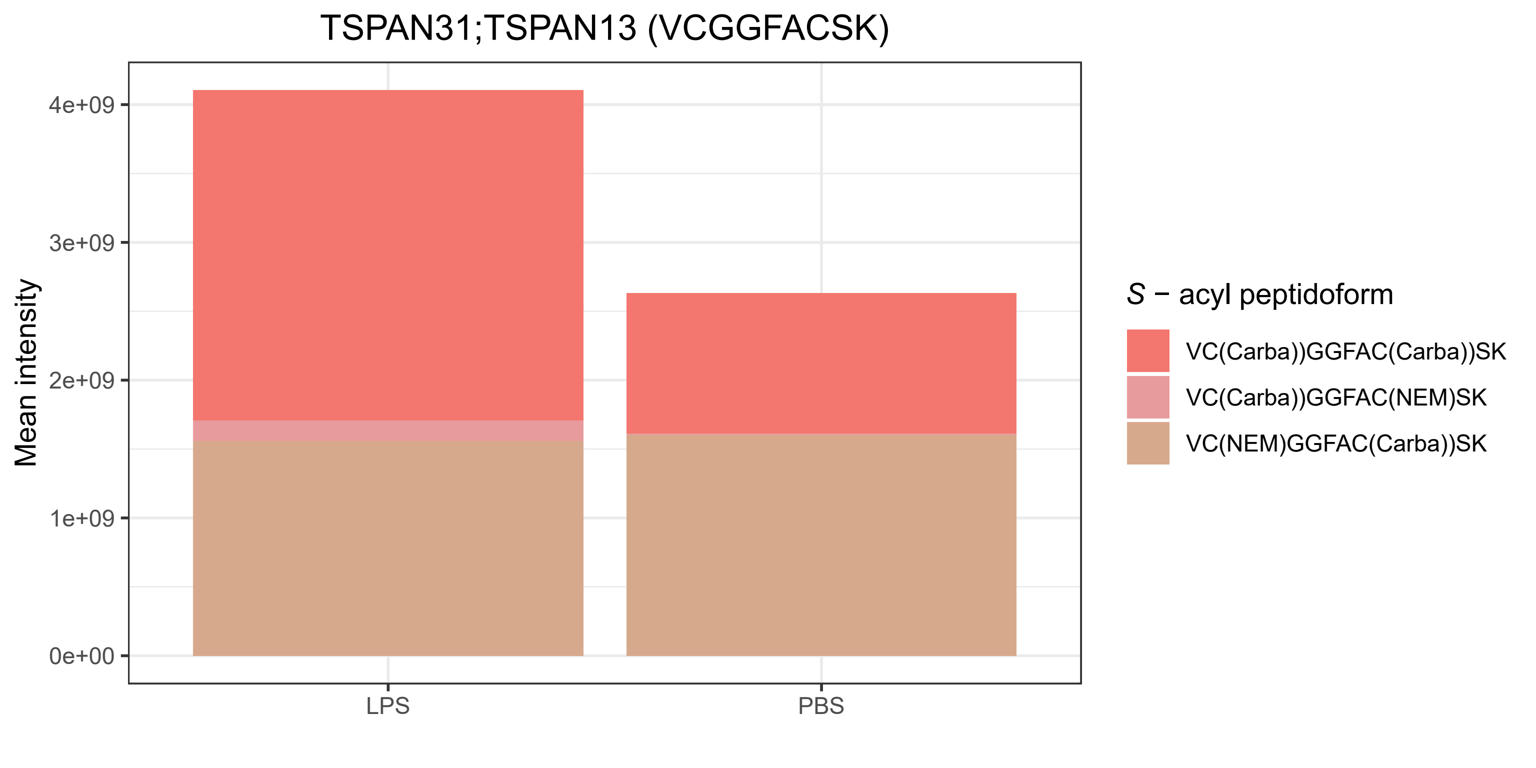

TSPAN31;TSPAN13 (VCGGFACSK)
Mean intensity
4e+09
3e+09
2e+09
1e+09
0e+00
LPS
PBS
S − acyl peptidoform
VC(Carba))GGFAC(Carba))SK
VC(Carba))GGFAC(NEM)SK
VC(NEM)GGFAC(Carba))SK

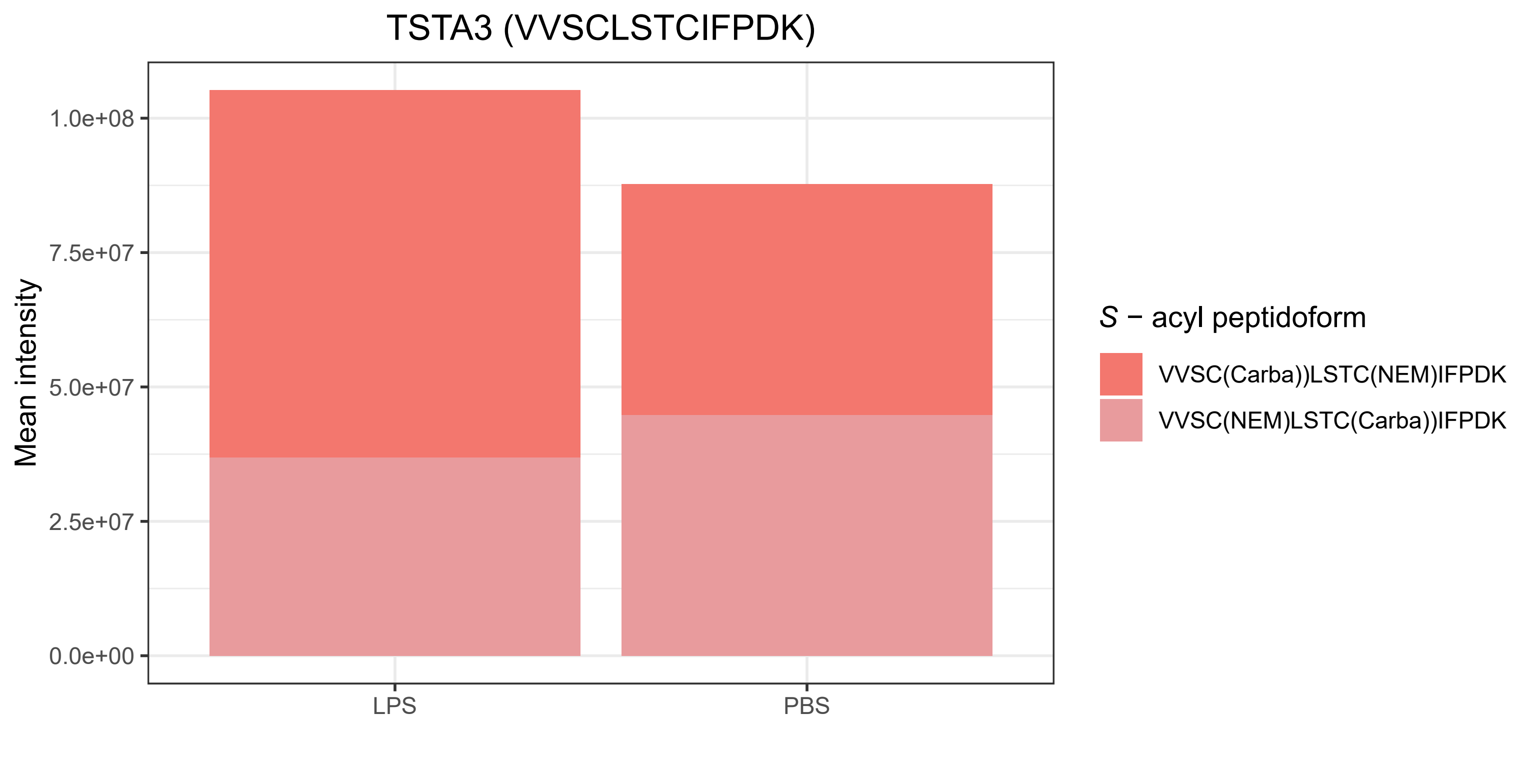
TSTA3 (VVSCLSTCIFPDK)
Mean intensity
1.0e+08
7.5e+07
5.0e+07
2.5e+07
0.0e+00
LPS
PBS
S − acyl peptidoform
VVSC(Carba))LSTC(NEM)IFPDK
VVSC(NEM)LSTC(Carba))IFPDK

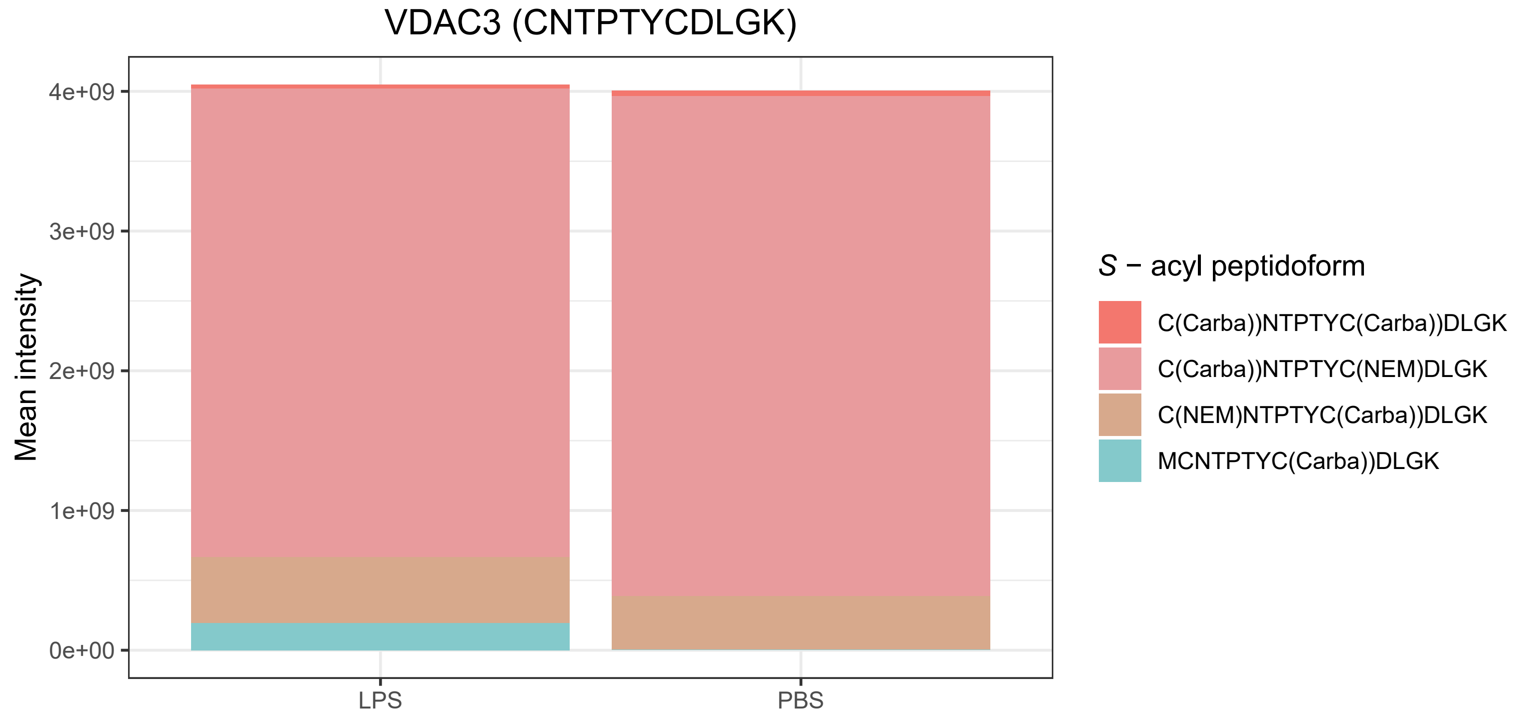
VDAC3 (CNTPTYCDLGK)
Mean intensity
4e+09
3e+09
2e+09
1e+09
0e+00
LPS
PBS
S − acyl peptidoform
C(Carba))NTPTYC(Carba))DLGK
C(Carba))NTPTYC(NEM)DLGK
C(NEM)NTPTYC(Carba))DLGK
MCNTPTYC(Carba))DLGK

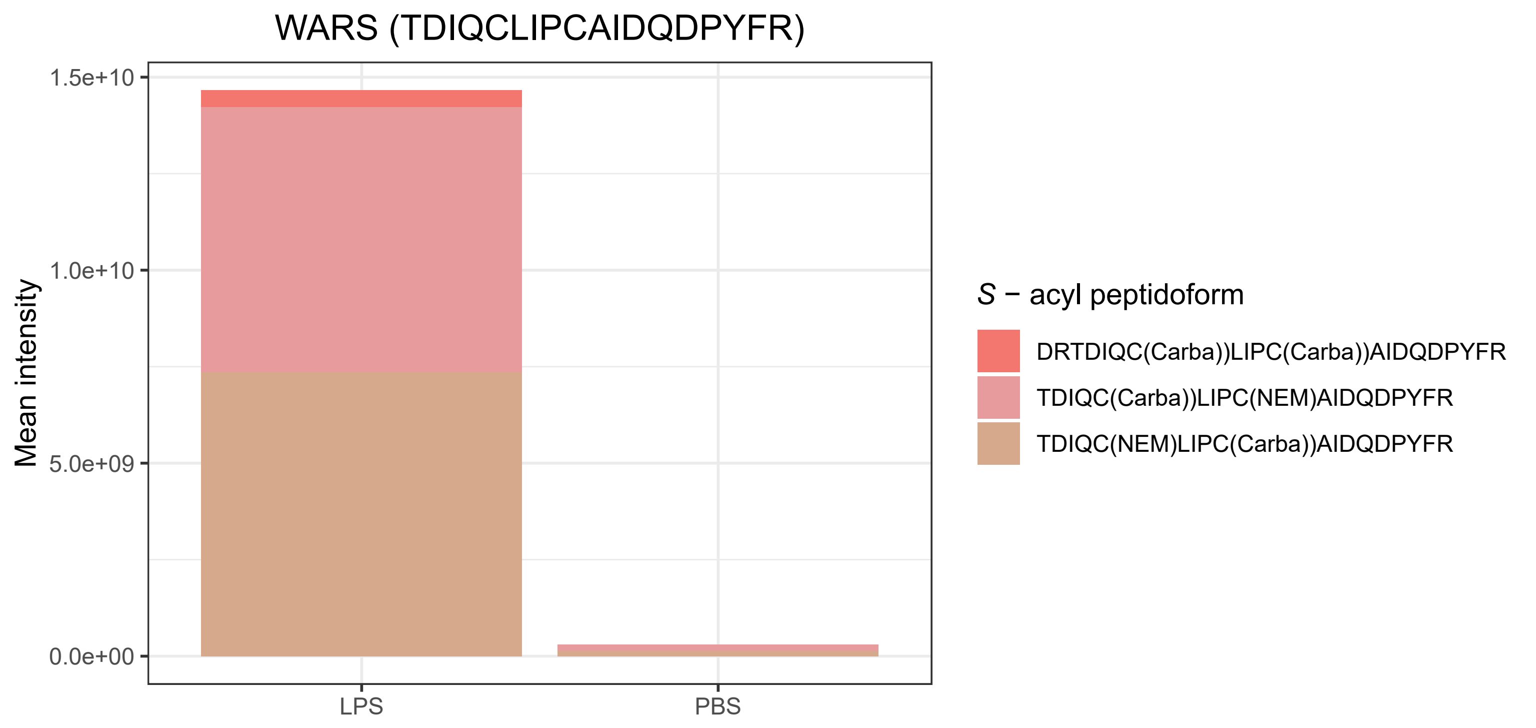
WARS (TDIQCLIPCAIDQDPYFR)
Mean intensity
1.5e+10
1.0e+10
5.0e+09
0.0e+00
LPS
PBS
S − acyl peptidoform
DRTDIQC(Carba))LIPC(Carba))AIDQDPYFR
TDIQC(Carba))LIPC(NEM)AIDQDPYFR
TDIQC(NEM)LIPC(Carba))AIDQDPYFR

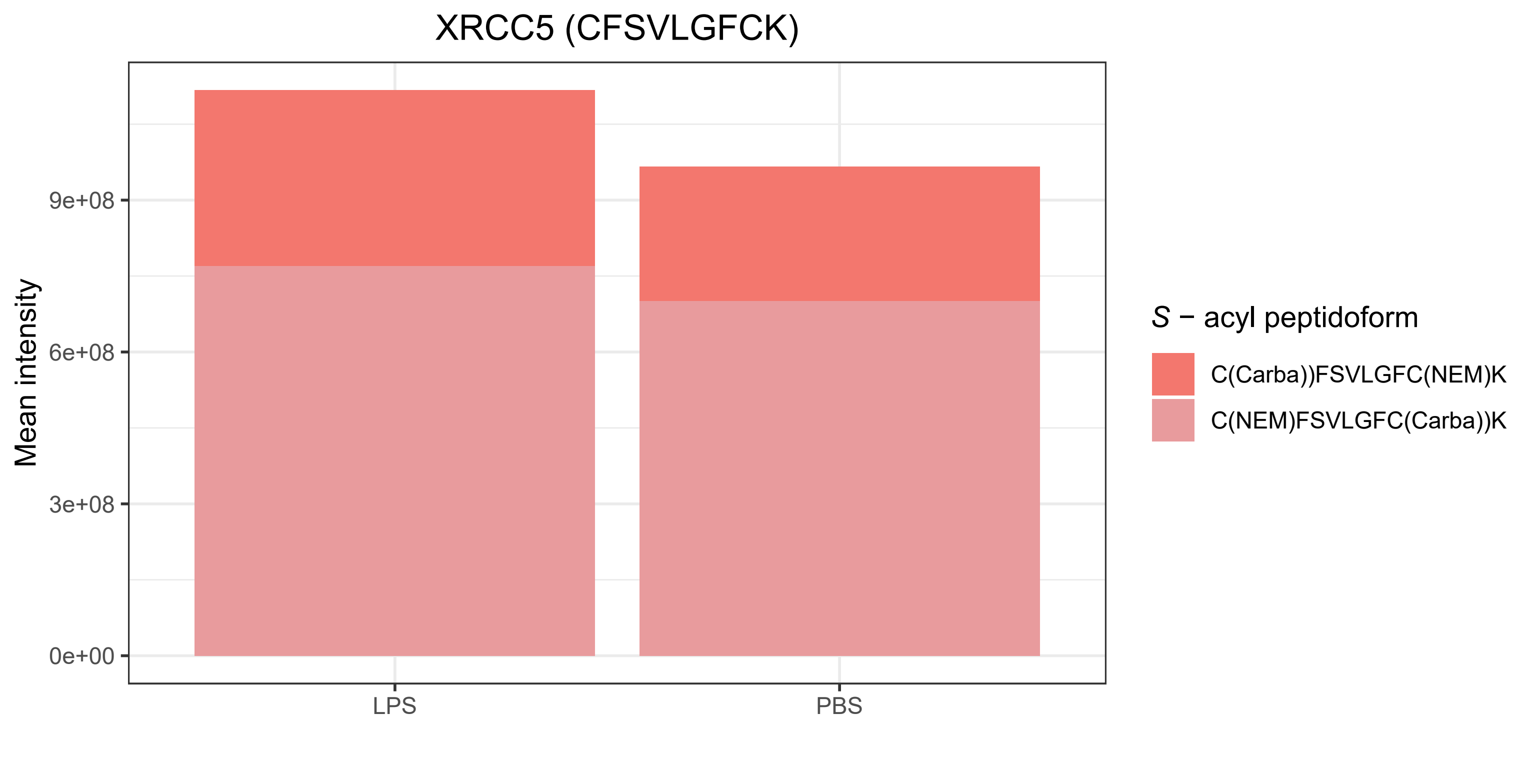

XRCC5 (CFSVLGFCK)
Mean intensity
9e+08
6e+08
3e+08
0e+00
LPS
PBS
S − acyl peptidoform
C(Carba))FSVLGFC(NEM)K
C(NEM)FSVLGFC(Carba))K

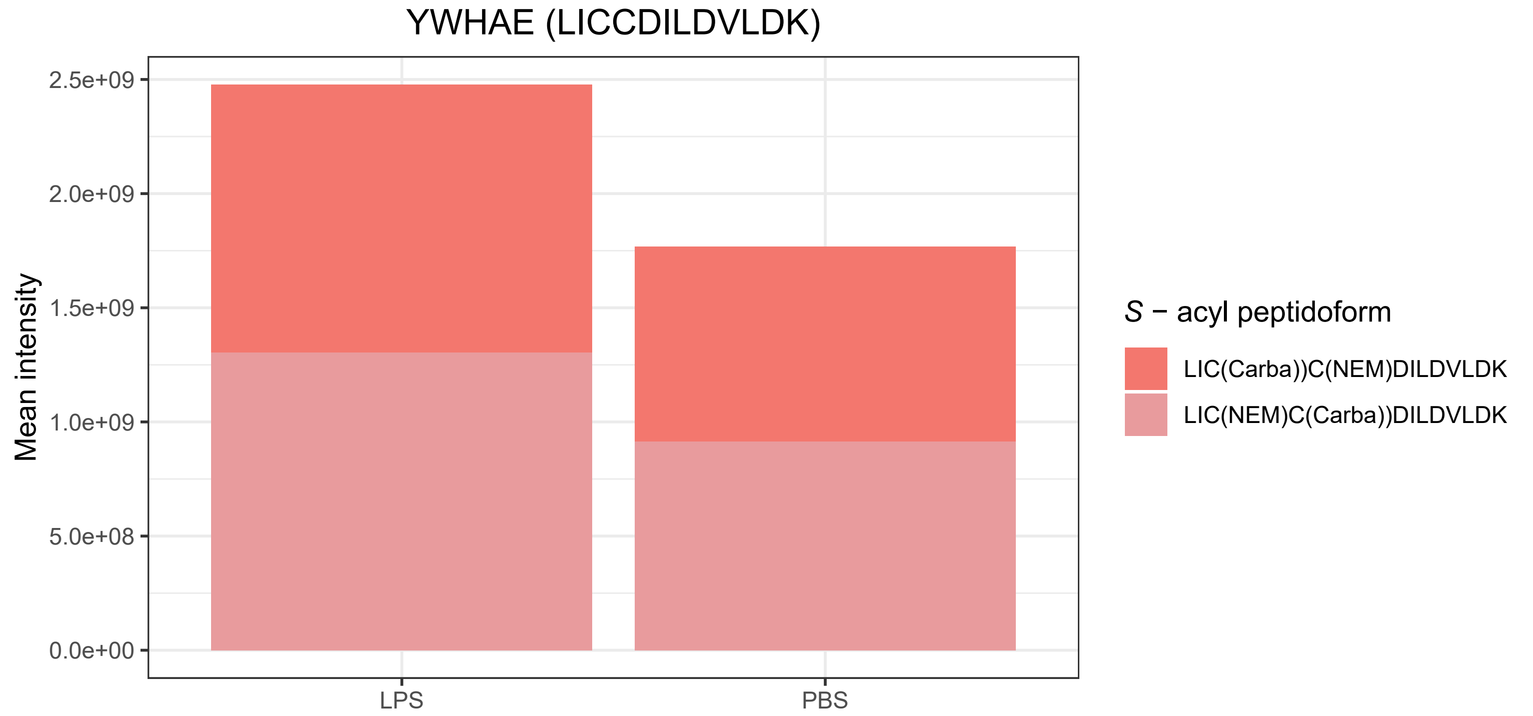

YWHAE (LICCDILDVLDK)
Mean intensity
2.5e+09
2.0e+09
1.5e+09
1.0e+09
5.0e+08
0.0e+00
LPS
PBS
S − acyl peptidoform
LIC(Carba))C(NEM)DILDVLDK
LIC(NEM)C(Carba))DILDVLDK

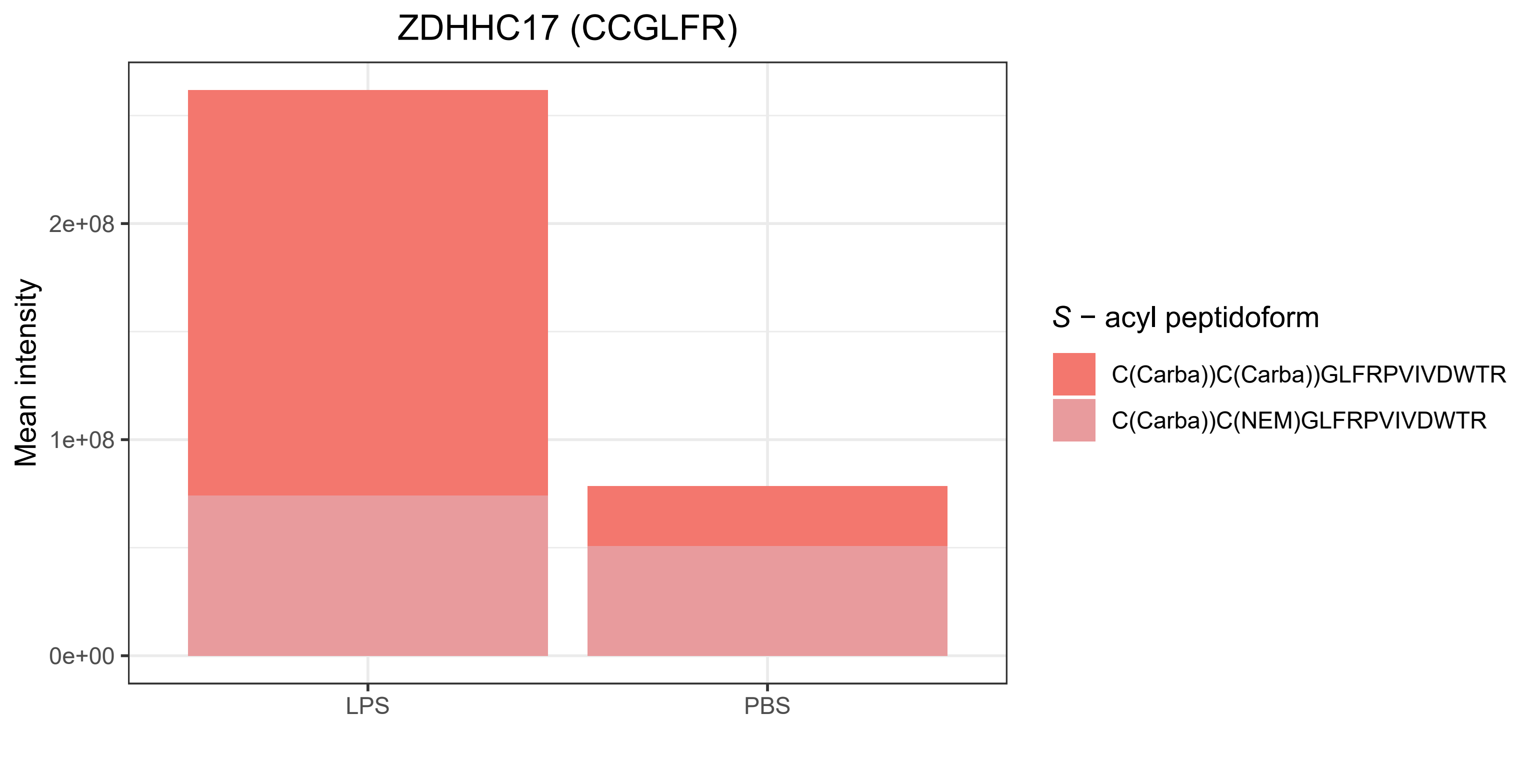

ZDHHC17 (CCGLFR)
Mean intensity
2e+08
1e+08
0e+00
LPS
PBS
S − acyl peptidoform
C(Carba))C(Carba))GLFRPVIVDWTR
C(Carba))C(NEM)GLFRPVIVDWTR

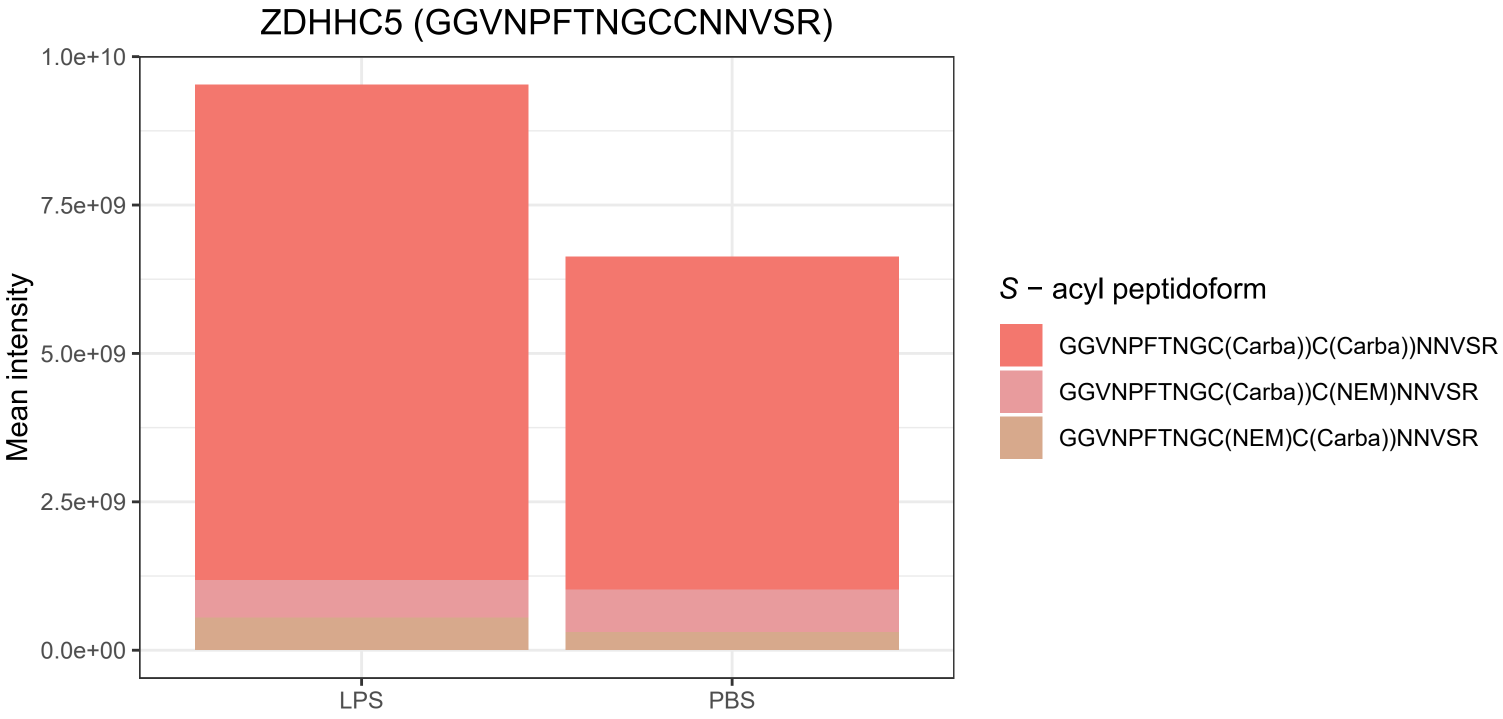
ZDHHC5 (GGVNPFTNGCCNNVSR)
1.0e+10
7.5e+09
5.0e+09
2.5e+09
0.0e+00
Mean intensity
LPS
PBS
S − acyl peptidoform
GGVNPFTNGC(Carba))C(Carba))NNVSR
GGVNPFTNGC(Carba))C(NEM)NNVSR
GGVNPFTNGC(NEM)C(Carba))NNVSR

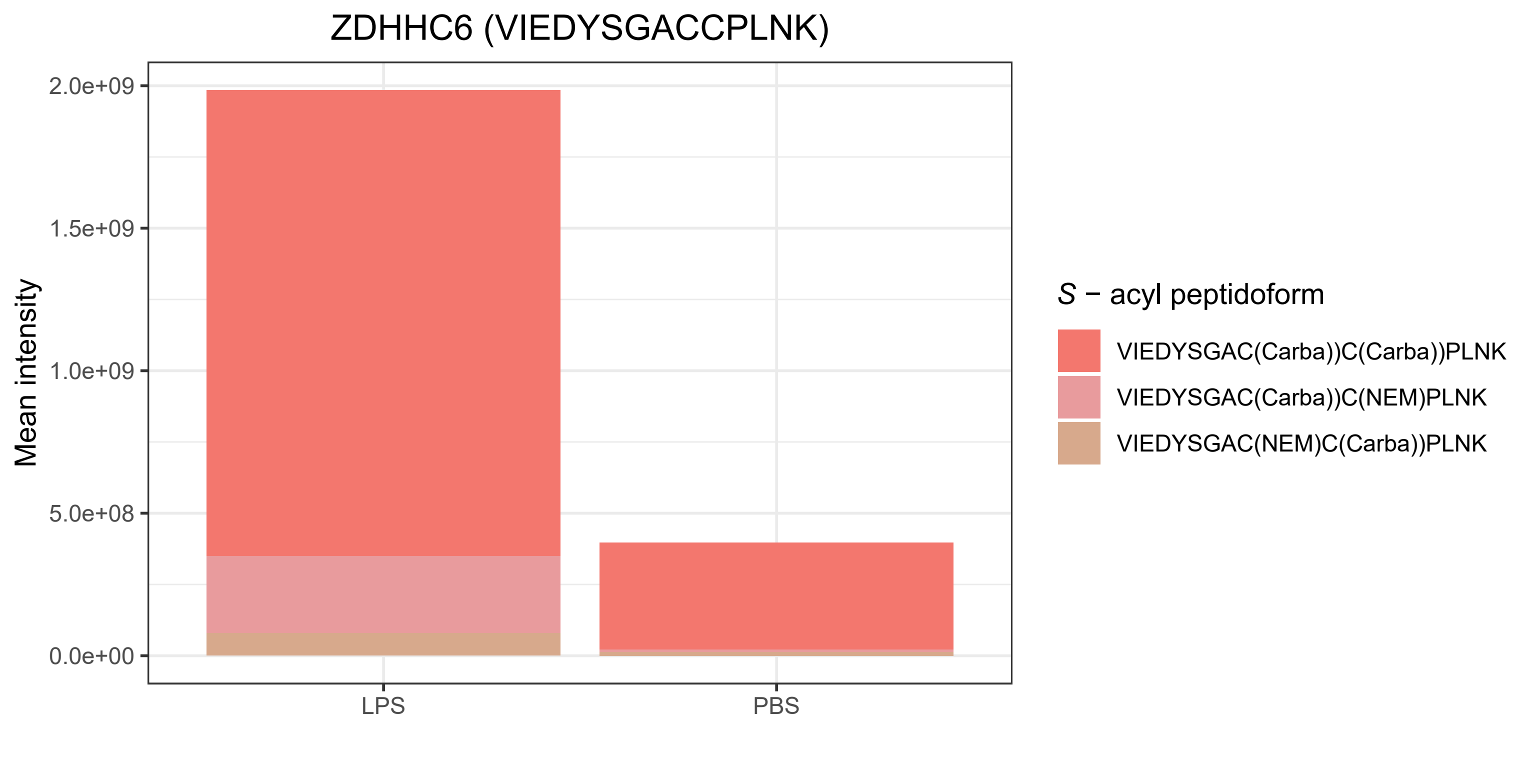

ZDHHC6 (VIEDYSGACCPLNK)
Mean intensity
2.0e+09
1.5e+09
1.0e+09
5.0e+08
0.0e+00
LPS
PBS
S − acyl peptidoform
VIEDYSGAC(Carba))C(Carba))PLNK
VIEDYSGAC(Carba))C(NEM)PLNK
VIEDYSGAC(NEM)C(Carba))PLNK

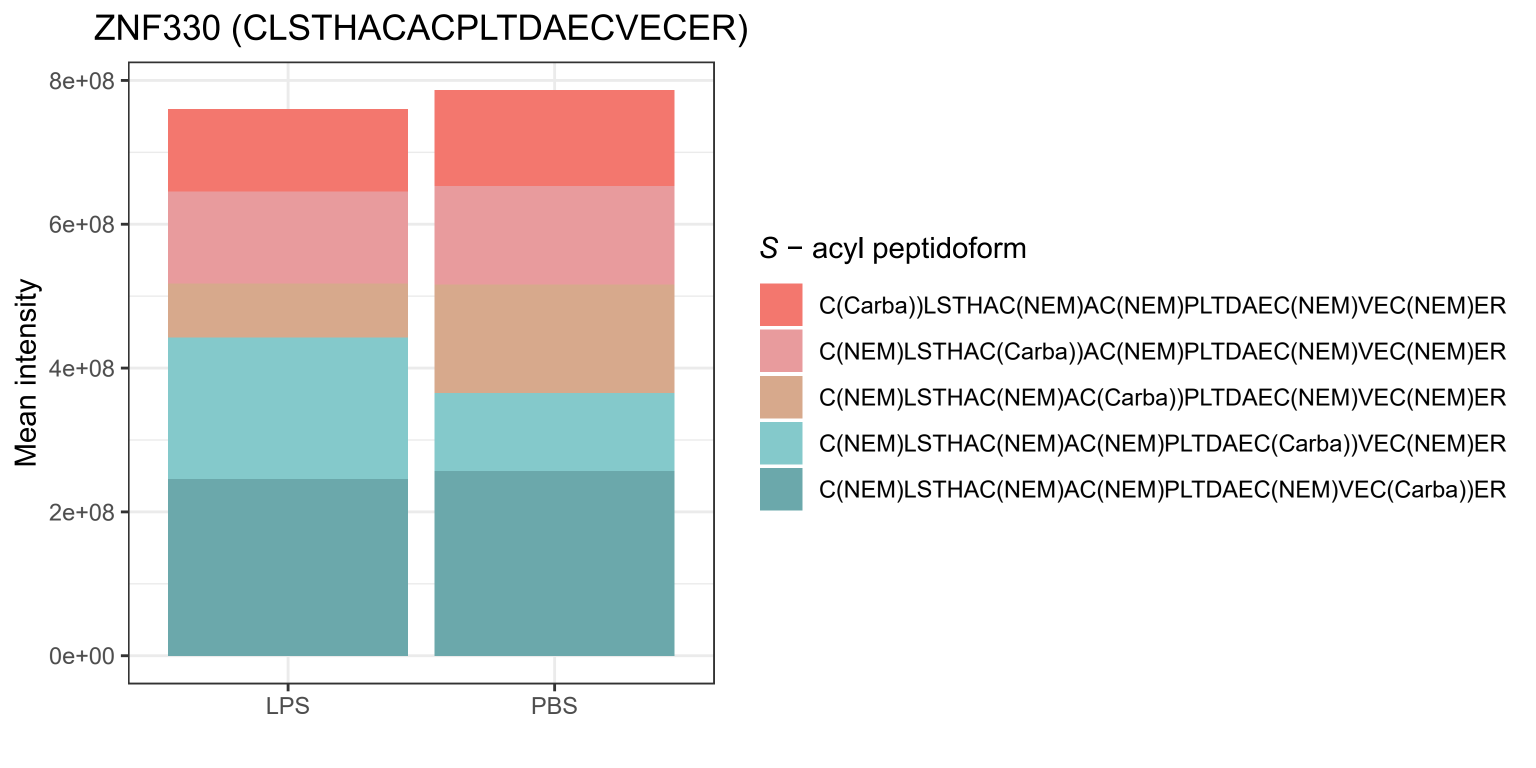
ZNF330 (CLSTHACACPLTDAECVECER)
Mean intensity
8e+08
6e+08
4e+08
2e+08
0e+00
LPS
PBS
S − acyl peptidoform
C(Carba))LSTHAC(NEM)AC(NEM)PLTDAEC(NEM)VEC(NEM)ER
C(NEM)LSTHAC(Carba))AC(NEM)PLTDAEC(NEM)VEC(NEM)ER
C(NEM)LSTHAC(NEM)AC(Carba))PLTDAEC(NEM)VEC(NEM)ER
C(NEM)LSTHAC(NEM)AC(NEM)PLTDAEC(Carba))VEC(NEM)ER
C(NEM)LSTHAC(NEM)AC(NEM)PLTDAEC(NEM)VEC(Carba))ER

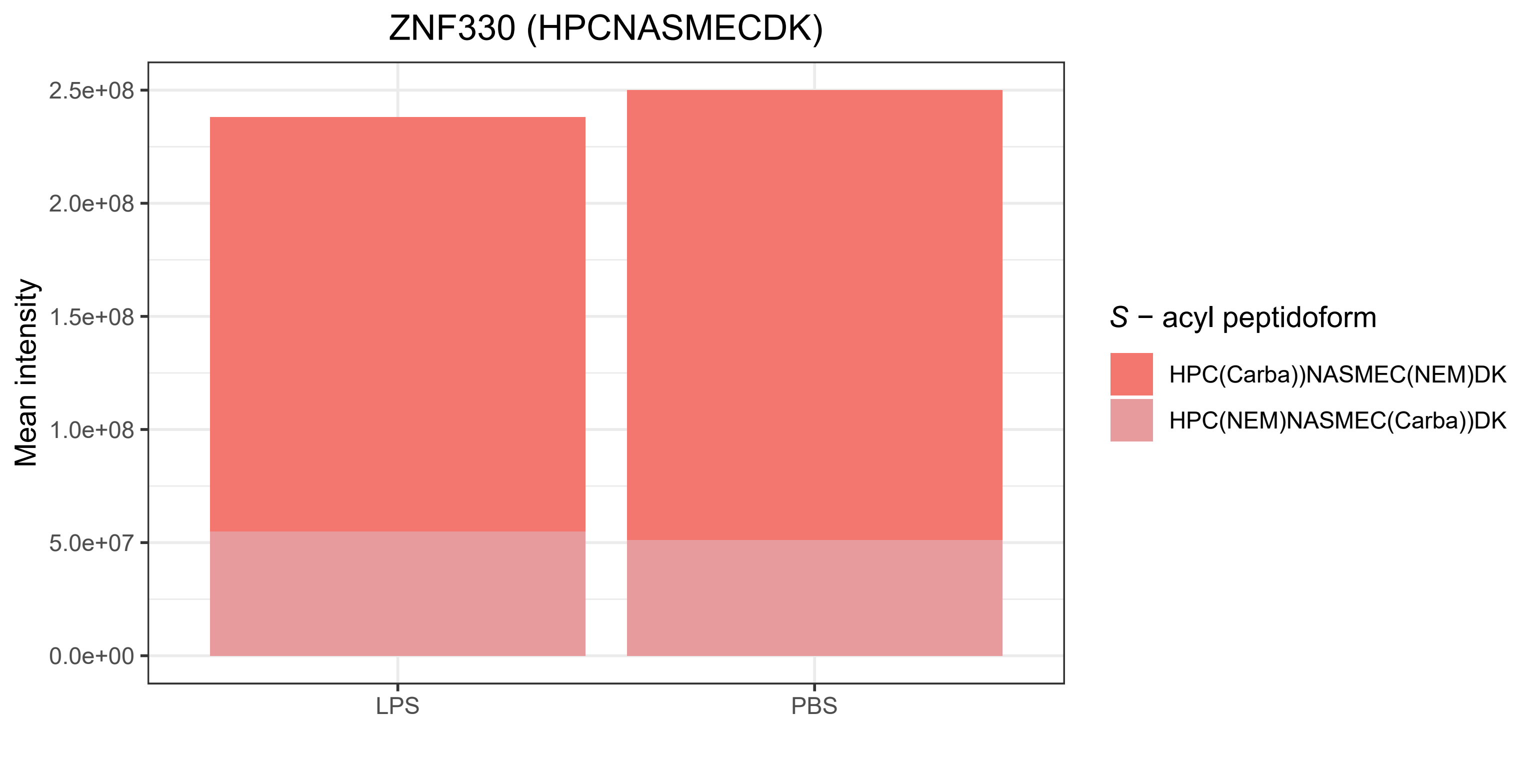

ZNF330 (HPCNASMECDK)
Mean intensity
2.5e+08
2.0e+08
1.5e+08
1.0e+08
5.0e+07
0.0e+00
LPS
PBS
S − acyl peptidoform
HPC(Carba))NASMEC(NEM)DK
HPC(NEM)NASMEC(Carba))DK
