# Supplementary material for: Long-Chain S-Acylation Is a Key Modulator During the Macrophage Inflammatory Response
Source: Mol Cell Proteomics. 2026 Jun 10;25(7):101600. doi: 10.1016/j.mcpro.2026.101600 (PMC13380729; doi:10.1016/j.mcpro.2026.101600)
Supplement: Supplemental Figures [file mmc3.pdf]

Supporting information

Long-chain *S*-acylation is a key modulator during the macrophage inflammatory response

Anneroos E. Nederstigt[1,2], Francine Rodrigues Ianiski[1,2], Samiksha Sardana[1,2], M.P. Baggelaar[1,2,*]

[1.] Biomolecular Mass Spectrometry and Proteomics, Bijvoet Center for Biomolecular Research and Utrecht Institute for Pharmaceutical Sciences, University of Utrecht, Padualaan 8, Utrecht 3584 CH, The Netherlands
[2.] Netherlands Proteomics Center, Padualaan 8, Utrecht 3584 CH, The Netherlands

[*] Contact details for correspondence: m.p.baggelaar@uu.nl

TABLE OF CONTENTS

Supporting figure 1. Heavy label incorporation in THP-1 peptides. 2
Supporting figure 2. Label-free differentiation experiment to evaluate ssABE workflow. 3
Supporting figure 3. Comparison of long-chain *S*-acylation sites detected in benchmark list with literature studies. 4
Supporting figure 4. Reproducibility metrics for label-free differentiation experiment. 5
Supporting figure 5. Summary of imputation for label-free differentiation experiment. 7
Supporting figure 6. SILAC total proteome replicate overview. 8
Supporting figure 7. SILAC total proteome 3D volcano plot. 9
Supporting figure 8. Sankey plot of proteins enriched in M(LPS+IFNɣ) polarized macrophages that belong to the top 7 enriched biological process GO terms. 10
Supporting figure 9. SILAC ssABE replicate and site overview. 11
Supporting figure 10. Simplified graphic: long-chain *S*-acylation mediated attenuation of purinoceptor 7 ATP desensitization. 13
Supporting figure 11. 81 long-chain *S*-acylated peripheral membrane proteins. 14
Supporting figure 12. Long-chain *S*-acylation sites relative to transmembrane domains.........15
Supporting figure 13. *S*-acyl-peptidoforms. 21
Supporting figure 14. Comparative secretome, proteome, and *S*-acyl-proteome analysis of PalmB/2-BP inhibited inflammatory macrophages. 23
Supporting figure 15. Mass spectra of detected peptides with endogenous protein N-terminal glycine myristoylation. 25

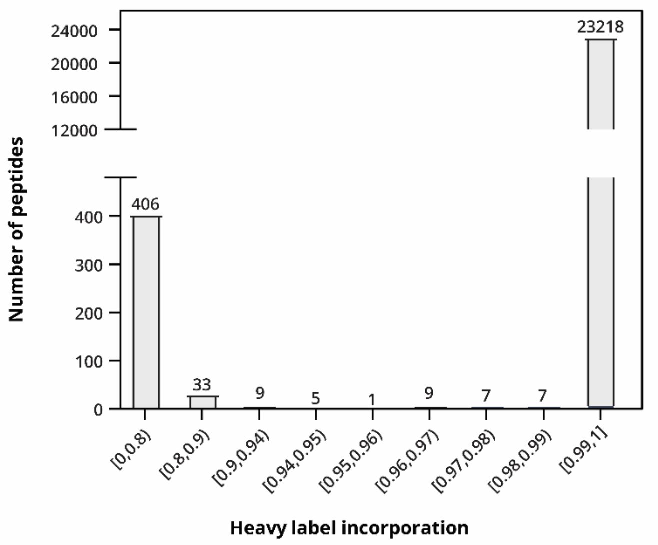

Supporting figure 1. Heavy label incorporation in THP-1 peptides.

THP-1 cells were cultured for ~2 weeks with $^{8}K^{10}R$ heavy medium. 20 µg of heavy proteome was processed by S-trap. For every detected peptide, the heavy intensity was divided by the sum of the heavy and light intensity, yielding a ratio (heavy label incorporation). Ratios were grouped in intervals (*x*-axis). *Y*-axis shows the number of peptides per ratio interval. ~98% of detected peptides had a heavy label incorporation between 0.99 and 1.

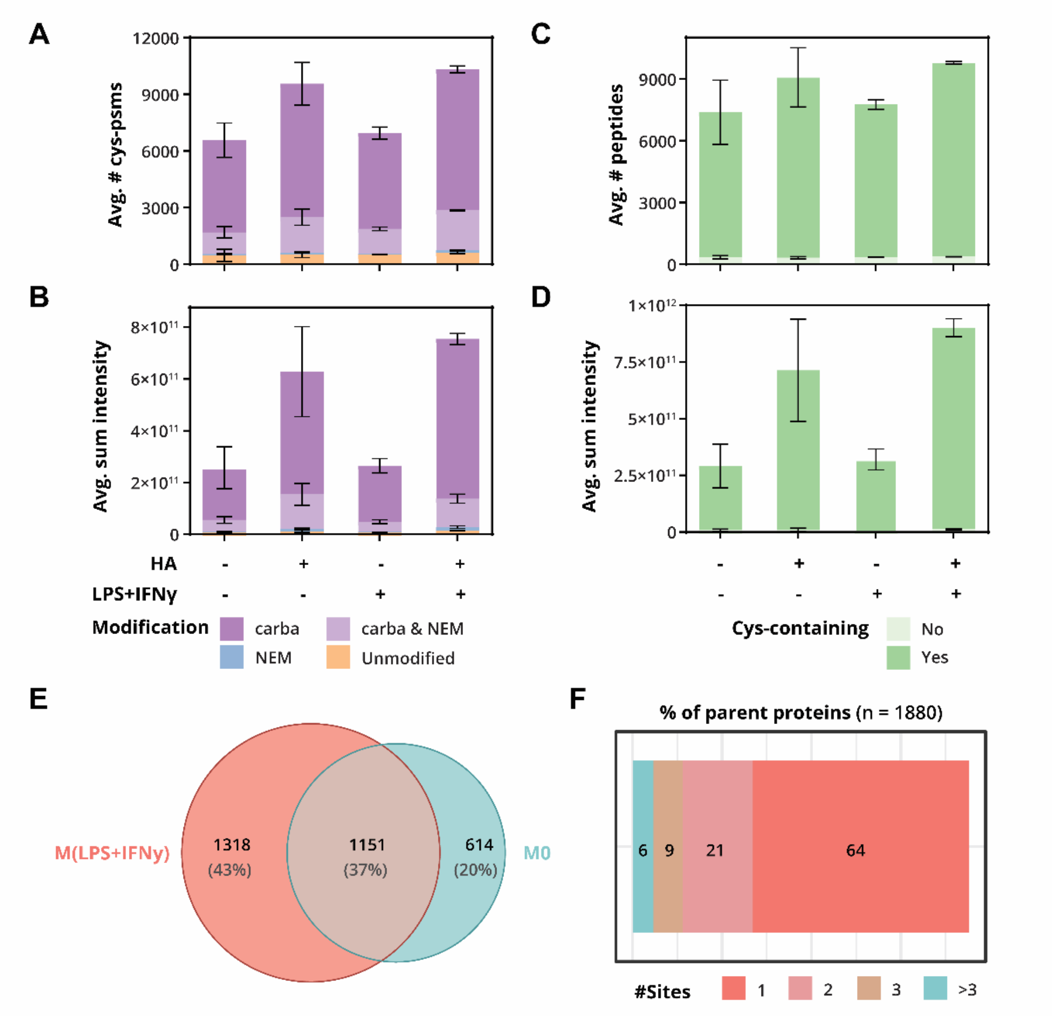

Supporting figure 2. Label-free differentiation experiment to evaluate ssABE workflow.

Label-free differentiation experiment to evaluate ssABE workflow. **(A-B)** Average cysteine carbamidomethylation efficiency of cysteine-containing PSMs in terms of **(A)** count and (**C**) sum intensity. **(C-D)** Average cysteine enrichment efficiency of peptides identified through ssABE in terms of **(C)** count and **(D)** sum intensity. **(E)** Venn diagram showing the overlap of HA-sensitive sites identified in pro-inflammatory and naïve macrophages. These sites were used to benchmark the SILAC ssABE experiment. **(F)** Overview of the percentage of unique parent proteins, grouped by the number of long-chain *S*-acylation sites detected.

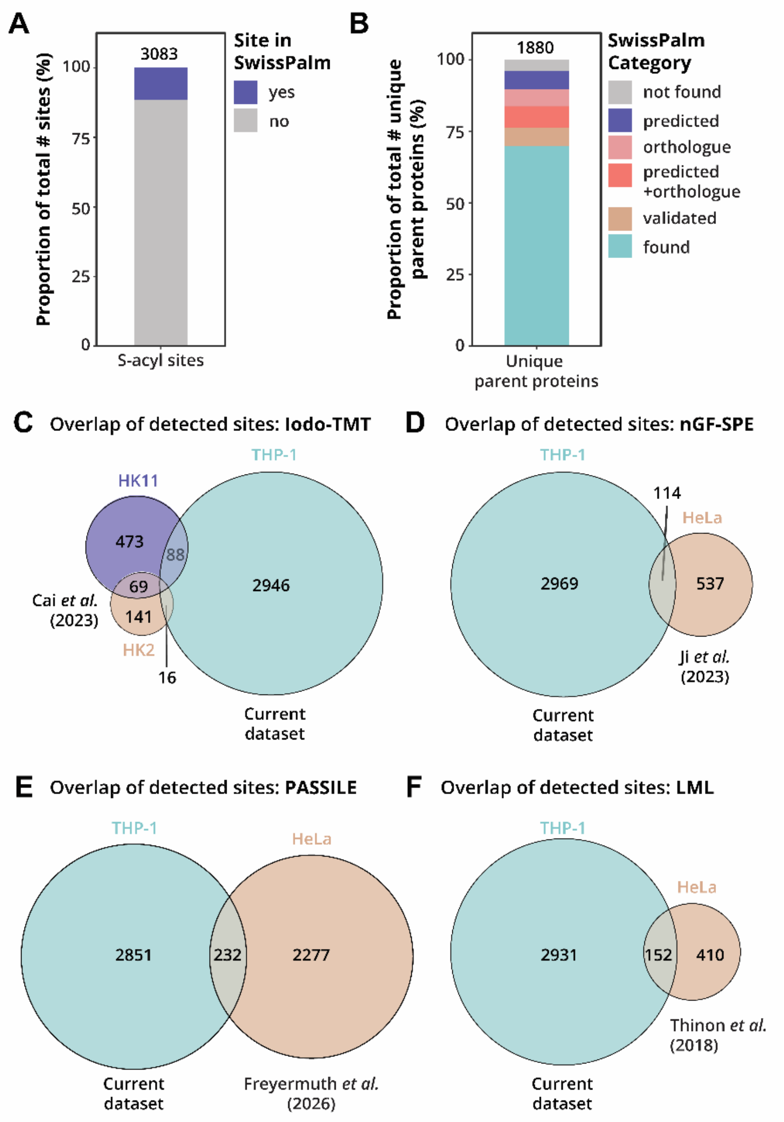

Supporting figure 3. Comparison of long-chain *S*-acylation sites detected in benchmark list with literature studies.

(A) Bar chart showing the percentage of sites that have been validated by e.g. site-directed mutagenesis or mass spectrometry according to SwissPalm. **(B)** Bart showing the number of unique *S*-acylated parent proteins classified by SwissPalm category. **(C-F)** Venn diagrams showing the overlap in long-chain *S*-acylation sites detected in our benchmark list, compared to various site-specific datasets. **(C)** Iodo-TMT in human kidney cell lines [1]. **(D)** direct detection with nanographite fluoride-based solid-phase extraction (nGF-SPE) [2]. **(E)** PASSILE method. Sites were taken from supplementary table si_003, the “averages” sheet. Cysteines detected in pair B were used. Cysteines were filtered for >10% average *S*-acylation percentage, and “reviewed” uniprot ID status [3]. **(F)** Indirect detection with Alk-C16 lipid metabolic labeling (LML) [4].

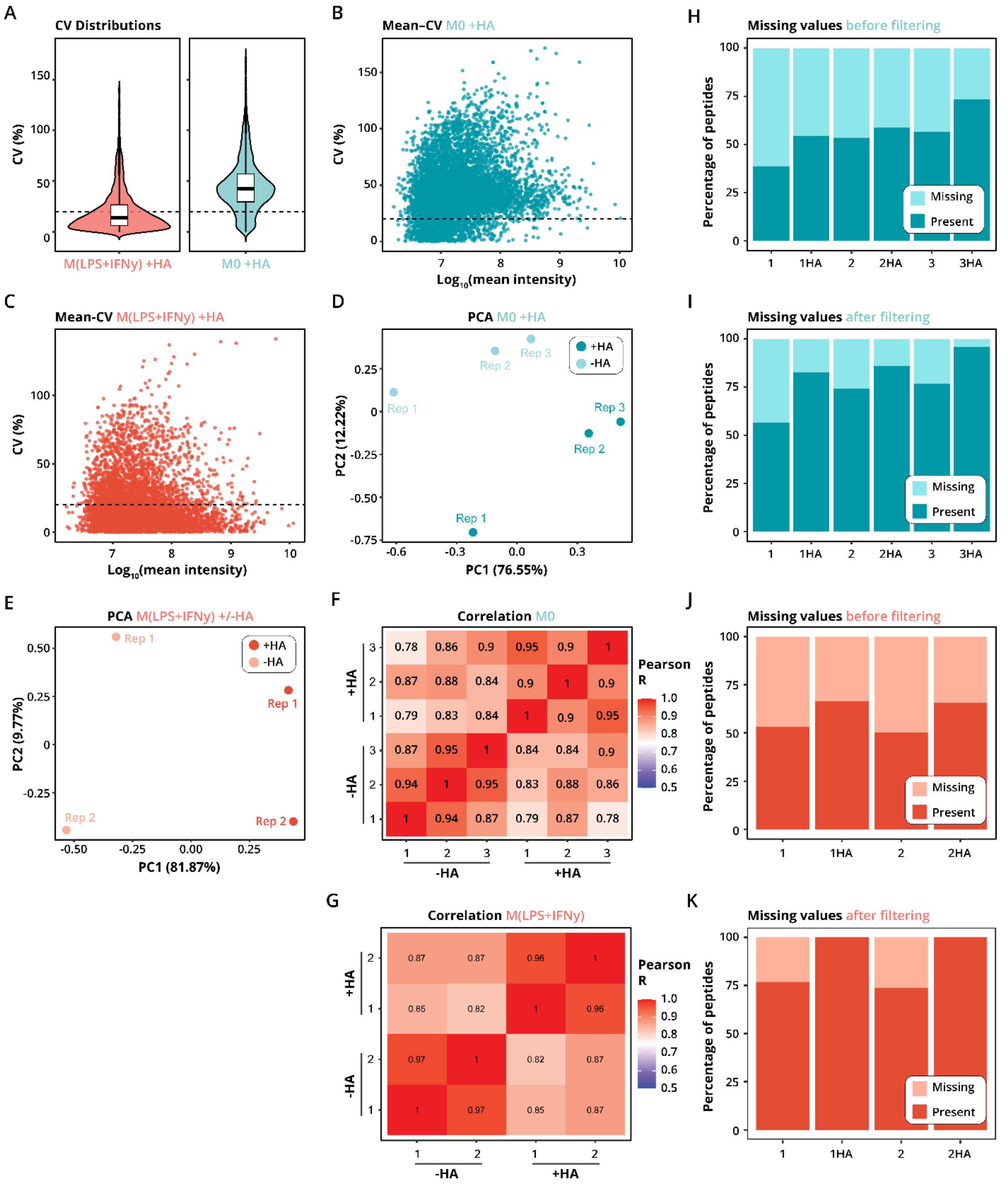

Supporting figure 4. Reproducibility metrics for label-free differentiation experiment.

(A) CV distributions of raw intensities after valid value and localization probability filtering. Horizontal dashed line represents a 20% CV. **(B-C) %** CV (*y*-axis) plotted against $\log_{10}$ mean intensities (*x*-axis) of **(B)** M0 (+HA) replicates and (**C**) M(LPS + IFNɣ) (+HA) replicates. **(D-E)** Principal component analysis (PCA) score plot of the $\log_2$ transformed intensities of site peptides found in **(D)** 2/3 biological replicates of M0 macrophages (+/- HA) and **(E)** 2/2 biological replicates M(LPS + IFNɣ) (+/- HA). **(F-G)** Pearson R correlation plots showing the correlation between individual

replicates after valid value and localization probability filtering of **(F)** M0 macrophages and **(G)** M(LPS + IFNɣ) macrophages. Tiles are colored according to Pearson R coefficient. **(H-K)** Overview of percentage of missing values for detected site peptides per individual replicate **(H)** before filtering in M0 macrophages **(I)** after filtering in M0 macrophages **(J)** before filtering in M(LPS + IFNɣ) macrophages and **(K)** after filtering in M(LPS + IFNɣ) macrophages.

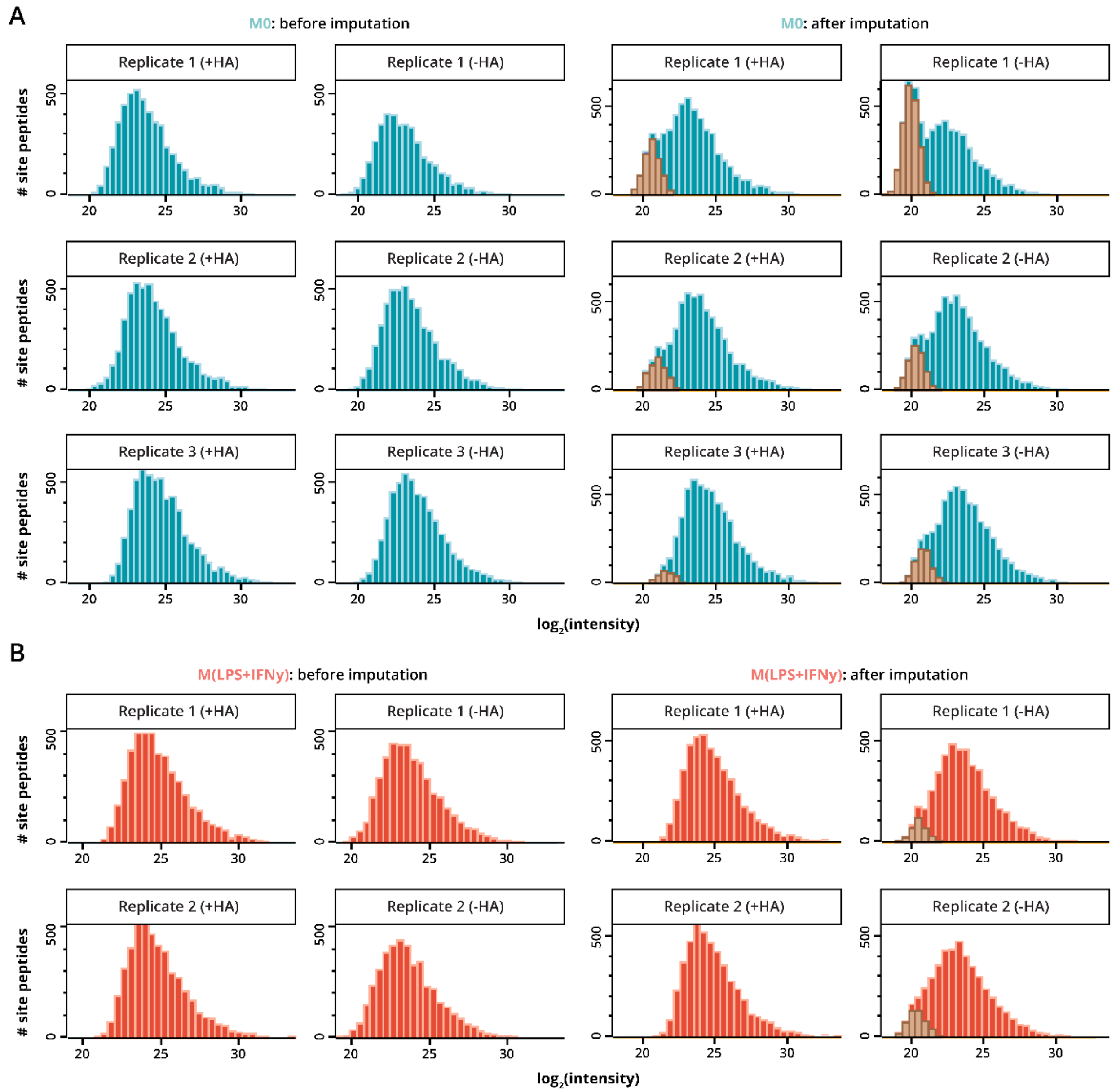

Supporting figure 5. Summary of imputation for label-free differentiation experiment.

(A-B) Histograms showing the distribution of detected site peptides versus their $\log_2$ intensities per individual replicates before and after imputation for **(A)** M0 macrophage replicates and **(B)** M(LPS + IFNɣ) macrophage replicates. Imputed values are represented by teal-colored bars. Histograms were produced with Perseus (v.1.6.14.0). Note, M(LPS + IFNɣ) +HA replicates were filtered for 2/2 valid values and thus have no imputed values.

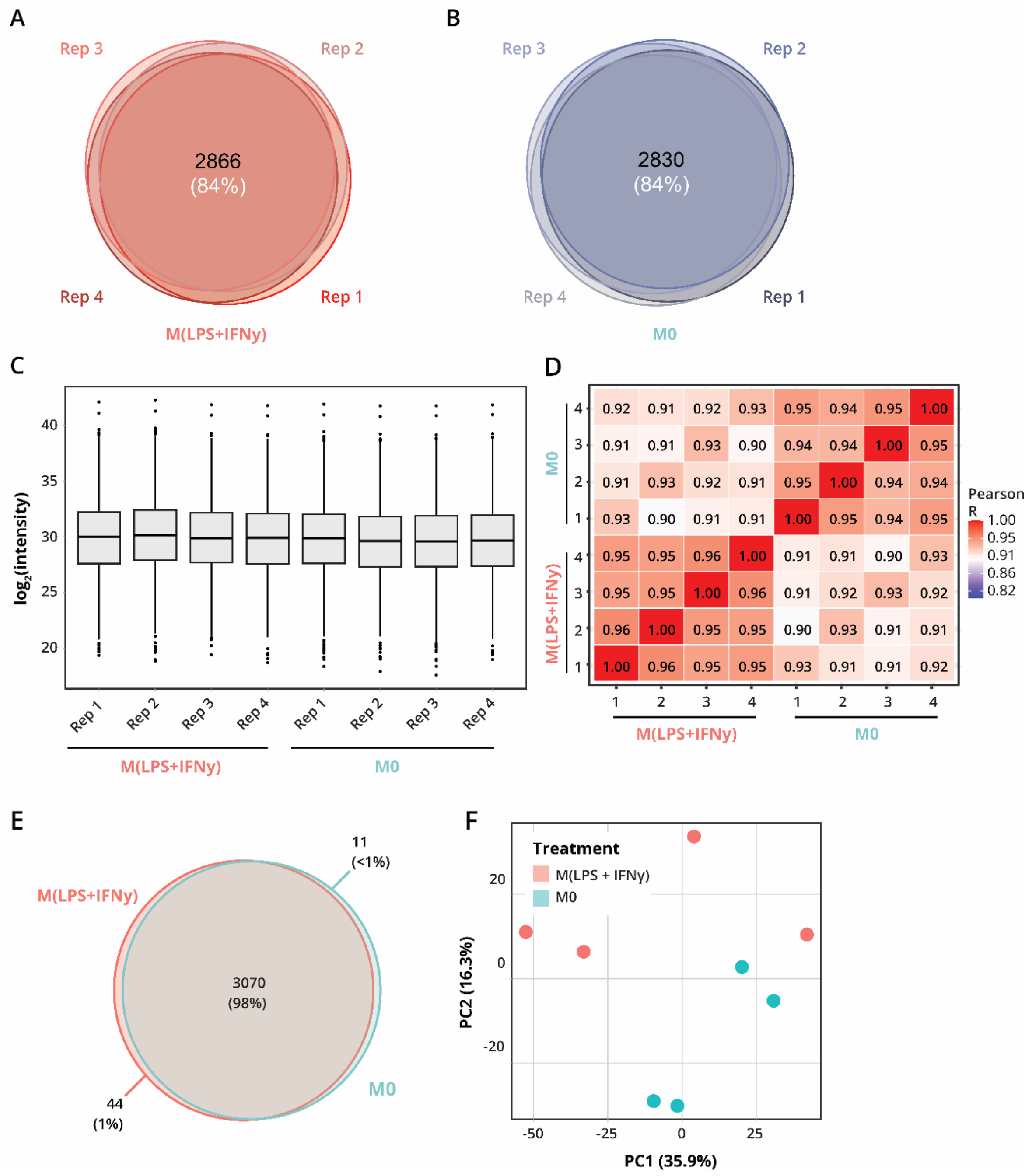

Supporting figure 6. SILAC total proteome replicate overview.

(A-B) Venn diagrams showing the overlap of protein identifications in **(A)** M(LPS + IFNɣ) replicates and **(B)** M0 replicates. **(C)** Box plot showing the distribution of log2 transformed raw intensities of proteins detected in each replicate. (D) Pearson R correlation plot showing the correlation between individual replicates after filtering for valid values in 3/4 replicates in both conditions **(E)** Venn-diagram shows the overlap of identified proteins in M0 and M(LPS+IFNɣ) treated macrophages. A protein is considered identified if it is detected in 3/4 biological replicates **(F)** Principal component analysis (PCA) score plot of the log_2 transformed intensities of proteins found in 3/4 biological replicates of M(LPS+IFNɣ) or M0 macrophages.

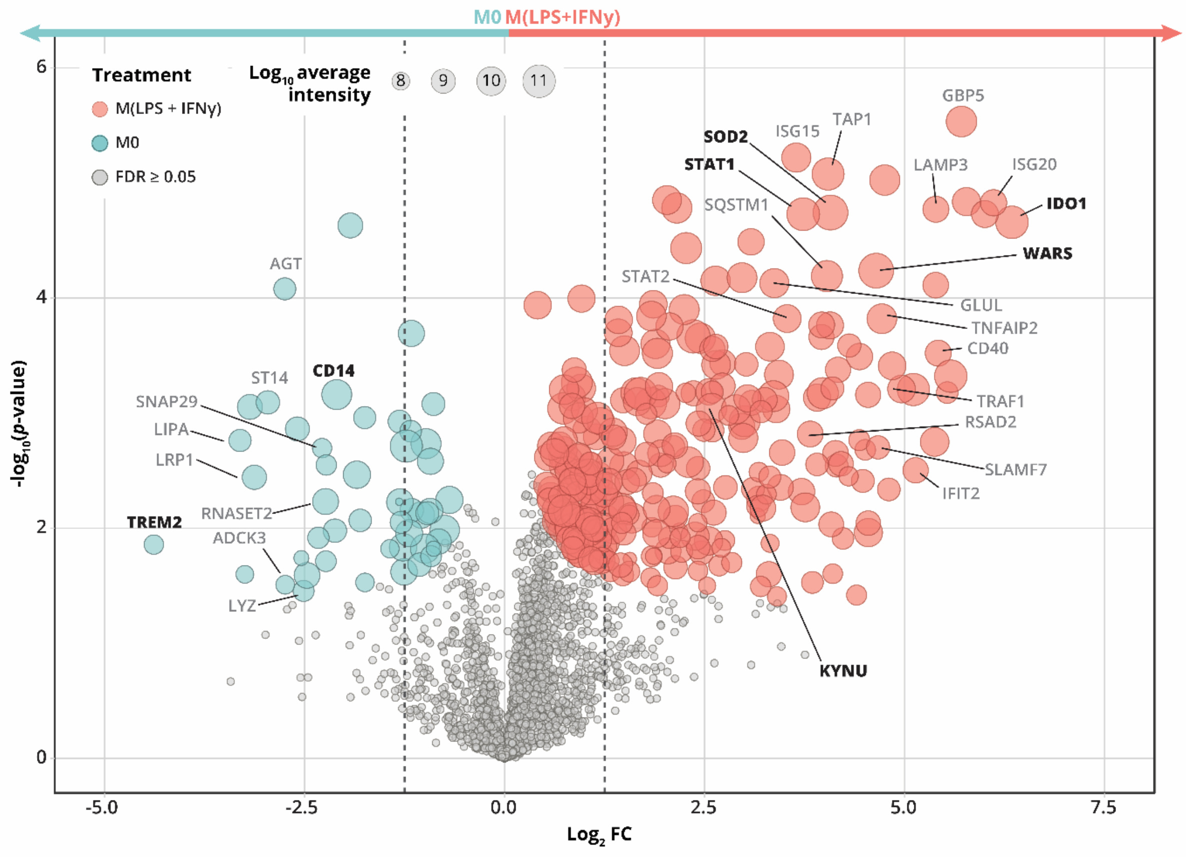

Supporting figure 7. SILAC total proteome 3D volcano plot.

Plot shows the differential abundance of identified proteins in both M(LPS+IFNγ) and M0 treated macrophages. For each protein, $\log_{10}$ intensity is plotted and colored according to the condition in which it is enriched (M1 = coral, M0 = cyan). Bubble size is determined by the average $\log_{10}$ intensity. Proteins not significantly up- or downregulated are depicted in grey and have the same bubble size regardless of their $\log_{10}$ intensity. Significance was determined by a two-sample paired *t*-test (FDR 0.05, S0 0.1 and *n*=4 biological replicates from two independent experiments). Vertical dashed lines are positioned at $|\log_2$ fold change$| = 1.25$. Some of the significantly up- or downregulated proteins with $-2 \geq \log_2$ fold change ≥ 2 are labelled.

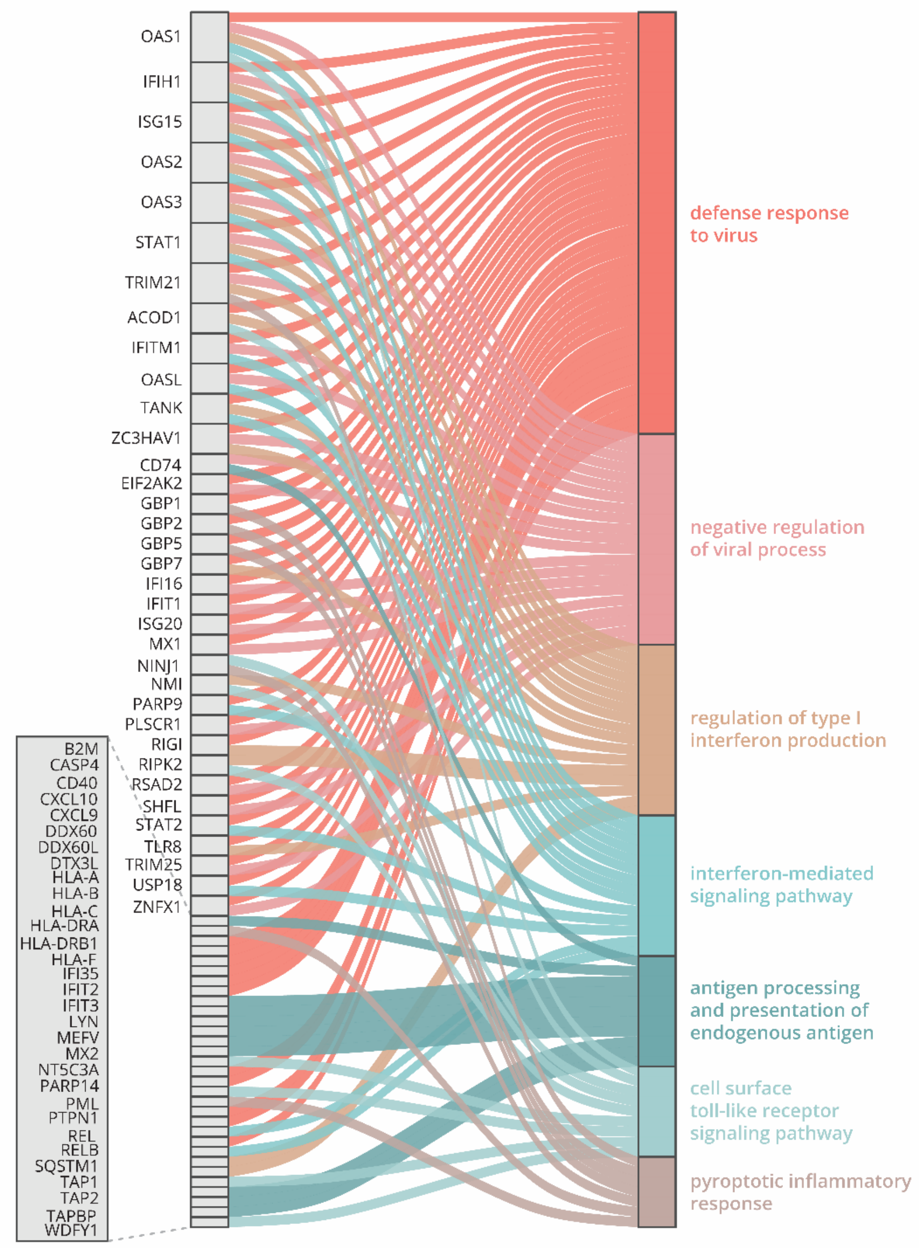

Supporting figure 8. Sankey plot of proteins enriched in M(LPS+IFNɣ) polarized macrophages that belong to the top 7 enriched biological process GO terms.

Overrepresentation analysis was performed using ClusterProfiler R package. GO term redundancy was reduced using the simplify() function with a cutoff of 0.4 based on semantic similarity. Protein tiles are sized based on number of GO terms. GO term tiles are sized based on the adjusted *p*-values and count of proteins.

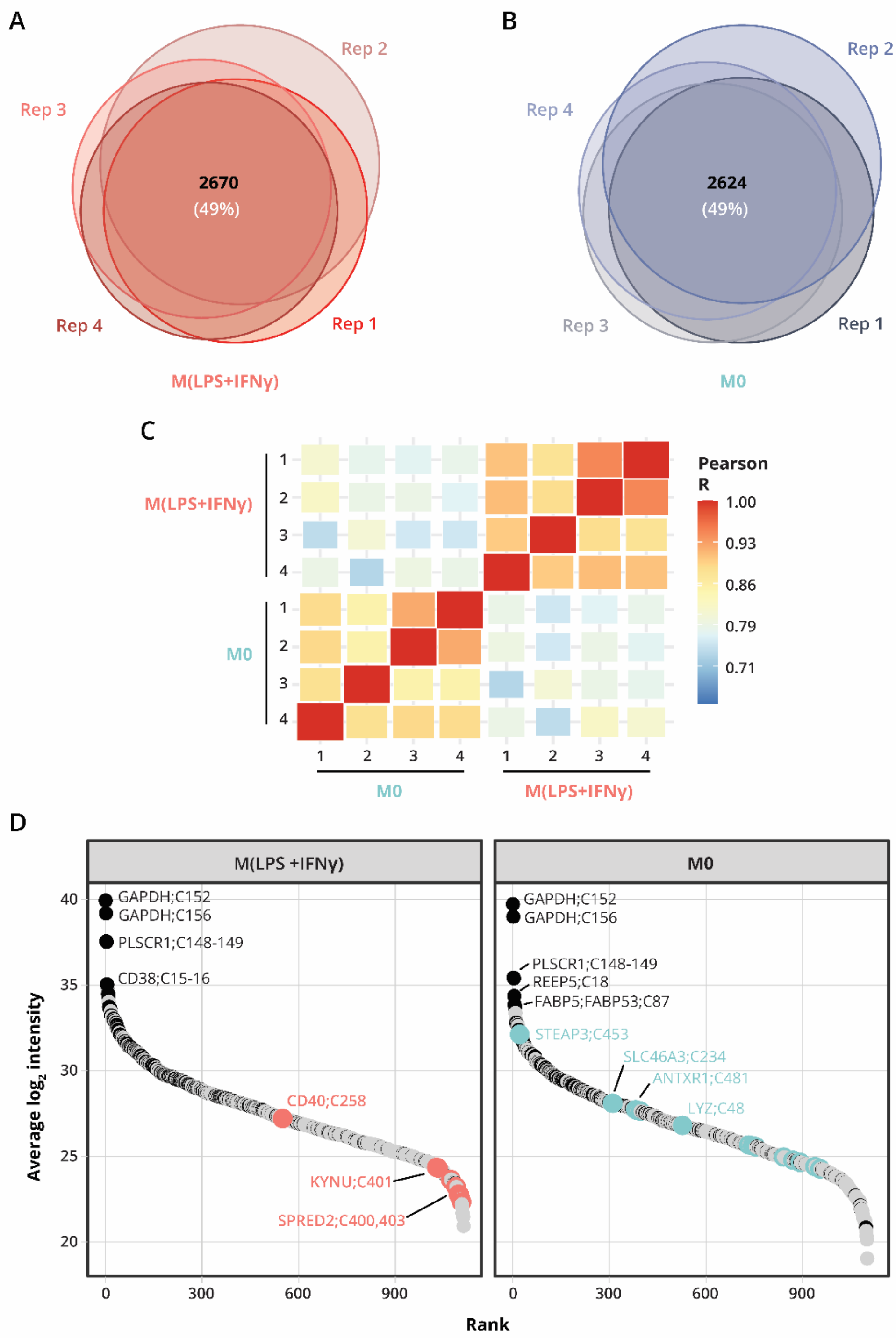

Supporting figure 9. SILAC ssABE replicate and site overview.

(A-B) Venn diagrams showing the overlap of site identifications in **(A)** M(LPS+IFNγ) replicates and **(B)** M0 replicates. **(C)** Pearson R correlation plot showing the correlation between individual replicates. Tiles are colored and sized according to Pearson R coefficient. **(D)** Rank-abundance curves of 1111 HA-sensitive sites. Left: M(LPS+IFNγ) macrophages. Right: M0 macrophages. *Y*-axes represent the average $\log_2$ site intensity; *x*-axes show rank. Black-colored sites are experimentally validated according to SwissPalm. Black-colored sites with a written label are the top

6 most abundant in each condition. Cyan represents sites with an average $\log_2(M1/M0) \leq -1.74$, while coral indicates sites unique to M(LPS+IFNɣ) macrophages (absent in M0).

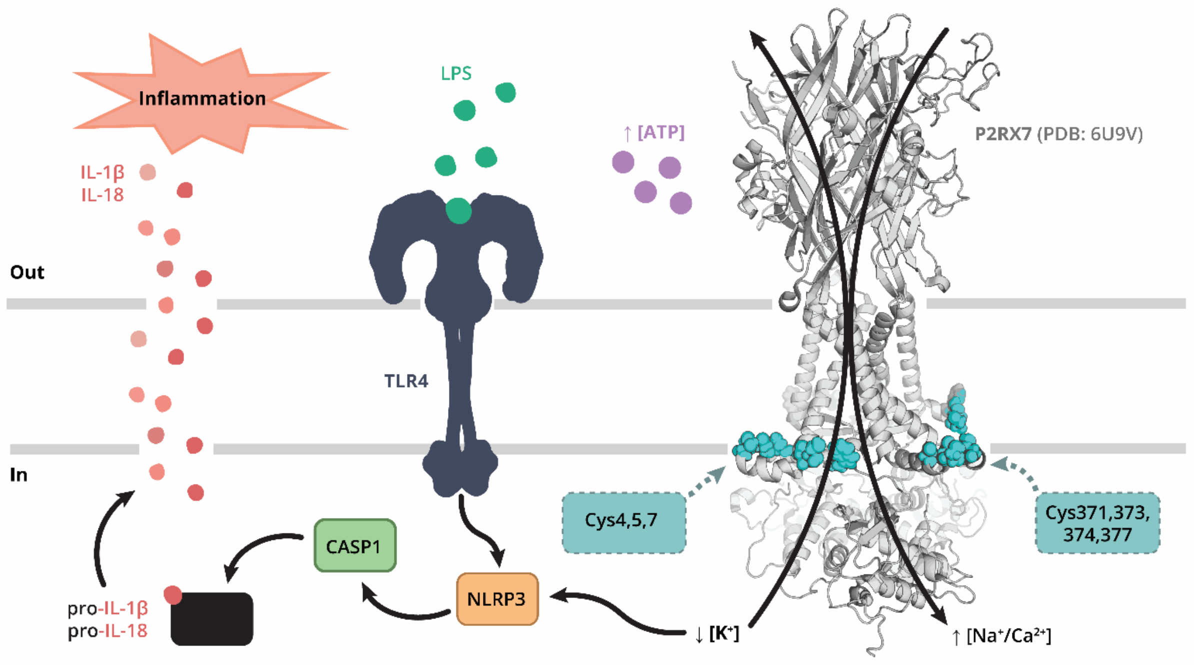

Supporting figure 10. Simplified graphic: long-chain *S*-acylation mediated attenuation of purinoceptor 7 ATP desensitization.

Desensitization results in maintained AIM2/NLRP3 inflammasome stimulation and caspase-1 mediated cleavage of pro-IL-18 and pro-IL-1-beta for release of IL-18 and IL-1-beta. Long-chain *S*-acylation sites on P2RX7 are colored cyan.

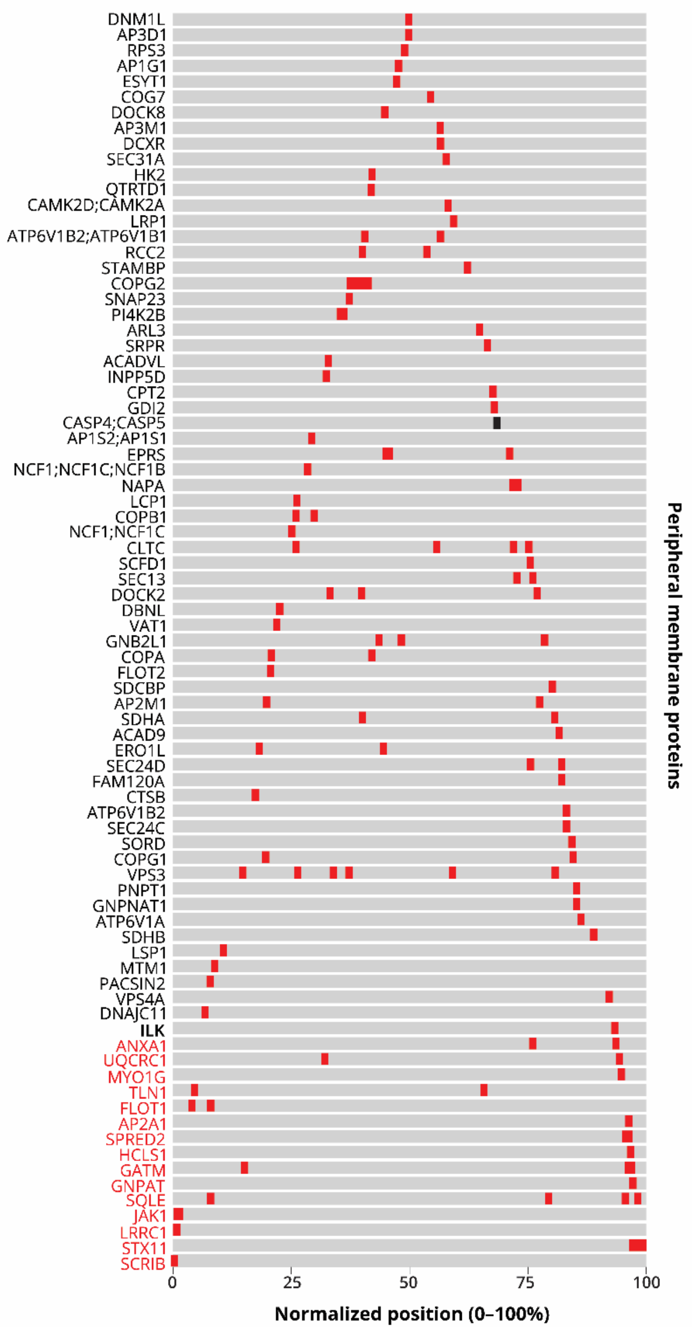

Supporting figure 11. 81 long-chain *S*-acylated peripheral membrane proteins.

Protein lengths are normalized to 100% and identified sites were mapped relative to protein length. Proteins are ordered top to bottom in decreasing relative site distance from either protein N- or C-terminus. Black vertical lines indicate position which also function as a catalytic site.

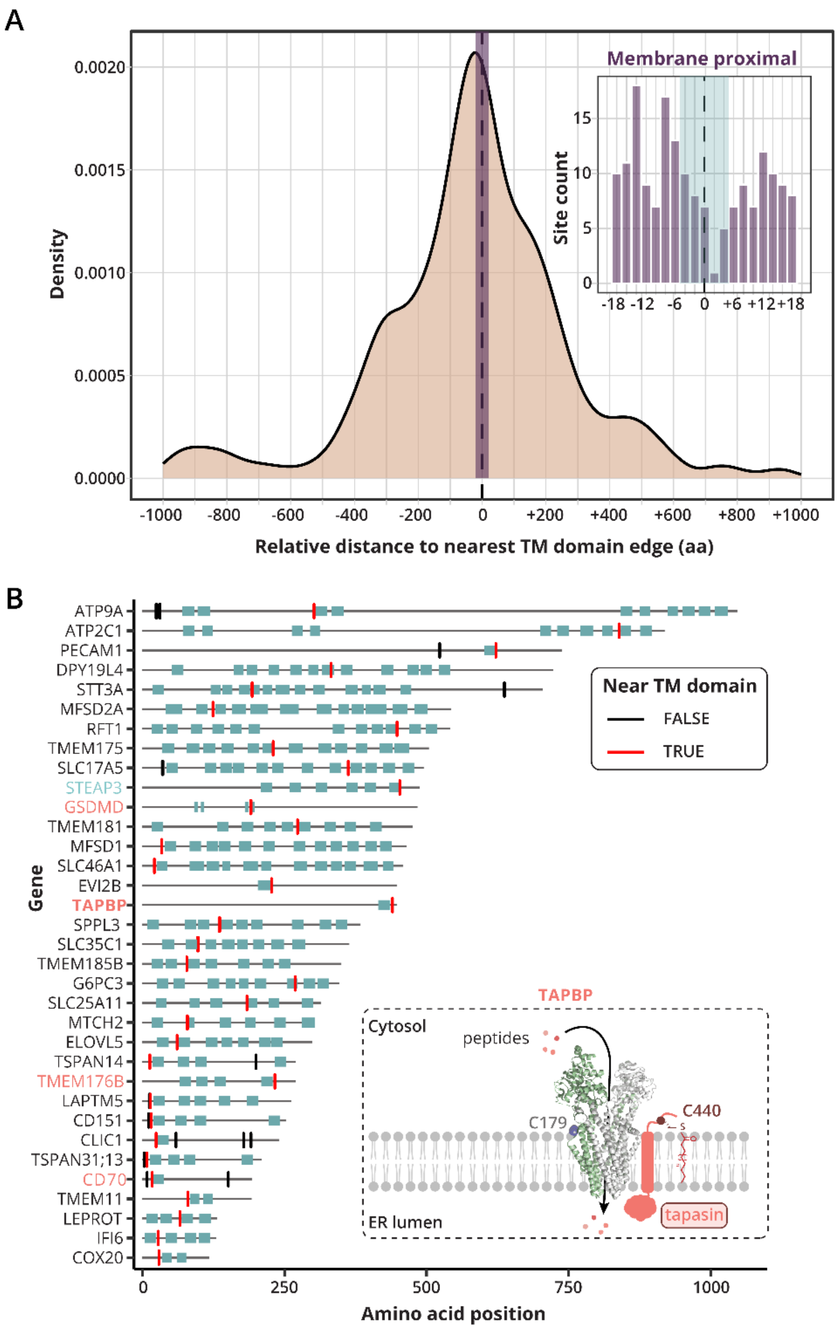

Supporting figure 12. Long-chain S-acylation sites relative to transmembrane domains.

(A) Distribution of the detected long-chain *S*-acylation sites in SILAC ssABE dataset, relative to the nearest TMD domain in the protein. Negative values indicate a relative position N-terminal of a TMD, positive values indicate a relative position C-terminal of a TMD. Purple bar indicates the region within 20 residues of the TMD edge, which was considered "membrane proximal" in Forrester *et al.* (2024) [5]. Inset is a zoom-in of the purple bar. **(B)** Site mapping on a subset of transmembrane proteins which have at least one detected site within 5 residues of a TMD edge. Sites are mapped along the absolute protein length. Blue bars indicate TMDs. Red vertical lines indicate long-chain *S*-acylation sites within 5 residues of the nearest TMD edge. Black vertical lines indicate long-chain *S*-acylation site outside 5 residues of the nearest TMD edge. Cyan-colored genes contain site significantly upregulated in M0 macrophages.

Coral-colored genes contain sites significantly upregulated in M(LPS+IFNɣ) macrophages. Graphic shows TAPBP (C440) in the context of its interaction with peptide transporter TAP1.

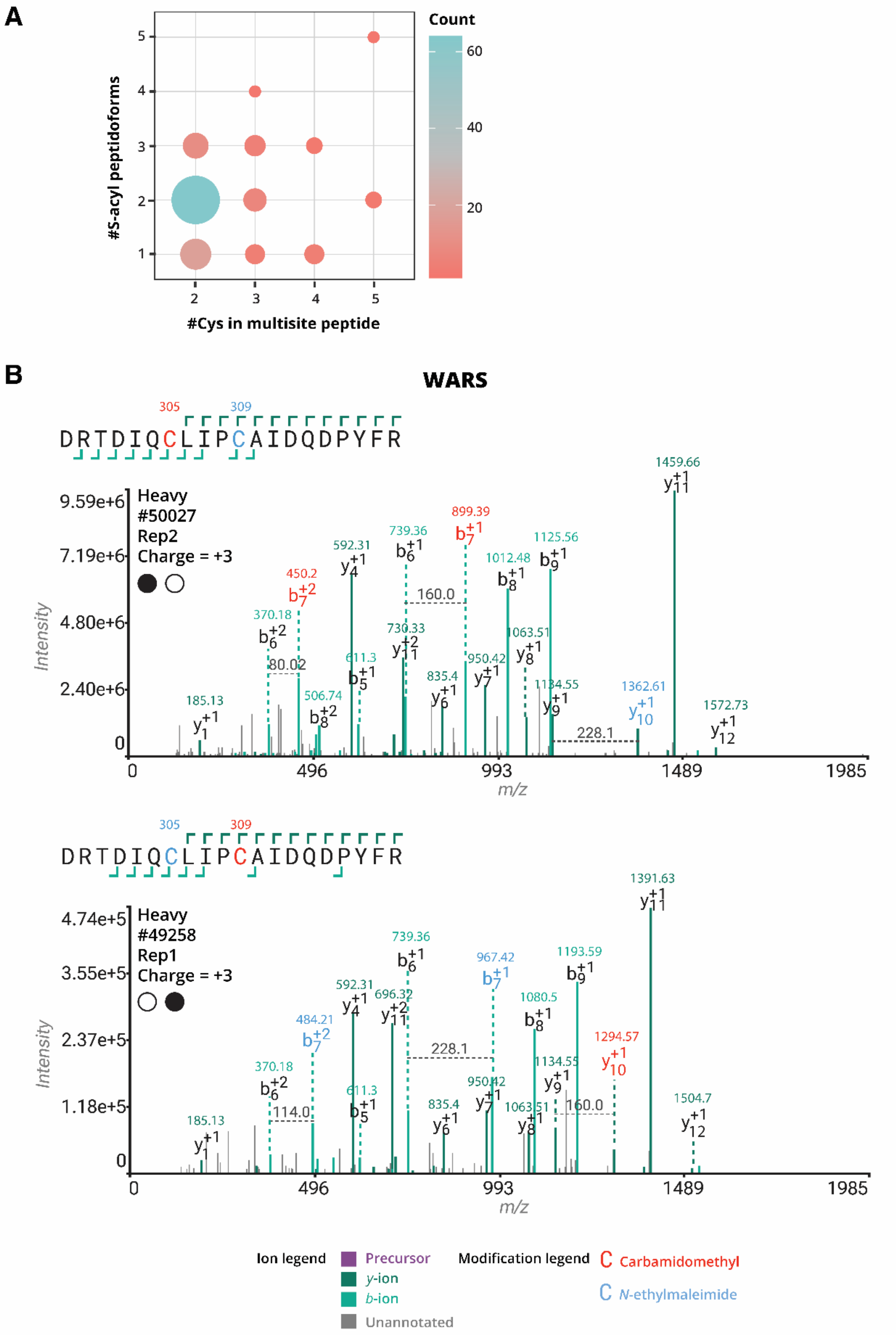
A
Count
#S-acyl peptidoforms
#Cys in multisite peptide
B
WARS
DRTDIQCLIPCAIDQDPYFR
Heavy
#50027
Rep2
Charge = +3
Intensity
m/z
Heavy
#49258
Rep1
Charge = +3
Ion legend
Precursor
y-ion
b-ion
Unannotated
Modification legend
Carbamidomethyl
N-ethylmaleimide

C **JAK1**

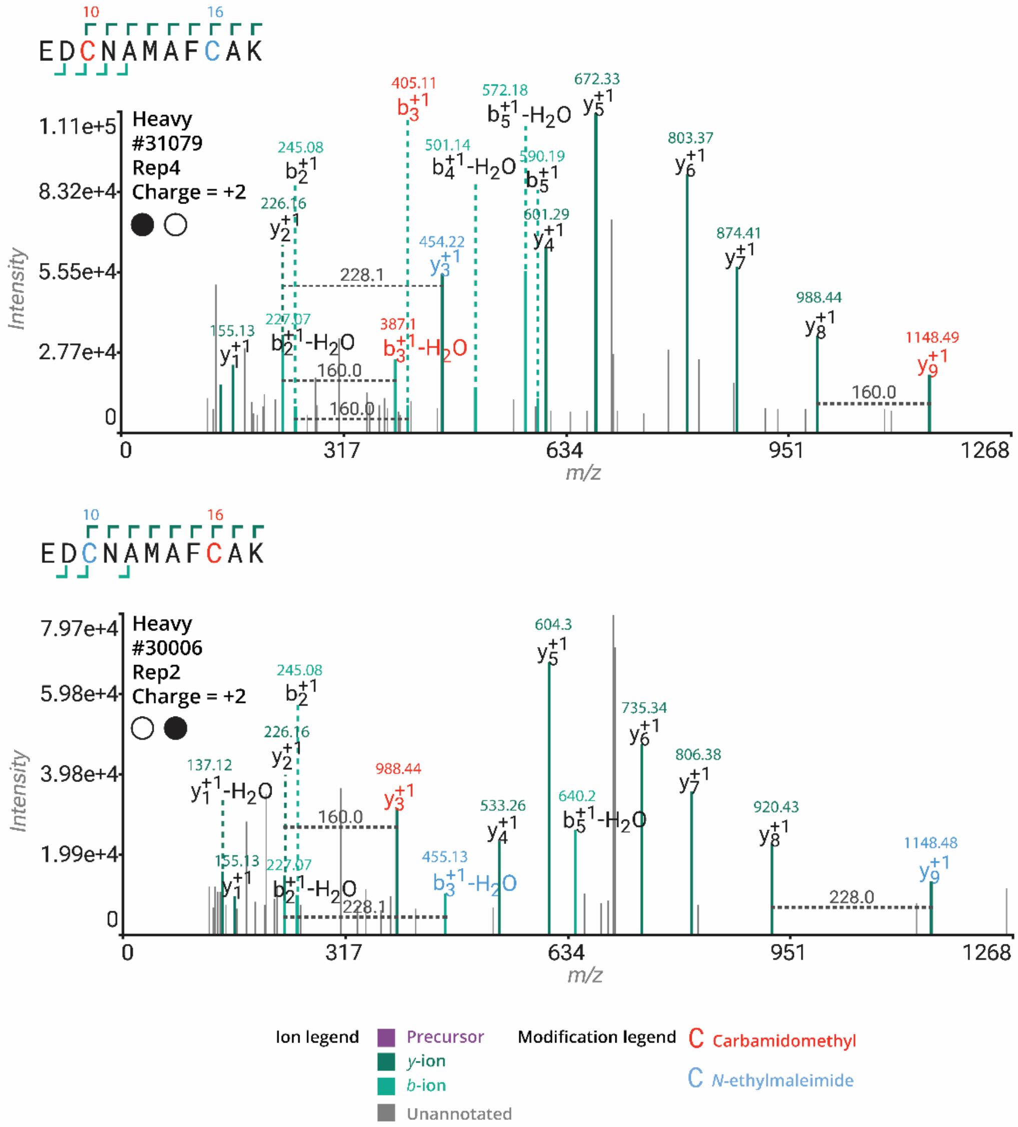

10
16
EDCNAMAFCAK
1.11e+5
8.32e+4
5.55e+4
2.77e+4
0
Heavy
#31079
Rep4
Charge = +2
Intensity
405.11
572.18
672.33
245.08
501.14
590.19
803.37
226.16
601.29
454.22
874.41
228.1
988.44
155.13
227.07
387.1
1148.49
160.0
160.0
160.0
0
317
634
951
1268
m/z
10
16
EDCNAMAFCAK
7.97e+4
5.98e+4
3.98e+4
1.99e+4
0
Heavy
#30006
Rep2
Charge = +2
Intensity
604.3
245.08
735.34
226.16
806.38
137.12
988.44
160.0
640.2
533.26
920.43
455.13
1148.48
155.13
227.07
228.1
228.0
0
317
634
951
1268
m/z
Ion legend
Precursor
y-ion
b-ion
Unannotated
Modification legend
C Carbamidomethyl
C N-ethylmaleimide

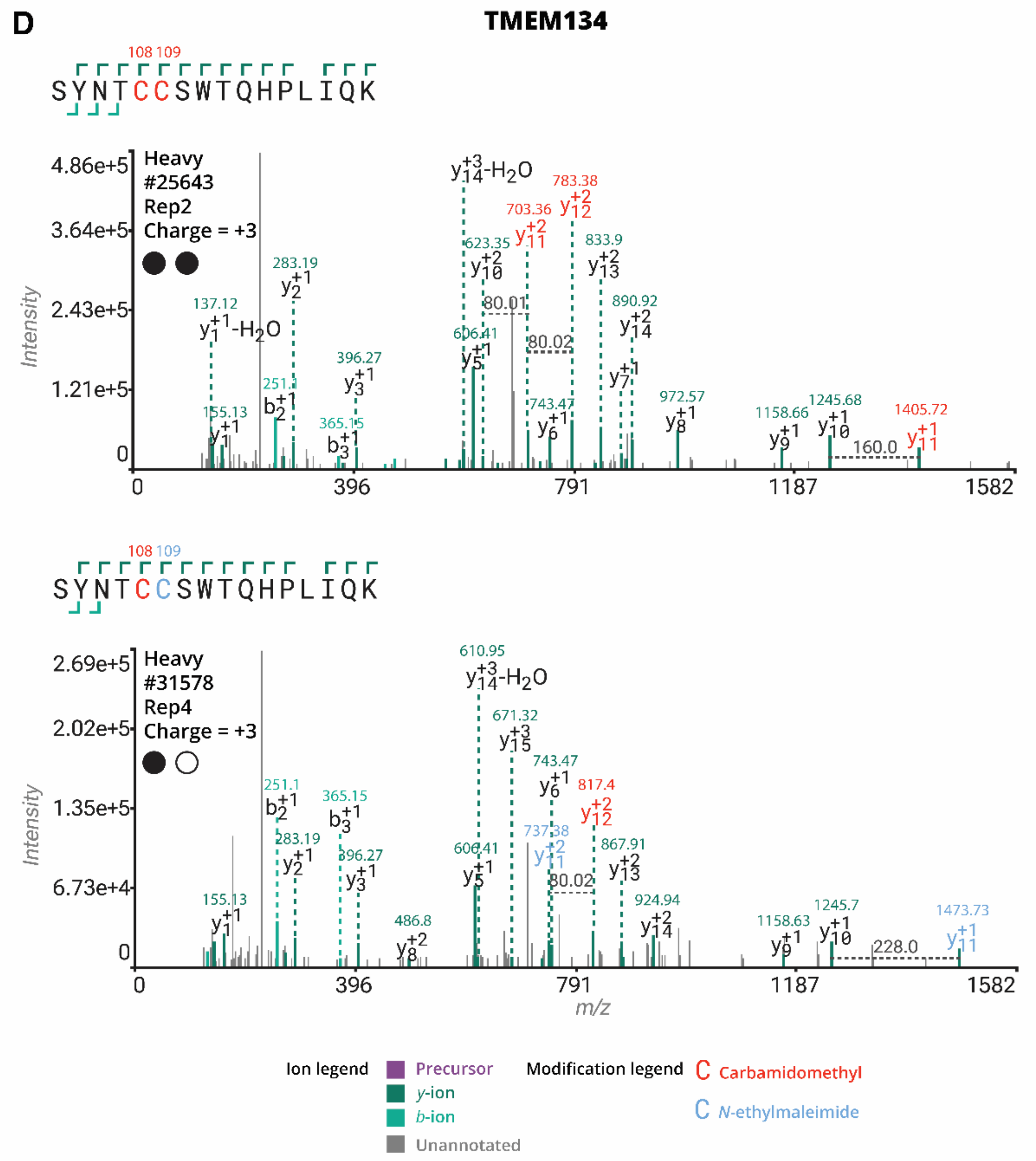
D
TMEM134
108 109
SYNTCCSWTQHPLIQK
Heavy
#25643
Rep2
Charge = +3
Intensity
4.86e+5
3.64e+5
2.43e+5
1.21e+5
0
396
791
1187
1582
Heavy
#31578
Rep4
Charge = +3
2.69e+5
2.02e+5
1.35e+5
6.73e+4
m/z
Ion legend
Precursor
y-ion
b-ion
Unannotated
Modification legend
C Carbamidomethyl
C N-ethylmaleimide

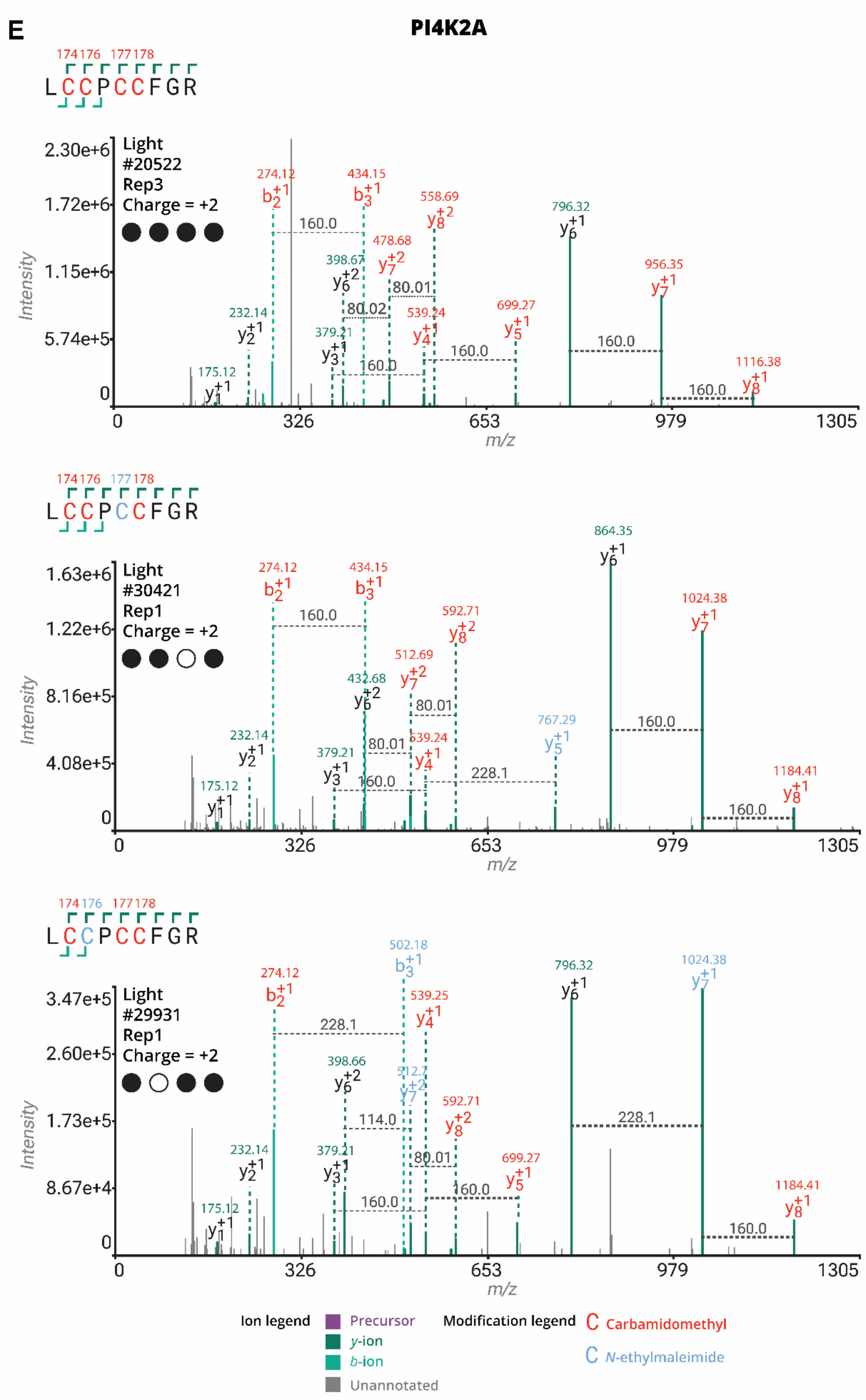
E
PI4K2A
Light
#20522
Rep3
Charge = +2
Light
#30421
Rep1
Charge = +2
Light
#29931
Rep1
Charge = +2
Intensity
m/z
Ion legend
Precursor
y-ion
b-ion
Unannotated
Modification legend
Carbamidomethyl
N-ethylmaleimide

Supporting figure 13. *S*-acyl-peptidoforms.

(A) Relationship between the number of cysteines in a peptide (x-axis) and the number of detected S-acyl peptidoforms for that peptide sequence (y-axis). Bubbles are sized and colored by number of *S*-acyl peptidoforms. **(B)** WARS, 2x heavy spectra. **(C)** JAK1, 2x heavy spectra. **(D)** TMEM134, 2x heavy spectra. **(E)** PI4K2A, 3x light spectra**.** For all spectra shown: The filled/empty circle codes found in the top left of the spectra correspond to the *S*-acyl-peptidoform legend as shown in figure 4 of the main text. Peaks are labelled with their ion type, charge and *m/z*. Cysteine residues in the peptide annotations are colored red if they are carbamidomethylated (= long-chain *S*-acylation site). In these cases, the label of their corresponding ion peak is also colored red. Cysteine residues in the peptide annotations are colored lightblue if they are modified with *N*-ethylmaleimide (NEM). In this case the label of their corresponding ion peak is also colored lightblue. Horizontal dotted lines denote the mass shifts between peaks surrounding the carbamidomethylated or NEM modified cysteines. The top left of each spectrum contains the following information, from top to bottom: Isotopic label of peptide (heavy or light; if heavy, this means that all R and K in the peptide sequence are heavy labelled), MS/MS scan number, raw file (refer to PRIDE submission), precursor charge.

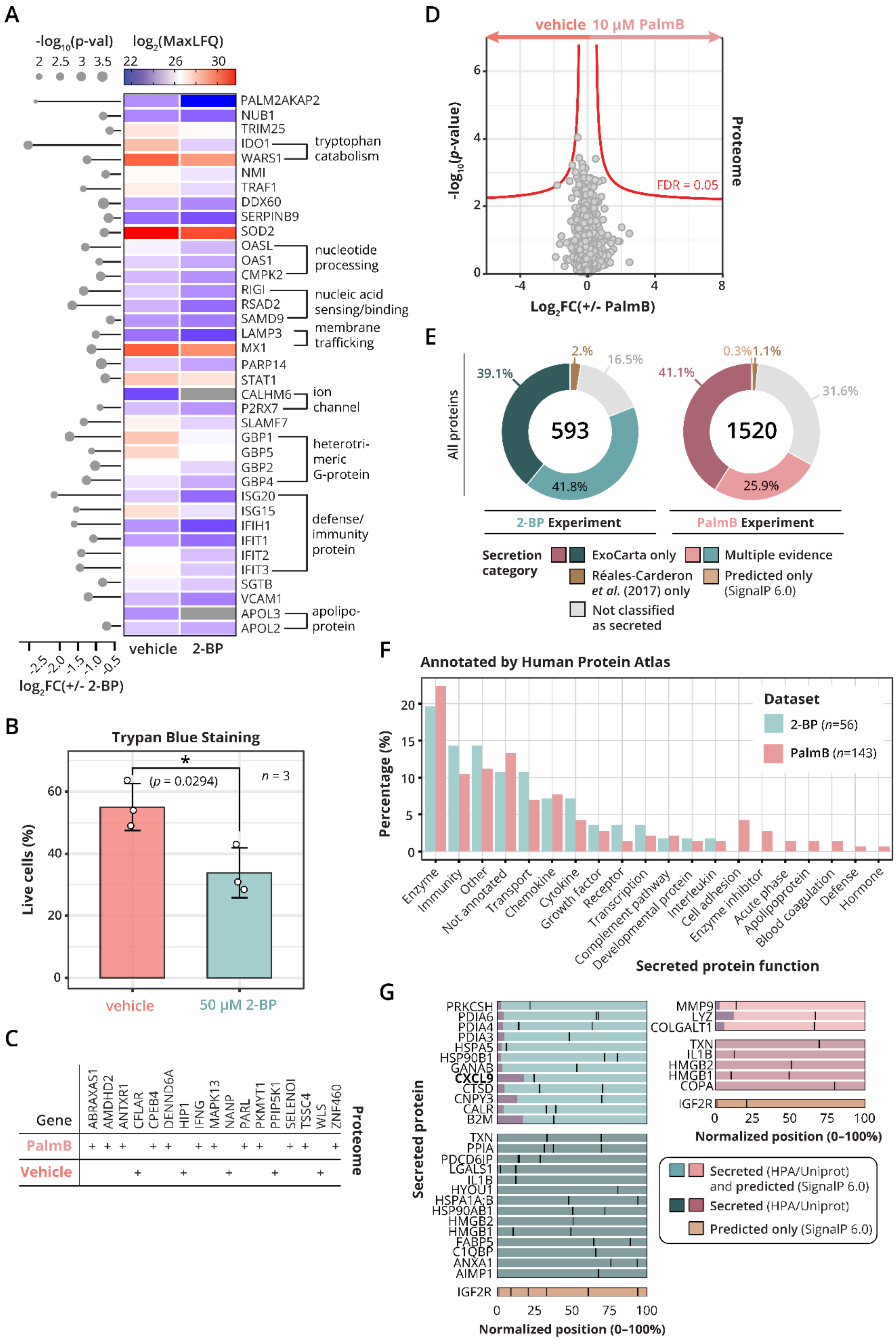
A
-log10(p-val)
log2(MaxLFQ)
PALM2AKAP2
NUB1
TRIM25
IDO1
WARS1
tryptophan catabolism
NMI
TRAF1
DDX60
SERPINB9
SOD2
OASL
OAS1
CMPK2
nucleotide processing
RIGI
RSAD2
SAMD9
nucleic acid sensing/binding
LAMP3
MX1
membrane trafficking
PARP14
STAT1
CALHM6
P2RX7
ion channel
SLAMF7
GBP1
GBP5
GBP2
GBP4
heterotri-meric G-protein
ISG20
ISG15
IFIH1
IFIT1
IFIT2
IFIT3
defense/ immunity protein
SGTB
VCAM1
APOL3
APOL2
apolipo-protein
vehicle
2-BP
log2FC(+/- 2-BP)
B
Trypan Blue Staining
(p = 0.0294)
n = 3
Live cells (%)
vehicle
50 μM 2-BP
C
Gene
ABRAXAS1
AMDHD2
ANTXR1
CFLAR
CPEB4
DENND6A
HIP1
IFNG
MAPK13
NANP
PARL
PKMYT1
PPIP5K1
SELENOI
TSSC4
WLS
ZNF460
PalmB
Vehicle
Proteome
D
vehicle
10 μM PalmB
-log10(p-value)
FDR = 0.05
Proteome
Log2FC(+/- PalmB)
E
All proteins
39.1%
2.%
16.5%
593
41.8%
41.1%
0.3%
1.1%
31.6%
1520
25.9%
2-BP Experiment
PalmB Experiment
Secretion category
ExoCarta only
Multiple evidence
Réales-Carderon et al. (2017) only
Predicted only (SignalP 6.0)
Not classified as secreted
F
Annotated by Human Protein Atlas
Percentage (%)
Dataset
2-BP (n=56)
PalmB (n=143)
Enzyme
Immunity
Other
Not annotated
Transport
Chemokine
Cytokine
Growth factor
Receptor
Transcription
Complement pathway
Developmental protein
Interleukin
Cell adhesion
Enzyme inhibitor
Acute phase
Apolipoprotein
Blood coagulation
Defense
Hormone
Secreted protein function
G
Secreted protein
PRKCSH
PDIA6
PDIA4
PDIA3
HSPA5
HSP90B1
GANAB
CXCL9
CTSD
CNPY3
CALR
B2M
TXN
PPIA
PDCD6IP
LGALS1
IL1B
HYOU1
HSPA1A;B
HSP90AB1
HMGB2
HMGB1
FABP5
C1QBP
ANXA1
AIMP1
IGF2R
MMP9
LYZ
COLGALT1
TXN
IL1B
HMGB2
HMGB1
COPA
IGF2R
Normalized position (0–100%)
Secreted (HPA/Uniprot) and predicted (SignalP 6.0)
Secreted (HPA/Uniprot)
Predicted only (SignalP 6.0)
Normalized position (0–100%)

Supporting figure 14. Comparative secretome, proteome, and *S*-acyl-proteome analysis of PalmB/2-BP inhibited inflammatory macrophages.

(A) Heatmap of proteins significantly enriched in the M(LPS+IFNɣ) condition of the SILAC total proteome experiment, that are found to be significantly downregulated (unpaired *t*-test, FDR 0.05, S0 0.1, $\log_2$ fold change ≤ -0.5) after 2-BP treatment in the current experiment. Proteins are shown in descending order from largest to smallest mean $\log_2$(MaxLFQ). **(B)** Trypan-blue staining to determine the % viability of pro-inflammatory macrophages treated with either vehicle or 50 µM 2-BP for 1hr 15min. Measurements were done on a separate 6-well experiment performed with identical conditions to the experiment featured in the main text. Data represent mean ± standard deviation of three biological replicates, each measured in technical duplicates. Individual datapoints represent means of each pair of technical duplicates. A two-sample t-test was performed to determine significant differences between conditions. ****p* < 0.001; ***p* < 0.01; **p* < 0.05; "ns" *p* > 0.05. **(C)** Table showing proteins uniquely detected in either PalmB treated macrophages or vehicle-treated macrophages. **(D)** Volcano plot showing the differential abundance of identified proteins in both PalmB and vehicle treated inflammatory macrophages. Significance was determined by a two-sample unpaired *t*-test (FDR 0.05, S0 0.1 and *n*=3 biological replicates). **(E)** Pie-charts showing the total number of proteins detected in either 2-BP or PalmB secretome experiments. Diagrams illustrate the distribution of proteins identified in the ExoCarta database [6], reported by Reales-Calderon *et al.* (2017) [7], annotated as secreted in the Human Protein Atlas (HPA) or UniProt, or predicted to be secreted by SignalP 6.0. Proteins classified under the "Multiple Evidence" category were supported by more than one of these resources. **(F)** Bar charts of HPA classified secreted proteins per dataset and their reported functions. **(G)** Overview of secreted proteins (as annotated by Uniprot or HPA) with their detected long-chain *S*-acylation sites. For the PalmB dataset the accompanying *S*-acyl-proteome data was used. For the 2-BP dataset, long-chain *S*-acylation sites from the benchmark list were used. Signal peptides are indicated in purple. Long-chain *S*-acylation sites are black lines.

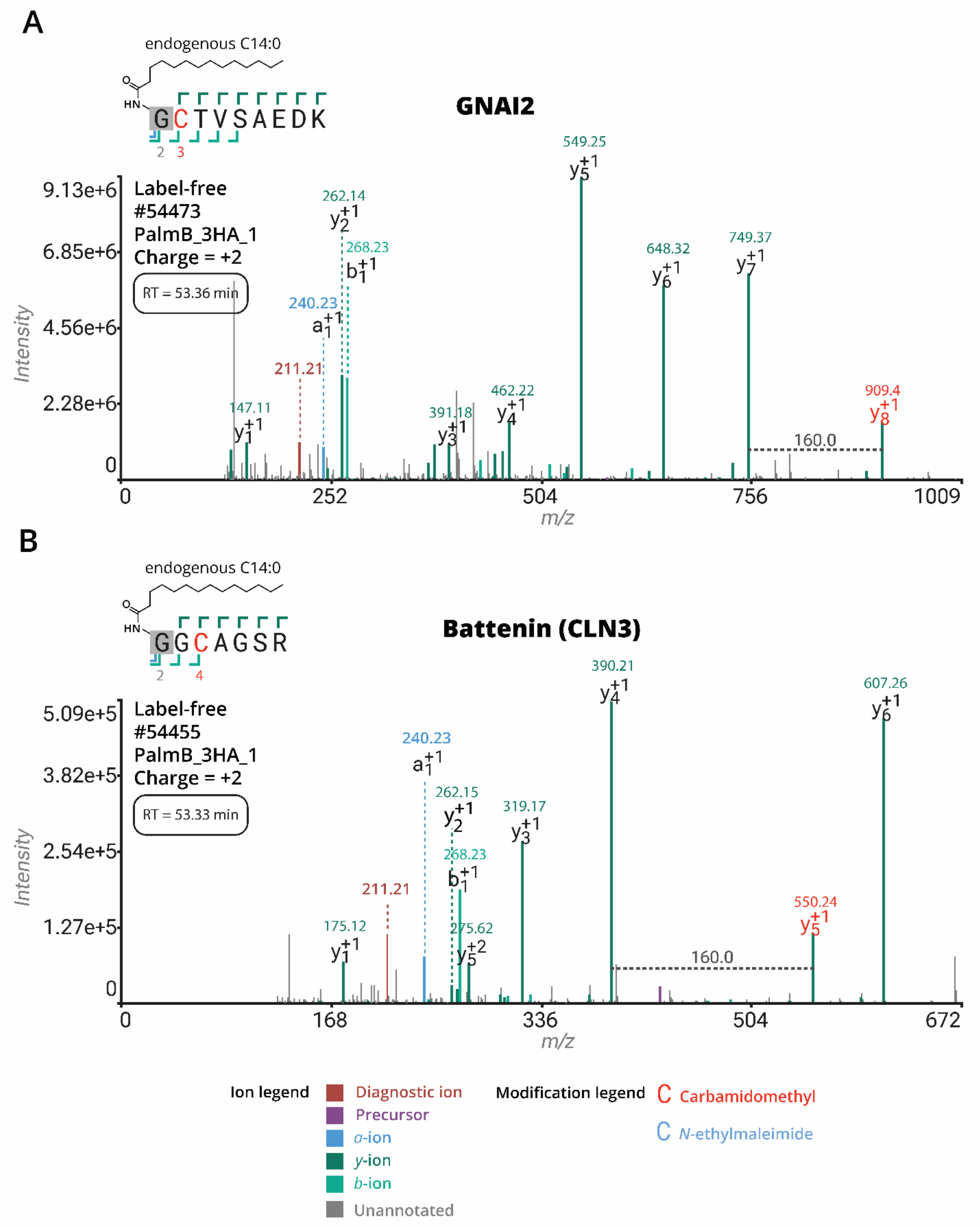
A
endogenous C14:0
GCTVSAEDK
GNAI2
Label-free
#54473
PalmB_3HA_1
Charge = +2
RT = 53.36 min
Intensity
m/z
549.25
262.14
268.23
240.23
211.21
648.32
749.37
147.11
391.18
462.22
909.4
160.0
B
endogenous C14:0
GGCAGSR
Battenin (CLN3)
Label-free
#54455
PalmB_3HA_1
Charge = +2
RT = 53.33 min
Intensity
m/z
390.21
607.26
240.23
262.15
319.17
268.23
211.21
550.24
175.12
275.62
160.0
Ion legend
Diagnostic ion
Precursor
a-ion
y-ion
b-ion
Unannotated
Modification legend
C Carbamidomethyl
C N-ethylmaleimide

Supporting figure 15. Mass spectra of detected peptides with endogenous protein N-terminal glycine myristoylation.

Spectra were annotated with Annotator software. Presence of myristoylation was confirmed by a diagnostic ion at m/z 211.21. Raw files were searched with both MaxQuant (v.2.5.5.0, glycine myristoylation +210.19836) and Fragpipe (v.22.0, protein N-term or any residue, +210.19836). Myristoylated and carbamidomethylated spectra shown here were confidently detected by both MaxQuant and Fragpipe. Mass spectra were visualized from the MaxQuant output. **(A)** Mass spectrum of GNAI2 **(B)** Mass spectrum of Battenin (CLN3).

References

1. Cai J, Song M, Li M, Merchant M, Benz F, McClain C, Klein J: **Site-Specific Identification of Protein S-Acylation by IodoTMT0 Labeling and Immobilized Anti-TMT Antibody Resin Enrichment**. *J Proteome Res* 2023, **23**(2):673–683.
2. Ji G, Wu R, Zhang L, Yao J, Zhang C, Zhang X, Liu Z, Liu Y, Wang T, Fang C *et al*: **Global Analysis of Endogenously Intact S-Acylated Peptides Reveals Localization Differentiation of Heterogeneous Lipid Chains in Mammalian Cells**. *Anal Chem* 2023, **95**(35):13055–13063.
3. Freyermuth C, Dupuy JW, Renault TT, Claverol S, Raymond AA, Thinon E: **A Chemical Proteomics Method to Quantify Cysteine S-Acylation**. *ACS Chem Biol* 2026, **21**(2):312–325.
4. Thinon E, Fernandez JP, Molina H, Hang HC: **Selective Enrichment and Direct Analysis of Protein S-Palmitoylation Sites**. *J Proteome Res* 2018, **17**(5):1907–1922.
5. Forrester MT, Egol JR, Ozbay S, Waddell FD, Singh R, Tata PR: **Topology-driven discovery of transmembrane protein S-palmitoylation**. *J Biol Chem* 2025, **301**(3):108259.
6. Keerthikumar S, Chisanga D, Ariyaratne D, Al Saffar H, Anand S, Zhao K, Samuel M, Pathan M, Jois M, Chilamkurti N *et al*: **ExoCarta: A Web-Based Compendium of Exosomal Cargo**. *J Mol Biol* 2016, **428**(4):688–692.
7. Reales-Calderon JA, Vaz C, Monteoliva L, Molero G, Gil C: **Candida albicans Modifies the Protein Composition and Size Distribution of THP-1 Macrophage-Derived Extracellular Vesicles**. *J Proteome Res* 2017, **16**(1):87–105.
